# One-pot functionalisation of *N*-substituted tetrahydroisoquinolines by photooxidation and tunable organometallic trapping of iminium intermediates

**DOI:** 10.3762/bjoc.10.316

**Published:** 2014-12-12

**Authors:** Joshua P Barham, Matthew P John, John A Murphy

**Affiliations:** 1 WestCHEM, Department of Pure and Applied Chemistry, University of Strathclyde, 295 Cathedral Street, Glasgow G1 1XL, United Kingdom; 2 GlaxoSmithKline Medicines Research Centre, Gunnels Wood Road, Stevenage, Hertfordshire SG1 2NY, United Kingdom

**Keywords:** iminium salt, organometallic, oxidative functionalisation, photoredox catalysis, tetrahydroisoquinoline

## Abstract

Nucleophilic trapping of iminium salts generated via oxidative functionalisation of tertiary amines is well established with stabilised carbon nucleophiles. The few reports of organometallic additions have limited scope of substrate and organometallic nucleophile. We report a novel, one-pot methodology that functionalises *N*-substituted tetrahydroisoquinolines by visible light-assisted photooxidation, followed by trapping of the resultant iminium ions with organometallic nucleophiles. This affords 1,2-disubstituted tetrahydroisoquinolines in moderate to excellent yields.

## Introduction

**Figure 1:**
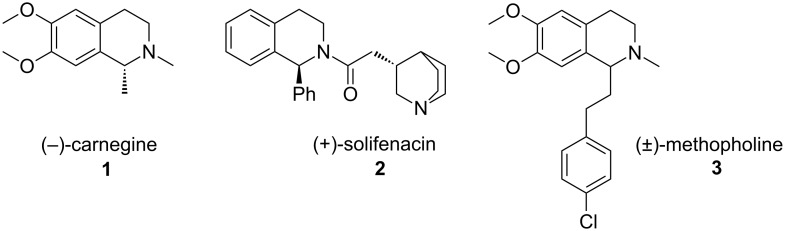
Examples of biologically active 1,2-disubstituted tetrahydroisoquinolines.

Tetrahydroisoquinolines (THIQs) are structural motifs prominent within biologically active natural products and pharmaceutical compounds [1–2]. From (−)-carnegine (**1**, a monoamine oxidase A inhibitor) [3] to (+)-solifenacin (**2**, a bladder-selective muscarinic M_3_ receptor antagonist) [4] to (±)-methopholine (**3**, an opioid analgesic) [5–6], a 1,2-disubstituted THIQ motif occurs throughout (Figure 1).

**Scheme 1:**
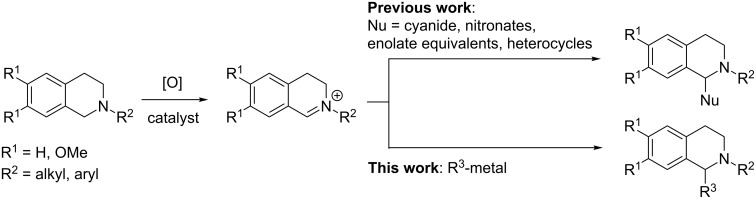
Oxidative C–H functionalisation and examples of previously reported nucleophilic trappings.

Environmental consciousness has initiated the development of methods which construct THIQs using green technologies, in mild conditions and with high atom efficiencies. Catalytic oxidative functionalisation of the C–H bond α- to the amine function is one such methodology. Iminium salts generated in this way can be intercepted by a nucleophile in a one-pot reaction (Scheme 1). Alternatively, the α-amino radicals can be trapped by electrophiles [7–9]. Oxidative C–H functionalisation of THIQs is reported using Cu(I) [10–11], Fe(III) [12], V(IV) [13] and I_2_ [14–15] catalysts, but also with heterogeneous [16], metal-organic [17–18] and organic [19–20] photocatalysts. Such catalysts are used in combination with various stoichiometric oxidants including oxygen [16,21].

However, nucleophilic trappings of resultant iminium salts are typically exemplified with highly stabilised carbon nucleophiles such as cyanides, nitronates, enolate equivalents and heterocycles (Scheme 1) [14,22–24]. Reports of organometallic additions to THIQs in this context are limited to aryl [25–27] and alkynyl [22,28–30] nucleophiles and the substrate scope is generally limited to *N*-aryl THIQs [31]. However, Li reported a hypervalent iodine mediated *N*-aryl THIQ oxidation which tolerated a wide range of organometallic nucleophiles [32] and Yu developed methodology for THIQ alkynylation which does not require *N*-aryl motifs [33].

We sought a general procedure for organometallic trapping of iminium salts generated by oxidative functionalisation; a methodology amenable to a range of tertiary amine substrates and unstabilised carbon nucleophiles. Recently, visible-light photoredox catalysis has gained interest as a technique for oxidative functionalisation [34–35]. An important feature of photoredox catalysis is that different photocatalysts have different redox potentials upon accessing the excited state [18,34,36]. The ability to adjust oxidising power through photocatalyst choice renders the transformation substrate-tunable. Thus, we selected photoredox catalysis as an oxidative functionalisation whose substrate scope might be extended (by catalyst selection) in future investigations. Herein, we report a one-pot, tandem visible-light powered oxidative functionalisation of *N*-substituted THIQs and organometallic trapping of iminium intermediates.

## Results and Discussion

Initially, a blue LED strip (λ_max_ = 458 nm), Ru(bpy)_3_(PF_6_)_2_ (1 mol %) and BrCCl_3_ (3.0 equiv) facilitated oxidation of *N*-phenyl THIQ (**4a**) (1 mmol) to its corresponding iminium salt (**5a**, Table 1) in anhydrous MeCN. As observed by Stephenson [37], photoredox activation of **4a** under these conditions required long reaction times (14–16 h) to reach full conversion. As reactions progressed, we observed precipitation and were able to collect half (by mass) of the crude iminium salt **5a** by filtration. Precipitation acts to stall reactions by shielding the photocatalyst from the light. Addition of vinylmagnesium bromide directly to the reaction mixture led to a complex mixture of products by HPLC.

**Table 1:** Organometallic additions to iminium salts generated via visible-light photoredox catalysis.

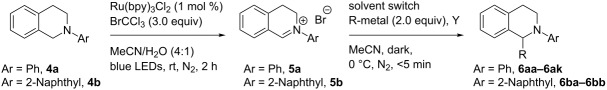					

Entry	R-Metal	Y	R	Product	Yield^a^

**1**	RMgBr^b^	–	vinyl	**6aa**	80
**2**	RMgBr^b^	–	Me	**6ab**	78
**3**	RMgCl^b^	–	Et	**6ac**	75
**4**	RMgCl^b^	–	iPr	**6ad**	78
**5**	RMgBr^b^	–	cyclopropyl	**6ae**	66
**6**	RMgBr^b,c^	–	Bn	**6af**	69
**7**	RMgBr^b^	–	Ph	**6ag**	90
**8**	RMgBr^b^	CuBr^d^	Ph	**6ag**	77
**9**	RMgBr^b^	–	4-FC_6_H_4_	**6ah**	72
**10**	RMgBr^b^	–	4-MeOC_6_H_4_	**6ai**	62
**11**^e^	RMgBr^b^	–	allyl	**6aj**	–
**12**^f^	RTMS	–	allyl	**6aj**	–
**13**	RMgBr^b^	ZnCl_2_^g^	allyl	**6aj**	37, 88^h^
**14**	RI	In^i^	allyl	**6aj**	92, 68^j^
**15**^e^	RMgBr^b^	–	2-methylallyl	**6ak**	–
**16**	RMgBr^b^	ZnCl_2_^g^	2-methylallyl	**6ak**	90
**17**	RMgCl^b^	ZnCl_2_^g^	2-butenyl	**6al**^k^	92

^a^Isolated (%) yields after chromatography. ^b^Commercially available solutions in THF or Et_2_O. ^c^6 equiv used. ^d^Grignard (2.0 equiv) premixed with CuBr (2.6 equiv). ^e^Complex mixture. ^f^No reaction. ^g^Grignard (2.0 equiv) premixed with a solution of ZnCl_2_ (2.6 equiv). ^h^Solvent switched to THF. ^i^Allyl iodide (3.0 equiv) premixed with In powder (2.0 equiv). ^j^Direct addition of R-metal without solvent switch. ^k^**6al** is a 1:1 mixture of diastereomers where R = 1-methyl-2-propenyl, see Supporting Information File 1.

We found that BrCCl_3_ and its byproduct CHCl_3_ [22] were responsible for poor organometallic reaction profiles. Generation of radical intermediates or carbenes upon reacting Grignard reagents or magnesium salts with BrCCl_3_ or CHCl_3_ are evidenced in the literature [38–39]. Initially, we took advantage of the volatilities of BrCCl_3_ and CHCl_3_ by removing them under vacuum and replacing the solvent. A solvent switch was also reported when photoredox activation of **4a** was combined with thiourea-catalysed enantioselective alkylation [37]. The enantioselectivity of the thiourea-catalysed alkylation was optimal in non-polar solvents, yet low photocatalyst solubility in these solvents precluded photoactivation of **4a**. Thus, a solvent switch was used to capitalise on the beneficial properties of both solvents.

At this stage, we employed an MeCN/H_2_O (4:1) solvent system and Ru(bpy)_3_Cl_2_ in photoactivations which facilitated full conversion of **4a** to **5a** in 2 h (Table 1). Here, MeCN/H_2_O (4:1) was chosen because MeCN forms an azeotrope with water [40] such that it could be easily dried by concentration. After dissolving resultant crude **5a** in anhydrous MeCN and shielding from ambient light, alkyl, aryl and vinyl Grignard reagents added virtually instantaneously to **5a**, affording **6aa**–**6ai** in good to excellent (62–90%) yields (Table 1). In general, the enhanced electrophilicity of **5a** compared to MeCN results in faster reaction of the Grignard with **5a** despite the solvent (MeCN) being present in vast excess.

**Figure 2:**
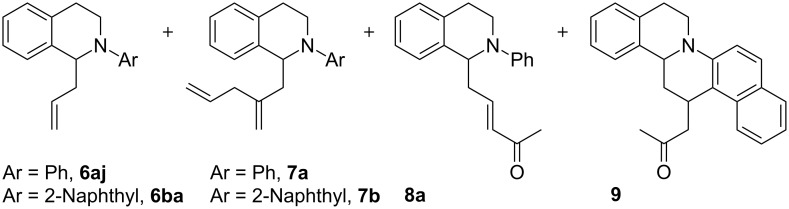
Products from allylzinc reagent addition to **5a** and **5b**.

Notably, allyl and 2-methylallyl Grignard additions (Table 1, entries 11 and 15) were exceptions and resulted in complex mixtures of products. We reasoned that use of a less reactive organometallic reagent would supress undesirable pathways. However, allyltrimethylsilane does not react with **5a** (Table 1, entry 12) [16,22]. As organometallics of intermediate reactivity, allylzinc halides were explored. Indeed, 2-methylallylzinc and 2-butenylzinc reagents added to **5a** to afford **6ak** and **6al** in 90% and 92% isolated yields, respectively. Conversely, addition of the allylzinc reagent to **5a** afforded side-products **7a** and **8a** in addition to **6aj** in a 1:4:4 ratio by LC–MS, respectively (Figure 2).

First, we sought to rule out the possibility of Ru(bpy)_3_Cl_2_ promoting these undesired pathways. Although Ru(bpy)_3_Cl_2_ could not be separated from **5a** after photoactivation due to their similar polarities, we successfully separated the less polar iminium salt **5b**. In absence of Ru(bpy)_3_Cl_2_, addition of the allylzinc reagent to **5b** gave **6ba** and side-products **7b** and **9** (Figure 2), and we now explored the origin of these byproducts.

The possibility of **6aj** or **6ba** being intermediates in these side-reactions was ruled out when **6aj** was exposed to the allylzinc reagent and no reaction was observed. When the allylzinc reagent was premixed (1:1) with MeCN before adding to **5a**, **6aj** was not observed. Instead, enone **8a** was observed as the sole product. Conversely, when the allylzinc reagent was added to **5a**, suspended in anhydrous THF, **8a** was not observed. The ratio of **6aj**:**7a** was 8:1 by LC–MS and an 88% yield of **6aj** resulted.

**Figure 3:**
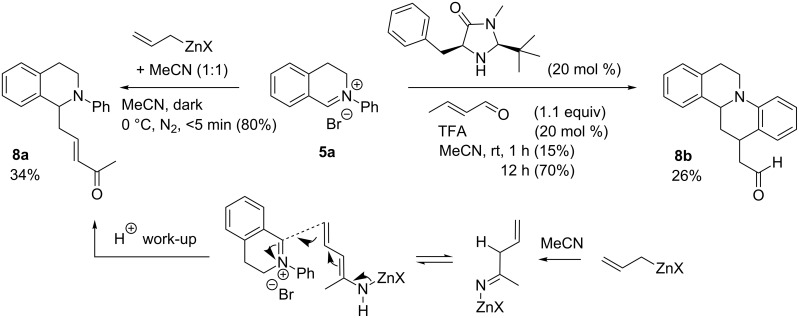
Proposed mechanism for formation of side-product **8a**. Analogous reactivity in the formation of cyclic product **8b** under enamine catalysis. LC–MS (%) yields in parenthesis. Isolated yields, %, after chromatography not in parenthesis.

We propose that allylzinc reagents are reactive enough to trap MeCN in competition with **5a**. The allylzinc reagent adds to MeCN to form an imine salt that is transformed in situ into a conjugated dienamine intermediate (Figure 3). Vinylogous nucleophilic addition of the enamine to **5a** generates **8a**. This hypothesis is supported by the reaction of **5a** with crotonaldehyde in the presence of a MacMillan-type imidazolidinone catalyst [41] and TFA which delivers **8b**. Formation of cyclic products **8b** and **9** is rationalised by intramolecular electrophilic aromatic substitution at the 2-position of the *N*-aryl moiety. (The isolation of enone **8a** and the fact that **5a** does not react with oct-1-ene under the same conditions rules out a Diels–Alder-type pathway to **8b**.)

**Figure 4:**
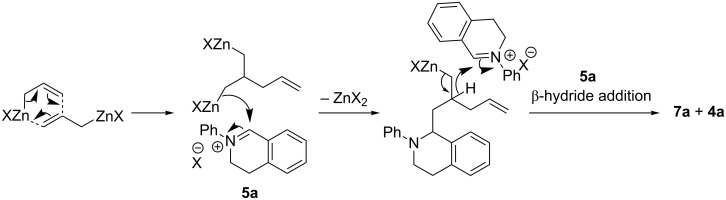
Mechanism for dimerisation of the allylzinc halide and β-hydride addition to **5a** [36].

Formation of side-products **7a** and **7b** can be rationalised by dimerisation of allylzinc halides as has been previously reported [42]. The authors describe generation of a bis-organozinc species which, upon addition to an electrophile, generates an intermediate which can undergo β-hydride delivery to a second electrophile. In this case addition to **5a** generates an intermediate organozinc species which, following β-hydride delivery to a second iminium (**5a**), generates **7a** and **4a** (Figure 4). Heating the allylzinc halide to promote dimerisation [42], prior to addition to **5a** in THF, altered the ratio of **6aj**:**7a** from 8:1 to 3:2 by LC–MS. In further support of this mechanism, reduction of **5a** to **4a** was also observed (the ratio of **6aj**:**7a**:**4a** = 5:3:1). However, our observations cannot rule out β-hydride addition as the first step. Consistent with our observations, the authors report that the more sterically hindered 2-methylallylzinc and 2-butenylzinc halides do not dimerise even after 48 h under reflux [42].

To avoid the pathways outlined in Figure 3 and Figure 4, we sought a less reactive allyl organometallic than the allylzinc reagent. Notably, allylindium reagents have attracted attention for their tolerance to water [43]. Such reagents have mediated reactions where allyl Grignards and allylzinc reagents have failed [44]. Gratifyingly, an allylindium reagent [45] appeared inert to pathways available to the allyl Grignard and allylzinc reagent, affording a 92% yield of **6aj**. Strikingly, the same reagent was added without a solvent switch and tolerated BrCCl_3_, CHCl_3_ and water, affording **6aj** albeit in lower yield (68%).

Murthy and Blechert and their respective co-workers reported allylation of THIQs under aerobic conditions using allyltrialkylstannanes [12,16] (Blechert’s studies also included success with allylboron reagents). Whilst our reactions are carried out under N_2_, the indium metal used, allylindium reagents generated and indium trihalide salt byproducts are non-toxic [43]. Our conditions benefit from the absence of amide side-products typically effected by peroxide intermediates in aerobic photoactivation of THIQs [16,22] and so our methodology serves to complement existing strategies in the literature.

The substrate scope of our methodology is outlined in Table 2. Iminium salts derived from a range of electronically diverse *N*-aryl THIQs (**4b** and **11a**–**14b**) were trapped with PhMgBr to afford products (**6bb** and **11b**–**14b** in fair to excellent (47–95%) yields. Substrates with both electron-rich (**12a**) and electron-poor (**13a**,**14a**) *N*-aryl substituents were tolerated. Although Ru(bpy)_3_Cl_2_ was ineffective at catalysing oxidation of *N*-Boc protected THIQ **17a**, we are pleased to report the first examples of Ru(bpy)_3_Cl_2_ catalysed oxidative functionalisation of *N*-alkyl THIQs. Subjecting **15a** and **16a** to photoactivation for 16 h furnished in both cases their corresponding benzylic *endo*-iminium salts, which were trapped by PhMgBr to afford **15b** and **16b** in 58% and 81% yield, respectively.

**Table 2:** Substrate scope of organometallic additions to iminium salts generated via visible-light photoredox catalysis.

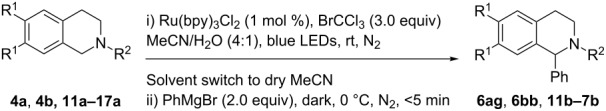				

Entry	R^1^/R^2^	Substrate	Product	Yield^a^

**1**^b,c^	H/Ph	**4a**	**6ag**	90
**2**^d^	H/2-Naphthyl	**4b**	**6bb**	47
**3**^c^	OMe/Ph	**11a**	**11b**	95
**4**^c^	H/4-MeOC_6_H_4_	**12a**	**12b**	52
**5**^c^	H/4-BrC_6_H_4_	**13a**	**13b**	53
**6**^c^	H/4-NO_2_C_6_H_4_	**14a**	**14b**	77
**7**^e^	H/Me	**15a**	**15b**	58
**8**^e^	OMe/Me	**16a**	**16b**	81
**9**^f^	H/CO_2_*t*-Bu	**17a**	**17b**	–

^a^Isolated (%) yields after chromatography. ^b^Entry 7, Table 1 given for comparison. ^c^Photoactivation time of 2 h. ^d^Photoactivation time of 4 h. ^e^Photoactivation time of 16 h. ^f^No reaction/photoactivation observed.

**Scheme 2:**
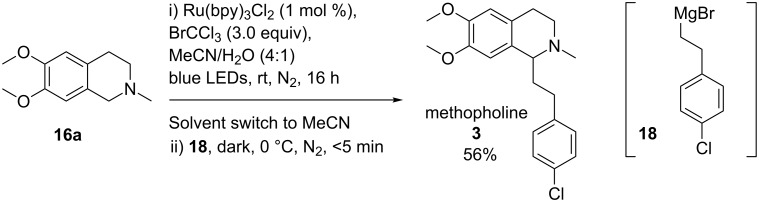
A concise synthesis of methopholine (**3**).

Encouraged by these results, we decided to apply our methodology to THIQ **16a** using Grignard **18** in a synthesis of methopholine (**3**, Scheme 2). Previous syntheses of **3** involving oxidative functionalisation of **16a** have used (4-chlorophenyl)acetylene as a pronucleophile [13,46]. However, isolation and hydrogenation of the resulting THIQ intermediate are required to access **3**. Photoactivation of **16a** and trapping of the resultant benzylic *endo*-iminium salt with **18** resulted in a 56% yield of **3**. To our knowledge, this concise synthesis of **3** is higher yielding (based on THIQ **16a**) than previously reported syntheses [13,46–47] with no intermediate isolations required.

Revisiting the concept of direct organometallic addition after photoactivation (thus far precluded by the use of BrCCl_3_), compatibility might be accomplished in two ways. Firstly, moderate the reactivity of the organometallic to tolerate BrCCl_3_ or secondly, find alternative oxidants which tolerate the organometallic. Whilst the former looked promising with the allylindium example, the latter approach was thought to be more general in terms of increasing nucleophile scope.

Stephenson reported diethyl bromomalonate as an effective oxidant to regenerate Ru(II) [22]. General application of this alkyl halide oxidant was ruled out due to potential side-reactions of the malonyl radical and diethyl malonate. We explored alternative alkyl halide oxidants that would form inert byproducts. No reaction was observed when substituting BrCCl_3_ with ClCH_2_CN (−0.72 V vs SCE [48]) but to our delight, BrCH_2_CN (−0.60 V vs SCE [48]) resulted in near-quantitative (90%) conversion of **4a** to **5a** in 3 h (anhydrous conditions). According to the mechanism proposed by Stephenson for BrCCl_3_ [22], BrCH_2_CN forms MeCN as an inert product. Grignard additions were unaffected by traces of residual BrCH_2_CN and a selection of substrates (**4a**, **11a**–**13a**) and Grignard reagents were employed, affording the products (**6aa**, **6ab**, **6ag**, **11b**–**13b**) in encouraging (50–77%) yields (Table 3).

**Table 3:** Direct one-pot organometallic additions to iminium salts generated via visible-light photoredox catalysis.

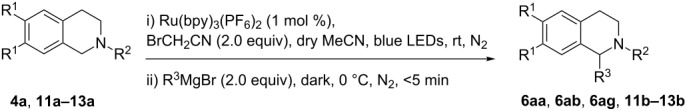				

Entry	R^1^/R^2^/R^3^	Substrate	Product	Yield^a^

**1**^b^	H/Ph/vinyl	**4a**	**6aa**	77
**2**^b^	H/Ph/Me	**4a**	**6ab**	73
**3**^b^	H/Ph/Ph	**4a**	**6ag**	61
**4**^b^	OMe/Ph/Ph	**11a**	**11b**	72
**5**^b^	H/4-MeOC_6_H_4_/Ph	**12a**	**12b**	73
**6**^c,d^	H/4-BrC_6_H_4_/Ph	**13a**	**13b**	50

^a^Isolated (%) yields after chromatography. ^b^Photoactivation time of 3 h. ^c^Photoactivation time of 5 h. ^d^Heating required to solubilise substrate.

## Conclusion

We have developed a user-friendly, one-pot methodology which combines visible-light photoredox catalysis and organometallic addition to deliver 1,2-disubstituted THIQs. It is rapid, performed under practical conditions and custom-made or commercially available organometallic solutions can be used directly. Highly reactive carbon nucleophiles (for example, allyl) have been harnessed by varying the organometallic species. Overall, this methodology is synthetically valuable for two reasons. Firstly, a virtually limitless host of carbon nucleophiles may be employed via organometallic chemistry (compounds **6aa**–**6af** are novel compounds derived from unstabilised carbon nucleophiles). Secondly, photoredox catalysis can be substrate tailored through photocatalyst selection. Having demonstrated the former reason herein, investigation of the latter is underway to extend the substrate scope beyond benzylic tertiary amines.

## Supporting Information

File 1 Experimental procedures, ^1^H and ^13^C spectra of all novel compounds and HPLC/LC–MS data from which conclusions were drawn.

## References

[1] Antkiewicz-Michaluk L, Wąsik A, Michaluk J (2014). Neurotoxic Res.

[2] Scott J D, Williams R M (2002). Chem Rev.

[3] Bembenek M E, Abell C W, Chrisey L A, Rozwadowska M D, Gessner W, Brossi A (1990). J Med Chem.

[4] Naito R, Yonetoku Y, Okamoto Y, Toyoshima A, Ikeda K, Takeuchi M (2005). J Med Chem.

[5] Wanner K T, Praschak I, Höfner G, Beer H (1996). Arch Pharm.

[6] Cass L J, Frederik W S (1963). Am J Med Sci.

[7] Kohls P, Jadhav D, Pandey G, Reiser O (2012). Org Lett.

[8] Ruiz Espelt L, Wiensch E M, Yoon T P (2013). J Org Chem.

[9] Prier C K, MacMillan D W C (2014). Chem Sci.

[10] Li Z, Li C-J (2005). J Am Chem Soc.

[11] Boess E, Schmitz C, Klussmann M (2012). J Am Chem Soc.

[12] Kumaraswamy G, Murthy A N, Pitchaiah A (2010). J Org Chem.

[13] Jones K M, Karier P, Klussmann M (2012). ChemCatChem.

[14] Dhineshkumar J, Lamani M, Alagiri K, Prabhu K R (2013). Org Lett.

[15] Nobuta T, Tada N, Fujiya A, Kariya A, Miura T, Itoh A (2013). Org Lett.

[16] Möhlmann L, Blechert S (2014). Adv Synth Catal.

[17] Condie A G, González-Gómez J C, Stephenson C R J (2010). J Am Chem Soc.

[18] Tucker J W, Stephenson C R J (2012). J Org Chem.

[19] Hari D P, König B (2011). Org Lett.

[20] Rueping M, Vila C, Bootwicha T (2013). ACS Catal.

[21] Hu J, Wang J, Nguyen T H, Zheng N (2013). Beilstein J Org Chem.

[22] Freeman D B, Furst L, Condie A G, Stephenson C R J (2012). Org Lett.

[23] Zhao G, Yang C, Guo L, Sun H, Chen C, Xia W (2012). Chem Commun.

[24] Shi L, Xia W (2012). Chem Soc Rev.

[25] Baslé O, Li C-J (2008). Org Lett.

[26] Muramatsu W, Nakano K, Li C-J (2013). Org Lett.

[27] Singh K N, Kessar S V, Singh P, Singh P, Kaur M, Batra A (2014). Synthesis.

[28] Li Z, Li C-J (2004). Org Lett.

[29] Fu W, Guo W, Zou G, Xu C (2012). J Fluorine Chem.

[30] Rueping M, Koenigs R M, Poscharny K, Fabry D C, Leonori D, Vila C (2012). Chem – Eur J.

[R31] 31There are two reports containing examples of *N*-alkyl THIQ oxidative functionalisation [27,30]; aryl [27] and alkynyl [30] organometallic nucleophiles are reported.

[32] Muramatsu W, Nakano K, Li C-J (2014). Org Biomol Chem.

[33] Zheng Q-H, Meng W, Jiang G-J, Yu Z-X (2013). Org Lett.

[34] Prier C K, Rankic D A, MacMillan D W C (2013). Chem Rev.

[35] Douglas J J, Nguyen J D, Cole K P, Stephenson C R J (2014). Aldrichimica Acta.

[36] Ravelli D, Fagnoni M, Albini A (2013). Chem Soc Rev.

[37] Bergonzini G, Schindler C S, Wallentin C-J, Jacobsen E N, Stephenson C R J (2014). Chem Sci.

[38] Kharasch M S, Reinmuth O, Urry W H (1947). J Am Chem Soc.

[39] Davis M, Deady L W, Finch A J, Smith J F (1973). Tetrahedron.

[40] Horsley L H (1947). Anal Chem.

[41] Paras N A, MacMillan D W C (2002). J Am Chem Soc.

[42] Courtois G, Miginiac L (1973). J Organomet Chem.

[43] Shen Z-L, Wang S-Y, Chok Y-K, Xu Y-H, Loh T-P (2013). Chem Rev.

[44] Lee K, Lee P H (2008). Org Lett.

[45] Araki S, Shimizu T, Johar P S, Jin S J, Butsugan Y (1991). J Org Chem.

[46] Singh K N, Singh P, Kaur A, Singh P (2012). Synlett.

[47] Richter H, Fröhlich R, Daniliuc C-G, García Mancheño O (2012). Angew Chem, Int Ed.

[48] Isse A A, Lin C Y, Coote M L, Gennaro A (2011). J Phys Chem B.

