# Supplementary material for: One-pot functionalisation of N-substituted tetrahydroisoquinolines by photooxidation and tunable organometallic trapping of iminium intermediates
Source: Beilstein J Org Chem. 2014 Dec 12;10:2981–8. doi: 10.3762/bjoc.10.316 (PMC4273301; doi:10.3762/bjoc.10.316)
Supplement: File 1 — Experimental procedures, 1H and 13C spectra of all novel compounds and HPLC/LC–MS data from which conclusions were drawn. [file Beilstein_J_Org_Chem-10-2981-s001.pdf]

Supporting Information

for

One-pot functionalisation of *N*-substituted tetrahydroisoquinolines by photo-oxidation and tunable organometallic trapping of iminium intermediates

Joshua P. Barham[1,2], Matthew P. John*[2] and John A. Murphy*[1]

Address: [1]WestCHEM, Department of Pure and Applied Chemistry, University of Strathclyde, 295 Cathedral Street, Glasgow G1 1XL, United Kingdom and [2]GlaxoSmithKline Medicines Research Centre, Gunnels Wood Road, Stevenage, Hertfordshire SG1 2NY, United Kingdom

Email: Joshua P. Barham - joshua.x.barham@gsk.com; Matthew P. John* - matthew.p.john@gsk.com; John A. Murphy* - john.murphy@strath.ac.uk

*Corresponding author

Experimental procedures, ^{1}H and ^{13}C spectra of all novel compounds and HPLC/LC–MS data from which conclusions were drawn

TABLE OF CONTENTS

1. General Experimental: S2
2. Reaction Apparatus: S2
3. General Procedure for Photoactivation of *N*-Substituted Tetrahydroisoquinolines S3
4. Procedures for Preparation of Organometallic Reagents S3
5. General Procedure for Organometallic Trapping of Iminium Intermediates S4
6. ^{1}H and ^{13}C NMR Spectra for All Novel Compounds..S19
7. Supporting data from which conclusions were drawn S69
8. References S71

1. GENERAL EXPERIMENTAL:

Unless specified otherwise, reactions were carried out under an inert (N_2) atmosphere. Cryogenic conditions (−78 °C) were achieved using dry ice/acetone baths. Temperatures of 0 °C were obtained by means of an ice bath. 'Room temperature' (rt) indicates temperatures in the range of 20-25 °C. HPLC and LC–MS were conducted using an Agilent™ 1100 instrument or Waters® ZQ 2000 instrument fitted with a Kinetex™ C18 column or Phenomenex Luna® C18 column, respectively. In both cases, a gradient of MeCN/H_2O with 0.05%(v/v) trifluoroacetic acid was used as eluant. For purposes of thin layer chromatography (tlc), POLYGRAM® SIL G/$_{UV254}$ silica plates were used, with UV light (λ = 254 nm) and potassium permanganate used for visualisation. Purification was achieved by column chromatography, either manually using Fluka analytical grade silica gel or, using a BIOTAGE® instru-ment with BIOTAGE® SNAP HP-Sil cartridges 25g, 50g, 100g and 300g. In some cases, purification was achieved by preparative LC–MS using a Waters® ZQ micromass ZQ 2000 instrument or, 'Mass Directed Autoprep' fitted with a Xbridge™ C18 column, and a gradient of MeCN/10 mM ammonium bicarbonate in H_2O. Removal of solvents (*in vacuo*) was achieved using Büchi® rotary evaporators. All NMR data were collected using a Bruker Avance™ 400 Ultrashield instrument. Data were manipulated using ACD/SpecManager© version 12.5. Reference values for residual solvents were taken as δ = 7.27 ($CDCl_3$) and 2.50 ppm (DMSO-d_6) for $_1$H NMR; δ = 77.16 ppm ($CDCl_3$) for ^{13}C NMR. Multiplicities for coupled signals were denoted as: s = singlet, d = doublet, t = triplet, q = quartet, m = multiplet, br. = broad, apt. = apparent and dd = double doublet *etc.* Coupling constants (*J*) are given in Hz and are uncorrected. Where appropriate, COSY, DEPT, HSQC, HMBC and ROESY experiments were car-ried out to aid assignment. High Resolution Mass spectral analyses were carried out at ESPRC National Mass Spectrometry Service Centre in Swansea on a LTQ Orbitrap XL using Atmospheric Pressure Chemical Ionisation (APCI) or High Resolution Nano-Electrospray (HNESP) using Electrospray Ionisation (ESI); masses observed are accurate to within 5 ppm. Infrared data were collected using a PerkinElmer® Spectrum 100 FTIR spectrometer as a thin film unless otherwise stated. Melting points are uncorrected and were recorded using a Stuart® Scientific SMP40 system. All solvents and reagents were purchased from Sigma-Aldrich and used as supplied. Except for (4-chlorophenethyl)magnesium bromide, all Grignard reagents were purchased from Sigma-Aldrich as solutions in THF or Et_2O. 2-Aryl-1,2,3,4-tetrahydroisoquinoline derivatives,[1,2,3] 2-methyl-1,2,3,4-tetrahydroisoquinoline de-rivatives[4] and *tert*-butyl 3,4-dihydroisoquinoline-2(1*H*)-carboxylate[5] were prepared by published literature proce-dures.

2. REACTION APPARATUS:

Visible light photochemical reactions were achieved using a Radleys® Carousel 12 Plus, positioned atop a mag-netic stirrer (Figure 1). A 2.5 m length of a 25 W blue LED strip (5050 SMD LEDs, λ_{max} = 458 nm) was wrapped around the inside pillars of the carousel resulting in ca. 5.0 cm distance between the LED strip and reaction ves-sels. The wells within the heating element were plugged and rare earth magnetic stirrer bars utilised to achieve effective stirring. Reactions were performed in Radleys® (RR91080 quick-thread glass reaction tubes for ca. 6 mmol and RR91088 quick-thread reduced volume for ca. 1 mmol) reaction vessels with lids (PTFE quick-thread) fitted with a gas tap and rubber septum. Degassing was achieved using 3 x freeze/pump/thaw cycles. Reaction vessels were triple evacuated/N_2 filled after sample taking.

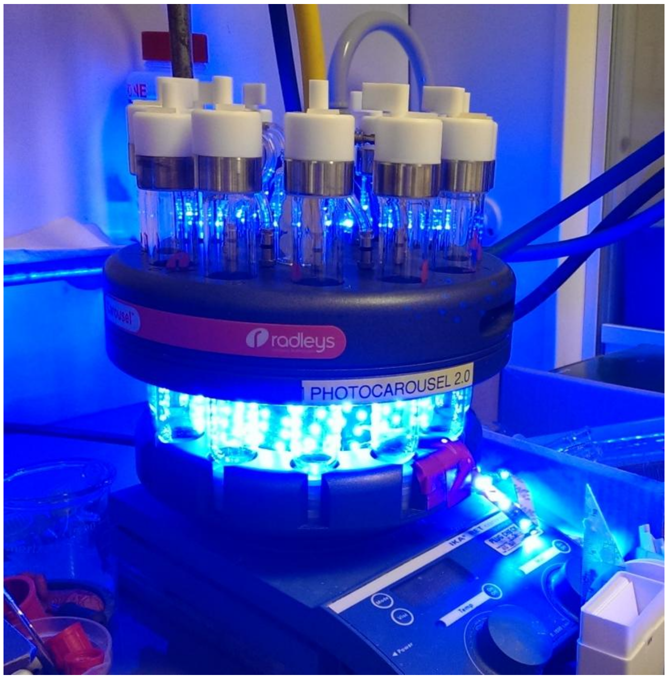

Figure 1: Parallel reactor setup for visible light photochemistry using 25W LED strip

3. General Procedure for Photoactivation of *N*-Substituted Tetrahydroisoquinolines

A reaction vessel was charged with *N*-phenyl-1,2,3,4-tetrahydroisoquinoline (**4a**) (1.00 mmol, 1.0 eq.), $Ru(bpy)_3Cl_2.6H_2O$ (10.0 μmol, 0.01 eq.), MeCN (6 mL) and H_2O (1.5 mL). The flask was degassed (3x freeze/pump/thaw) before $BrCCl_3$ (3.00 mmol, 3.0 eq.) was added at rt. The mixture was then placed within the aforementioned photochemical reactor (Figure 1) and irradiated with blue LEDs whilst under N_2. After starting material was consumed as determined by HPLC (2 h), the reaction mixture was concentrated to dryness and re-dissolved in the minimal amount of MeCN. Toluene (10 mL) was added and the reaction mixture was concentrated to dryness. DCM (10 mL) was added and the reaction mixture concentrated *in vacuo* to yield crude **5a** as a brown solid, which was used without further purification. Alternatively the same procedure is used with $BrCH_2CN$ as the oxidant (2.00 mmol, 2.0 eq.), $Ru(bpy)_3(PF_6)_2$ (10.0 μmol, 0.01 eq.) as the catalyst and anhydrous MeCN (7.5 mL) as the solvent to allow organometallic reagents to be added directly.

4. Procedures for Preparation of Organometallic Reagents

Prior to use, all reaction vessels were dried overnight in a vacuum oven (>50 ^oC). All manipulations were performed under N_2. Monoorganozinc halides were prepared by addition of the appropriate Grignard reagent (2.0 eq.) to $ZnCl_2$ (1.9 M in THF, 2.6 eq.) at -78 ^oC, stirring for 30 min at -78 ^oC, then stirring for 30 min at rt. Monoorganocopper reagents were prepared by addition of the appropriate Grignard reagent (2.0 eq.) to CuBr (2.6 eq.) in anhydrous THF (ca. 1.0 mL/1.00 mmol CuBr) at -78 ^oC, stirring for 30 min at -78 ^oC, then stirring for 30 min at rt. The allylic indium sesquihalide was prepared by a literature procedure[6] using In powder (-100 mesh, 2.0 eq.) and allyl iodide (3.0 eq.). A solution of (4-chlorophenethyl)magnesium bromide (1.5 M in THF) was prepared by charging a reaction vessel with magnesium turnings (10.0 mmol, 10.0 eq.), anhydrous THF (8.0 mL) and a small crystal of I_2 before adding 4-chlorophenethyl bromide (7.70 mmol, 7.70 eq.) dropwise under N_2. A color change from light brown to colourless was apparent. After refluxing for 10 min under N_2 and cooling to rt, the resultant turbid solution was removed by syringe (5.25 mL, 1.5 M in THF) and used as necessary.

5. General Procedure for Organometallic Trapping of Iminium Intermediates

Following photoactivation of **4a** (1.00 mmol, 1.0 eq.) crude **5a** was dissolved in anhydrous MeCN (5 mL) and the reaction vessel was covered in foil. After cooling to 0 °C, phenylmagnesium bromide (1 M in THF) (2.00 mmol, 2.0 eq.) was added dropwise over 15 min under N_2. After consumption of **5a** as indicated by HPLC (< 5 min), sat. NH_4Cl (10 mL) was carefully added and the resulting mixture poured into a separatory funnel containing EtOAc (50 mL) and water (50 mL). The layers were separated and aqueous layer extracted with further EtOAc (2 x 50 mL). The combined organic layers were dried ($MgSO_4$), filtered and concentrated *in vacuo* to yield the crude product. The residue was purified by silica gel column chromatography in the solvent mixture indicated to afford the desired product.

2-Phenyl-1-vinyl-1,2,3,4-tetrahydroisoquinoline (6aa)

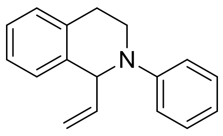

Following photoactivation of **4a** (197.0 mg, 0.94 mmol) according to the general procedure, crude **5a** was dissolved in anhydrous MeCN (5 mL). After cooling to 0 °C, vinylmagnesium bromide (1 M in THF) (1.90 mL, 1.90 mmol) was added dropwise over 15 min. Work up gave the crude product as a brown oil. Purification by flash column chromatography (a gradient of 10 - 20% DCM (1% Et_3N)/heptane) gave ***6aa*** as a yellow oil (176.2 mg, 80%); IR ν_{max} (neat) 3023 - 2908 (C-H), 1596 (Ar), 1502 (Ar), 1474, 1455, 1431, 1383, 1358, 1344, 1326, 1300, 1259, 1226 cm^{-1}; ^1H NMR (400 MHz, $CDCl_3$) δ 7.23 (2H, apt. t, *J* = 7.1 Hz, Ar*H*), 7.15 (4H, m, Ar*H*), 6.90 (2H, d, *J* = 8.1 Hz, Ar*H*), 6.73 (1H, t, *J* = 7.3 Hz, Ar*H*), 5.99 (1H, ddd, *J* = 17.0, 10.3, 5.1 Hz, C*H*=CH_2), 5.22 (1H, d, *J* = 4.9 Hz, C*H*N), 5.10 (2H, m, CH=CH_2), 3.58 (2H, apt. t, *J* = 6.0 Hz, CH_2CH_2N), 2.97 (1H, m, CH_2CH_2N), 2.90 (1H, m, CH_2CH_2N); ^{13}C NMR (101 MHz, $CDCl_3$) δ 149.7 (*C*), 138.7 (*C*H), 136.6 (*C*), 135.5 (*C*), 129.0 (*C*H), 128.3 (*C*H), 127.5 (*C*H), 126.7 (*C*H), 126.0 (*C*H), 117.8 (*C*H), 115.4 (*C*H_2), 114.5 (*C*H), 61.7 (*C*H), 42.9 (*C*H_2), 28.3 (*C*H_2); HRMS (+ESI) *m/z* calculated for $C_{17}H_{18}N$ [M+H$^+$] 236.1439; Found 236.1431.

Alternatively, following photoactivation of **4a** (205.0 mg, 0.98 mmol) with $BrCH_2CN$ (0.14 mL, 2.00 mmol) and $Ru(bpy)_3(PF_6)_2$ (8.4 mg, 9.8 μmol) in anhydrous MeCN (7.5 mL), the reaction mixture was cooled to 0 °C. To the reaction mixture was added vinylmagnesium bromide (1 M in THF) (2.00 mL, 2.00 mmol) dropwise over 15 min. Work up and purification as above gave ***6aa*** as a colourless oil (178.5 mg, 77%);

1-Methyl-2-phenyl-1,2,3,4-tetrahydroisoquinoline (6ab)

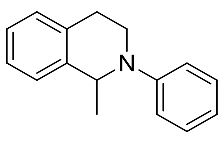

Following photoactivation of **4a** (197.0 mg, 0.94 mmol) according to the general procedure, crude **5a** was dissolved in anhydrous MeCN (5 mL). After cooling to 0 °C, methylmagnesium bromide (3.2 M in 2-MeTHF) (0.60 mL, 1.92 mmol) was added dropwise over 15 min. Work up gave the crude product as a brown oil. Purification by flash column chromatography (a gradient of 5 - 20% DCM (1% Et_3N)/heptane) gave ***6ab*** as a pale yellow oil (164.5 mg, 78%); IR ν_{max} (neat) 3022 - 2922 (C-H), 1597 (Ar), 1502 (Ar), 1447, 1386, 1328, 1287, 1266, 1228 cm^{-1}; ^1H NMR (400 MHz, $CDCl_3$) δ 7.22 (2H, apt. t, *J* = 8.0 Hz, Ar*H*), 7.18 - 7.12 (4H, m, Ar*H*), 6.92 (2H, d, *J* = 8.2 Hz, Ar*H*), 6.73 (1H, t, *J* = 7.3 Hz, Ar*H*), 4.90 (1H, q, *J* = 6.6 Hz, CH_3CHN), 3.65 - 3.60 (1H, m, CH_2CH_2N), 3.53 - 3.46 (1H, m, CH_2CH_2N), 3.07 - 2.99 (1H, m, CH_2CH_2N), 2.91 - 2.84 (1H, m, CH_2CH_2N), 1.43 (3H, d, *J* = 6.8 Hz, CH_3), ^{13}C NMR (101 MHz, $CDCl_3$) δ 149.7 (*C*), 140.2 (*C*), 134.5 (*C*), 129.2 (*C*H), 128.6 (*C*H), 126.7 (*C*H), 126.2

(*C*H), 125.9 (*C*H), 117.8 (*C*H), 115.0 (*C*H), 54.5 (*C*H), 41.4 (CH_2), 28.5 (CH_2), 20.7 (CH_3); HRMS (+ACPI) *m/z* calculated for $C_{16}H_{17}N$ [M+H$^+$] 224.1439; Found 224.1431.

Alternatively, following photoactivation of **4a** (207.0 mg, 0.99 mmol) with $BrCH_2CN$ (0.14 mL, 2.00 mmol) and $Ru(bpy)_3(PF_6)_2$ (8.1 mg, 9.4 μmol) in anhydrous MeCN (7.5 mL), the reaction mixture was cooled to 0 °C. To the reaction mixture was added methylmagnesium bromide (3.2 M in 2-MeTHF) (0.62 mL, 1.98 mmol) dropwise over 15 min. Work up and purification as above gave ***6ab*** as a colourless oil (161.2 mg, 73%);

1-Ethyl-2-Phenyl-1,2,3,4-tetrahydroisoquinoline (6ac)

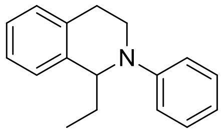

Following photoactivation of **4a** (197.0 mg, 0.94 mmol) according to the general procedure, crude **5a** was dissolved in anhydrous MeCN (5 mL). After cooling to 0 °C, ethylmagnesium chloride (2 M in THF) (0.95 mL, 1.90 mmol) was added dropwise over 15 min. Work up gave the product as a brown oil. Purification by flash column chromatography (a gradient of 5 - 20% DCM (1% Et_3N)/heptane) gave ***6ac*** as a pale yellow oil (166.3 mg, 75%); IR ν_{max} (neat) 3025 - 2929 (C-H), 1597 (Ar), 1502 (Ar), 1473, 1456, 1390, 1331, 1294, 1266, 1221 cm^{-1}; ^{1}H NMR (400 MHz, $CDCl_3$) δ 7.25 (2H, td, *J* = 8.7, 1.5 Hz, Ar*H*), 7.17 - 7.09 (4H, m, Ar*H*), 6.88 (2H, d, *J* = 8.2 Hz, Ar*H*), 6.71 (1H, t, *J* = 7.3 Hz, Ar*H*), 4.55 (1H, apt. t, *J* = 7.0 Hz, CH_3CH_2CHN), 3.65 - 3.53 (2H, m, CH_2CH_2N), 3.05 - 2.98 (1H, m, CH_2CH_2N), 2.90 - 2.83 (1H, m, CH_2CH_2N), 2.03 - 1.93 (1H, m, CH_3CH_2CHN) 1.80 - 1.69 (1H, m, CH_3CH_2CHN) 1.00 (3H, t, *J* = 7.4 Hz, CH_3CH_2CHN); ^{13}C NMR (101 MHz, $CDCl_3$) δ 150.1 (*C*), 139.0 (*C*), 135.0 (*C*), 129.2 (*C*H), 128.4 (*C*H), 127.4 (*C*H), 126.4 (*C*H), 125.7 (*C*H), 116.9 (*C*H), 113.6 (*C*H), 60.7 (*C*H), 42.0 (CH_2), 29.6 (CH_2), 27.3 (CH_2), 11.4 (CH_3); HRMS (+ACPI) *m/z* calculated for $C_{17}H_{20}N$ [M+H$^+$] 238.1596; Found 238.1589.

1-Isopropyl-2-phenyl-1,2,3,4-tetrahydroisoquinoline (6ad)

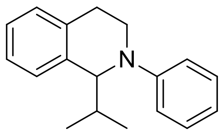

Following photoactivation of **4a** (197.0 mg, 0.94 mmol) according to the general procedure, crude **5a** was dissolved in anhydrous MeCN (5 mL). After cooling to 0 °C, isopropylmagnesium chloride (2 M in THF) (0.95 mL, 1.90 mmol) was added dropwise over 15 min. Work up gave the product as a brown oil. Purification by flash column chromatography (a gradient of 5 - 20% DCM (1% Et_3N)/heptane) gave ***6ad*** as a pale yellow oil (184.7 mg, 78%); IR ν_{max} (neat) 3100 - 2957 (C-H), 1596 (Ar), 1502 (Ar), 1472, 1392, 1365, 1344, 1323, 1297, 1278, 1221 cm^{-1}; ^{1}H NMR (400 MHz, $CDCl_3$) δ 7.20 - 7.05 (6H, m, Ar*H*), 6.86 (2H, d, *J* = 8.1 Hz, Ar*H*), 6.66 (1H, t, *J* = 7.2 Hz, Ar*H*), 4.39 (1H, d, *J* = 7.9 Hz, $(CH_3)_2CHCHN$), 3.75 - 3.69 (1H, m, CH_2CH_2N), 3.53 - 3.46 (1H, m, CH_2CH_2N), 2.96 (2H, apt. t, *J* = 6.4 Hz, CH_2CH_2N), 2.18 - 2.09 (1H, m, $(CH_3)_2CHCHN$), 1.06 (3H, d, *J* = 6.8 Hz, CH_3) 0.92 (3H, d, *J* = 6.6 Hz, CH_3); ^{13}C NMR (101 MHz, $CDCl_3$) δ 150.4 (*C*), 138.0 (*C*), 135.3 (*C*), 129.0 (*C*H), 128.2 (2 x *C*H), 126.5 (*C*H), 125.2 (*C*H), 116.9 (*C*H), 113.8 (*C*H), 64.8 (*C*H), 43.1 (CH_2), 34.4 (*C*H), 27.4 (CH_2), 20.6 (CH_3), 20.1 (CH_3); HRMS (+ACPI) *m/z* calculated for $C_{18}H_{22}N$ [M+H$^+$] 252.1752; Found 252.1743.

1-Cyclopropyl-2-phenyl--1,2,3,4-tetrahydroisoquinoline (6ae)

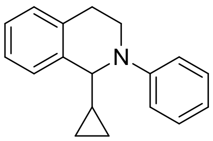

Following photoactivation of **4a** (192.0 mg, 0.92 mmol) according to the general procedure, crude **5a** was dissolved in anhydrous MeCN (5 mL). After cooling to 0 °C, cyclopropylmagnesium bromide (1 M in 2-MeTHF) was added dropwise over 15 min. Work up gave the product as a brown oil. Purification by flash column chromatography (a gradient of 10 - 20% DCM(1% Et_3N)/heptane) gave ***6ae*** as an orange microcrystalline solid (151.1 mg, 66%); m.p. 56 - 58 °C; IR ν_{max} (neat) 3052 - 2834 (C-H), 1592 (Ar), 1496 (Ar), 1463, 1451, 1427, 1385, 1352, 1315, 1259, 1226, 1211 cm^{-1}; ^1H NMR (400 MHz, $CDCl_3$) δ 7.24 - 7.19 (2H, m, Ar*H*), 7.17 - 7.11 (4H, m, Ar*H*), 6.93 (2H, d, *J* = 7.9 Hz, Ar*H*), 6.74 (1H, apt. t, *J* = 7.3 Hz, Ar*H*), 4.41 (1H, d, *J* = 5.3 Hz, $(CH_2)_2CHCHN$), 3.79 - 3.72 (1H, m, CH_2CH_2N), 3.63 - 3.57 (1H, m, CH_2CH_2N), 3.04 - 2.97 (1H, m, CH_2CH_2N), 2.89 - 2.84 (1H, m, CH_2CH_2N), 1.33 - 1.26 (1H, m, $(CH_2)_2CHCHN$), 0.54 - 0.42 (2H, m, $(CH_2)_2CH$) 0.33 - 0.23 (2H, m, $(CH_2)_2CH$); ^{13}C NMR (101 MHz, $CDCl_3$) δ 150.1 (*C*), 137,4 (*C*), 135.2 (*C*), 129.2 (*C*H), 128.5 (*C*H), 127.5 (*C*H), 126.7 (*C*H), 125.7 (*C*H), 117.8 (*C*H), 115.0 (*C*H), 61.9 (*C*H), 42.3 (CH_2), 27.5 (CH_2), 16.6 (*C*H), 3.9 (CH_2), 3.0 (CH_2); HRMS (+ACPI) *m/z* calculated for $C_{18}H_{20}N$ [$M+H^+$] 250.1596; Found 250.1591.

1-Benzyl-2-phenyl-1,2,3,4-tetrahydroisoquinoline (6af)

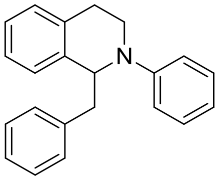

Following photoactivation of **4a** (198.0 mg, 0.95 mmol) according to the general procedure, crude **5a** was dissolved in anhydrous MeCN (5 mL). After cooling to 0 °C, benzylmagnesium bromide in THF (1 M) (5.70 mL, 5.70 mmol)* was added dropwise over 15 min. Work up gave the crude product as a brown oil. Purification by Mass Directed Autoprep gave ***6af*** as a yellow oil (194.9 mg, 69%); IR ν_{max} (neat) 3061 - 2919 (C-H), 1596 (Ar), 1502 (Ar), 1494, 1473, 1452, 1391, 1328, 1275, 1230 cm^{-1}; ^1H NMR (400 MHz, $CDCl_3$) δ 7.22 - 7.12 (7H, m, Ar*H*), 7.05 - 7.00 (3H, m, Ar*H*), 6.85 (2H, d, *J* = 7.9 Hz, Ar*H*), 6.74 - 6.70 (2H, m, Ar*H*), 4.89 (1H, apt. t, *J* = 6.4 Hz, ArC*H*N), 3.67 - 3.61 (1H, m, CH_2CH_2N), 3.56 - 3.50 (1H, m, CH_2CH_2N), 3.27 - 3.22 (1H, m, $PhCH_2CHN$), 3.02 - 2.94 (2H, m, $PhCH_2CHN$, CH_2CH_2N), 2.77 - 2.70 (1H, m, CH_2CH_2N); ^{13}C NMR (101 MHz, $CDCl_3$) δ 149.3 (*C*), 138.9 (*C*), 137.6 (*C*), 135.1 (*C*), 129.8 (*C*H), 129.2 (*C*H), 128.2 (*C*H), 128.1 (*C*H), 127.6 (*C*H), 126.6 (*C*H), 126.2 (*C*H), 125.5 (*C*H), 117.2 (*C*H), 113.7 (*C*H), 61.5 (*C*H), 42.4 (CH_2), 42.1 (CH_2), 27.5 (CH_2); HRMS (+ACPI) *m/z* calculated for $C_{22}H_{22}N$ [$M+H^+$] 300.1752; Found 300.1745.

*HPLC analysis revealed that the reaction did not go to completion with 2.0 eq. of benzylmagnesium bromide.

1,2-Diphenyl-1,2,3,4-tetrahydroisoquinoline (6ag)[7]

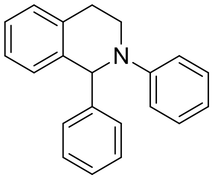

Following photoactivation of **4a** (197.0 mg, 0.94 mmol) according to the general procedure, crude **5a** was dissolved in anhydrous MeCN (5 mL). After cooling to 0 °C, phenylmagnesium bromide (1 M in THF) (1.90 mL, 1.90 mmol) was added dropwise over 15 min. Work up gave the crude product as a brown oil. Purification by flash column chromatography (a gradient of 5 - 10% DCM (1% Et_3N)/heptane) gave a yellow oil, which crystallised overnight to yield **6ag** as a microcrystalline orange solid (241.4 mg, 90%); m.p. 68 - 71 °C; IR ν_{max} (neat) 3022 - 2854 (C-H), 1593 (Ar), 1573 (Ar), 1504 (Ar), 1491, 1472, 1457, 1445, 1432, 1382, 1360, 1342, 1326, 1300, 1268, 1251, 1227, 1209 cm^{-1}; ^1H NMR (400 MHz, $CDCl_3$) δ 7.21 - 7.10 (11H, m, Ar*H*), 6.87 (2H, d, *J* = 8.0 Hz, Ar*H*), 6.72 (1H, t, *J* = 7.1 Hz, Ar*H*), 5.82 (1H, s, ArC*H*N), 3.70 - 3.64 (1H, m, CH_2CH_2N), 3.54 - 3.48 (1H, m, CH_2CH_2N), 2.98 - 2.84 (2H, m, CH_2CH_2N); ^{13}C NMR (101 MHz, $CDCl_3$) δ 149.6 (*C*), 143.2 (*C*), 137.9 (*C*), 135.8 (*C*), 129.2 (*C*H), 128.3 (*C*H), 128.2 (*C*H), 127.9 (*C*H), 127.4 (*C*H), 127.1 (*C*H), 126.8 (*C*H), 126.2 (*C*H), 117.5 (*C*H), 113.9 (*C*H), 62.9 (*C*H), 43.9 (*C*H_2), 28.1 (*C*H_2); HRMS (+ACPI) *m/z* calculated for $C_{21}H_{20}N$ [M+H^+] 286.1596; Found 286.1593.

Alternatively, following photoactivation of **4a** (198.0 mg, 0.95 mmol) according to the general procedure, crude **5a** was dissolved in MeCN (5 mL). After cooling to 0 °C, the organocopper reagent derived from phenylmagnesium bromide (1.90 mL, 1.90 mmol) and copper bromide (0.35 g, 2.44 mmol) was added dropwise over 15 min. Work up and purification as before gave **6ag** as a microcrystalline orange solid (208.9 mg, 77%).

Alternatively, following photoactivation of **4a** (204.0 mg, 0.98 mmol) with $BrCH_2CN$ (0.14 mL, 2.00 mmol) and $Ru(bpy)_3(PF_6)_2$ (8.8 mg, 10.2 μmol) in anhydrous MeCN (7.5 mL), the reaction mixture was cooled to 0 °C. To the reaction mixture was added phenylmagnesium bromide (1.0 M in THF) (2.00 mL, 2.00 mmol) dropwise over 15 min. Work up and purification as above gave **6ag** as a microcrystalline orange solid (170.4 mg, 61%);

Data consistent with literature.[7]

1-(4-Fluorophenyl)-2-phenyl-1,2,3,4-tetrahydroisoquinoline (6ah)[8]

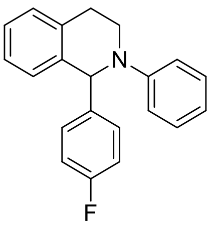

Following photoactivation of **4a** (192.0 mg, 0.92 mmol) according to the general procedure, crude **5a** was dissolved in anhydrous MeCN (5 mL). After cooling to 0 °C, 4-fluorophenylmagnesium bromide (1 M in THF) (1.85 mL, 1.85 mmol) was added dropwise over 15 min. Work up gave the crude product as a brown oil. Purification by flash column chromatography (a gradient of 5 - 10% DCM (1% Et_3N)/heptane) gave **6ah** as a yellow oil (199.6 mg, 72%); IR ν_{max} (neat) 3027 - 2916 (C-H), 1596 (Ar), 1503 (Ar), 1475, 1457, 1410, 1382, 1359, 1346, 1327, 1300, 1253, 1219 cm^{-1}; ^1H NMR (400 MHz, $CDCl_3$) δ 7.24 - 7.16 (8H, m, Ar*H*), 6.90 (2H, td, *J* = 8.6, 2.1 Hz, Ar*H*), 6.85 (2H, d, *J* = 8.1 Hz, Ar*H*), 6.76 (1H, apt. t, *J* = 7.3 Hz, Ar*H*), 5.79 (1H, s, ArC*H*N), 3.69 - 3.63 (1H, m, CH_2CH_2N), 3.51 - 3.45 (1H, m, CH_2CH_2N), 2.98 - 2.92 (1H, m, CH_2CH_2N), 2.89 - 2.82 (1H, m, CH_2CH_2N); ^{13}C NMR (101 MHz, $CDCl_3$) δ 161.8 (d, J_{C-F} = 245.3 Hz, *C*), 149.5 (*C*), 138.8 (d, J_{C-F} = 3.2 Hz, *C*), 137.6 (*C*), 135.7 (*C*), 129.2 (*C*H), 129.0 (d, J_{C-F} = 8.0 Hz, *C*H), 128.2 (*C*H), 127.8 (*C*H), 127.1 (*C*H), 126.2 (*C*H), 117.9 (*C*H), 115.0 (d, J_{C-F} = 21.4 Hz, *C*H), 114.2 (*C*H), 62.3 (*C*H), 43.7 (*C*H_2), 28.0 (*C*H_2); HRMS (+APCI) *m/z* calculated for $C_{21}H_{19}FN$ [M+H^+] 304.1502; Found 304.1500.

Data consistent with literature.[8]

1-(4-Methoxyphenyl)-2-phenyl-1,2,3,4-tetrahydroisoquinoline (**6ai**)[7]

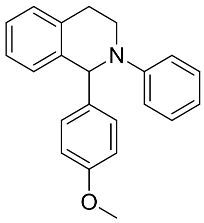

Following photoactivation of **4a** (192.0 mg, 0.92 mmol) according to the general procedure, crude **5a** was dissolved in anhydrous MeCN (5 mL). After cooling to 0 °C, 4-methoxyphenylmagnesium bromide (0.5 M in THF) (3.70 mL, 1.85 mmol) was added dropwise over 15 min. Work up gave the crude product as a brown oil. Purification by flash column chromatography (a gradient of 20 - 30% DCM (1% Et_3N)/heptane) gave **6ai** as a yellow oil (179.9 mg, 62%); IR ν_{max} (neat) 2908 - 2835 (C-H), 1596 (Ar), 1504 (Ar), 1474, 1463, 1441, 1383, 1360, 1345, 1328, 1301, 1246 cm^{-1}; ^1H NMR (400 MHz, $CDCl_3$) δ 7.24 - 7.12 (8H, m, Ar*H*), 6.87 (2H, d, *J* = 8.7 Hz, Ar*H*), 6.77 - 6.73 (3H, m, Ar*H*), 5.79 (1H, s, ArC*H*N), 3.74 (3H, s, OC*H*3), 3.71 - 3.65 (1H, m, CH_2CH_2N), 3.53 - 3.46 (1H, m, CH_2CH_2N), 2.99 - 2.85 (2H, m, CH_2CH_2N); ^{13}C NMR (101 MHz, $CDCl_3$) δ 158.5 (*C*), 149.7 (*C*), 138.1 (*C*), 135.7 (*C*), 135.2 (*C*), 129.1 (*C*H), 128.5 (*C*H), 128.2 (*C*H), 127.8 (*C*H), 126.9 (*C*H), 126.1 (*C*H), 117.5 (*C*H), 114.2 (*C*H), 113.5 (*C*H), 62.3 (*C*H), 55.2 (CH_3), 43.6 (CH_2), 28.0 (CH_2); HRMS (+APCI) *m/z* calculated for $C_{22}H_{22}NO$ [M+H$^+$] 316.1701; Found 316.1701.

Data consistent with literature.[7]

1-(2-Methylallyl)-2-phenyl-1,2,3,4-tetrahydroisoquinoline (**6ak**)[9]

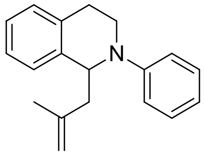

Following photoactivation of **4a** (198.0 mg, 0.95 mmol) according to the general procedure, crude **5a** was dissolved in anhydrous MeCN (5 mL). After cooling to 0 °C, the allylzinc reagent derived from 2-methylallylmagnesium bromide (0.5 M in Et_2O) (3.80 mL, 1.90 mmol) and zinc chloride (1.9 M in THF) (1.30 mL, 2.47 mmol) was added dropwise over 15 min. Work up gave the crude product as a brown oil. Purification by flash column chromatography (a gradient of 0 - 5% toluene/heptane (1% Et_3N)) gave **6ak** as a yellow oil (224.8 mg, 90%); IR ν_{max} (neat) 3024 - 2914 (C-H), 1646 (C=C), 1597 (Ar), 1502 (Ar), 1474, 1451, 1390, 1330, 1291, 1268, 1210 cm^{-1}; ^1H NMR (400 MHz, $CDCl_3$) δ 7.22 (3H, apt. t, *J* = 7.2 Hz, Ar*H*), 7.14 - 7.07 (4H, m, Ar*H*), 6.91 (1H, d, *J* = 8.0 Hz, Ar*H*), 6.72 (1H, t, J = 7.3 Hz, Ar*H*), 4.84 (1H, apt. t, *J* = 7.0 Hz, ArC*H*N), 4.80 (1H, br. s, CH_2=C), 4.66 (1H, br. s, CH_2=C), 3.62 (2H, apt. t, *J* = 6.2 Hz, CH_2CH_2N), 3.05 - 2.97 (1H, m, CH_2CH_2N), 2.85 - 2.79 (1H, m, CH_2CH_2N), 2.71 - 2.66 (1H, m, CH_2CHN), 2.42 - 2.36 (1H, m, CH_2CHN), 1.78 (3H, s, CH_3); ^{13}C NMR (101 MHz, $CDCl_3$) δ 149.5 (*C*), 142.9 (*C*), 138.5 (*C*), 134.7 (*C*), 129.2 (*C*H), 128.5 (*C*H), 127.4 (*C*H), 126.5 (*C*H), 125.6 (*C*H), 117.4 (*C*H), 114.3 (*C*H), 113.5 (CH_2), 58.2 (*C*H), 44.6 (CH_2), 41.7 (CH_2), 27.0 (CH_2), 22.9 (CH_3); HRMS (+ACPI) *m/z* calculated for $C_{19}H_{22}N$ [M+H$^+$] 264.1752; Found 264.1747.

Data consistent with literature.[9]

1-(but-3-en-2-yl)-2-phenyl-1,2,3,4-tetrahydroisoquinoline (*6al*)

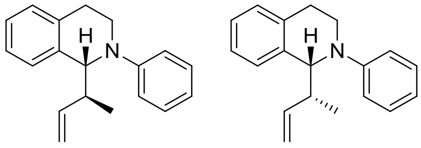 (a 1:1 mixture of diastereomers).

Following photoactivation of **4a** (198.0 mg, 0.95 mmol) according to the general procedure, crude **5a** was dissolved in anhydrous MeCN (5 mL). After cooling to 0 ^{o}C, the allylzinc reagent derived from 2-butenylmagnesium bromide (0.5 M in THF) (4.00 mL, 2.00 mmol) and zinc chloride (1.9 M in THF) (1.30 mL, 2.47 mmol) was added dropwise over 15 min. Work up gave the crude product as a brown oil. Purification by flash column chromatography (a gradient of 2 - 5% toluene/heptane (1% Et_3N)) gave ***6al*** as a yellow oil (229.1 mg, 92%); IR ν_{max} (neat) 3023 - 2923 (C-H), 1637 (C=C), 1574 (Ar), 1597 (Ar), 1502 (Ar), 1474, 1456, 1418, 1392, 1371, 1299, 1269, 1218 cm^{-1}; ^{1}H NMR (400 MHz, $CDCl_3$) <u>diastereomer 1:</u> δ 7.25 - 7.07 (6H, m, Ar*H*), 6.87 (2H, apt. d, *J* = 6.8 Hz, Ar*H*), 6.72 - 6.68 (1H, m, Ar*H*), 5.99 - 5.91 (1H, m, C*H*=CH$_2$), 5.05 - 4.87 (2H, m, C*H*=CH$_2$), 4.57 (1H, d, *J* = 7.4 Hz, CH(CH$_3$)C*H*N), 3.78 - 3.69 (1H, m, CH$_2$C*H*$_2$N), 3.50 - 3.42 (1H, m, CH$_2$C*H*$_2$N), 3.08 - 2.91 (2H, m, C*H*$_2$CH$_2$N), 2.79 - 2.70 (1H, m, C*H*(CH$_3$)CHN), 1.08 (3H, d, *J* = 6.6, CH(C*H*$_3$)CHN); <u>diastereomer 2:</u> δ 7.25 - 7.07 (6H, m, Ar*H*), 6.87 (2H, apt. d, *J* = 6.8 Hz, Ar*H*), 6.72 - 6.68 (1H, m, Ar*H*), 5.72 - 5.63 (1H, m, C*H*=CH$_2$), 5.05 - 4.87 (2H, m, C*H*=CH$_2$), 4.51 (1H, d, *J* = 6.8 Hz, CH(CH$_3$)C*H*N), 3.78 - 3.69 (1H, m, CH$_2$C*H*$_2$N), 3.50 - 3.42 (1H, m, CH$_2$C*H*$_2$N), 3.08 - 2.91 (2H, m, C*H*$_2$CH$_2$N), 2.79 - 2.70 (1H, m, C*H*(CH$_3$)CHN), 1.18 (3H, d, *J* = 7.0, CH(C*H*$_3$)CHN); ^{13}C NMR (101 MHz, $CDCl_3$) <u>diastereomer 1</u> and <u>diastereomer 2:</u> δ 149.9 (*C*), 149.8 (*C*), 142.0 (*C*H), 141.4 (*C*H), 137.3 (*C*), 136.7 (*C*), 135.5 (2 x *C*), 129.2 (*C*H), 129.1 (*C*H), 128.5 (*C*H), 128.3 (*C*H), 126.7 (2 x *C*H), 125.5 (*C*H), 125.2 (*C*H), 116.8 (*C*H), 116.8 (*C*H), 115.1 (*C*H$_2$), 113.9 (*C*H$_2$), 113.6 (*C*H), 113.2 (CH), 63.7 (2 x *C*H), 44.6 (*C*H), 43.9 (*C*H), 43.0 (*C*H$_2$), 42.9 (*C*H$_2$), 27.7 (*C*H$_2$), 27.4 (*C*H$_2$), 18.2 (*C*H$_3$), 16.9 (*C*H$_3$); HRMS (+ESI) *m/z* calculated for $C_{19}H_{22}N$ [M+H^{+}] 264.1747; Found 264.1747.

2-Phenyl-1-allyl-1,2,3,4-tetrahydroisoquinoline (6aj)[10]

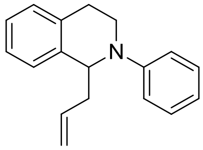

Following photoactivation of **4a** (198.0 mg, 0.95 mmol) according to the general procedure, crude **5a** was dissolved in anhydrous MeCN (5 mL). After cooling to 0 ^{o}C, the allylindium reagent derived from reaction of allyl iodide (0.25 mL, 2.75 mmol) and In powder (0.21 g, 1.83 mmol) in DMF (2.0 mL) was added dropwise over 15 min. Work up gave the crude product as a brown oil. Purification by flash column chromatography (a gradient of 10 - 20% DCM (1% Et_3N)/heptane) gave **6aj** as a pale yellow oil (217.6 mg, 92%); IR ν_{max} (neat) 3023 - 2835 (C-H), 1638 (C=C) 1596 (Ar), 1502 (Ar), 1473, 1451, 1390, 1344, 1327, 1297, 1267, 1222 cm^{-1}; ^{1}H NMR (400 MHz, $CDCl_3$) δ 7.24 (2H, apt. t, *J* = 8.1 Hz, Ar*H*), 7.17 - 7.10 (4H, m, Ar*H*), 6.90 (2H, d, *J* = 8.0 Hz, Ar*H*), 6.73 (1H, td, *J* = 7.3, 0.9 Hz, Ar*H*), 5.90 - 5.80 (1H, m, C*H*=CH$_2$), 5.08 - 5.03 (2H, m, CH=C*H*$_2$), 4.74 (1H, t, *J* = 6.8 Hz C*H*N), 3.67 - 3.56 (2H, m, CH$_2$C*H*$_2$N), 3.06 - 2.99 (1H, m, C*H*$_2$CH$_2$N), 2.91 - 2.84 (1H, m, C*H*$_2$CH$_2$N), 2.77 - 2.70 (1H, m, C*H*$_2$CHN), 2.52 - 2.45 (1H, m, C*H*$_2$CHN); ^{13}C NMR (101 MHz, $CDCl_3$) δ 149.4 (*C*), 138.1 (*C*), 135.7 (*C*H), 135.0 (*C*), 129.3 (*C*H), 128.5 (*C*H), 127.3 (*C*H), 126.6 (*C*H), 125.7 (*C*H), 117.3 (*C*H), 117.0 (*C*H$_2$), 114.0 (*C*H), 59.4 (*C*H), 42.0 (*C*H$_2$), 40.9 (*C*H$_2$), 27.4 (*C*H$_2$); HRMS (+ACPI) *m/z* calculated for $C_{18}H_{20}N$ [M+H^{+}] 250.1596; Found 250.1587.

Data consistent with literature.[10]

Alternatively, following photoactivation of **4a** (201.0 mg, 0.96 mmol), the reaction mixture was cooled to 0 ^{o}C. The allylindium reagent derived from reaction of allyl iodide (0.25 mL, 2.75 mmol) and In powder (0.22 g, 1.92 mmol) in DMF (2.0 mL) was added directly to the reaction mixture dropwise over 15 min. Work up and purification as above gave **6aj** as a pale yellow oil (162.5 mg, 68%);

Alternatively, following photoactivation of **4a** (196.0 mg, 0.94 mmol), crude **5a** was suspended in anhydrous THF (5 mL). After cooling to 0 °C, The allylzinc reagent derived from from allylmagnesium bromide (1.0 M in Et_2O) (1.90 mL, 1.90 mmol) and zinc chloride (1.9 M in THF) (1.30 mL, 2.47 mmol) was added dropwise over 15 min. was added directly to the reaction mixture dropwise over 15 min. Work up and purification as above gave **6aj** as a pale yellow oil (205.5 mg, 88%);

Alternatively, following photoactivation of **4a** (198.0 mg, 0.95 mmol), crude **5a** was dissolved in anhydrous MeCN (5 mL). After cooling to 0 °C, the allylzinc reagent derived from allylmagnesium bromide (1.0 M in Et_2O) (1.90 mL, 1.90 mmol) and zinc chloride (1.9 M in THF) (1.30 mL, 2.47 mmol) was added dropwise over 15 min. Work up and purification by Mass Directed Autoprep gave **6aj** as a colourless oil (86.9 mg, 37%). This reaction also afforded **7a** as a side-product.

1-(2-Methylenepent-4-en-1-yl)-2-phenyl-1,2,3,4-tetrahydroisoquinoline (7a)

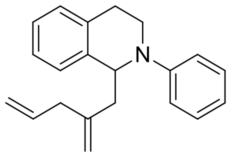

Following addition of the allylzinc reagent to **5a**, side-product ***7a*** was obtained as a colourless oil (11.8 mg, 4%); ^{1}H NMR (400 MHz, $CDCl_3$) δ 7.22 (2H, apt. t, *J* = 8.1 Hz, Ar*H*), 7.17 - 7.07 (4H, m, Ar*H*), 6.92 (2H, d, *J* = 8.1 Hz, Ar*H*), 6.73 (1H, apt. t, *J* = 7.3, Ar*H*), 5.82 - 5.78 (1H, m, C*H*=CH_2), 5.05 - 4.99 (2H, m, CH=CH_2), 4.86 (1H, m, C=CH_2), 4.84 (1H, t, *J* = 7.1 Hz C*H*N), 4.80 (1H, m, C=CH_2), 3.65 - 3.61 (2H, m, CH_2CH_2N), 3.06 - 2.98 (1H, m, C$H_2$$CH_2$N), 2.87 - 2.81 (1H, m, C$H_2$$CH_2$N), 2.77 - 2.69 (3H, m, CH_2CHN, CH_2=CHCH_2), 2.43 - 2.38 (1H, m, CH_2CHN); ^{13}C NMR (101 MHz, $CDCl_3$) δ 149.5* (*C*), 145.2 (*C*), 138.4 (*C*), 136.3 (*C*H), 134.8 (*C*), 129.2 (*C*H), 128.5 (*C*H), 127.5 (*C*H), 126.5 (*C*H), 125.6 (*C*H), 117.5 (C*H*), 116.4 (*C*H_2), 114.4 (*C*H), 113.7 (*C*H_2), 58.7 (*C*H), 42.3 (*C*H_2), 41.8 (*C*H_2) 41.0 (*C*H_2), 26.9 (*C*H_2); HRMS (+ACPI) *m/z* calculated for $C_{21}H_{24}N$ [M+H$^+$] 290.1909; Found 290.1904. *The quarternary aniline signal could not be observed by ^{13}C NMR. Coupling was observed to this carbon by HMBC NMR.

LC–MS – Addition of allyl organozinc reagent to **5a** in MeCN (Table 1, entry 13)

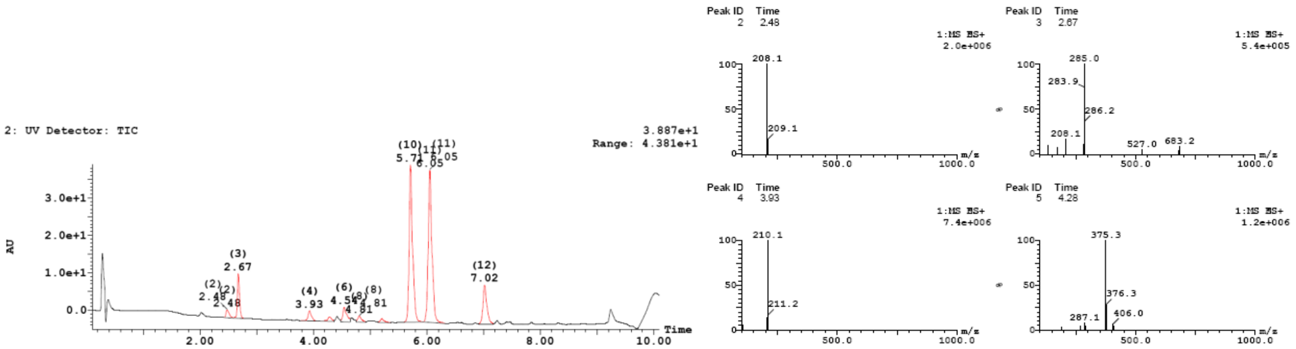

Peaks 10 ($M+H^+$ = 292, **8a**), 11 ($M+H^+$ = 250, **6aj**) and 12 ($M+H^+$ = 290, **7a**) in a 4 : 4 : 1 ratio, respectively.

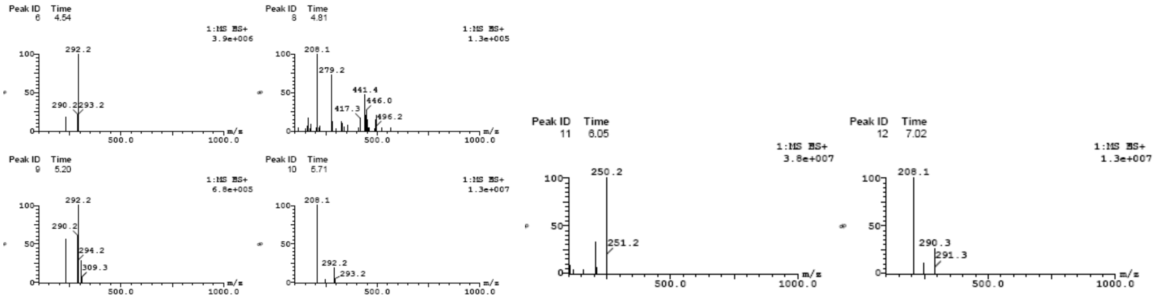

LC–MS – Addition of allyl organozinc reagent (premixed 1 : 1 with MeCN) to **5a** in MeCN

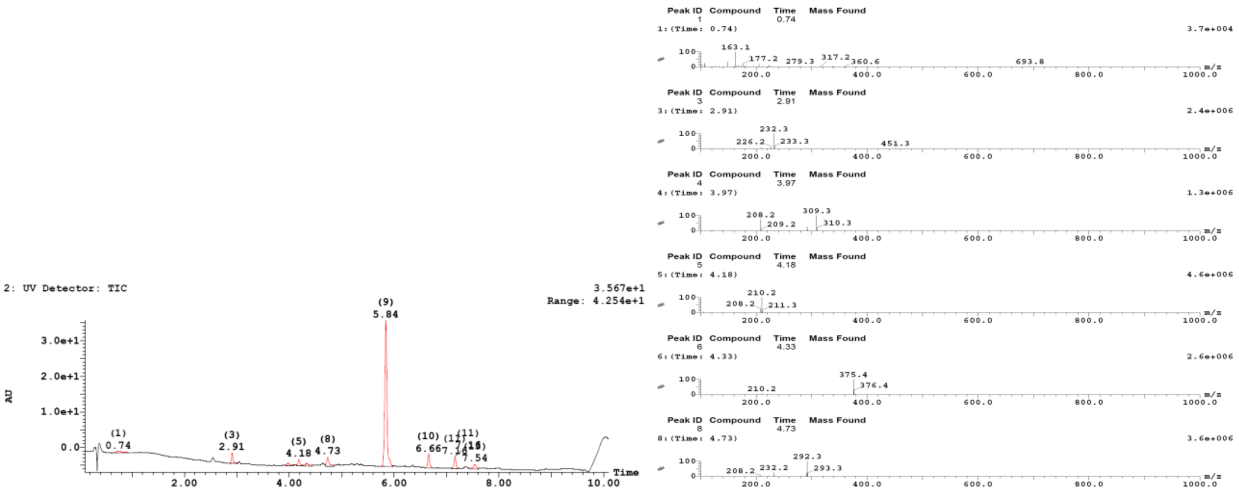

Peak 9 ($M+H^+$ = 292, **8a**). Only a trace of **7a**.

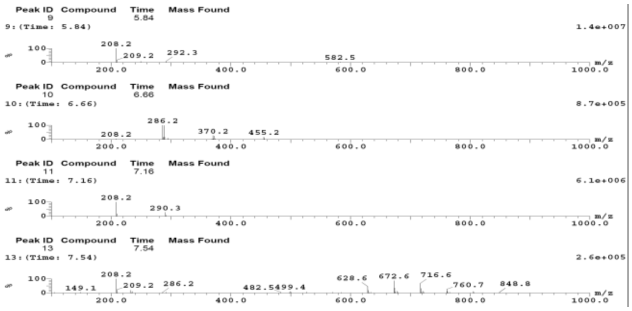

LC–MS – Addition of allyl organozinc reagent to **5a** in THF

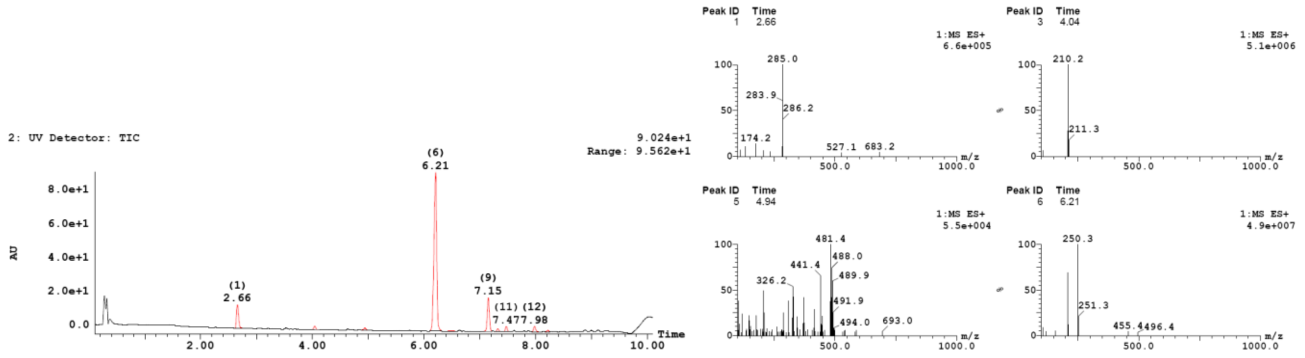

Peaks 6 (M+H$^+$ = 250, **6aj**) and 9 (M+H$^+$ = 290, **7a**) in a 7 : 1 ratio.

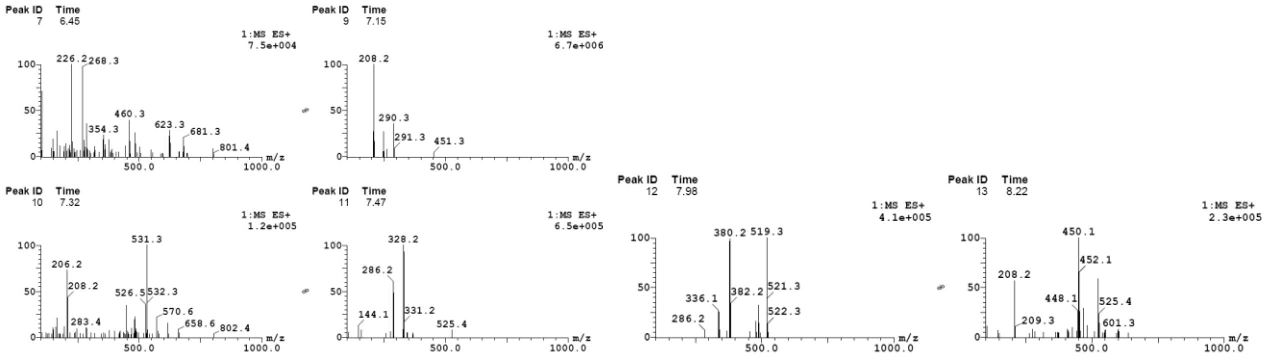

LC–MS – Addition of allyl organozinc reagent (which had been refluxed for 5 h) to **5a** in THF

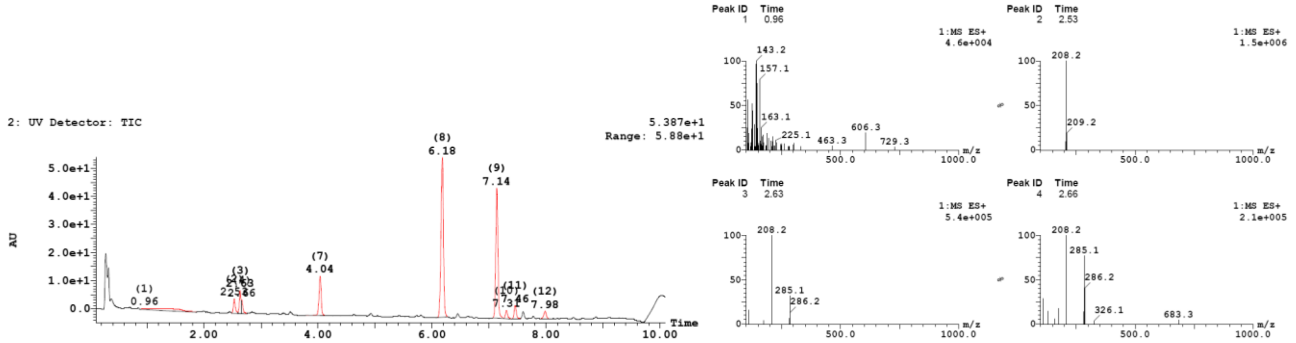

Peaks 7 (M+H$^+$ = 210, **4a**), 8 (M+H$^+$ = 250, **6aj**) and 9 (M+H$^+$ = 290, **7a**) in a 1 : 5 : 3 ratio, respectively. Ratio **6aj** : **7a** = 3 : 2.

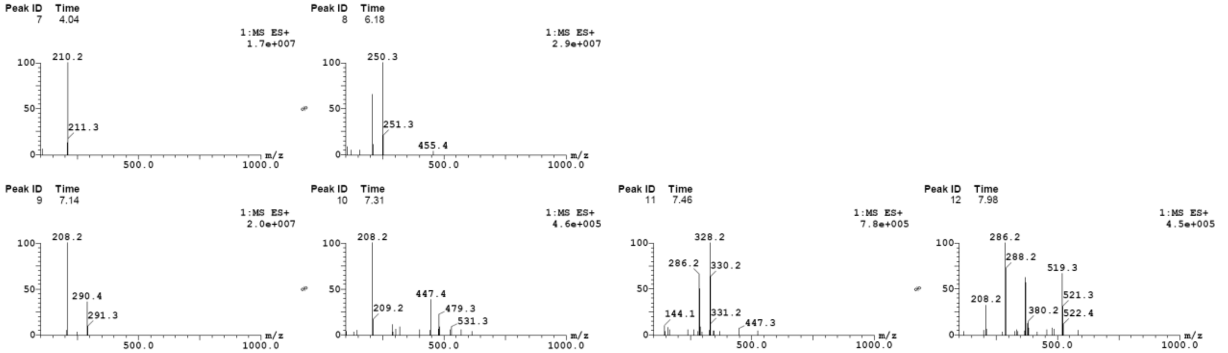

(*E*)-5-(2-phenyl-1,2,3,4-tetrahydroisoquinolin-1-yl)pent-3-en-2-one (8a)

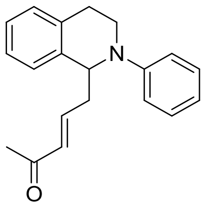

Following photoactivation of **4a** (197.0 mg, 0.94 mmol), crude **5a** was dissolved in anhydrous MeCN (5 mL) and a dropping funnel fitted to the flask was charged with anhydrous MeCN (0.14 mL, 2.62 mmol). The allylzinc reagent derived from from allylmagnesium bromide (1.0 M in Et_2O) (1.90 mL, 1.90 mmol) and zinc chloride (1.9 M in THF) (1.30 mL, 2.47 mmol) was added to the dropping funnel dropwise over 15 min at rt. The resulting cloudy black solution was added to **5a** dropwise over 15 min at 0 °C. Work up and purification by Mass Directed Autoprep gave ***8a*** as a yellow oil (91.8 mg, 34%); IR ν_{max} (neat) 3023 - 2837 (C-H), 1695, 1670 (C=O), 1625 (C=C), 1597 (Ar), 1503 (Ar), 1451, 1428, 1392, 1360, 1326, 1288, 1254, 1222 cm^{-1}; ^1H NMR (400 MHz, $CDCl_3$) δ 7.25 (2H, t, *J* = 8.1 Hz, Ar*H*), 7.20 - 7.14 (3H, m, Ar*H*), 7.08 (1H, m, Ar*H*), 6.90 (2H, d, *J* = 8.1 Hz, Ar*H*), 6.80 - 6.72 (2H, m, Ar*H*, C*H*CHC(O)CH_3), 6.05 (1H, d, *J* = 15.9 Hz, CHC*H*C(O)CH_3), 4.88 (1H, t, *J* = 6.9 Hz, C*H*N), 3.66 - 3.55 (2H, m, CH_2CH_2NAr), 3.08 - 3.00 (1H, m, CH_2CH_2NAr), 2.92 - 2.82 (2H, m, CH_2CH_2NAr, CH_2CHN), 2.70 - 2.62 (1H, m, CH_2CHN), 2.15 (3H, m, CHCHC(O)CH_3), ^{13}C NMR (101 MHz, $CDCl_3$) δ 198.5 (*C*=O), 149.5 (*C*), 145.0 (*C*H), 137.4 (*C*), 134.9 (*C*), 133.1 (*C*H), 129.4 (*C*H), 128.8 (*C*H), 127.0 (*C*H), 126.1 (*C*H), 118.2 (*C*H), 114.8 (*C*H), 58.9 (*C*H), 42.0 (CH_2), 39.7 (CH_2), 27.4 (CH_2), 26.7 (CH_3); HRMS (+ESI) *m/z* calculated for $C_{20}H_{22}NO$ [M+H^+] 292.1696; Found 292.1696.

1-Allyl-2-(naphthalen-2-yl)-1,2,3,4-tetrahydroisoquinoline (6ba)[10]

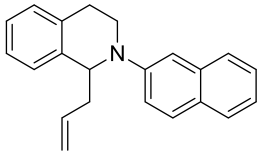

Following photoactivation of **4b** (236.0 mg, 0.91 mmol) according to the general procedure, crude **5b** was added to a separatory funnel containing water (20 mL) and DCM (20 mL). The layers were separated and the aqueous layer was extracted rigorously with DCM until complete extraction of 5b as deemed by HPLC. The combined organic layers were concentrated to dryness and the resultant crude product dissolved in minimal MeCN. The resultant crude product was washed into a separatory funnel with toluene (100 mL) and water (100 mL). The layers were separated and the organic layer was extracted rigorously with water until completion as deemed by HPLC. After extraction of the aqueous layers into DCM as before, the combined organic layers were dried ($MgSO_4$), filtered and concentrated *in vacuo* to yield crude **5b**, which was dissolved in anhydrous MeCN (5 mL). After cooling to 0 °C, the allylzinc reagent derived from allylmagnesium bromide (1.0 M in Et_2O) (1.90 mL, 1.90 mmol) and zinc chloride (1.9 M in THF) (1.30 mL, 2.47 mmol) was added dropwise over 15 min. Work up and purification by Mass Directed Autoprep gave **6ba** as a light brown oil (29.2 mg, 11%); ^1H NMR (400 MHz, $CDCl_3$) δ 7.76 (1H, d, *J* = 9.0 Hz, Ar*H*), 7.72 (1H, d, *J* = 8.1 Hz, Ar*H*), 7.67 (1H, d, *J* = 8.3 Hz, Ar*H*), 7.38 (1H, td, *J* = 8.1, 1.2 Hz, Ar*H*), 7.33 (1H, dd, *J* = 9.1, 2.6 Hz, Ar*H*), 7.26 - 7.18 (5H, m, Ar*H*), 7.11 (1H, d, *J* = 2.5 Hz, Ar*H*), 5.97 - 5.87 (1H, m, C*H*=CH_2), 5.14 - 5.07 (2H, m, CH=CH_2), 4.94 (1H, t, *J* = 6.8 Hz, C*H*N), 3.83 - 3.72 (2H, m, CH_2CH_2N), 3.15 - 3.08 (1H, m, CH_2CH_2N), 2.97 - 2.90 (1H, m, CH_2CH_2N), 2.84 - 2.77 (1H, m, CH_2CHN), 2.62 - 2.55 (1H, m, CH_2CHN); ^{13}C NMR (101 MHz, $CDCl_3$) δ 147.4 (*C*), 138.1 (*C*), 135.7 (*C*H), 135.0 (*C*), 134.9 (*C*), 128.9 (*C*H), 128.7 (*C*H), 127.4 (2 x *C*H, 1 x *C*), 126.6 (*C*H), 126.3 (*C*H), 126.2 (*C*H), 125.8 (*C*H), 122.4 (*C*H), 117.7 (*C*H), 117.1 (CH_2),

108.3 (*C*H), 59.4 (*C*H), 42.1 (*C*H_2), 40.9 (*C*H_2), 27.5 (*C*H_2); HRMS (+ACPI) *m/z* calculated for $C_{22}H_{22}N$ [M+H$^+$] 300.1752; Found 300.1746.

Data not consistent with literature.[10] We are confident in our assignment.

This reaction also afforded **7b** and **9** as side-products.

1-(2-Methylenepent-4-en-1-yl)-2-(napthalen-2-yl)-1,2,3,4-tetrahydroisoquinoline (7b)

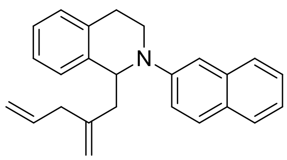

Following addition of the allylzinc reagent to **5b**, side-product ***7b*** was obtained as a light brown oil (3.1 mg, 1%); ^{1}H NMR (400 MHz, $CDCl_3$) δ 7.71 (1H, d, *J* = 9.1 Hz, Ar*H*), 7.68 (1H, d, *J* = 8.1 Hz, Ar*H*), 7.62 (1H, d, *J* = 8.4 Hz, Ar*H*), 7.36 - 7.32 (1H, m, Ar*H*), 7.31 (1H, dd, *J* = 9.1, 2.6 Hz, Ar*H*), 7.22 - 7.18 (1H, m, Ar*H*), 7.17 - 7.11 (4H, m, Ar*H*), 7.09 (1H, d, *J* = 2.5 Hz, Ar*H*), 5.83 - 5.73 (1H, m, C*H*=CH_2), 5.05 - 4.98 (3H, m, CH=CH_2, C*H*N), 4.87 (1H, m, C=CH_2), 4.83 (1H, m, C=CH_2), 3.83 - 3.69 (2H, m, CH_2CH_2N), 3.11 - 3.03 (1H, m, C$H_2$$CH_2$N), 2.89 - 2.83 (1H, m, C$H_2$$CH_2$N), 2.80 - 2.73 (3H, m, CH_2CHN, CH_2CH=CH_2), 2.50 - 2.44 (1H, m, CH_2CHN);

Assigned tentatively by ^{1}H NMR data.

1-(9,13b,14,15-tetrahydro-8*H*-benzo[*f*]isoquino[2,1-*a*]quinolin-15-yl)propan-2-one (9)

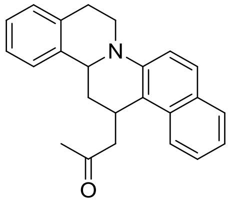

Following addition of the allylzinc reagent to **5b**, side-product ***9*** was obtained as a light brown oil (6.0 mg, 2%); ^{1}H NMR (400 MHz, $CDCl_3$) δ 7.76 (1H, d, *J* = 8.6 Hz, Ar*H*), 7.71 (1H, d, *J* = 8.1 Hz, Ar*H*), 7.65 (1H, d, *J* = 9.1 Hz, Ar*H*), 7.45 - 7.41 (1H, m, Ar*H*), 7.25 - 7.18 (6H, m, Ar*H*), 4.57 (1H, dd, *J* = 12.2, 3.1 Hz, C*H*N), 4.22 - 4.18 (1H, m, C*H*Ar), 3.96 - 3.91 (1H, m, CH_2CH_2N), 3.46 - 3.40 (1H, m, CH_2CH_2N), 3.11 - 2.93 (4H, m, C$H_2$$CH_2N, CH_2$C(O)$CH_3$), 2.47 - 2.42 (1H, m, C$H_2$CHN), 2.27 (3H, s, C$H_3$), 2.12 - 2.04 (1H, m, *J* = 4.7 Hz, CH_2CHN); ^{13}C NMR (101 MHz, $CDCl_3$) δ 207.7 (*C*=O), 143.2 (*C*), 138.4 (*C*), 135.4 (*C*), 131.9 (*C*), 128.7 (*C*H), 128.4 (*C*H), 128.1 (*C*H), 127.7 (*C*), 126.7 (*C*H), 126.4 (*C*H), 126.3 (*C*H), 125.5 (*C*H), 122.0 (*C*H), 121.0 (*C*H), 117.1 (*C*), 116.7 (*C*H), 51.6 (*C*H), 49.6 (*C*H_2), 45.5 (*C*H_2), 32.1 (*C*H_2), 30.8 (*C*H_3), 29.9 (*C*H_2), 27.7 (*C*H); HRMS (+ACPI) *m/z* calculated for $C_{24}H_{24}NO$ [M+H$^+$] 342.1858; Found 342.1853.

2-(7,11b,12,13-tetrahydro-6*H*-isoquinolino[2,1-*a*]quinolin-13-yl)acetaldehyde (8b)

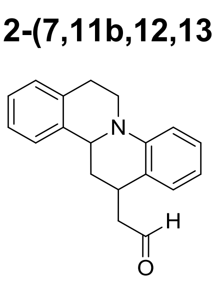

Following photoactivation of **4a** (197.0 mg, 0.94 mmol), a flask was charged with crude **5a** and (2*S*,5*S*)-(−)-2-*tert*-Butyl-3-methyl-5-benzyl-4-imidazolidinone (46.0 mg, 0.19 mmol) in MeCN (6 mL). The mixture was cooled to 0 °C

before Crotonaldehyde (0.09 mL, 1.09 mmol) and TFA (0.15 mL, 2.09 mmol) was added. The reaction mixture was stirred for 12 h (the ratio of **5a** : **8b** was 1 : 2 by HPLC) at rt before pouring into a separatory funnel with DCM (30 mL), sat. $NaHCO_3$ (10 mL) and water (20 mL). The layers were separated and aqueous layer extracted with DCM (2 x 30 mL). The combined organic layers were dried ($MgSO_4$), filtered and concentrated *in vacuo* to yield a brown oil. Purification by Mass Directed Autoprep gave ***8b*** as a red oil (67.9 mg, 26%), IR ν_{max} (neat) 3060 - 2832 (C-H), 1720 (C=O) 1600 (Ar), 1572 (Ar), 1489 (Ar), 1455, 1381, 1355, 1322, 1308, 1265, 1230 cm^{-1}; ^1H NMR (400 MHz, $CDCl_3$) δ 9.86 (1H, s, $CHCH_2CHO$), 7.24 - 7.13 (5H, m, Ar*H*), 7.05 (1H, d, *J* = 7.3 Hz, Ar*H*), 6.85 (1H, d, *J* = 8.1 Hz, Ar*H*), 6.74 (1H, t, J = 7.3 Hz, Ar*H*), 4.46 (1H, dd, *J* = 10.8, 3.4 Hz, C*H*N), 3.92 - 3.87 (1H, m, CH_2CH_2N), 3.69 - 3.62 (1H, m, $CHCH_2CHO$), 3.20 - 3.14 (1H, m, CH_2CH_2N), 3.13 - 3.05 (1H, m, CH_2CH_2N), 2.97 (1H, dd, *J* = 17.4, 4.7 Hz, $CHCH_2CHO$), 2.89 - 2.83 (1H, m, CH_2CH_2N), 2.74 - 2.64 (2H, m, CH_2CHN, $CHCH_2CHO$), 1.82 (1H, apt. q, *J* = 10.8 Hz, CH_2CHN); ^{13}C NMR (101 MHz, $CDCl_3$) δ 200.1 (*C*HO), 145.1 (*C*), 136.4 (*C*), 132.9 (*C*), 126.7 (*C*H), 125.7 (*C*H), 124.8 (*C*), 124.7 (*C*H), 124.4 (2 x *C*H), 123.9 (*C*H), 115.9 (*C*H), 110.9 (*C*H), 54.5 (*C*H), 48.0 (CH_2), 42.0 (CH_2), 35.8 (CH_2), 29.5 (*C*H), 27.2 (CH_2); HRMS (+ESI) *m/z* calculated for $C_{19}H_{20}NO$ [M+H$^+$] 292.1539; Found 292.1537.

2-(Naphthalen-2-yl)-1-phenyl-1,2,3,4-tetrahydroisoquinoline (6bb)

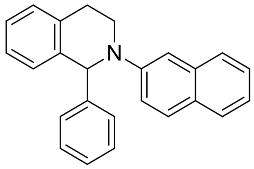

Following photoactivation of **4b** (251.0 mg, 0.97 mmol) according to the general procedure, crude **5b** was dissolved in anhydrous MeCN (10 mL). After cooling to 0 °C, phenylmagnesium bromide (1 M in THF) (2.10 mL, 2.10 mmol) was added dropwise over 15 min. Work up gave the crude product as a brown oil. Purification by Mass Directed Autoprep to yield ***6bb*** as a pale brown oil (153.1 mg, 47%); IR ν_{max} (neat) 3025 - 2917 (C-H), 1626 (Ar), 1597 (Ar), 1509 (Ar), 1492, 1470, 1446, 1386, 1356, 1319, 1300, 1259, 1217 cm^{-1}; ^1H NMR (400 MHz, $CDCl_3$) δ 7.69 - 7.66 (2H, m, Ar*H*), 7.60 (1H, d, *J* = 8.2 Hz, Ar*H*), 7.35 - 7.15 (12H, m, Ar*H*), 7.05 (1H, d, *J* = 2.4 Hz, Ar*H*), 5.99 (1H, s, ArC*H*N), 3.80 - 3.74 (1H, m, CH_2CH_2N), 3.67 - 3.60 (1H, m, CH_2CH_2N), 3.03 - 2.88 (2H, m, CH_2CH_2N); ^{13}C NMR (101 MHz, $CDCl_3$) δ 147.4 (*C*), 142.9 (*C*), 137.6 (*C*), 135.7 (*C*), 134.9 (*C*), 128.8 (*C*H), 128.2 (2 x *C*H), 128.0 (*C*H), 127.5 (2 x *C*H), 127.4 (*C*H, *C*), 127.1 (*C*H), 126.9 (*C*H), 126.3 (*C*H), 126.2 (*C*H), 122.4 (*C*H), 117.4 (*C*H), 108.2 (*C*H), 62.8 (*C*H), 43.8 (CH_2), 28.0 (CH_2); HRMS (+ACPI) *m/z* calculated for $C_{25}H_{22}N$ [M+H$^+$] 336.1752; Found 336.1745.

6,7-Dimethoxy-1,2-diphenyl-1,2,3,4-tetrahydroisoquinoline (11b)

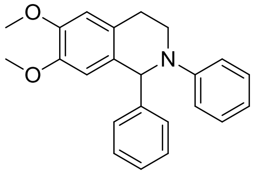

Following photoactivation of **11a** (266.0 mg, 0.99 mmol) according to the general procedure, the crude iminium salt was dissolved in anhydrous MeCN (5 mL). After cooling to 0 °C, phenylmagnesium bromide (1 M in THF) (2.0 mL, 2.0 mmol) was added dropwise over 15 min. Work up gave the crude product as a brown oil. Purification by flash column chromatography (a gradient of 70 - 100% toluene/heptane (1% Et_3N)) gave a yellow oil, which crystallised overnight to yield ***11b*** as a microcrystalline yellow solid (323.3 mg, 95%); m.p. 103 - 105 °C; IR ν_{max} 3058 - 2832 (C-H), 1596 (Ar), 1524, 1502 (Ar), 1470, 1457, 1442, 1416, 1379, 1339, 1278, 1244, 1218 cm^{-1}; ^1H NMR (400 MHz, $CDCl_3$) δ 7.27 - 7.17 (7H, m, Ar*H*), 6.90 (2H, d, *J* = 8.4 Hz, Ar*H*), 6.76 (1H, t, *J* = 7.3 Hz, Ar*H*), 6.73 (1H, s, Ar*H*), 6.66 (1H, s, Ar*H*), 5.76 (1H, s, ArC*H*N), 3.86 (3H, s, OCH_3), 3.84 (3H, s, OCH_3), 3.62 - 3.48 (2H, m, CH_2CH_2N), 2.94 - 2.87 (1H, m, CH_2CH_2N), 2.80 - 2.75 (1H, m, CH_2CH_2N); ^{13}C NMR (101 MHz, $CDCl_3$) δ 149.8 (*C*), 148.0 (*C*), 147.3 (*C*), 143.4 (*C*), 129.3 (*C*), 129.2 (*C*H), 128.2 (*C*H), 127.8 (*C*), 127.6 (*C*H), 126.9 (*C*H), 117.9

(*C*H), 114.7 (*C*H), 111.3 (2 x *C*H), 62.4 (*C*H), 56.1 (CH_3), 55.9 (CH_3), 43.2 (CH_2), 27.1 (CH_2); HRMS (+APCI) *m/z* calculated for $C_{23}H_{24}NO_2$ [M+H$^+$] 346.1807; Found 346.1800.

Alternatively, following photoactivation of **11a** (257.0 mg, 0.97 mmol) with $BrCH_2CN$ (0.14 mL, 2.00 mmol) and $Ru(bpy)_3(PF_6)_2$ (8.4 mg, 9.8 μmol) in anhydrous MeCN (7.5 mL), the reaction mixture was cooled to 0 °C. To the reaction mixture was added phenylmagnesium bromide (1.0 M in THF) (2.00 mL, 2.00 mmol) dropwise over 15 min. Work up and purification as above gave ***11b*** as a microcystalline yellow solid (170.4 mg, 72%);

2-(4-Methoxyphenyl)-1-phenyl-1,2,3,4-tetrahydroisoquinoline (12b)[7]

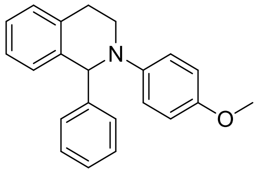

Following photoactivation of **12a** (223.0 mg, 0.93 mmol) according to the general procedure, the crude iminium salt was dissolved in anhydrous MeCN (5 mL). After cooling to 0 °C, phenylmagnesium bromide (1 M in THF) (1.90 mL, 1.90 mmol) was added dropwise over 15 min. Work up gave the crude product as a brown oil. Purification by flash column chromatography (a gradient of 50 - 70% toluene/heptane (1% Et_3N)) gave a yellow oil, which crystallised overnight. The yellow solid was recrystallised from MeCN to yield **12b** as a microcrystalline yellow solid (152.5 mg, 52%); m.p. 129 - 131 °C; IR ν_{max} (neat) 3023 - 2823 (C-H), 1508 (Ar), 1493, 1464, 1451, 1440, 1370, 1293, 1264, 1242, 1216, 1204 cm^{-1}; ^{1}H NMR (400 MHz, $CDCl_3$) δ 7.23 - 7.15 (9H, m, Ar*H*), 6.83 - 6.77 (4H, m, Ar*H*), 5.66 (1H, s, ArC*H*N), 3.74 (3H, s, OCH_3), 3.60 - 3.54 (1H, m, CH_2CH_2N), 3.44 - 3.38 (1H, m, CH_2CH_2N), 3.01 - 2.88 (2H, m, CH_2CH_2N); ^{13}C NMR (101 MHz, $CDCl_3$) δ 152.9 (*C*), 144.5 (*C*), 143.3 (*C*), 137.7 (*C*), 135.5 (*C*), 128.4 (*C*H), 128.1 (2 x *C*H), 128.0 (*C*H), 126.8 (*C*H) 126.7 (*C*H), 126.0 (*C*H), 117.8 (*C*H), 114.5 (*C*H), 64.4 (*C*H), 55.7 (CH_3), 44.6 (CH_2), 28.2 (CH_2); HRMS (+ACPI) *m/z* calculated for $C_{22}H_{22}NO$ [M+H$^+$] 316.1701; Found 316.1697.

Data consistent with literature.[7]

Alternatively, following photoactivation of **12a** (238.9 mg, 1.00 mmol) with $BrCH_2CN$ (0.14 mL, 2.00 mmol) and $Ru(bpy)_3(PF_6)_2$ (8.6 mg, 10.0 μmol) in anhydrous MeCN (7.5 mL), the reaction mixture was cooled to 0 °C. To the reaction mixture was added phenylmagnesium bromide (1.0 M in THF) (2.00 mL, 2.00 mmol) dropwise over 15 min. Work up and purification as above gave **12b** as a microcrystalline yellow solid (231.3 mg, 73%);

2-(4-Bromophenyl)-1-phenyl-1,2,3,4-tetrahydroisoquinoline (13b)

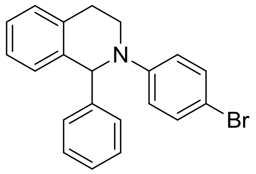

Following photoactivation of **13a** (278.0 mg, 0.95 mmol) according to the general procedure, the crude iminium salt was dissolved in dry MeCN (10 mL). After cooling to 0 °C, phenylmagnesium bromide (1 M in THF) (2.0 mL, 2.0 mmol) was added dropwise over 15 min. Work up gave the crude product as a yellow brown oil. Purification by Mass Directed Autoprep gave ***13b*** as a colourless oil (181.6 mg, 53%); IR ν_{max} (neat) 3025 - 2841 (C-H), 1587 (Ar), 1490 (Ar), 1473, 1456, 1447, 1382, 1361, 1331, 1299, 1250, 1227 cm^{-1}; ^{1}H NMR (400 MHz, $CDCl_3$) δ 7.29 - 7.16 (11H, m, Ar*H*), 6.70 (2H, d, *J* = 9.1 Hz, Ar*H*), 5.75 (1H, s, C*H*N), 3.72 - 3.66 (1H, m, CH_2CH_2N), 3.48 - 3.42 (1H, m, CH_2CH_2N), 2.96 - 2.86 (2H, m, CH_2CH_2N); ^{13}C NMR (101 MHz, $CDCl_3$) δ 148.5 (*C*), 142.6 (*C*), 137.7 (*C*), 135.5 (*C*), 131.9 (*C*H), 128.4 (*C*H), 128.1 (*C*H), 127.8 (*C*H), 127.3 (*C*H), 127.2 (*C*H), 127.1 (*C*H), 126.4 (*C*H),

115.4 (*C*H), 109.4 (*C*), 62.8 (*C*H), 44.1 (*C*H_2), 28.0 (*C*H_2); HRMS (+APCI) *m/z* calculated for $C_{21}H_{19}BrN$ [$M+H^+$] 364.0701; Found 364.0696.

Alternatively, following photoactivation of **13a** (185.9 mg, 0.65 mmol) with $BrCH_2CN$ (0.09 mL, 1.29 mmol) and $Ru(bpy)_3(PF_6)_2$ (5.6 mg, 6.5 μmol) in anhydrous MeCN (6 mL) and anhydrous THF (3 mL) (every 1 h, the reaction mixture was heated gently until the precipitate dissolved for a reaction time of 5 h), the reaction mixture was co-oled to 0 °C. To the reaction mixture was added phenylmagnesium bromide (1.0 M in THF) (1.30 mL, 1.30 mmol) dropwise over 15 min. Work up and purification as above gave ***13b*** as a yellow oil (117.3 mg, 50%);

2-(4-Nitrophenyl)-1-phenyl-1,2,3,4-tetrahydroisoquinoline (14b)[11]

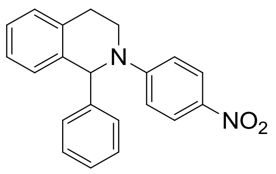

Following photoactivation of **14a** (245.0 mg, 0.97 mmol) according to the general procedure, the crude iminium salt was dissolved in anhydrous MeCN (10 mL). After cooling to 0 °C, phenylmagnesium bromide (1 M in THF) (2.0 mL, 2.0 mmol) was added dropwise over 15 min. Work up gave the crude product as a yellow brown oil. Purification by flash column chromatography (a gradient of 70 - 100 % toluene/heptane (1% Et_3N)) gave **14b** as a yellow microcrystalline solid (244.9 mg, 77%); m.p. 141 - 143 °C; IR ν_{max} (neat) 3075 - 2947 (C-H), 1598 (Ar), 1584 (N-O), 1506 (Ar), 1482, 1466, 1446, 1436, 1389, 1359, 1313 (N-O), 1299, 1257, 1231, 1219, 1207 cm^{-1}; ^1H NMR (400 MHz, $CDCl_3$) δ 8.14 (2H, d, *J* = 9.5 Hz, Ar*H*), 7.40 (1H, dd, *J* = 6.8, 2.0 Hz, Ar*H*), 7.32 - 7.20 (8H, m, Ar*H*), 6.79 (2H, d, *J* = 9.5 Hz, Ar*H*), 5.97 (1H, s, ArC*H*N), 3.89 (1H, dt, *J* = 11.2, 4.8 Hz CH_2CH_2N), 3.65 - 3.58 (1H, m, CH_2CH_2N), 2.97 (2H, dd, *J* = 7.8, 4.8 Hz, CH_2CH_2N); ^{13}C NMR (101 MHz, $CDCl_3$) δ 153.6 (*C*), 140.8 (*C*), 137.7 (*C*), 137.1 (*C*), 135.1 (*C*), 128.7 (*C*H), 128.0 (*C*H), 127.9 (*C*H), 127.6 (*C*H), 127.4 (*C*H), 126.8 (*C*H), 126.5 (*C*H), 126.2 (*C*H), 111.2 (*C*H), 62.3 (*C*H), 44.8 (*C*H_2), 27.9 (*C*H_2); HRMS (+APCI) *m/z* calculated for $C_{21}H_{19}N_2O_2$ [$M+H^+$] 331.1447; Found 331.1442.

Data consistent with literature.[11]

2-Methyl-1-phenyl-1,2,3,4-tetrahydroisoquinoline (15b)[12]

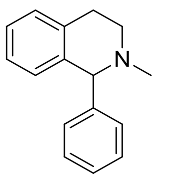

Following photoactivation of **15a** (147.2 mg, 1.00 mmol) according to the general procedure, the crude iminium salt was dissolved in dry MeCN (5 mL). After cooling to 0 °C, phenylmagnesium bromide (1 M in THF) (2.00 mL, 2.00 mmol) was added dropwise over 15 min. Work up gave the crude product as a brown oil. Purification by flash column chromatography (SNAP 50g, a gradient of 20 - 40% EtOAc (1% Et_3N)/heptane) gave **15b** as a yellow oil (129.8 mg, 58%); IR ν_{max} (neat) 3061 - 2701 (C-H), 1601 (Ar), 1492 (Ar), 1450, 1426, 1366, 1345, 1315, 1289, 1248, 1218 cm^{-1}; ^1H NMR (400 MHz, $CDCl_3$) δ 7.33 - 7.24 (5H, m, Ar*H*), 7.13 - 7.07 (2H, m, Ar*H*), 6.97 (1H, apt. t, *J* = 7.7 Hz, Ar*H*), 6.64 (1H, d, *J* = 7.8 Hz, Ar*H*), 4.23 (1H, s, ArC*H*N), 3.31 - 3.23 (1H, m, CH_2CH_2N), 3.15 - 3.10 (1H, m, CH_2CH_2N), 2.85 - 2.79 (1H, m, CH_2CH_2N), 2.67 - 2.60 (1H, m, CH_2CH_2N), 2.23 (3H, s, NCH_3); ^{13}C NMR (101 MHz, $CDCl_3$) δ 144.1 (*C*), 138.7 (*C*), 134.4 (*C*), 129.7 (*C*H), 128.6 (*C*H), 128.3 (2 x *C*H), 127.3 (*C*H), 125.9 (*C*H), 125.9 (*C*H), 125.7 (*C*H), 71.6 (*C*H), 52.4 (*C*H_2), 44.4 (*C*H_3), 29.6 (*C*H_2).

Data consistent with literature.[12]

6,7-Dimethoxy-2-methyl-1-phenyl-1,2,3,4-tetrahydroisoquinoline (16b)[13]

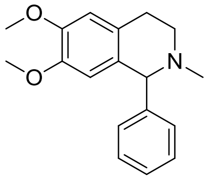

Following photoactivation of **16a** (201.2 mg, 0.97 mmol) according to the general procedure, the crude iminium salt was dissolved in dry MeCN (5 mL). After cooling to 0 °C, phenylmagnesium bromide (1 M in THF) (1.95 mL, 1.95 mmol) was added dropwise over 15 min. Work up gave the crude product as a brown oil. Purification by flash column chromatography (SNAP 50g, a gradient of 40 - 50% EtOAc (1% Et_3N)/heptane) gave **16b** as a pale yellow oil, which crystallised overnight to yield an off-white microcrystalline solid (222.4 mg, 81%); m.p. 82 - 84 °C, IR ν_{max} (neat) 2950 - 2789 (C-H), 1610 (Ar), 1515 (Ar), 1467, 1453, 1404, 1366, 1332, 1307, 1252, 1216 cm^{-1}; ^1H NMR (400 MHz, $CDCl_3$) δ 7.35 - 7.27 (5H, m, Ar*H*), 6.63 (1H, s, Ar*H*), 6.12 (1H, s, Ar*H*), 4.21 (1H, s, C*H*N), 3.87 (3H, s, OCH_3) 3.59 (3H, s, OCH_3), 3.24 - 3.10 (2H, m, CH_2CH_2N), 2.78 - 2.74 (1H, m, CH_2CH_2N), 2.67 - 2.60 (1H, m, CH_2CH_2N), 2.26 (3H, s, CH_3N), ^{13}C NMR (101 MHz, $CDCl_3$) δ 147.0 (2 x *C*), 143.9 (*C*), 130.4 (*C*), 129.5 (*C*H), 128.3 (*C*H), 127.3 (*C*H), 126.5 (*C*), 111.4 (*C*H), 110.7 (*C*H), 71.1 (*C*H), 55.8 (2 x CH_3), 52.2 (CH_2), 44.3 (CH_3), 29.0 (CH_2); HRMS (+ACPI) *m/z* calculated for $C_{18}H_{22}NO_2$ $[M+H^+]$ 284.1651; Found 284.1648.

Data consistent with literature.[13]

1-(4-Chlorophenethyl)-6,7-dimethoxy-2-methyl-1,2,3,4-tetrahydroisoquinoline/(±)-Methopholine (3)[14]

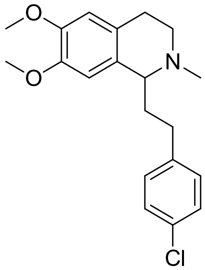

Following photoactivation of **16a** (199.0 mg, 0.96 mmol) according to the general procedure, the crude iminium salt was dissolved in dry MeCN (5 mL). After cooling to 0 °C, (4-chlorophenethyl)magnesium bromide (1.5 M in THF) (0.75 mL, 1.10 mmol) was added dropwise over 15 min. Work up gave the crude product as a pale red oil. Purification by flash column chromatography (a gradient of 20 - 100% EtOAc/Heptane) gave **3** as an off-white powder (185.3 mg, 56%); m.p. 111 - 113 °C, IR ν_{max} (neat) 2937 - 2786 (C-H), 1611 (Ar), 1518 (Ar), 1491, 1467, 1448, 1418, 1405, 1372, 1357, 1338, 1320, 1253, 1212 cm^{-1}; ^1H NMR (400 MHz, $CDCl_3$) δ 7.26 (2H, d, *J* = 8.1 Hz, Ar*H*), 7.13 (2H, d, *J* = 8.3 Hz, Ar*H*), 6.59 (1H, s, Ar*H*), 6.55 (1H, s, Ar*H*), 3.88 (3H, s, OCH_3), 3.85 (3H, s, OCH_3), 3.42 (1H, apt. t, *J* = 5.0 Hz, C*H*N), 3.17 - 3.12 (1H, m, CH_2CH_2N), 2.81 - 2.70 (4H, m, CH_2CH_2N, CH_2CH_2N, CH_2CH_2CHN), 2.57 - 2.52 (1H, m, CH_2CH_2CHN), 2.49 (3H, s, CH_3), 2.07 - 2.02 (2H, m, CH_2CH_2CHN); ^{13}C NMR (101 MHz, $CDCl_3$) δ 147.3 (2 x *C*), 141.4 (*C*), 131.2 (*C*), 129.8 (*C*H), 129.6 (*C*), 128.3 (*C*H), 126.8 (*C*), 111.3 (*C*H), 109.9 (*C*H), 62.6 (*C*H), 56.0 (OCH_3), 55.8 (OCH_3), 48.2 (CH_2), 42.7 (CH_3), 36.8 (CH_2), 30.8 (CH_2), 25.5 (CH_2); HRMS (+APCI) *m/z* calculated for $C_{20}H_{25}ClNO_2$ $[M+H^+]$ 346.1568; Found 346.1571.

^1H NMR and IR data are consistent with literature.[14]

6. ^{1}H AND ^{13}C NMR SPECTRA FOR ALL NOVEL COMPOUNDS

^{1}H NMR – **6aa**

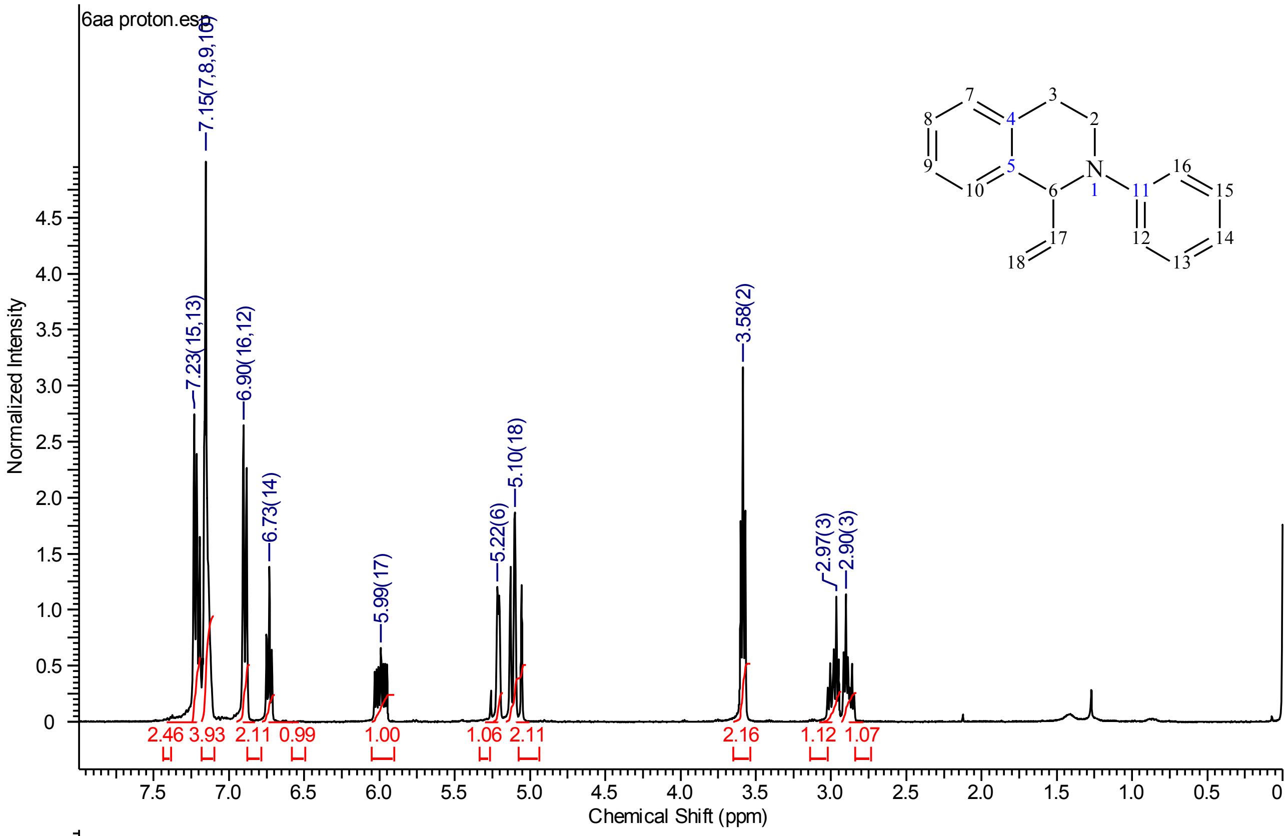

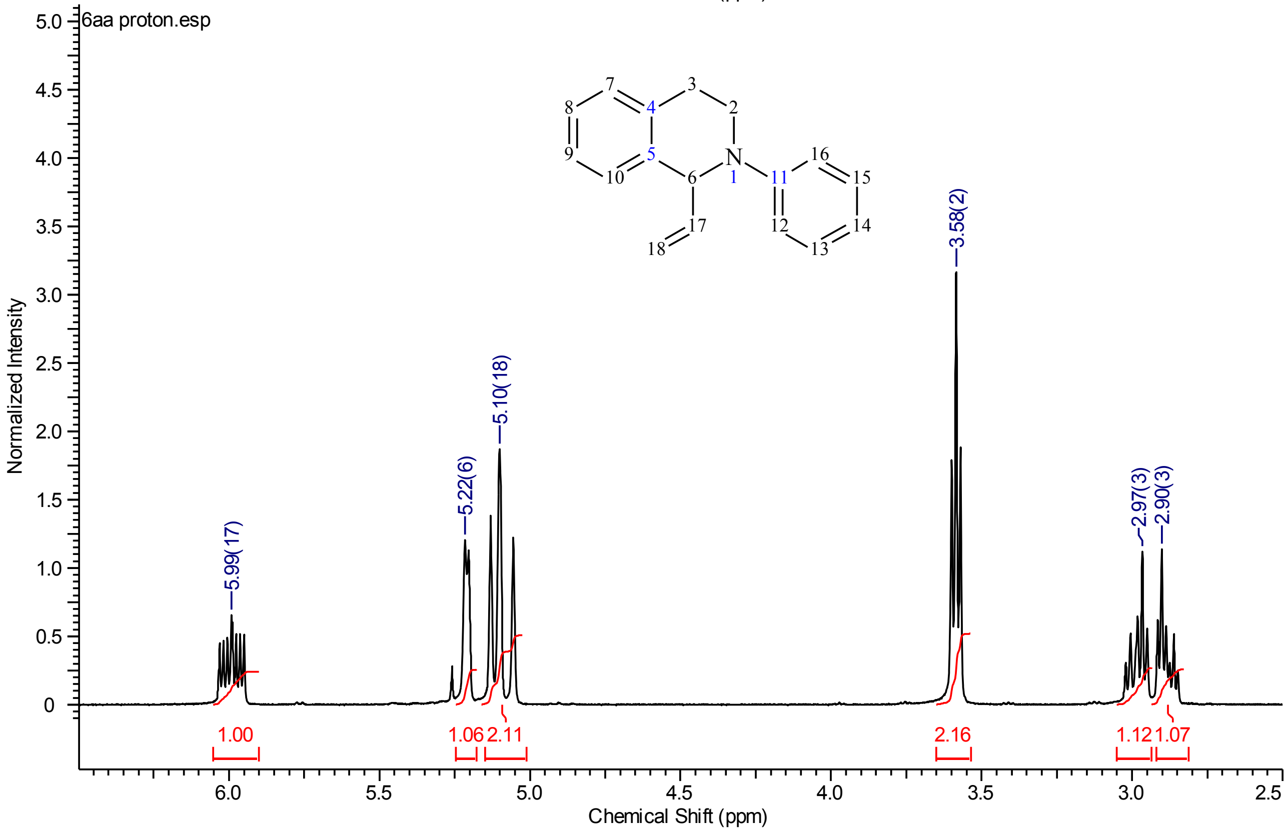

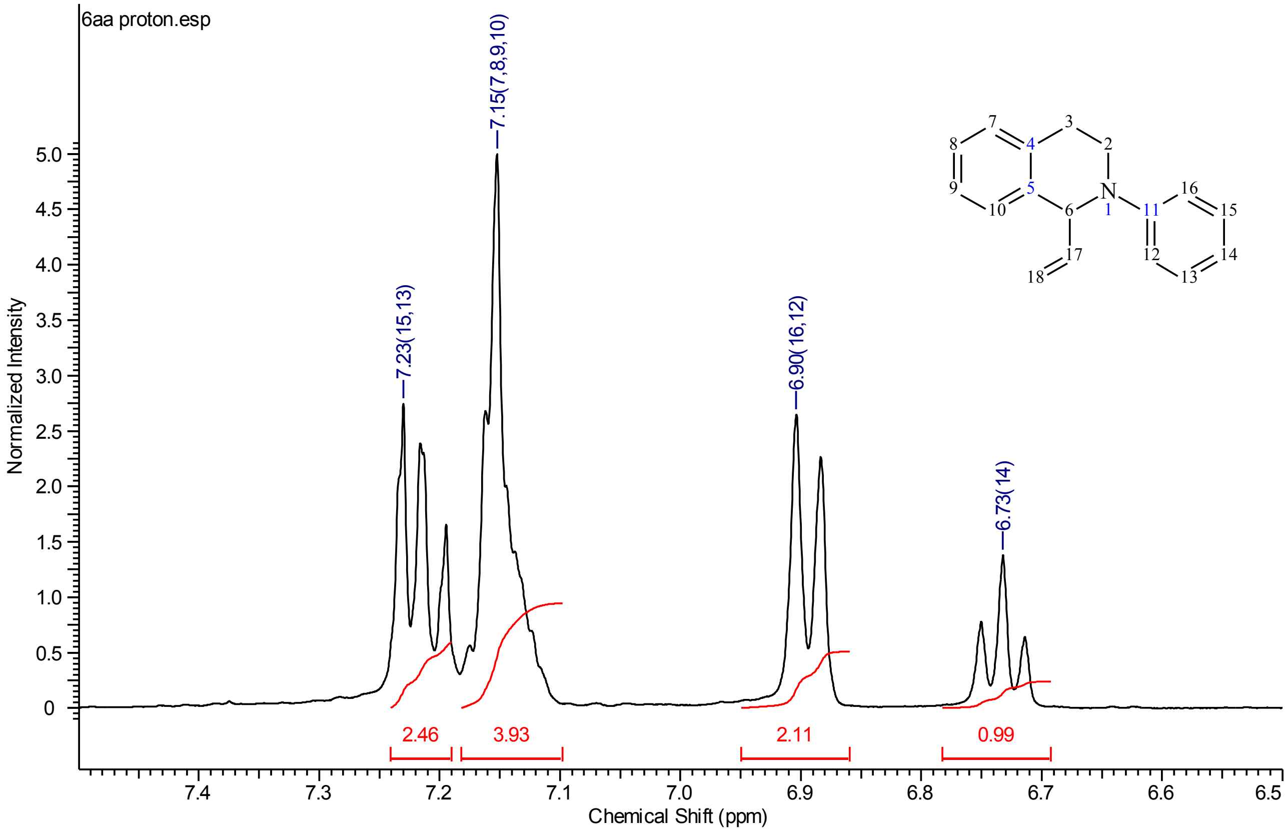
6aa proton.esp
7.15(7,8,9,10)
7.23(15,13)
6.90(16,12)
6.73(14)
2.46
3.93
2.11
0.99
Normalized Intensity
Chemical Shift (ppm)

^{13}C NMR – **6aa**

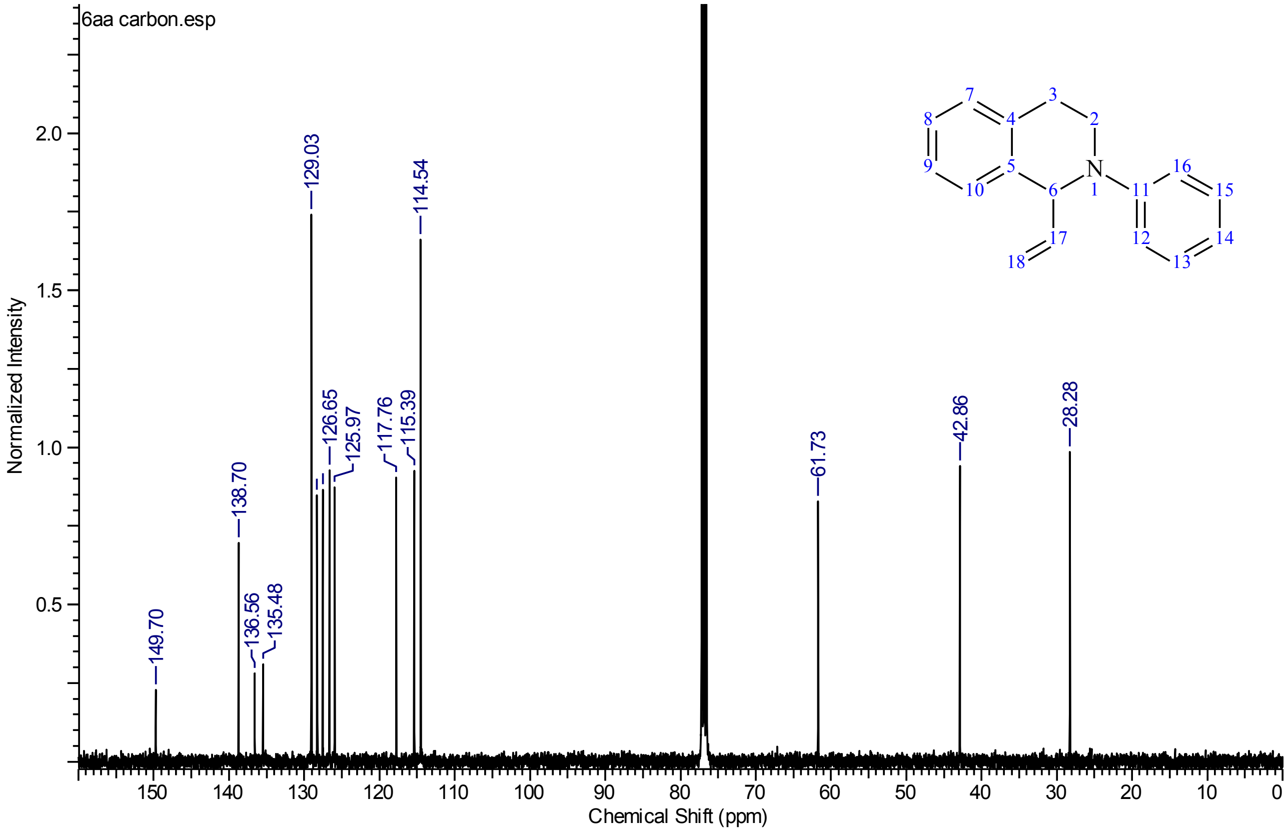
6aa carbon.esp
149.70
138.70
136.56
135.48
129.03
126.65
125.97
117.76
115.39
114.54
61.73
42.86
28.28
Normalized Intensity
Chemical Shift (ppm)

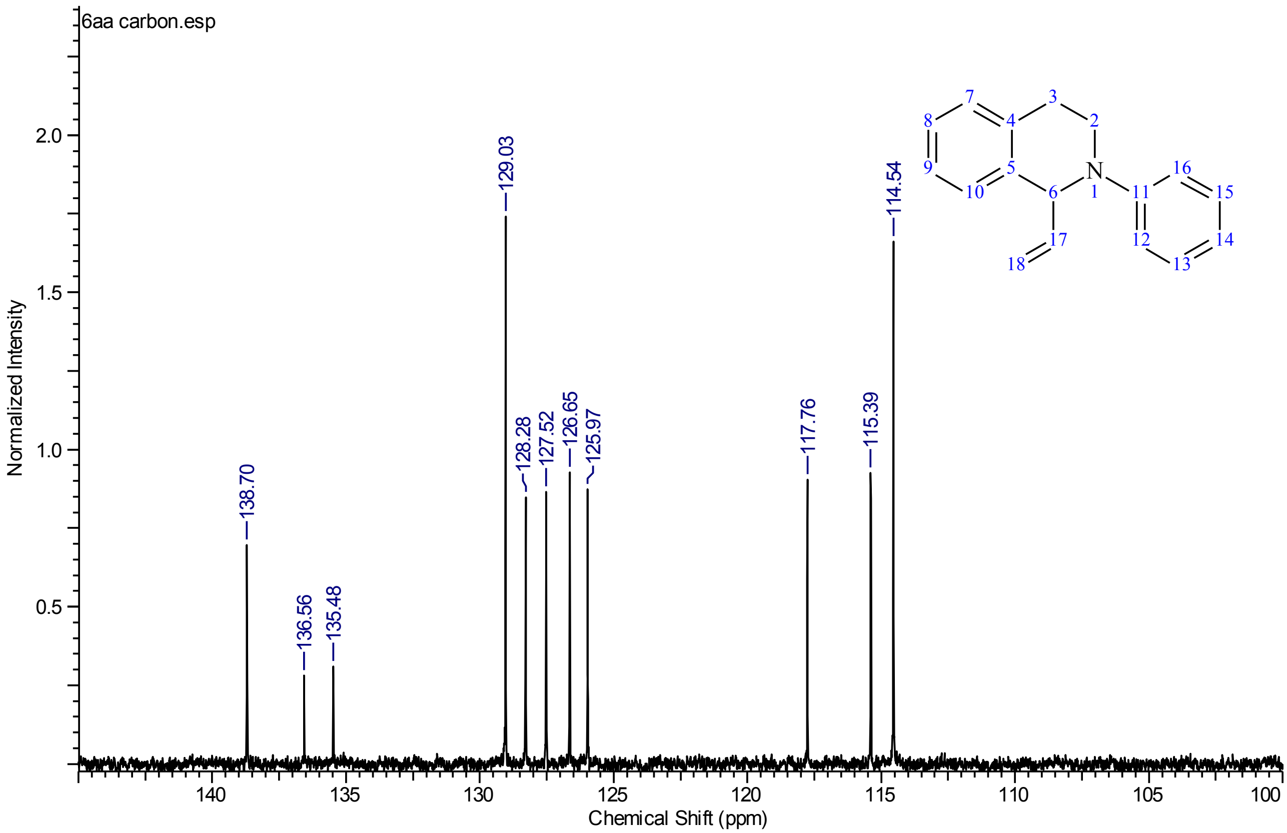
6aa carbon.esp
Normalized Intensity
Chemical Shift (ppm)
138.70
136.56
135.48
129.03
128.28
127.52
126.65
125.97
117.76
115.39
114.54

^{1}H NMR – **6ab**

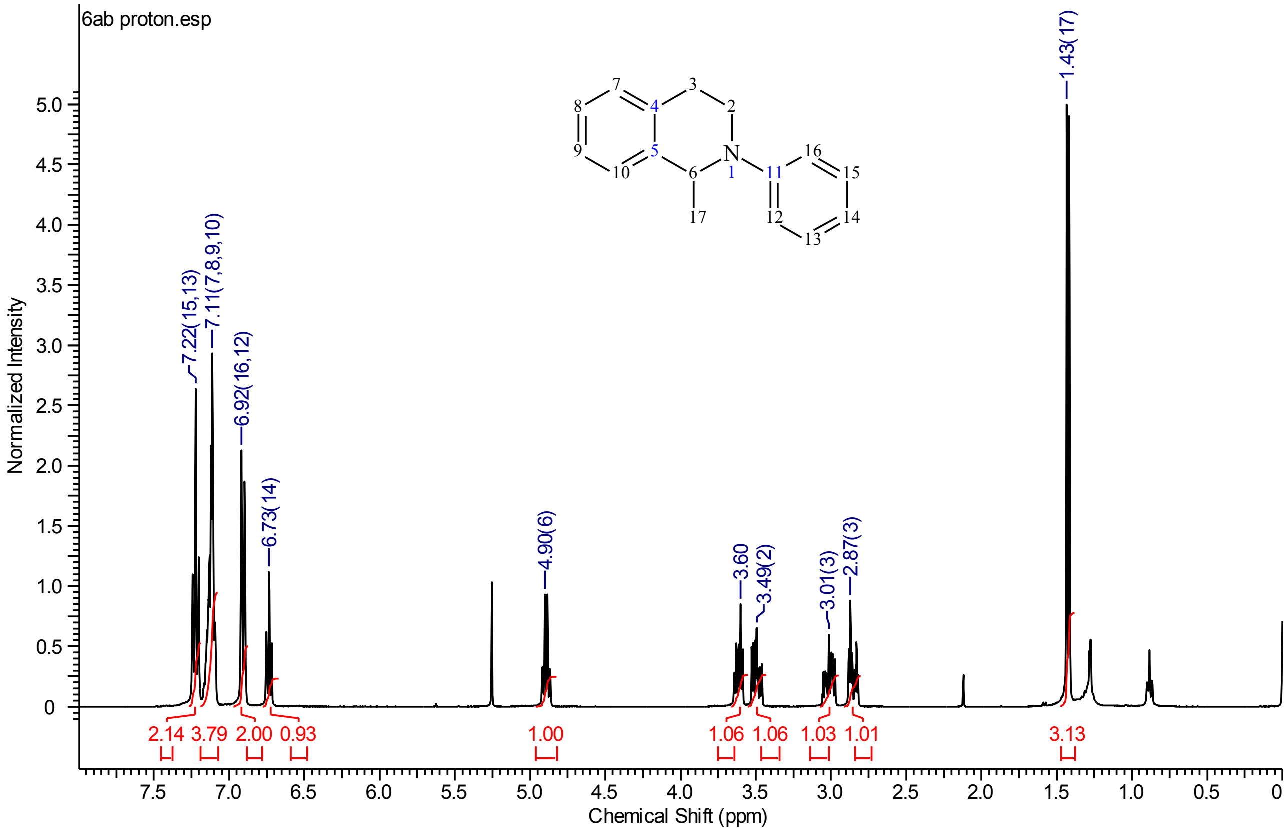
6ab proton.esp
Normalized Intensity
Chemical Shift (ppm)
7.22(15,13)
7.11(7,8,9,10)
6.92(16,12)
6.73(14)
4.90(6)
3.60
3.49(2)
3.01(3)
2.87(3)
1.43(17)
2.14
3.79
2.00
0.93
1.00
1.06
1.06
1.03
1.01
3.13

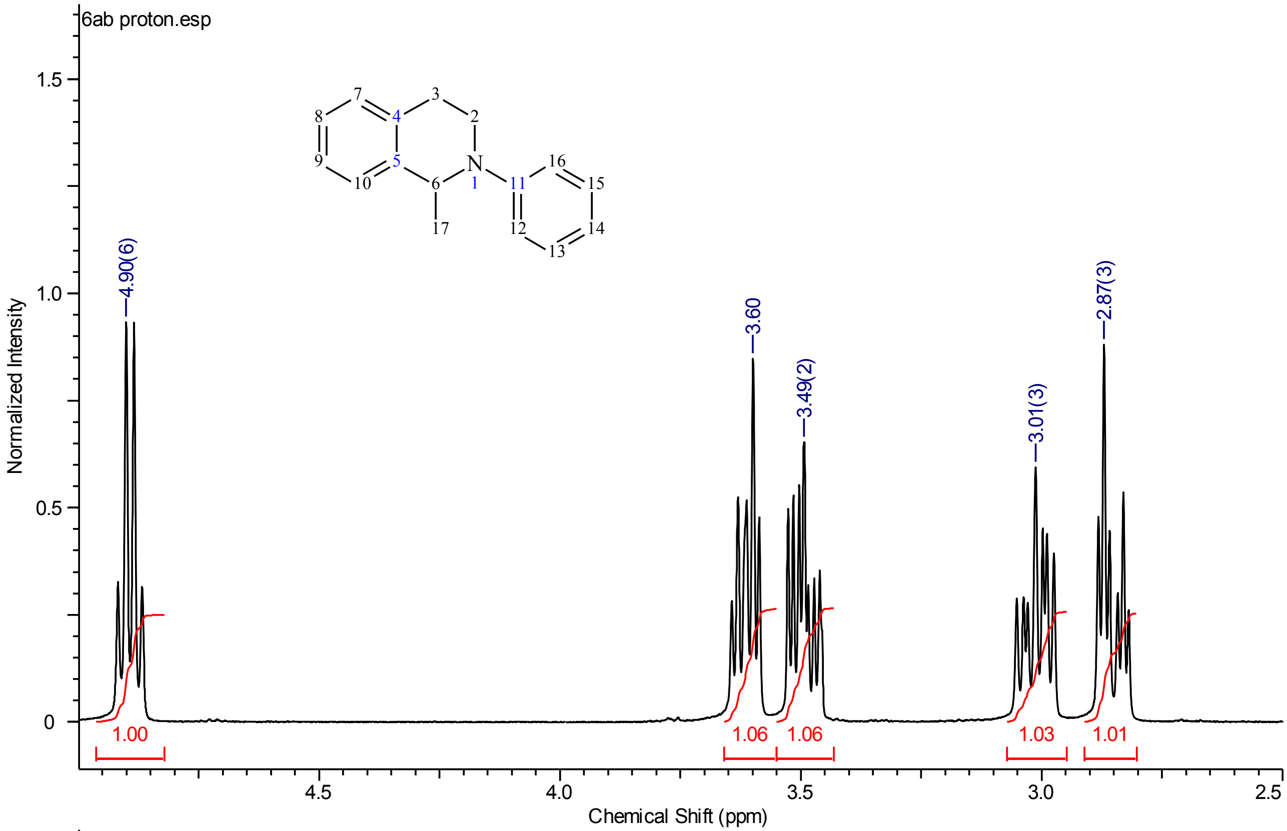
6ab proton.esp
4.90(6)
3.60
3.49(2)
3.01(3)
2.87(3)
1.00
1.06
1.06
1.03
1.01
Normalized Intensity
Chemical Shift (ppm)

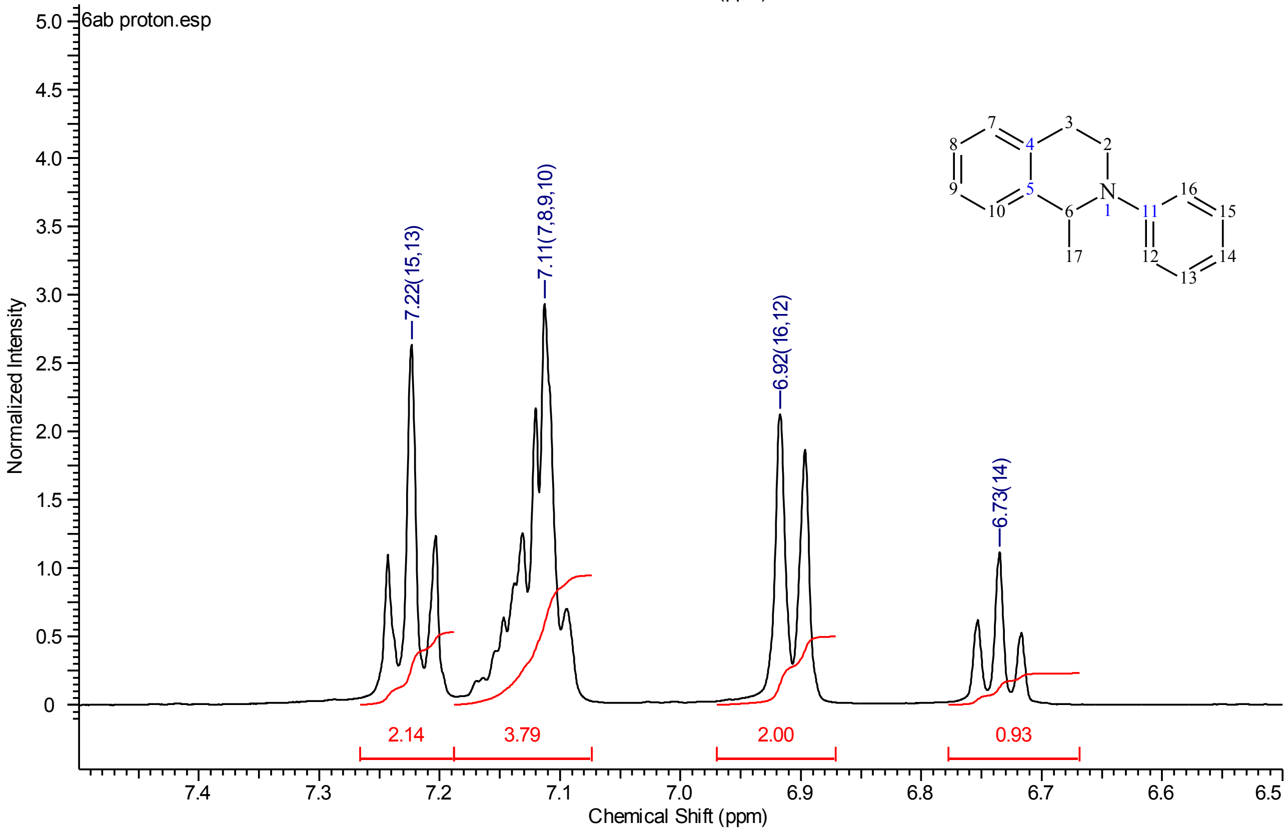
6ab proton.esp
7.22(15,13)
7.11(7,8,9,10)
6.92(16,12)
6.73(14)
2.14
3.79
2.00
0.93
Normalized Intensity
Chemical Shift (ppm)

^{13}C NMR – **6ab**

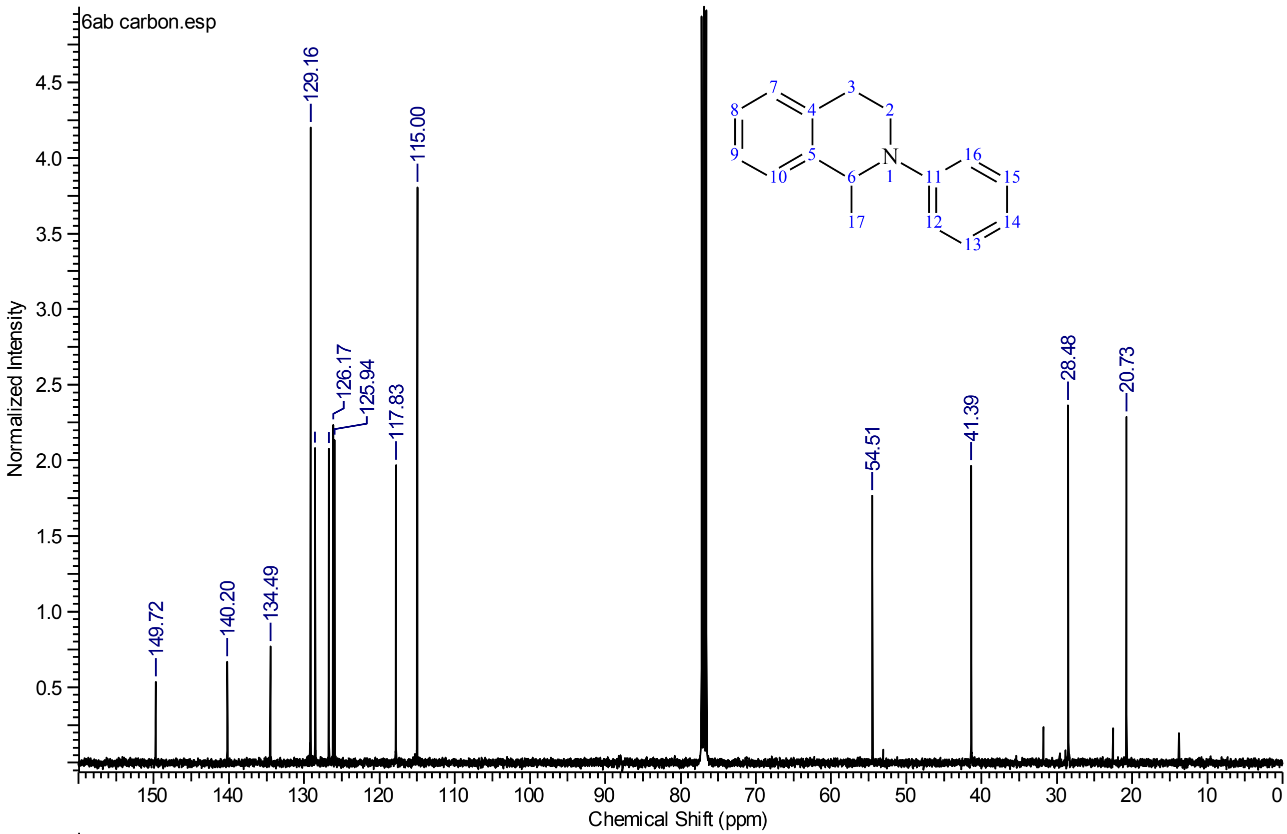
6ab carbon.esp
149.72
140.20
134.49
129.16
126.17
125.94
117.83
115.00
54.51
41.39
28.48
20.73
Normalized Intensity
Chemical Shift (ppm)
N
1
2
3
4
5
6
7
8
9
10
11
12
13
14
15
16
17

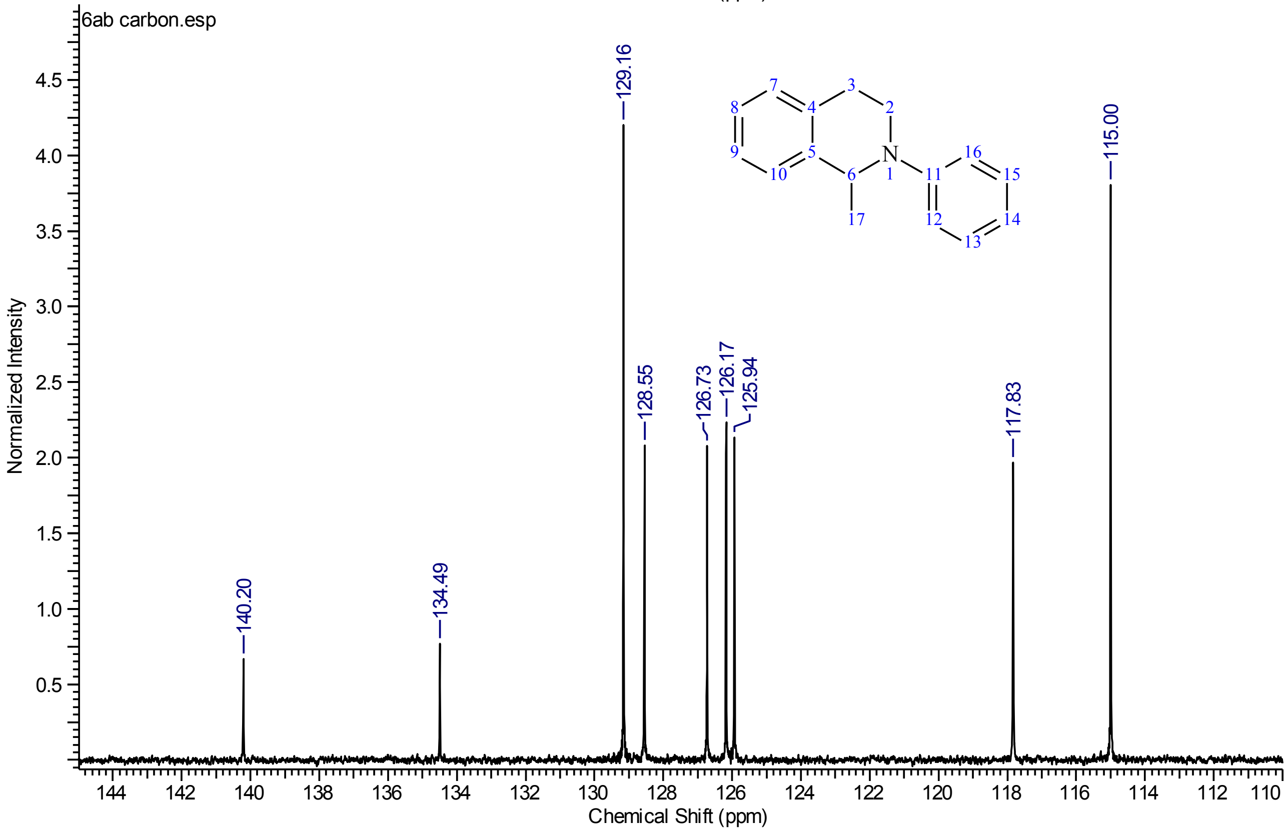
6ab carbon.esp
140.20
134.49
129.16
128.55
126.73
126.17
125.94
117.83
115.00
Normalized Intensity
Chemical Shift (ppm)
N
1
2
3
4
5
6
7
8
9
10
11
12
13
14
15
16
17

^{1}H NMR – **6ac**

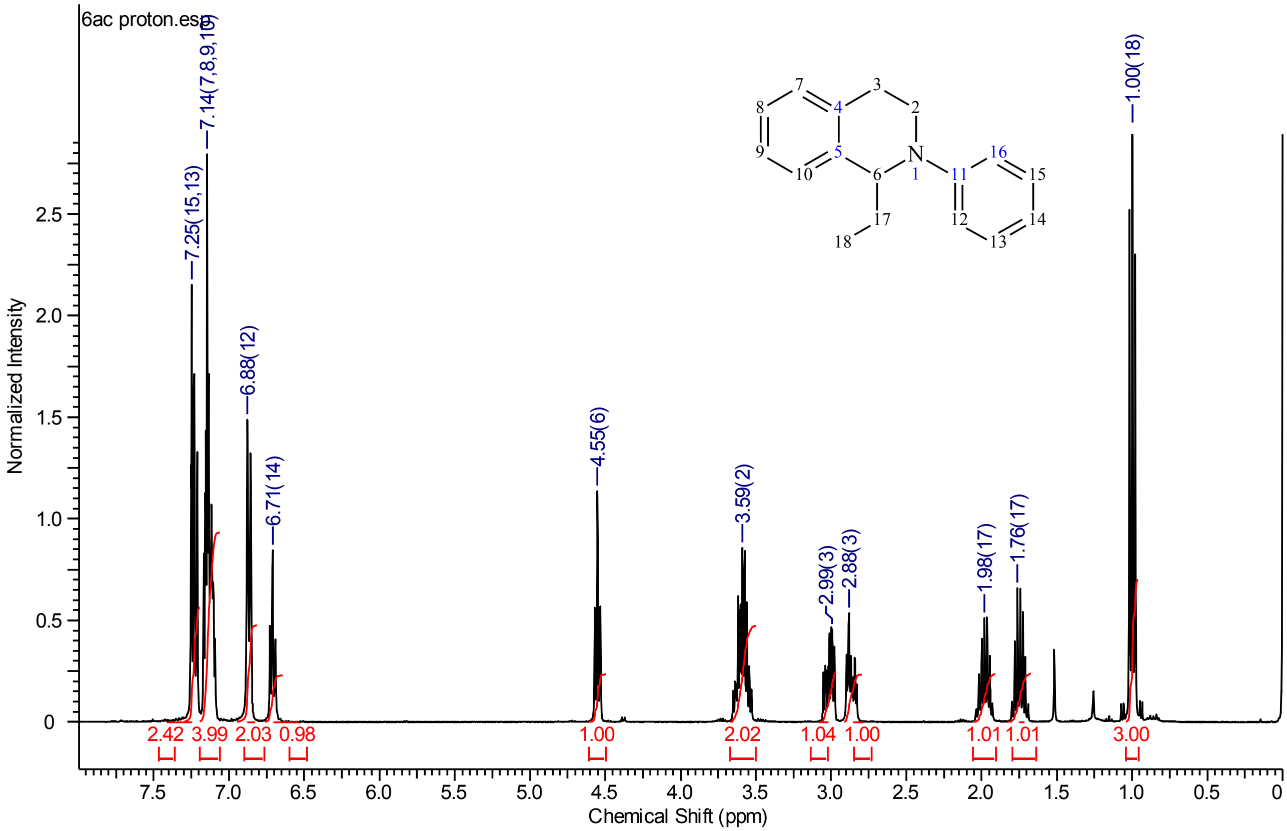
6ac proton.esp
7.25(15,13)
7.14(7,8,9,10)
6.88(12)
6.71(14)
4.55(6)
3.59(2)
2.99(3)
2.88(3)
1.98(17)
1.76(17)
1.00(18)
2.42
3.99
2.03
0.98
1.00
2.02
1.04
1.00
1.01
1.01
3.00
Normalized Intensity
Chemical Shift (ppm)

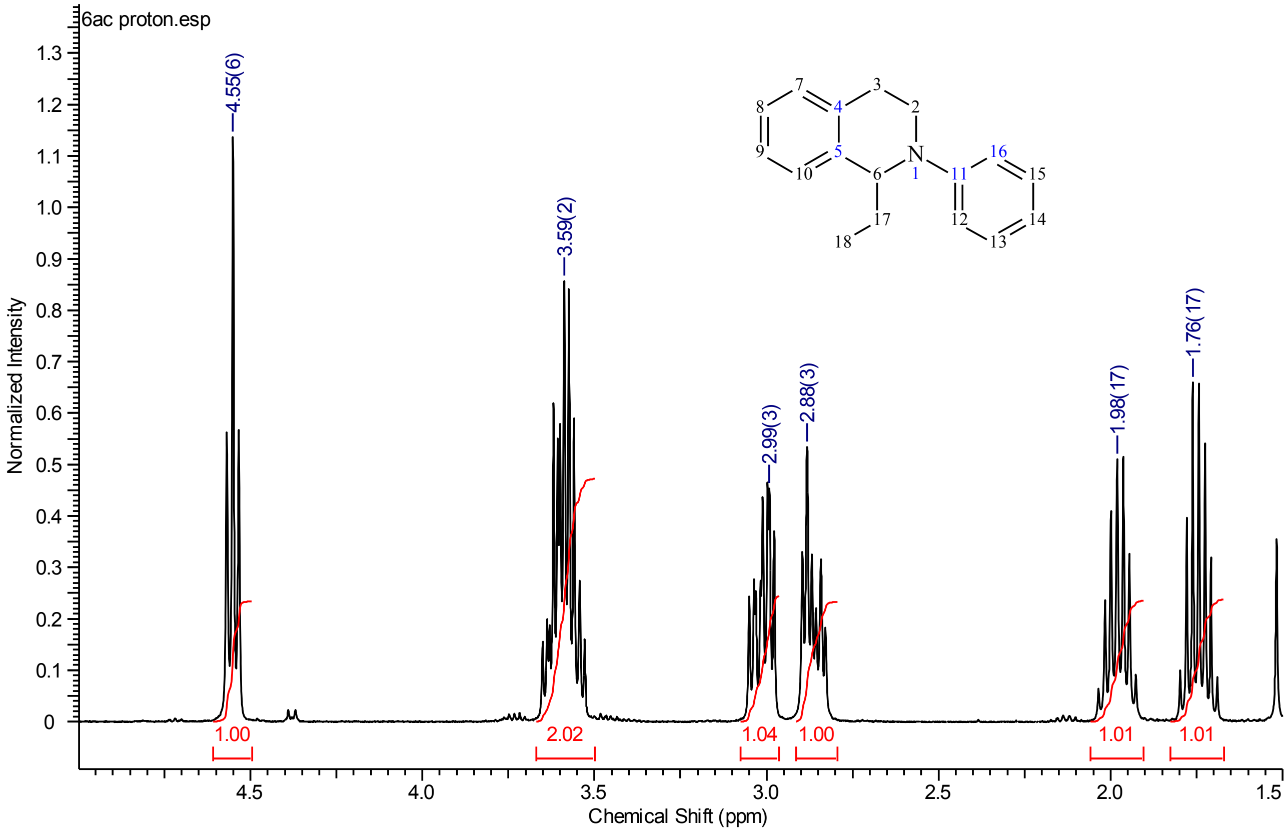
6ac proton.esp
4.55(6)
3.59(2)
2.99(3)
2.88(3)
1.98(17)
1.76(17)
1.00
2.02
1.04
1.00
1.01
1.01
Normalized Intensity
Chemical Shift (ppm)

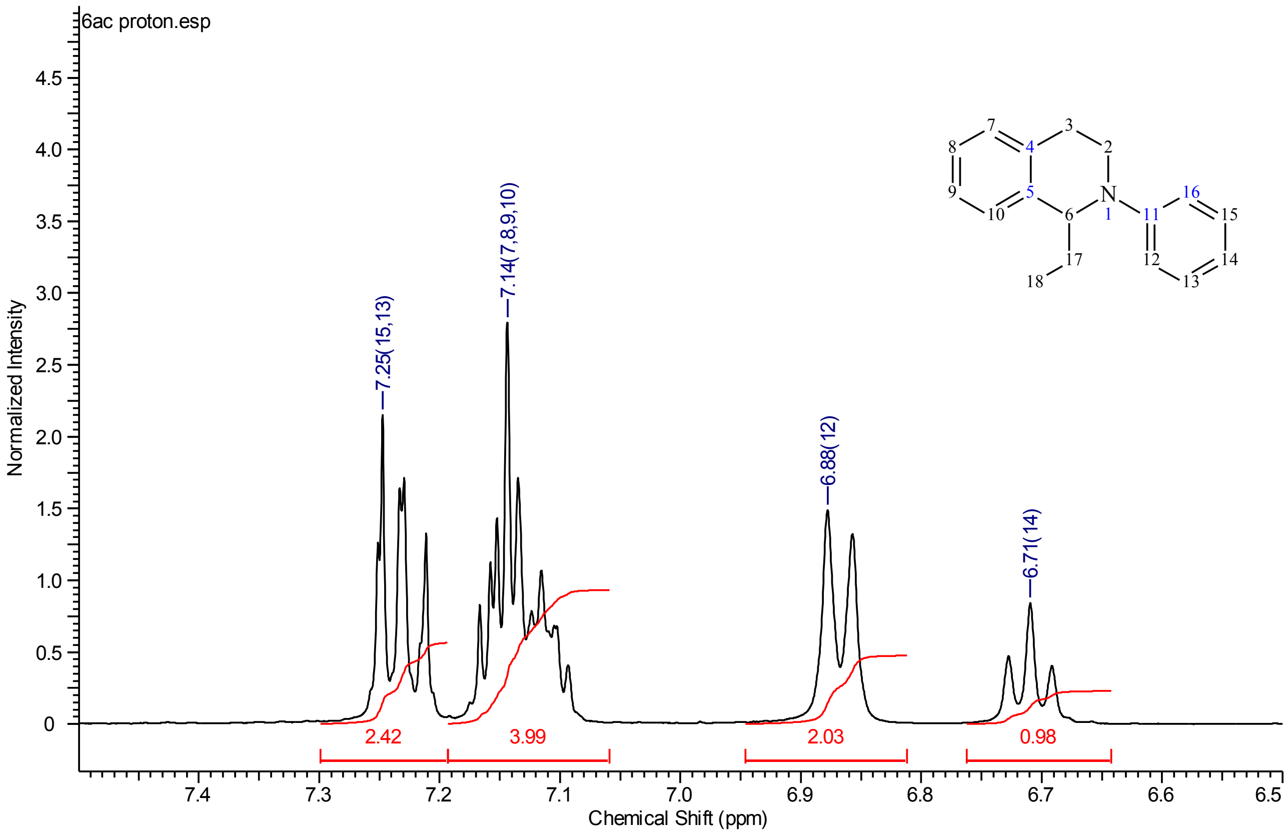
6ac proton.esp
7.25(15,13)
7.14(7,8,9,10)
6.88(12)
6.71(14)
2.42
3.99
2.03
0.98
Normalized Intensity
Chemical Shift (ppm)

^{13}C NMR – **6ac**

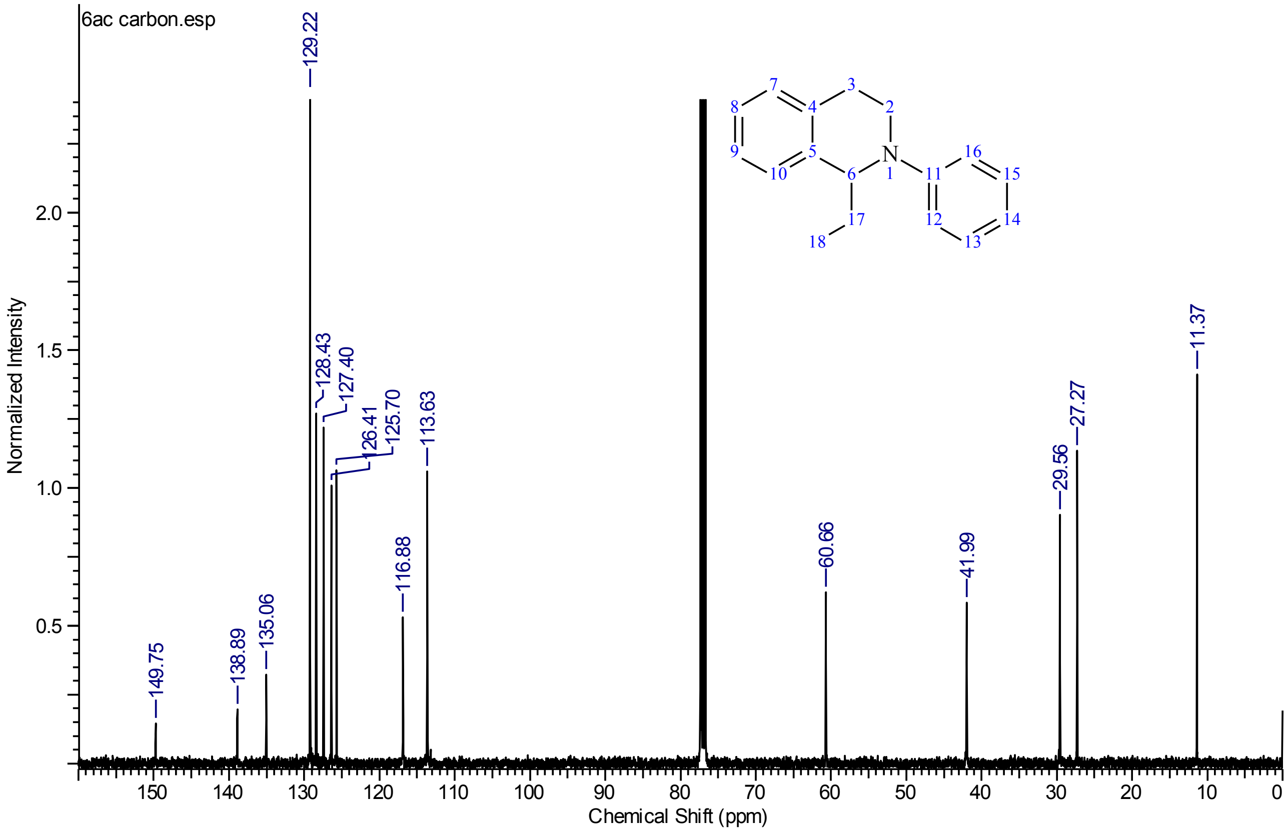
6ac carbon.esp
129.22
128.43
127.40
126.41
125.70
113.63
116.88
149.75
138.89
135.06
60.66
41.99
29.56
27.27
11.37
Normalized Intensity
Chemical Shift (ppm)

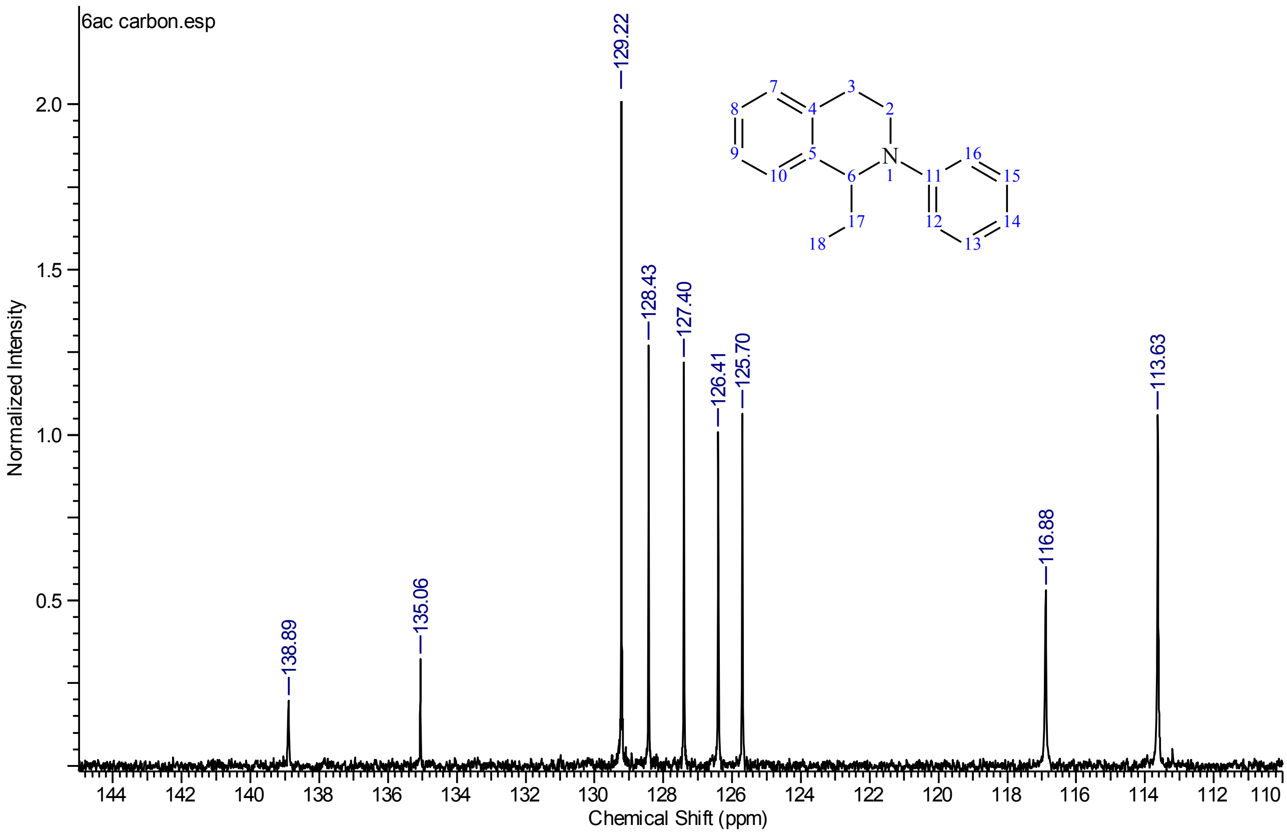
6ac carbon.esp
129.22
128.43
127.40
126.41
125.70
138.89
135.06
116.88
113.63
Normalized Intensity
Chemical Shift (ppm)

^{1}H NMR – **6ad**

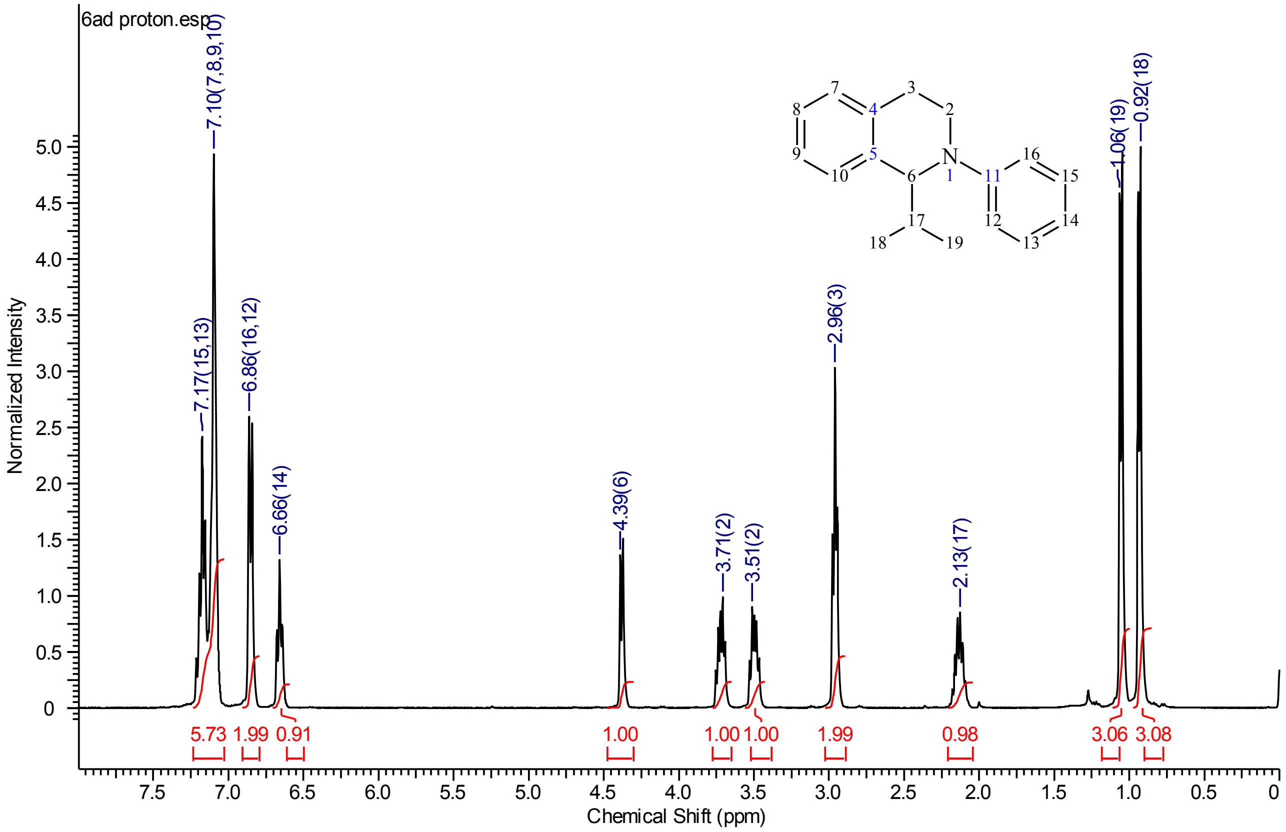
6ad proton.esp
7.10(7,8,9,10)
7.17(15,13)
6.86(16,12)
6.66(14)
4.39(6)
3.71(2)
3.51(2)
2.96(3)
2.13(17)
1.06(19)
0.92(18)
5.73
1.99
0.91
1.00
1.00
1.00
1.99
0.98
3.06
3.08
Normalized Intensity
Chemical Shift (ppm)

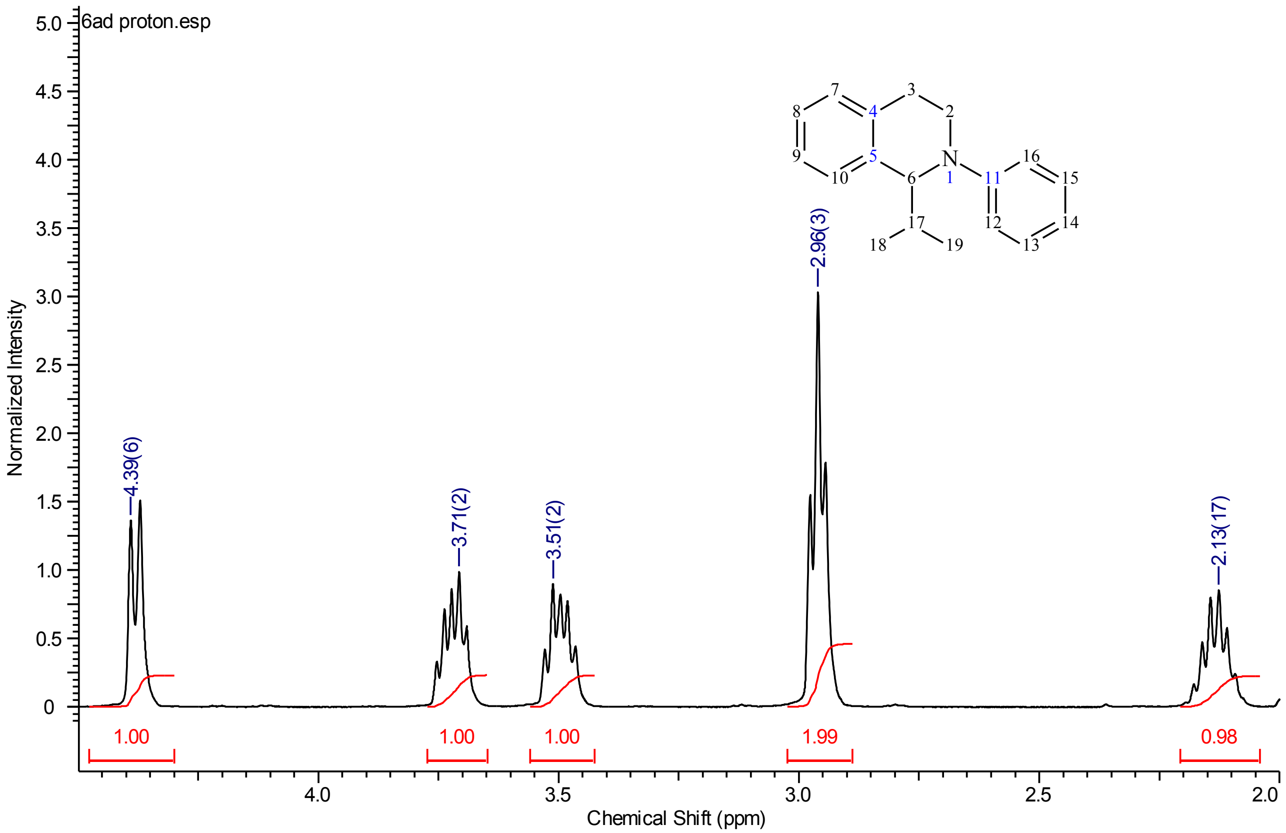
6ad proton.esp
Normalized Intensity
Chemical Shift (ppm)
4.39(6)
3.71(2)
3.51(2)
2.96(3)
2.13(17)
1.00
1.00
1.00
1.99
0.98

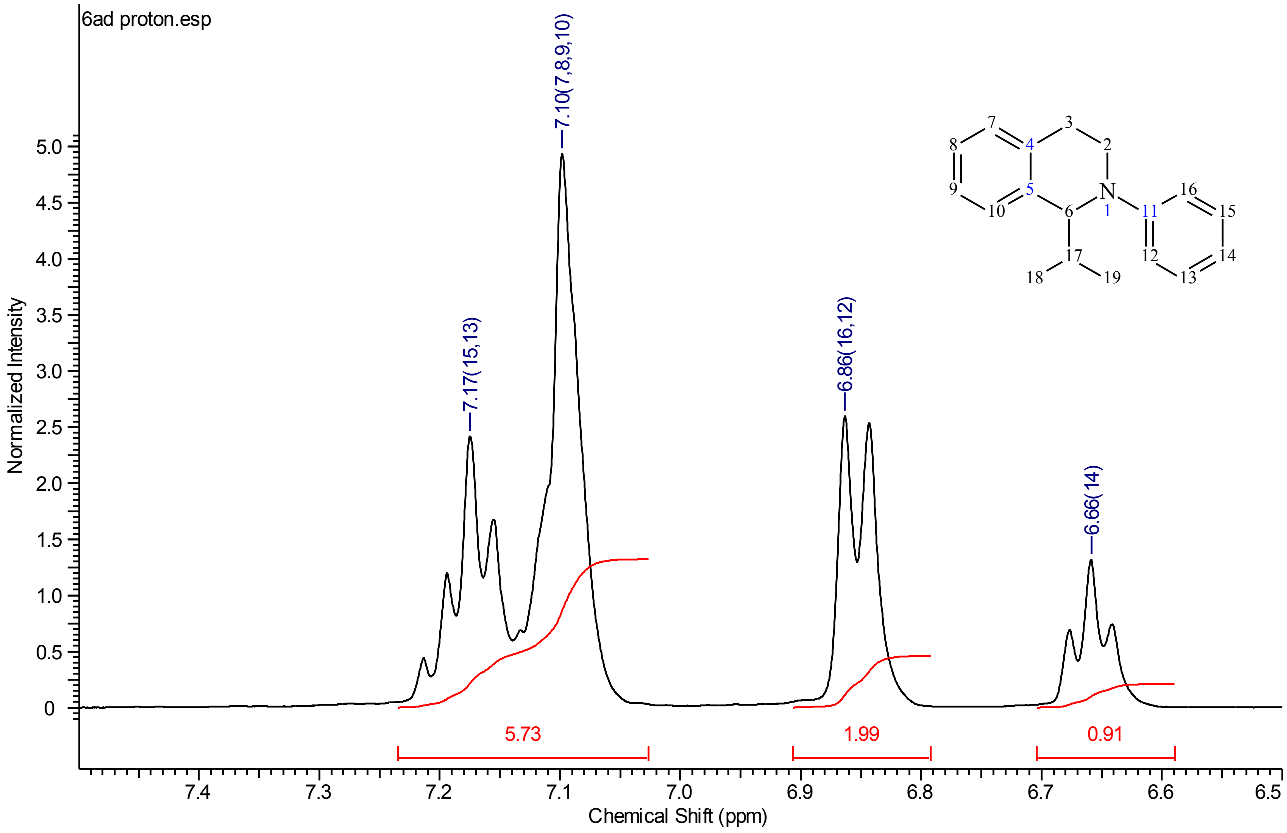
6ad proton.esp
Normalized Intensity
Chemical Shift (ppm)
7.17(15,13)
7.10(7,8,9,10)
6.86(16,12)
6.66(14)
5.73
1.99
0.91

^{13}C NMR – **6ad**

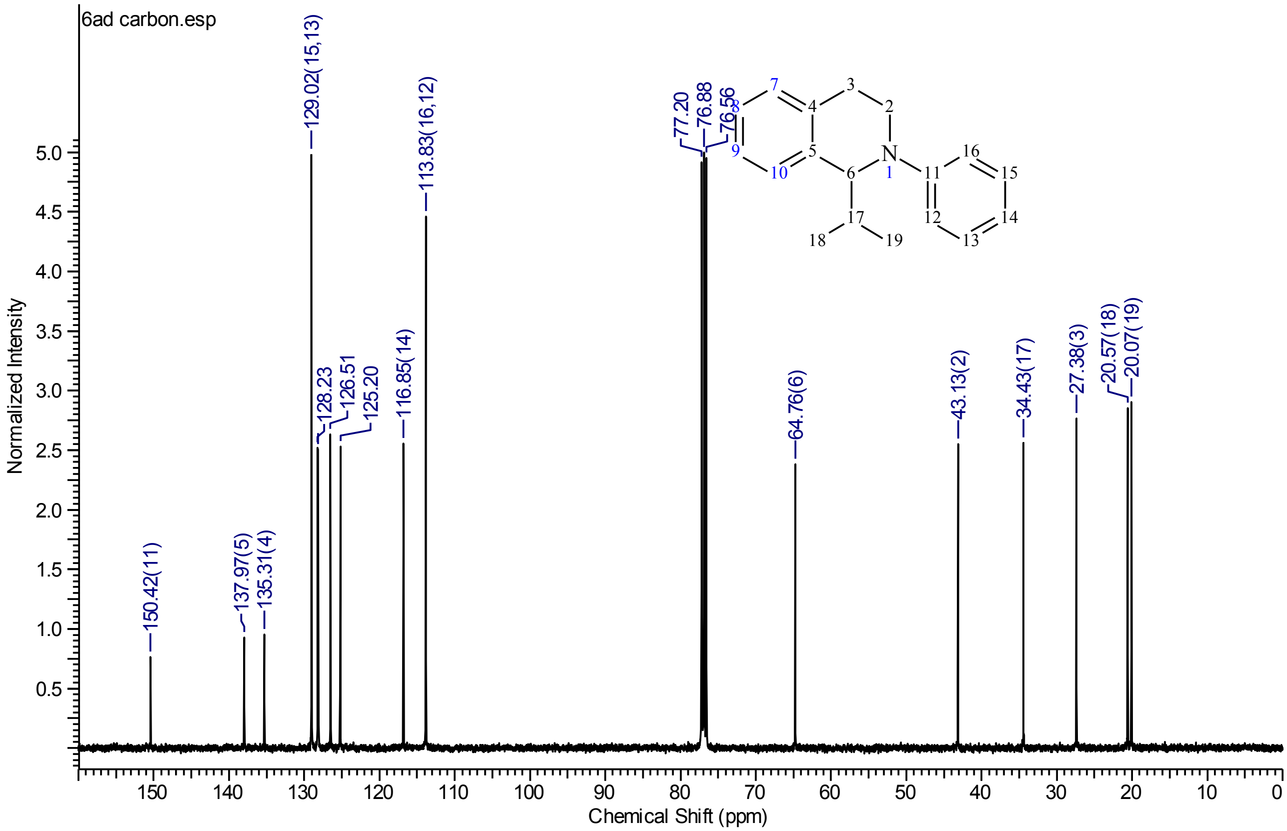
6ad carbon.esp
150.42(11)
137.97(5)
135.31(4)
129.02(15,13)
128.23
126.51
125.20
116.85(14)
113.83(16,12)
77.20
76.88
76.56
64.76(6)
43.13(2)
34.43(17)
27.38(3)
20.57(18)
20.07(19)
Normalized Intensity
Chemical Shift (ppm)

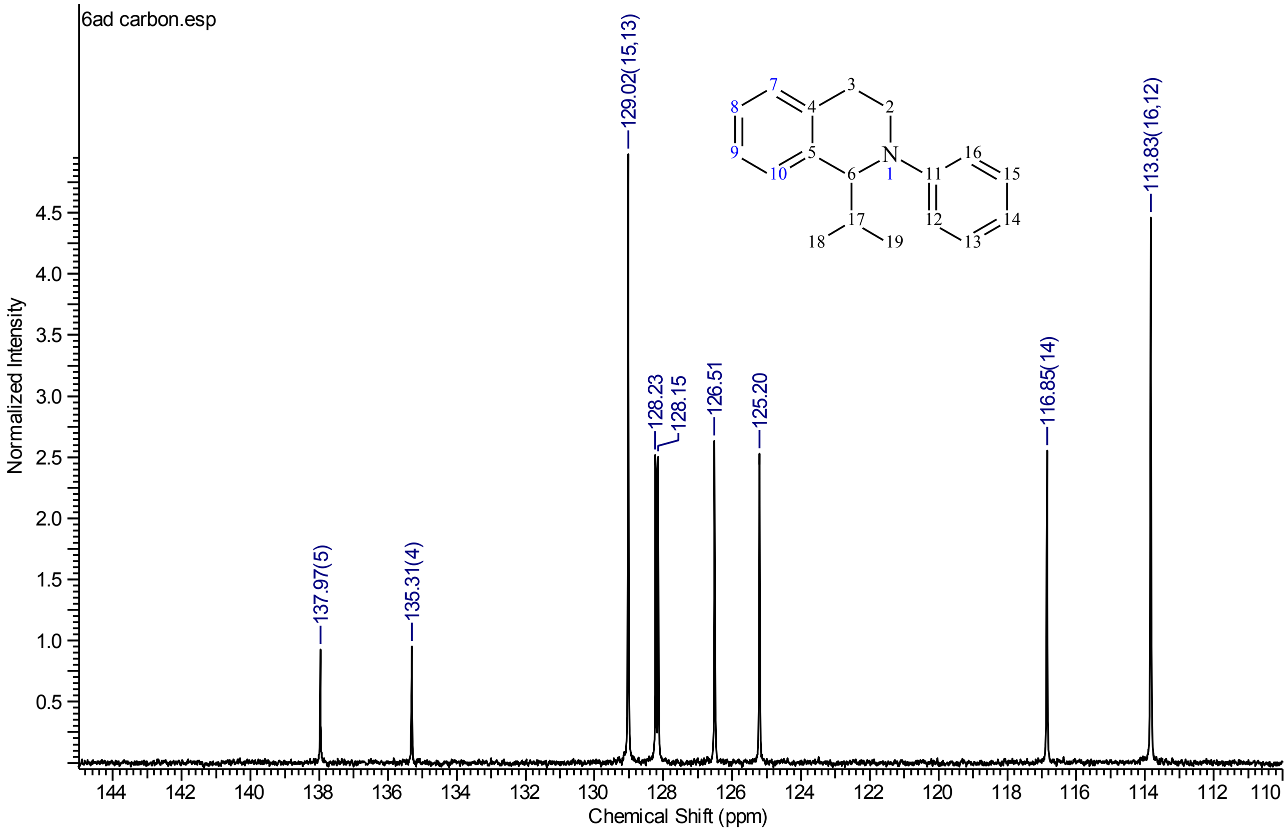
6ad carbon.esp
137.97(5)
135.31(4)
129.02(15,13)
128.23
128.15
126.51
125.20
116.85(14)
113.83(16,12)
Normalized Intensity
Chemical Shift (ppm)

^{1}H NMR – **6ae**

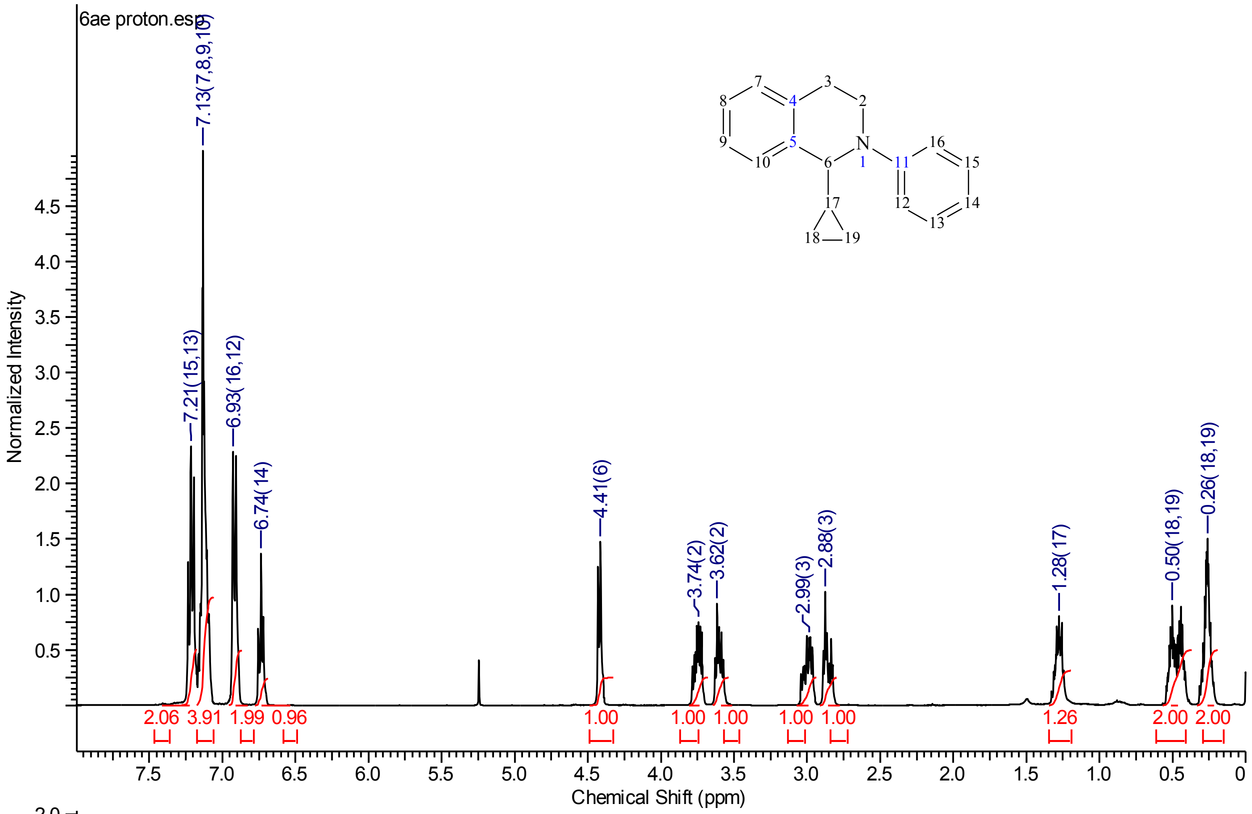
6ae proton.esp
7.13(7,8,9,10)
7.21(15,13)
6.93(16,12)
6.74(14)
4.41(6)
3.74(2)
3.62(2)
2.99(3)
2.88(3)
1.28(17)
0.50(18,19)
0.26(18,19)
2.06
3.91
1.99
0.96
1.00
1.00
1.00
1.00
1.00
1.26
2.00
2.00
Normalized Intensity
Chemical Shift (ppm)

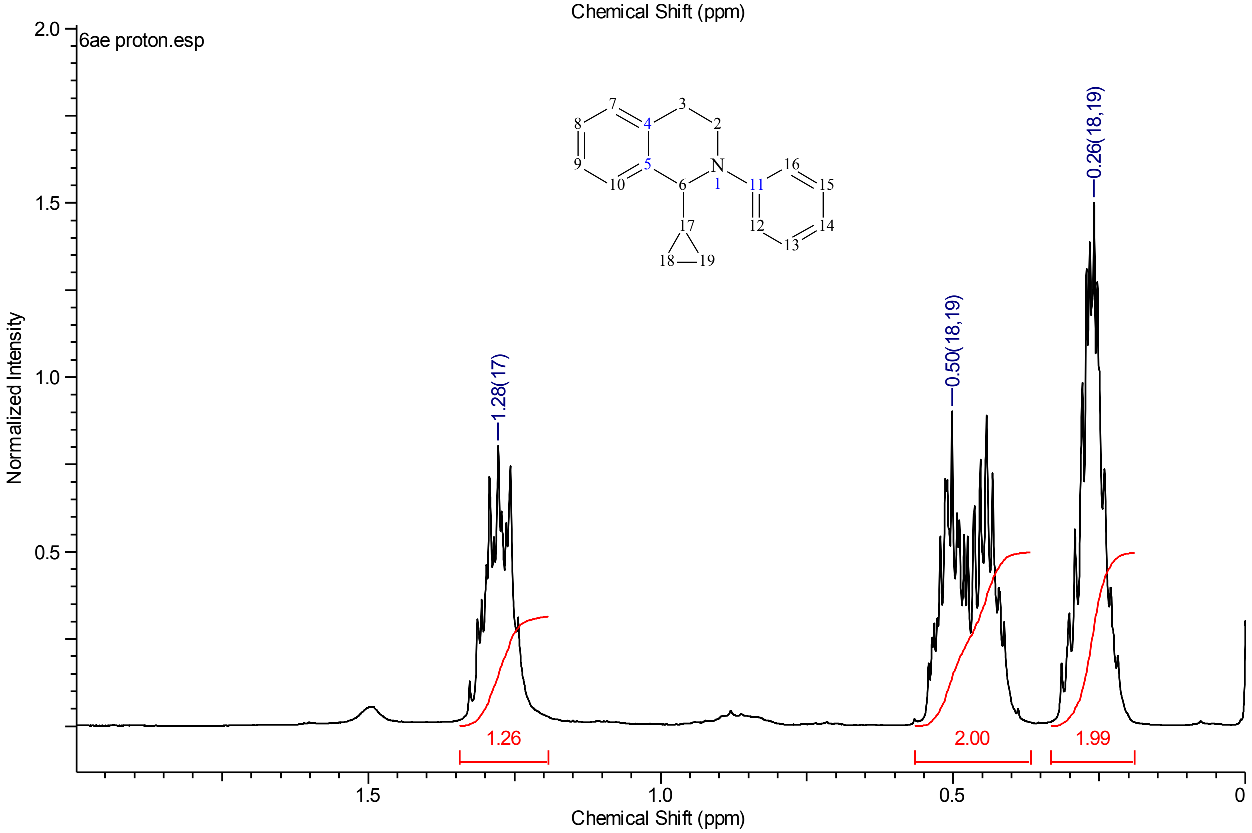
6ae proton.esp
1.28(17)
0.50(18,19)
0.26(18,19)
1.26
2.00
1.99
Normalized Intensity
Chemical Shift (ppm)

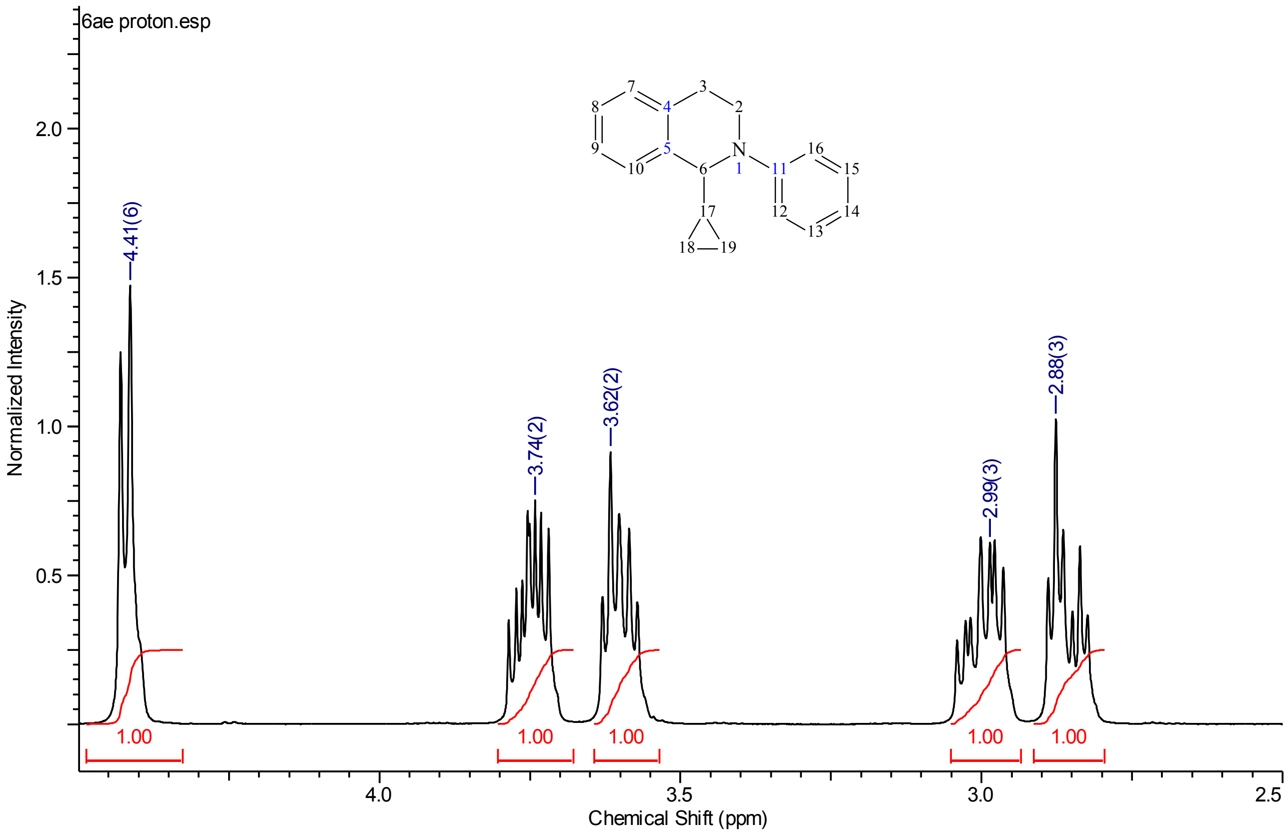
6ae proton.esp
Normalized Intensity
Chemical Shift (ppm)
4.41(6)
3.74(2)
3.62(2)
2.99(3)
2.88(3)
1.00
1.00
1.00
1.00
1.00

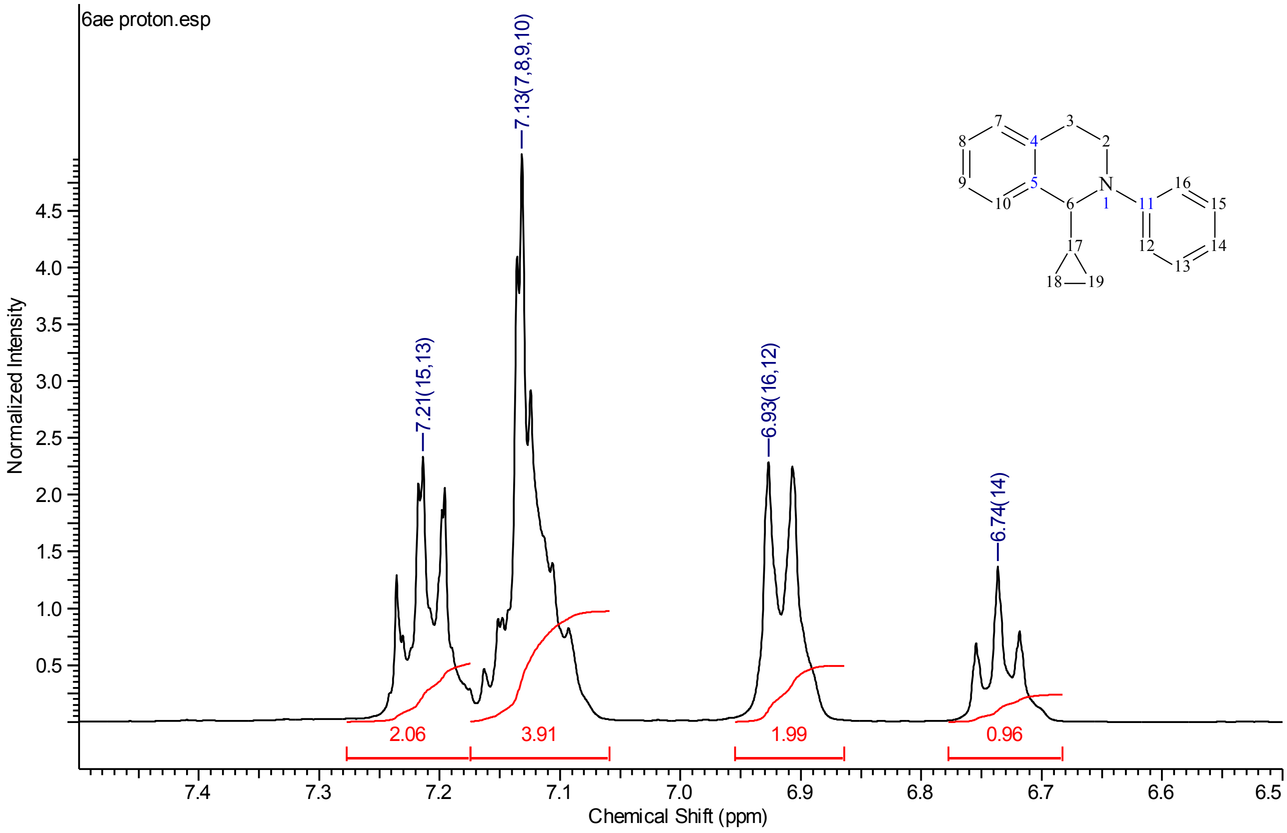
6ae proton.esp
Normalized Intensity
Chemical Shift (ppm)
7.21(15,13)
7.13(7,8,9,10)
6.93(16,12)
6.74(14)
2.06
3.91
1.99
0.96

^{13}C NMR – **6ae**

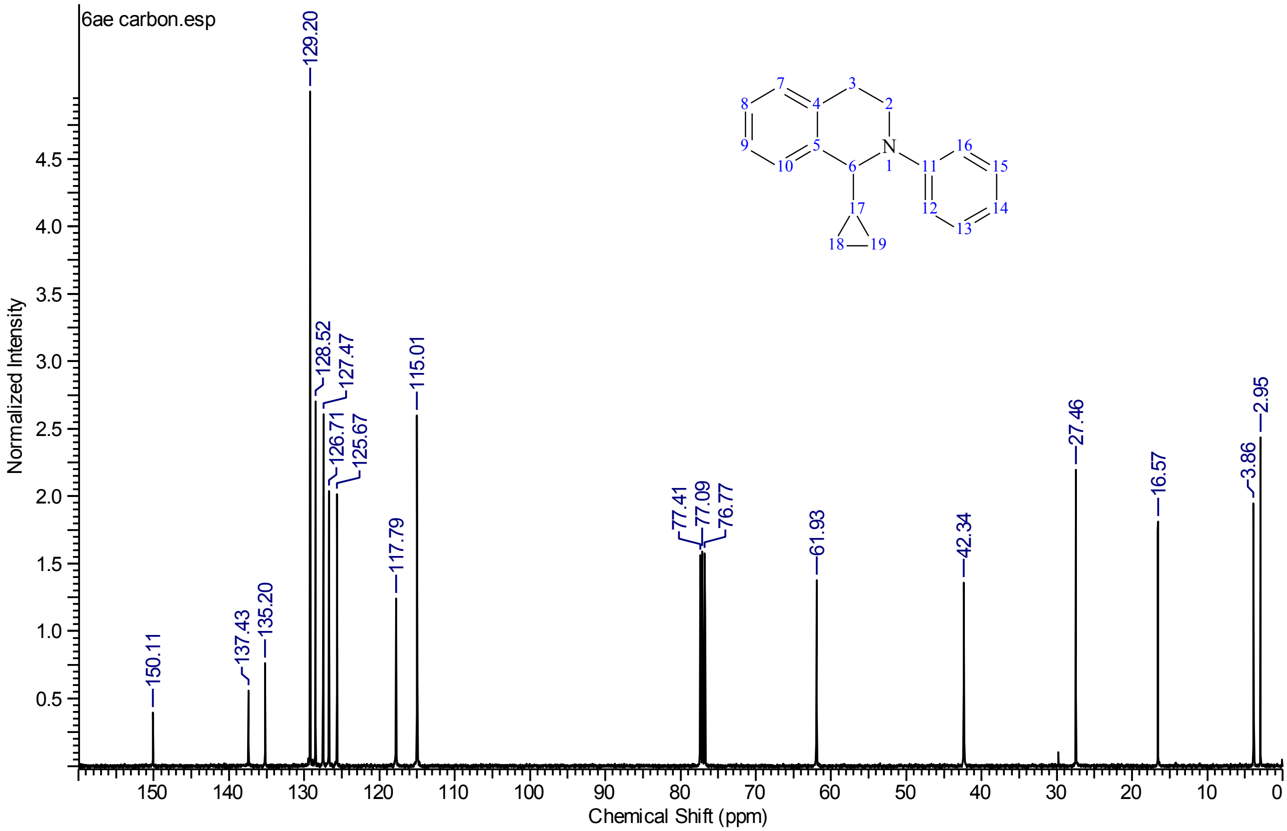
6ae carbon.esp
129.20
128.52
127.47
126.71
125.67
115.01
117.79
150.11
137.43
135.20
77.41
77.09
76.77
61.93
42.34
27.46
16.57
3.86
2.95
N
Normalized Intensity
Chemical Shift (ppm)

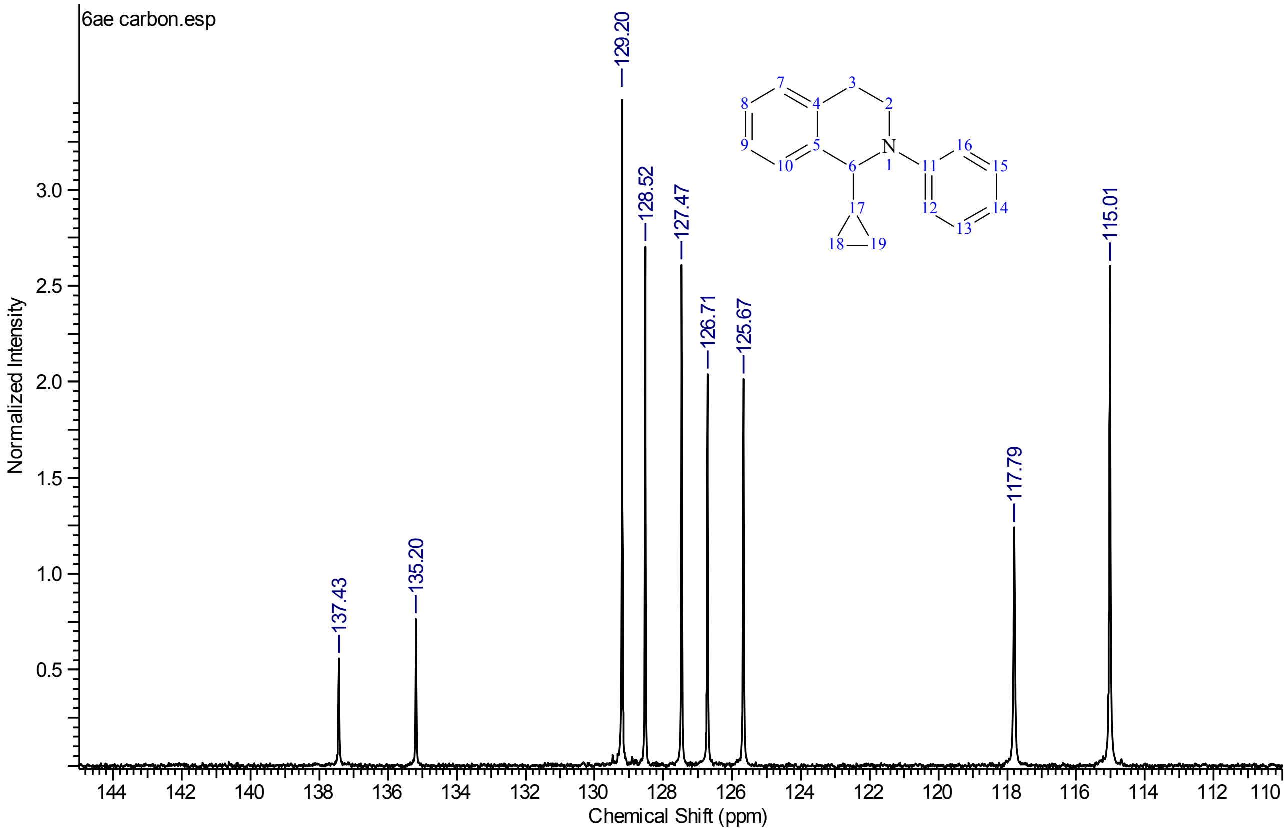
6ae carbon.esp
129.20
128.52
127.47
126.71
125.67
117.79
115.01
137.43
135.20
N
Normalized Intensity
Chemical Shift (ppm)

^{1}H NMR – **6af**

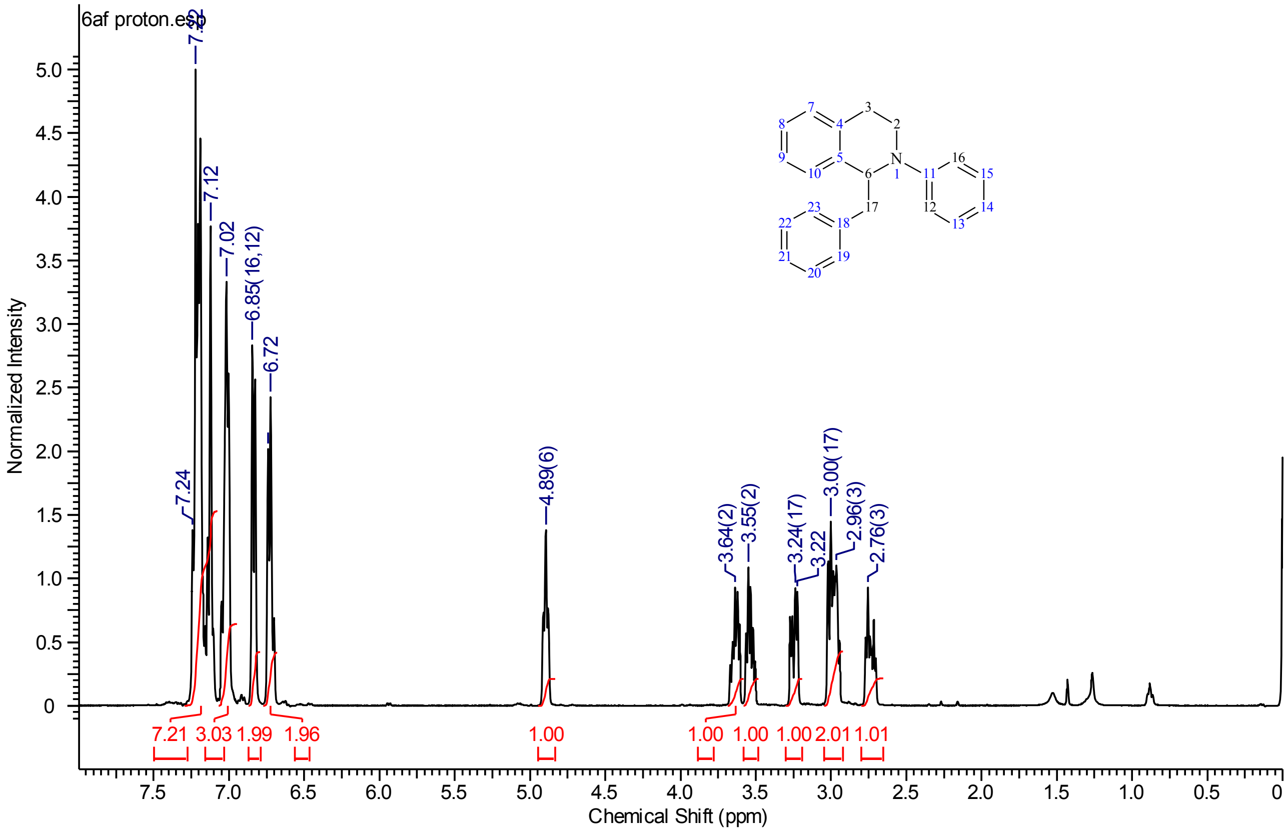
6af proton.esp
Normalized Intensity
Chemical Shift (ppm)
7.24
7.20
7.12
7.02
6.85(16,12)
6.72
4.89(6)
3.64(2)
3.55(2)
3.24(17)
3.22
3.00(17)
2.96(3)
2.76(3)
7.21
3.03
1.99
1.96
1.00
1.00
1.00
1.00
2.01
1.01

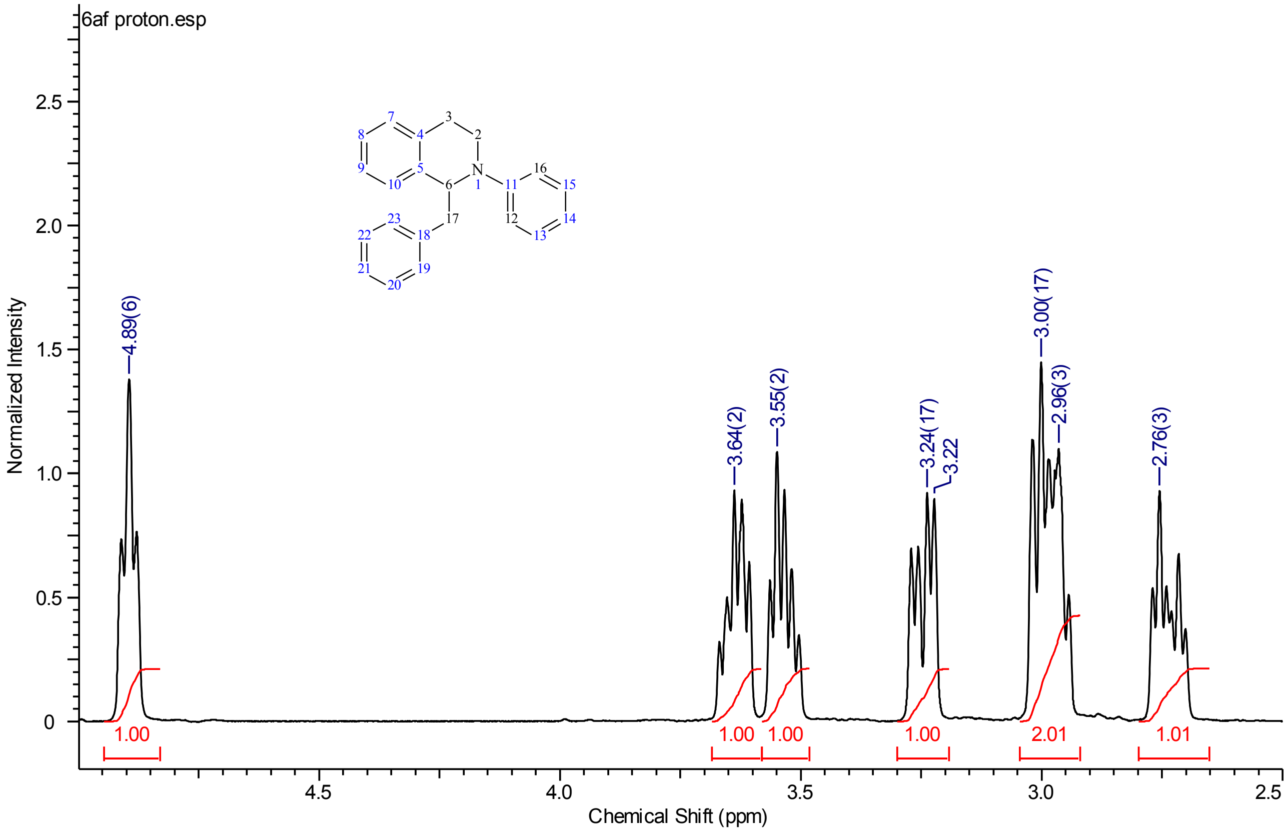
6af proton.esp
Normalized Intensity
Chemical Shift (ppm)
4.89(6)
3.64(2)
3.55(2)
3.24(17)
3.22
3.00(17)
2.96(3)
2.76(3)
1.00
1.00
1.00
1.00
2.01
1.01

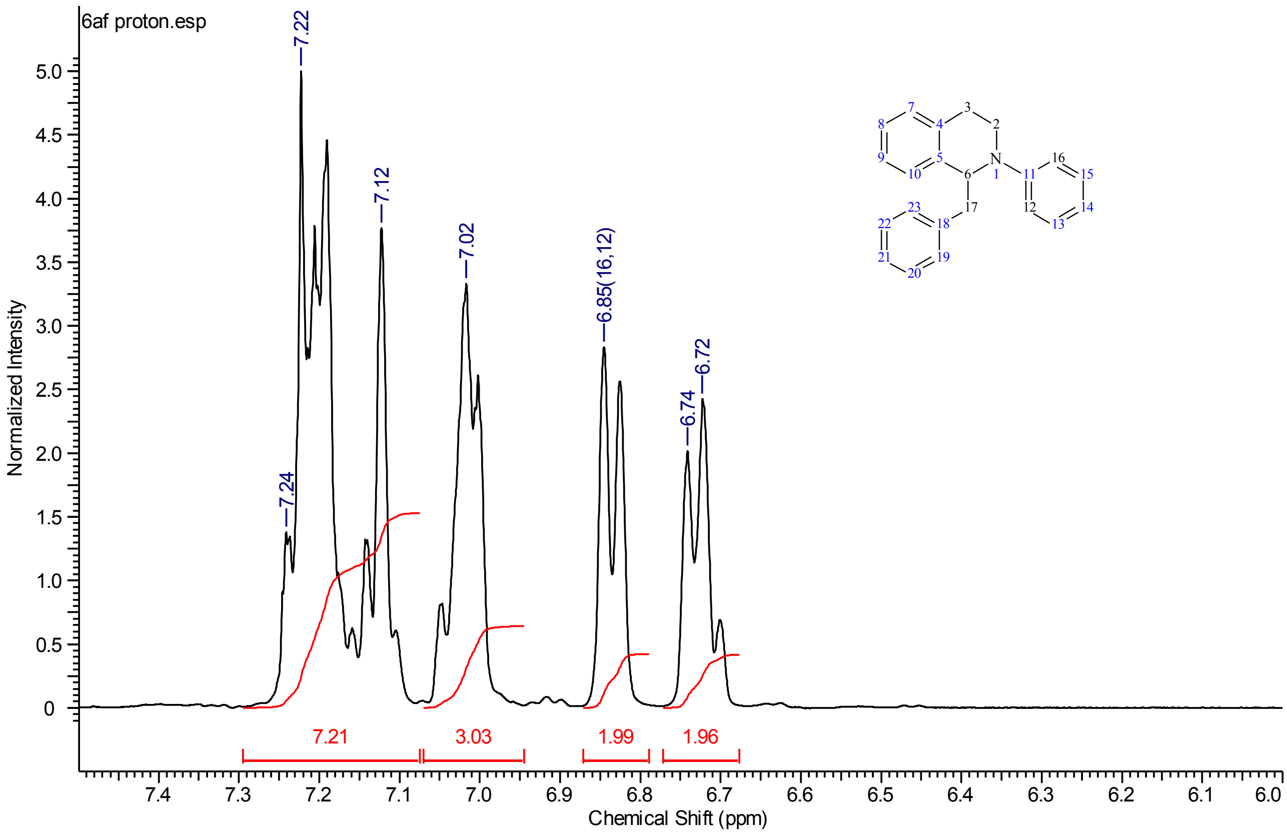
6af proton.esp
7.24
7.22
7.12
7.02
6.85(16,12)
6.74
6.72
7.21
3.03
1.99
1.96
Normalized Intensity
Chemical Shift (ppm)

^{13}C NMR – **6af**

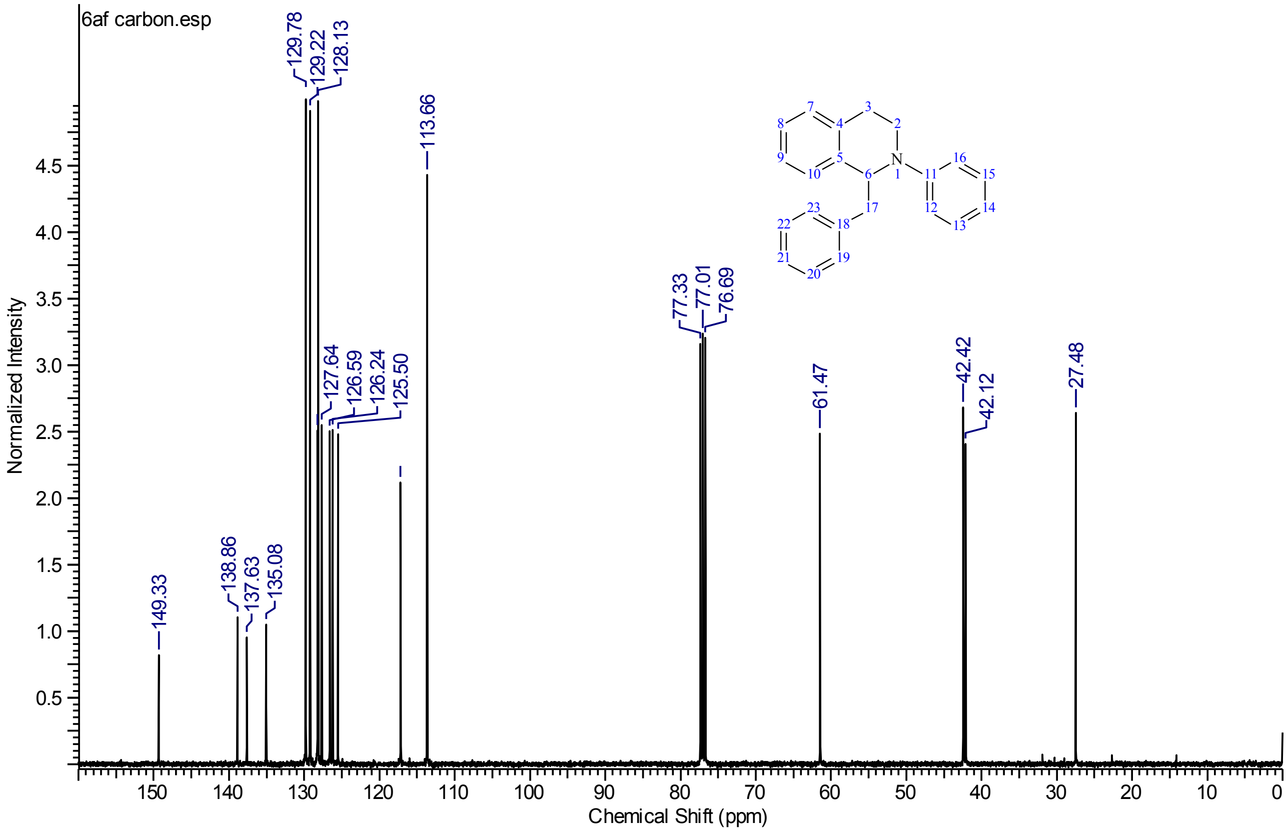
6af carbon.esp
149.33
138.86
137.63
135.08
129.78
129.22
128.13
127.64
126.59
126.24
125.50
113.66
77.33
77.01
76.69
61.47
42.42
42.12
27.48
Normalized Intensity
Chemical Shift (ppm)

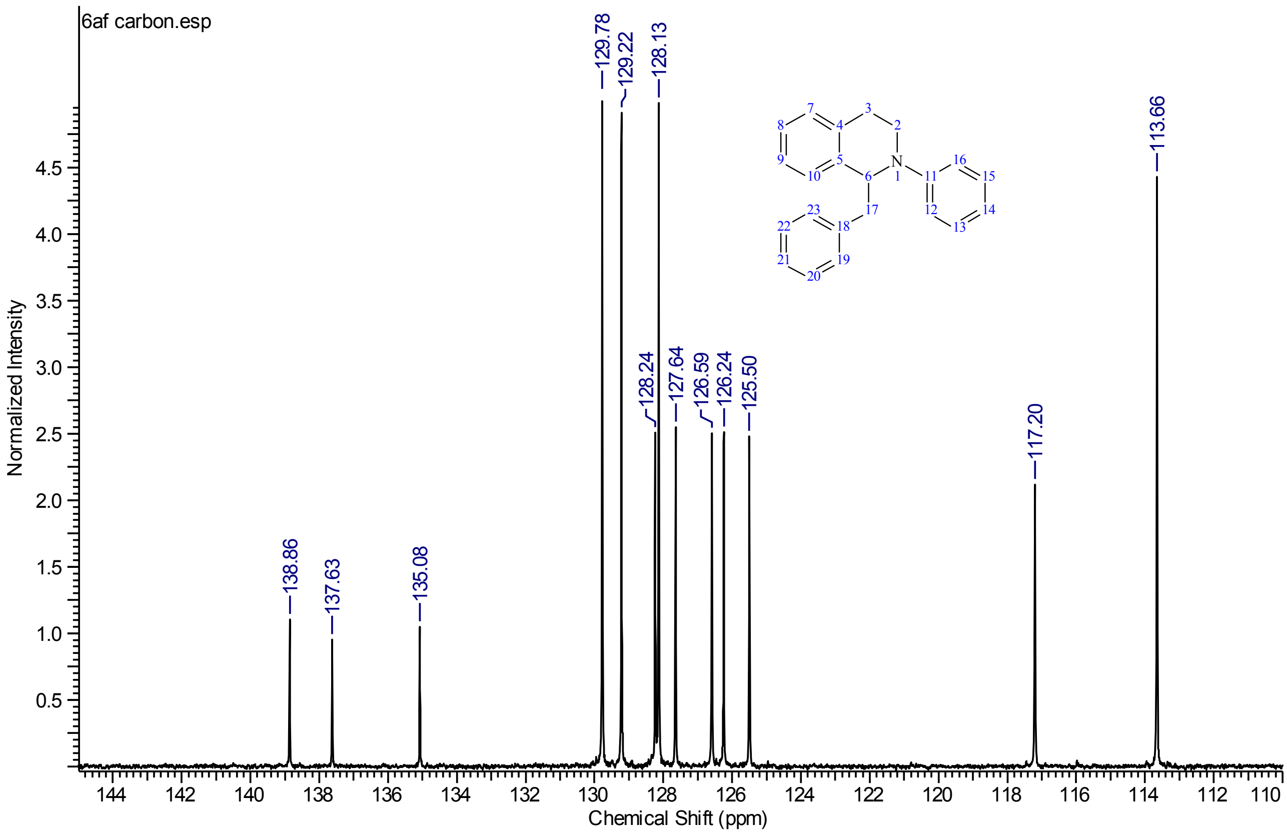
6af carbon.esp
138.86
137.63
135.08
129.78
129.22
128.24
128.13
127.64
126.59
126.24
125.50
117.20
113.66
Normalized Intensity
Chemical Shift (ppm)

^{1}H NMR – **6aj**

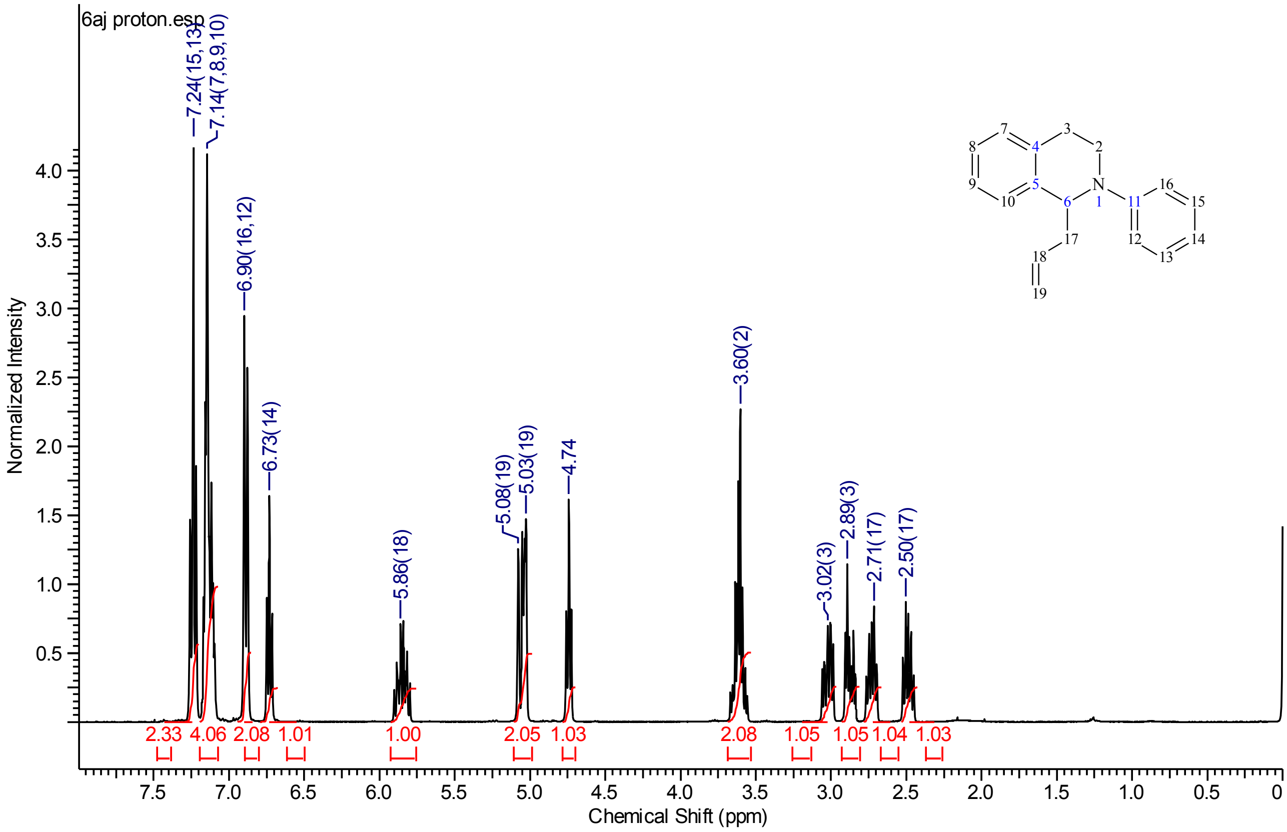
6aj proton.esp
7.24(15,13)
7.14(7,8,9,10)
6.90(16,12)
6.73(14)
5.86(18)
5.08(19)
5.03(19)
4.74
3.60(2)
3.02(3)
2.89(3)
2.71(17)
2.50(17)
2.33
4.06
2.08
1.01
1.00
2.05
1.03
2.08
1.05
1.05
1.04
1.03
Normalized Intensity
Chemical Shift (ppm)

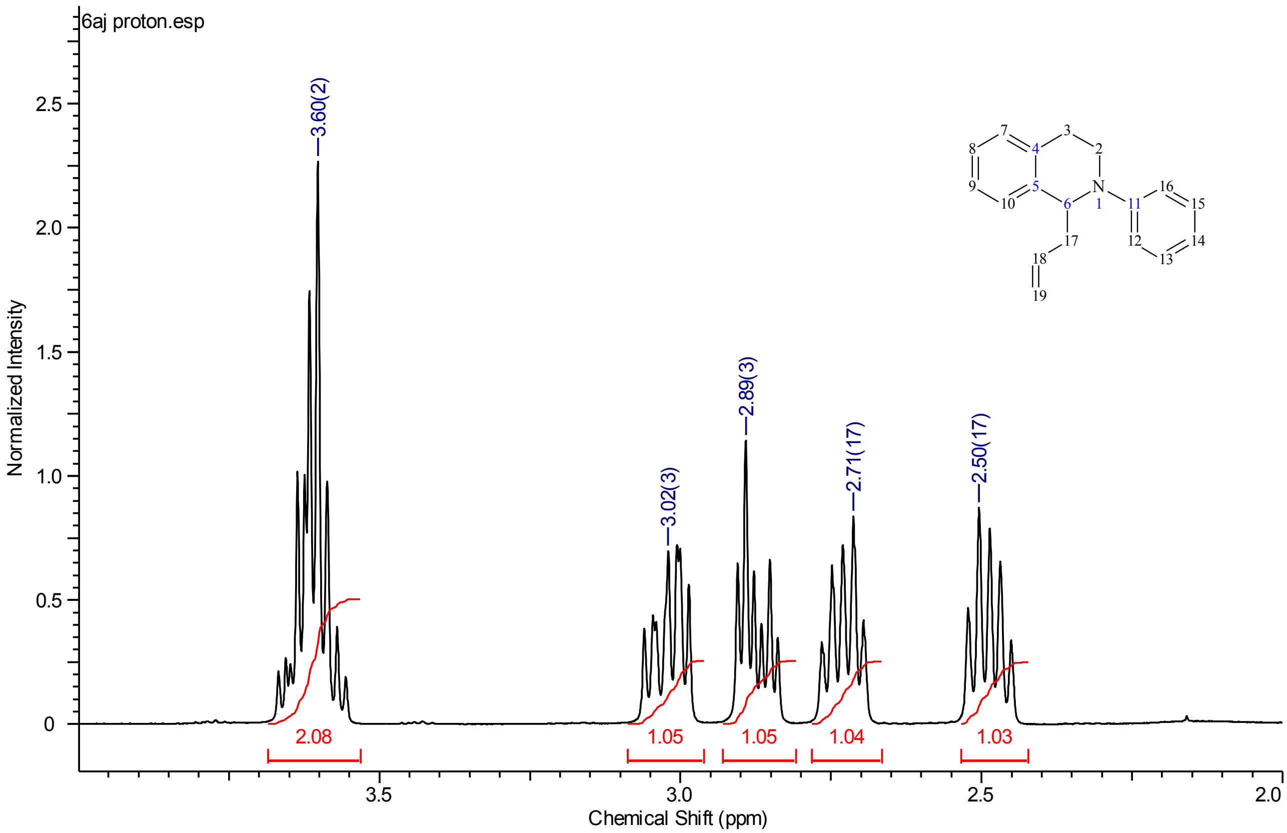
6aj proton.esp
3.60(2)
3.02(3)
2.89(3)
2.71(17)
2.50(17)
2.08
1.05
1.05
1.04
1.03
Normalized Intensity
Chemical Shift (ppm)

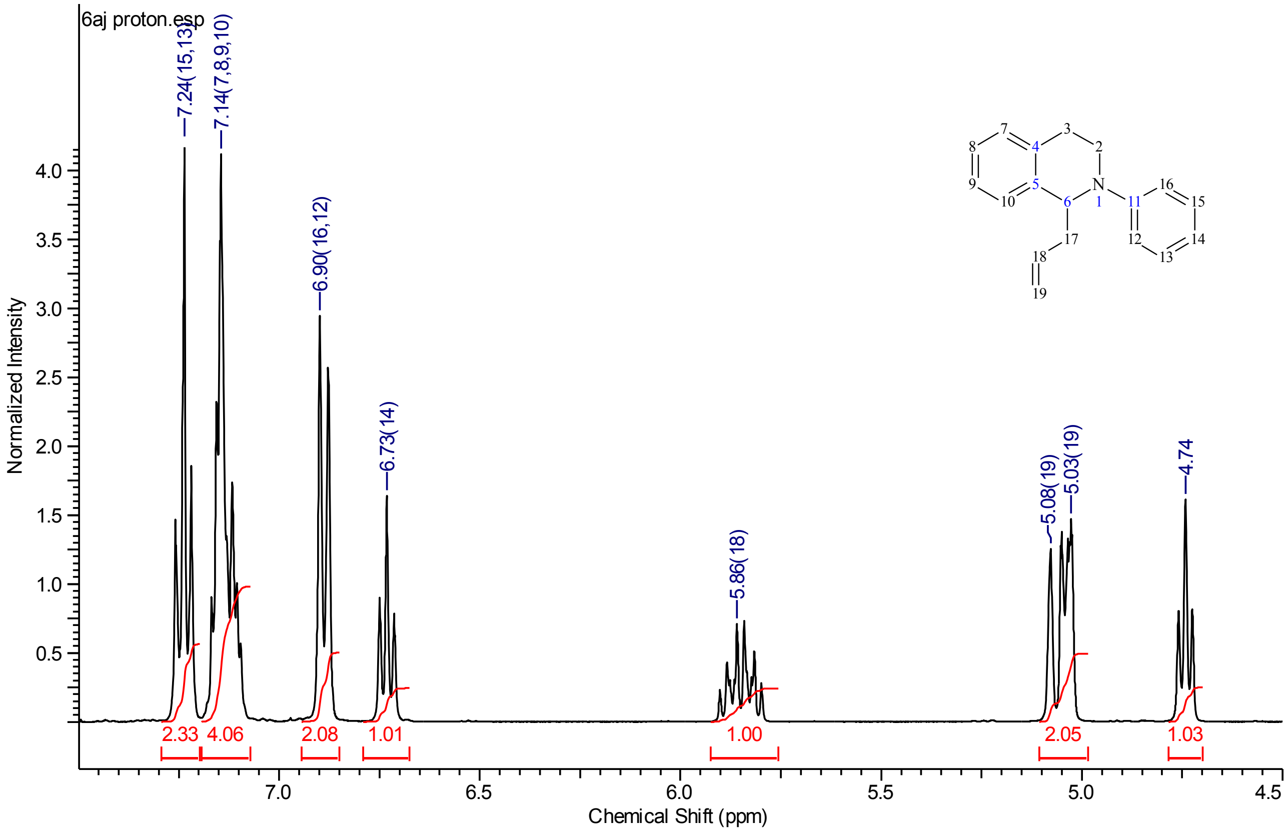
6aj proton.esp
7.24(15,13)
7.14(7,8,9,10)
6.90(16,12)
6.73(14)
5.86(18)
5.08(19)
5.03(19)
4.74
2.33
4.06
2.08
1.01
1.00
2.05
1.03
Normalized Intensity
Chemical Shift (ppm)

^{13}C NMR – **6aj**

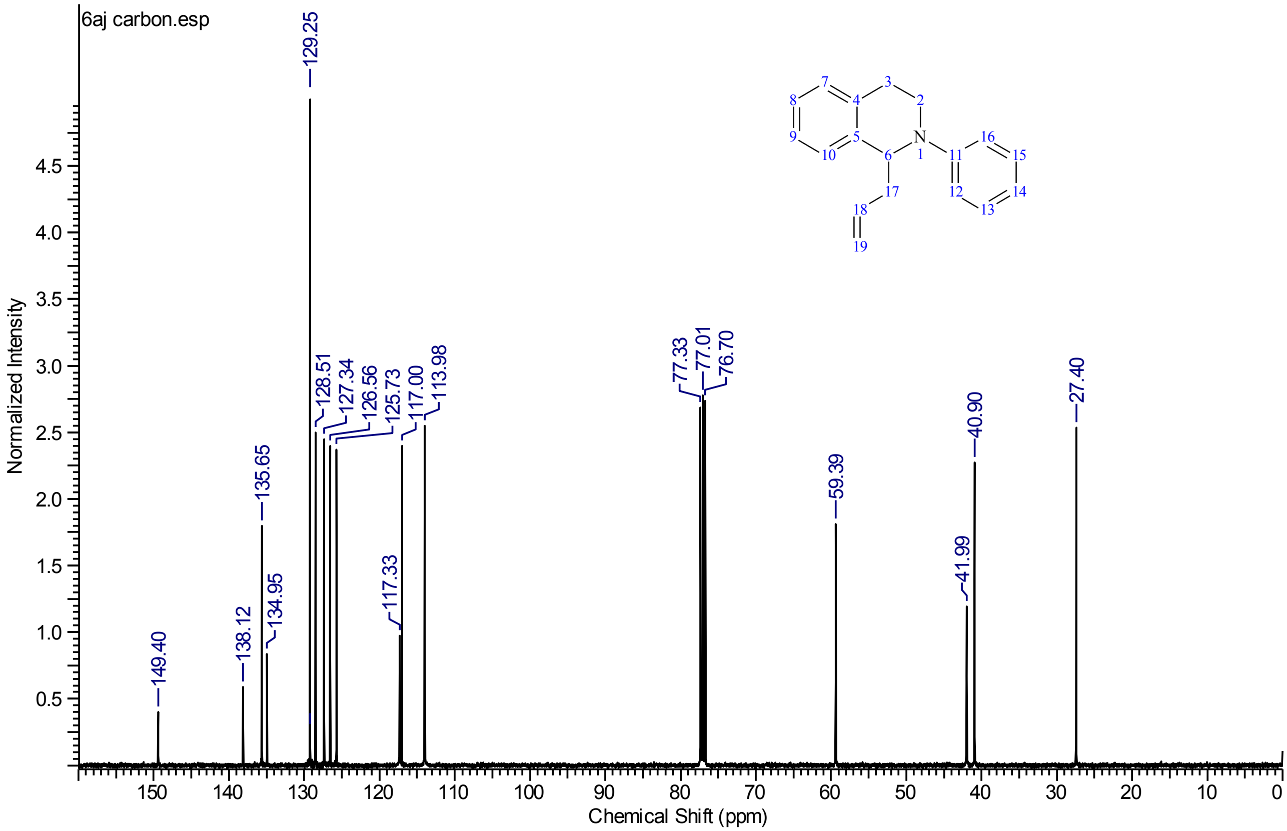

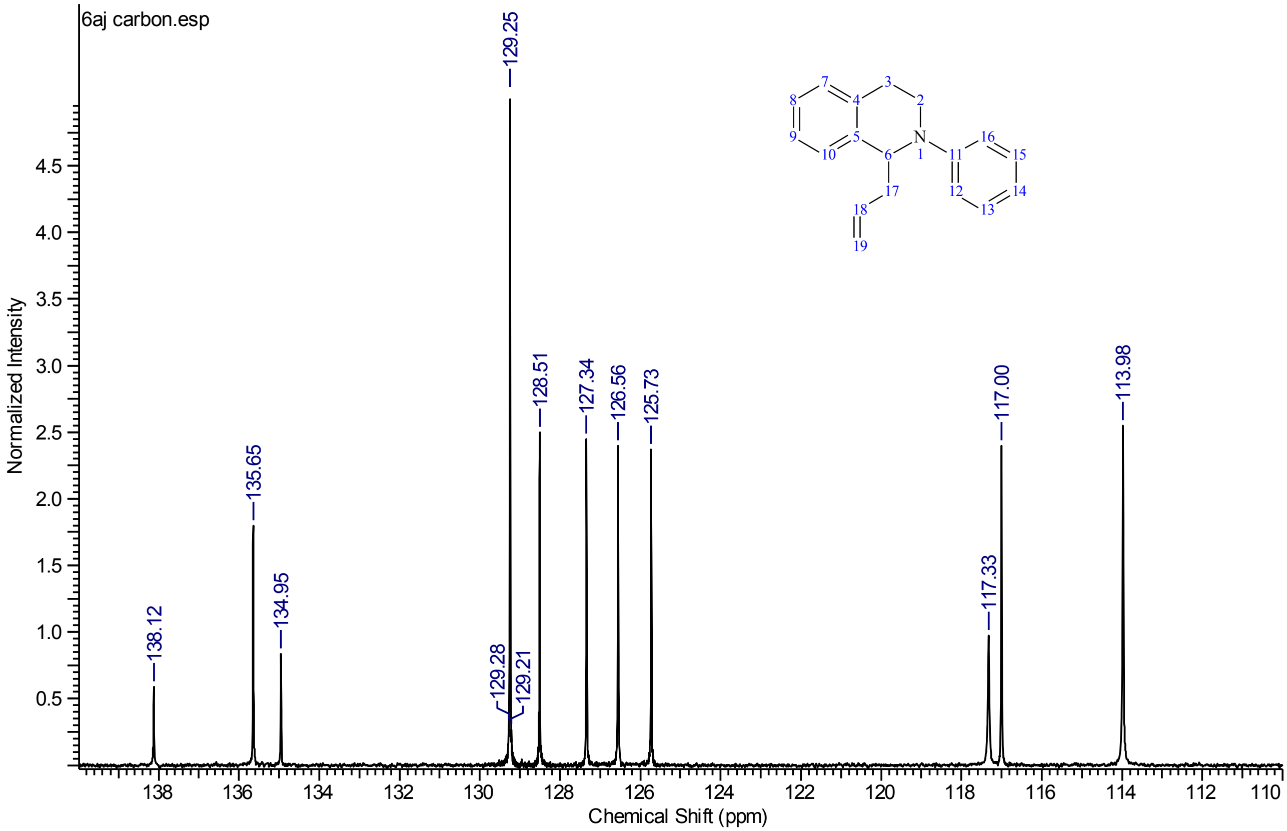

^{1}H NMR – **7a**

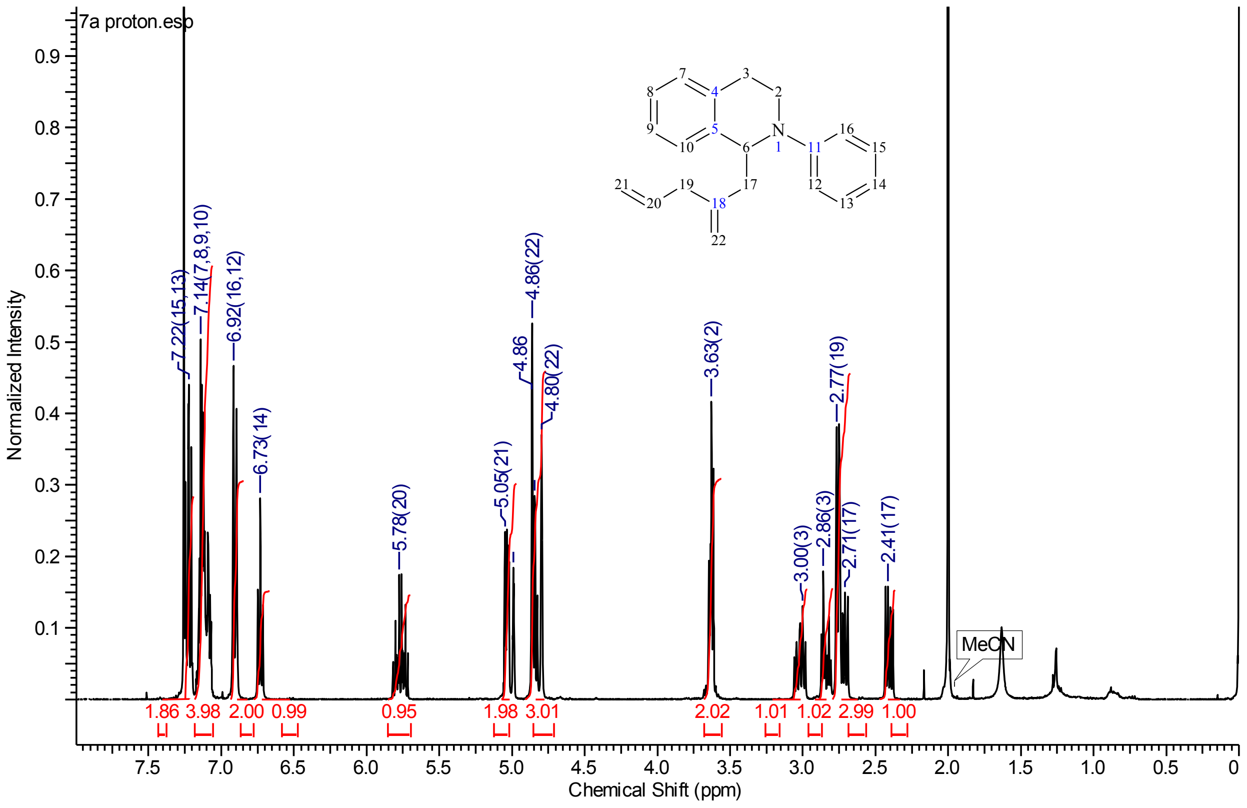
7a proton.esp
Normalized Intensity
Chemical Shift (ppm)
7.22(15,13)
7.14(7,8,9,10)
6.92(16,12)
6.73(14)
5.78(20)
5.05(21)
4.86
4.86(22)
4.80(22)
3.63(2)
3.00(3)
2.86(3)
2.77(19)
2.71(17)
2.41(17)
MeCN
1.86
3.98
2.00
0.99
0.95
1.98
3.01
2.02
1.01
1.02
2.99
1.00

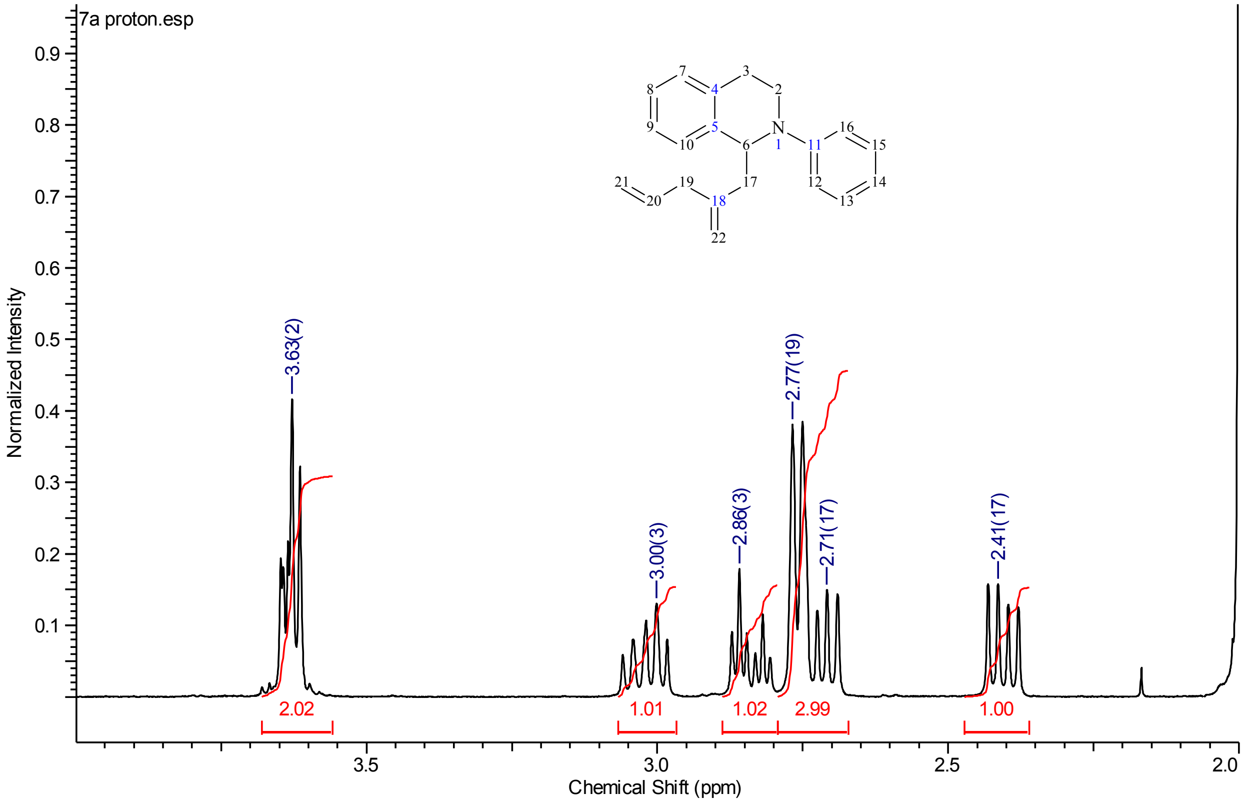
7a proton.esp
Normalized Intensity
Chemical Shift (ppm)
3.63(2)
3.00(3)
2.86(3)
2.77(19)
2.71(17)
2.41(17)
2.02
1.01
1.02
2.99
1.00

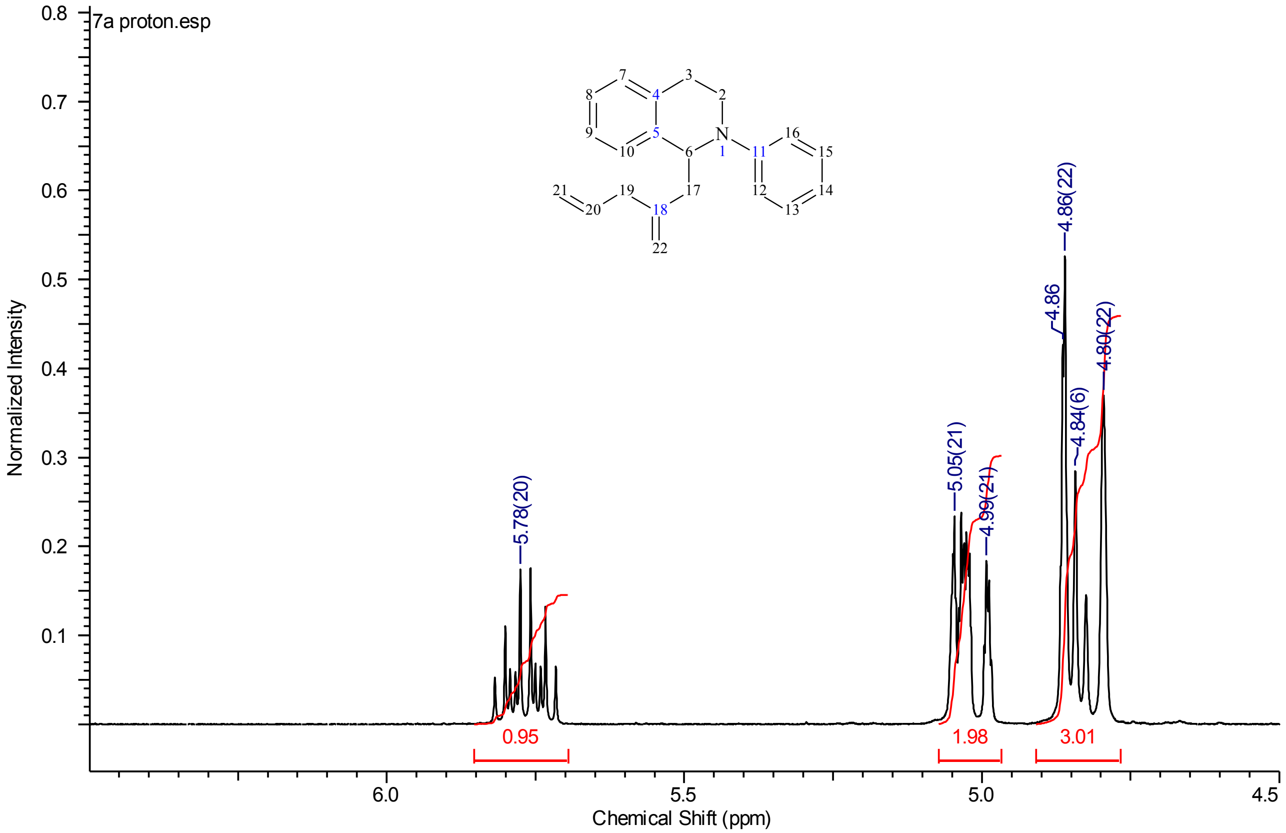
7a proton.esp
Normalized Intensity
Chemical Shift (ppm)
5.78(20)
5.05(21)
4.99(21)
4.86
4.86(22)
4.84(6)
4.80(22)
0.95
1.98
3.01

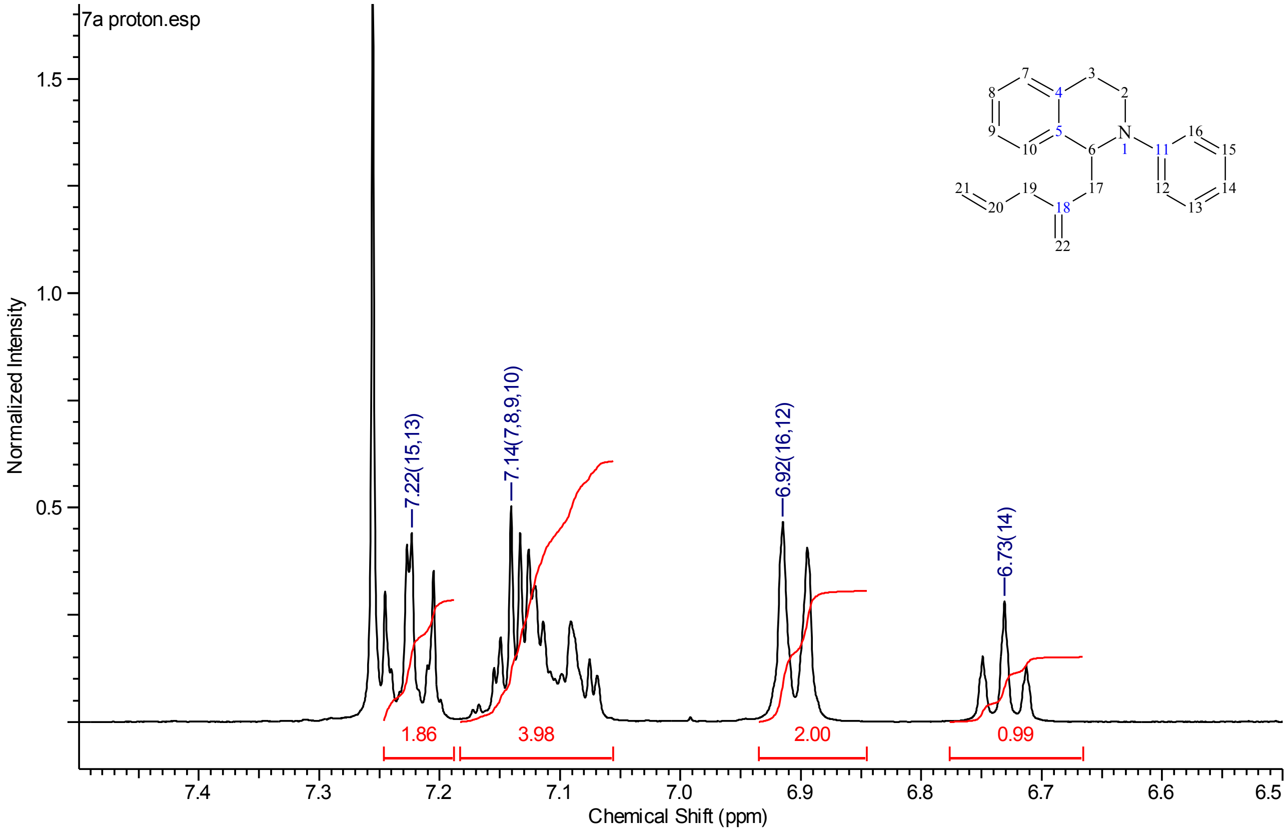
7a proton.esp
Normalized Intensity
Chemical Shift (ppm)
7.22(15,13)
7.14(7,8,9,10)
6.92(16,12)
6.73(14)
1.86
3.98
2.00
0.99

^{13}C NMR – **7a**

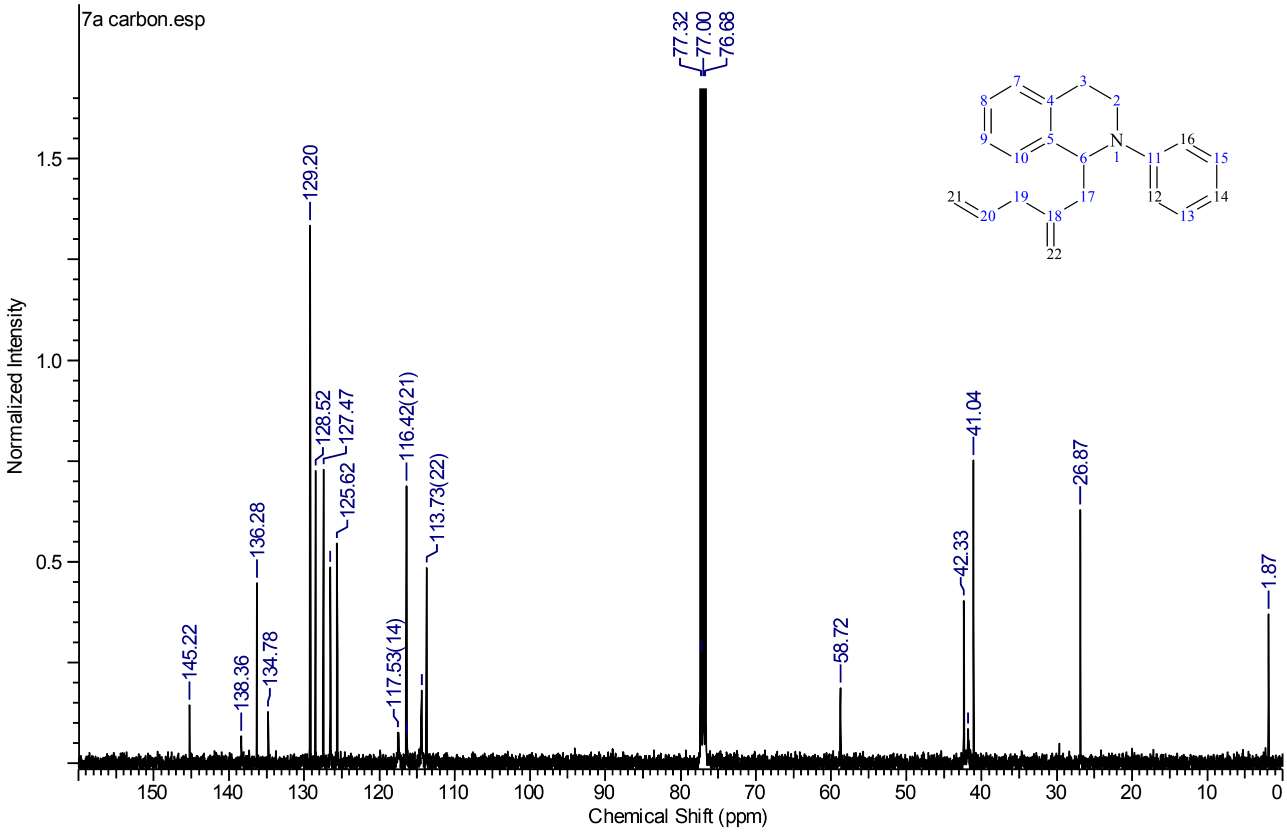
7a carbon.esp
77.32
77.00
76.68
129.20
128.52
127.47
125.62
136.28
145.22
138.36
134.78
117.53(14)
116.42(21)
113.73(22)
58.72
42.33
41.04
26.87
1.87
Normalized Intensity
Chemical Shift (ppm)

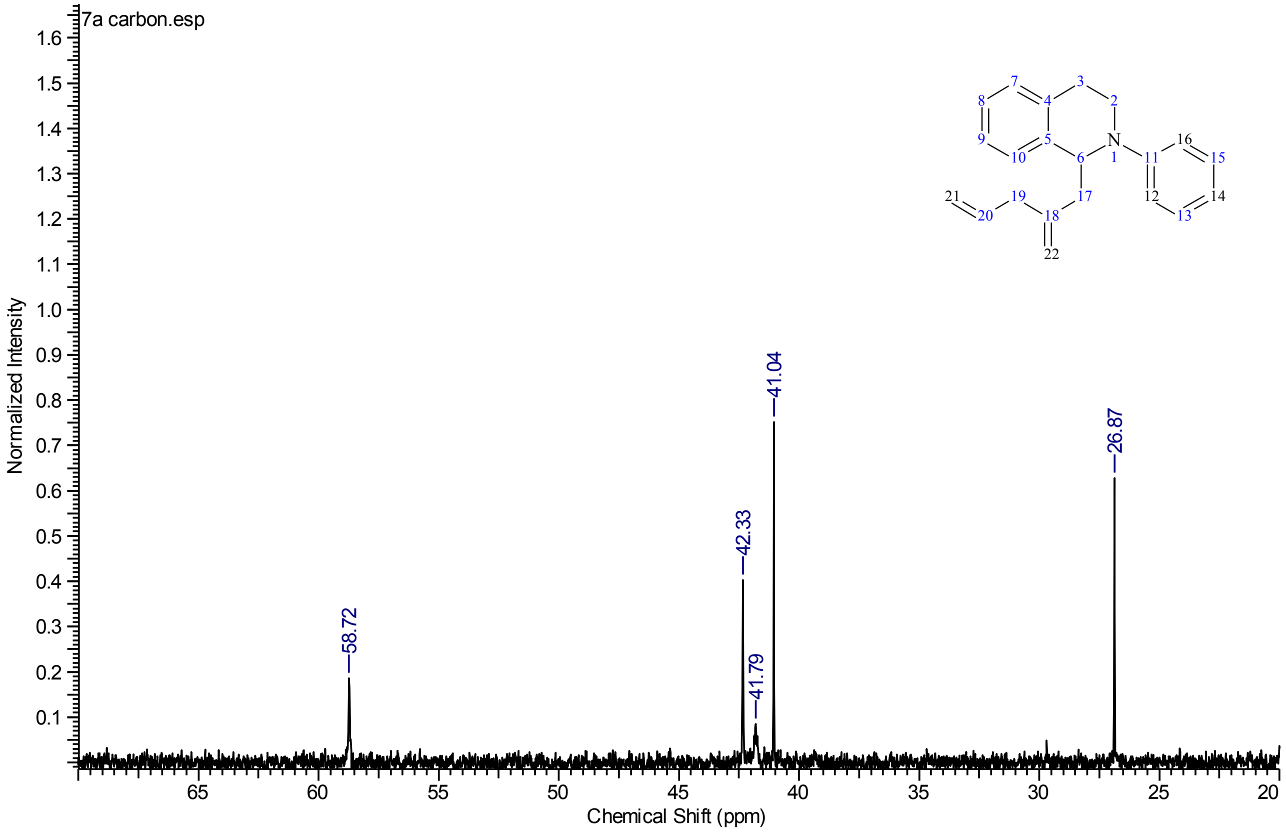
7a carbon.esp
58.72
42.33
41.79
41.04
26.87
Normalized Intensity
Chemical Shift (ppm)

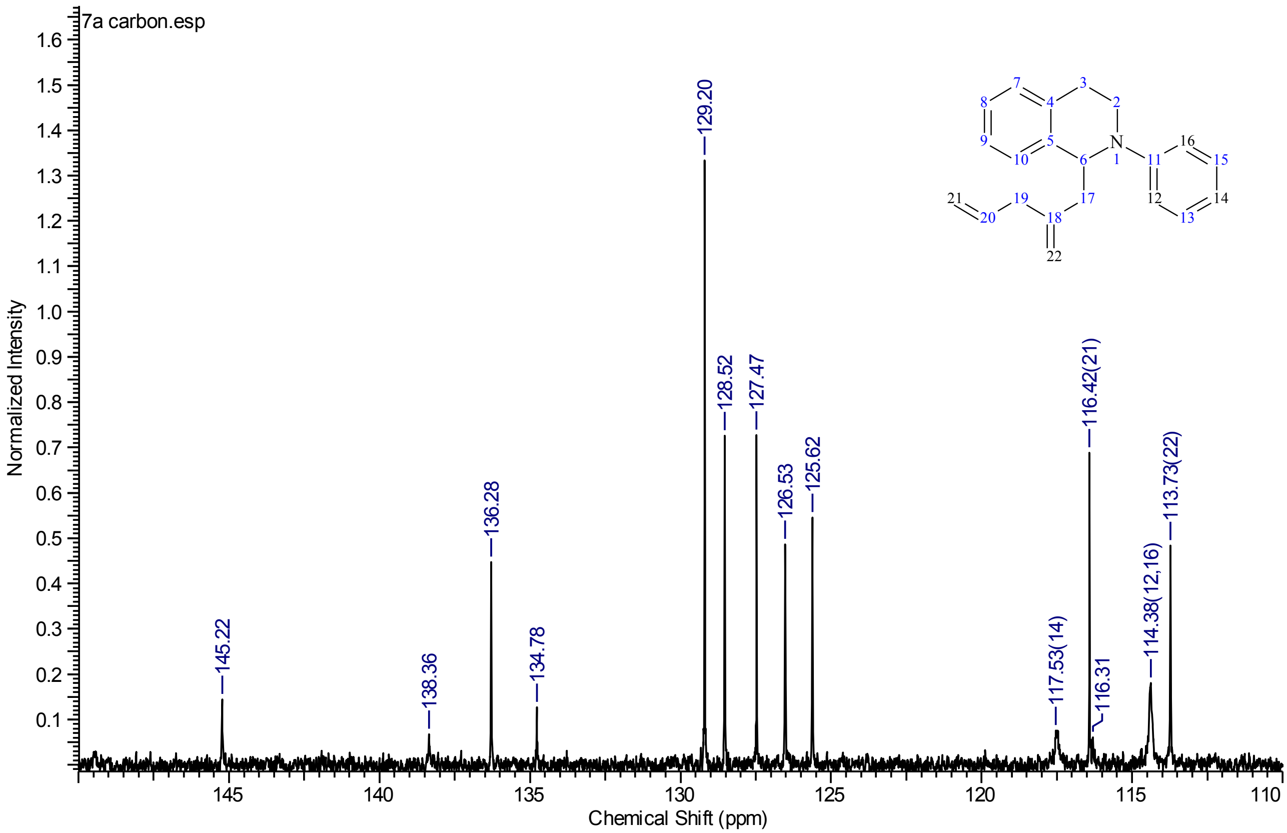

^{13}C DEPT NMR – **7a**

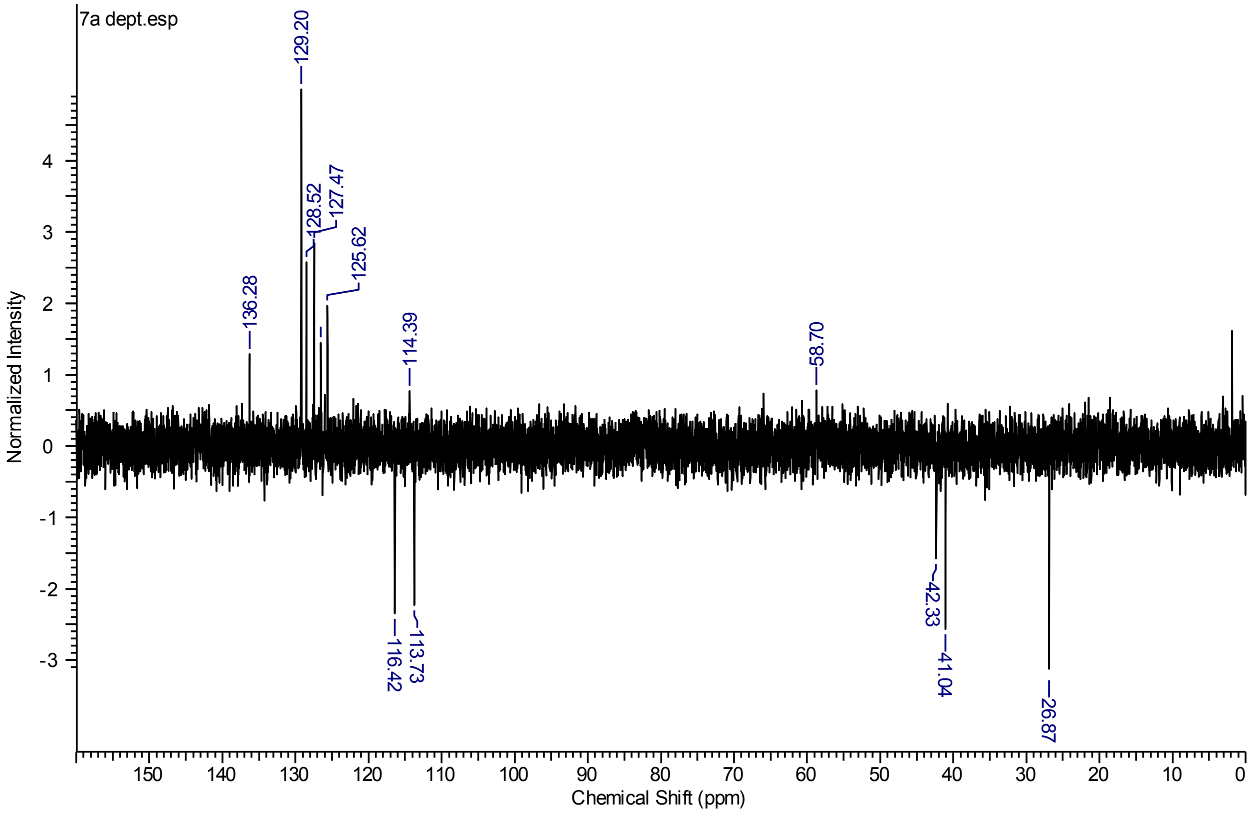

COSY NMR – **7a**

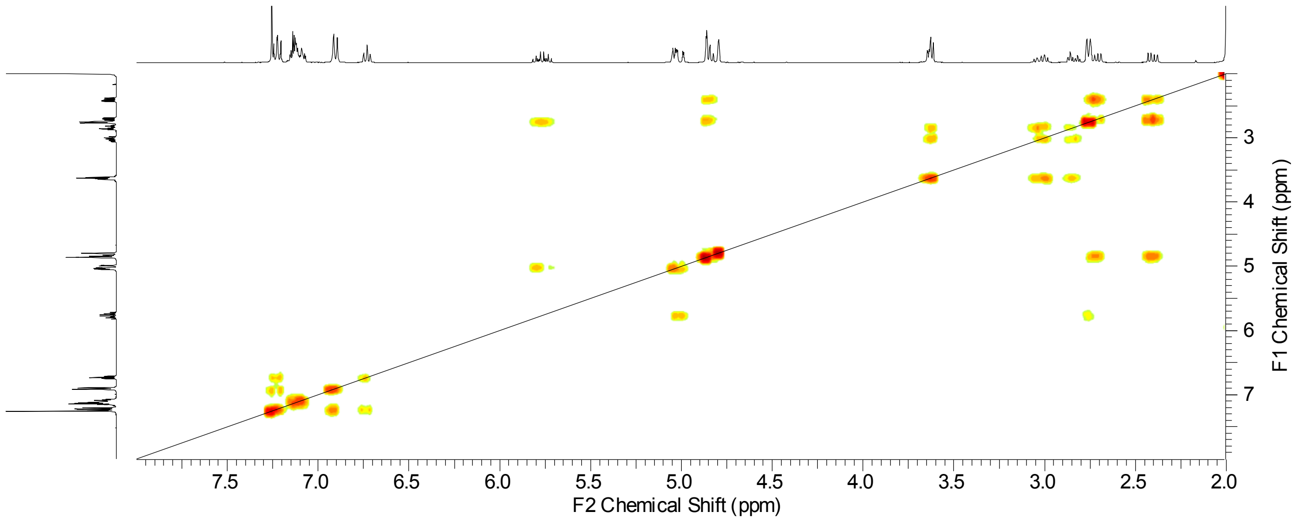

HSQC NMR – **7a**

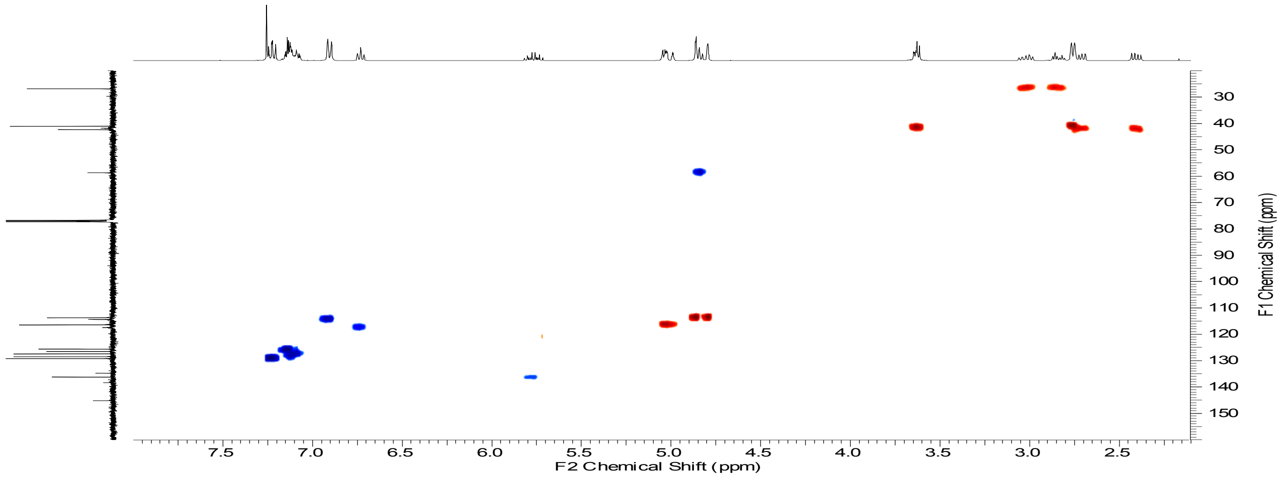

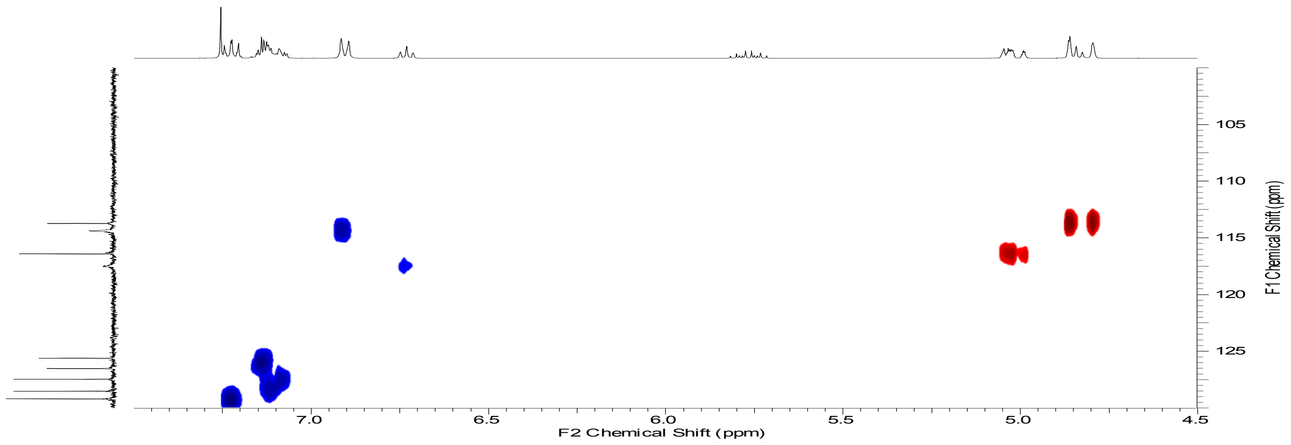

HMBC NMR – **7a**

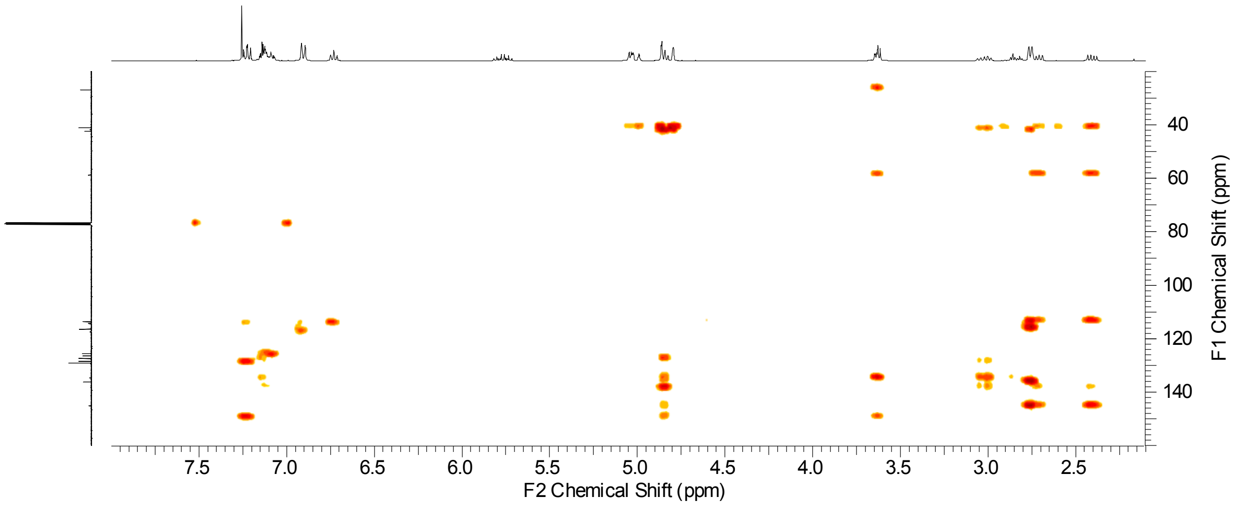

^{1}H NMR – **8a**

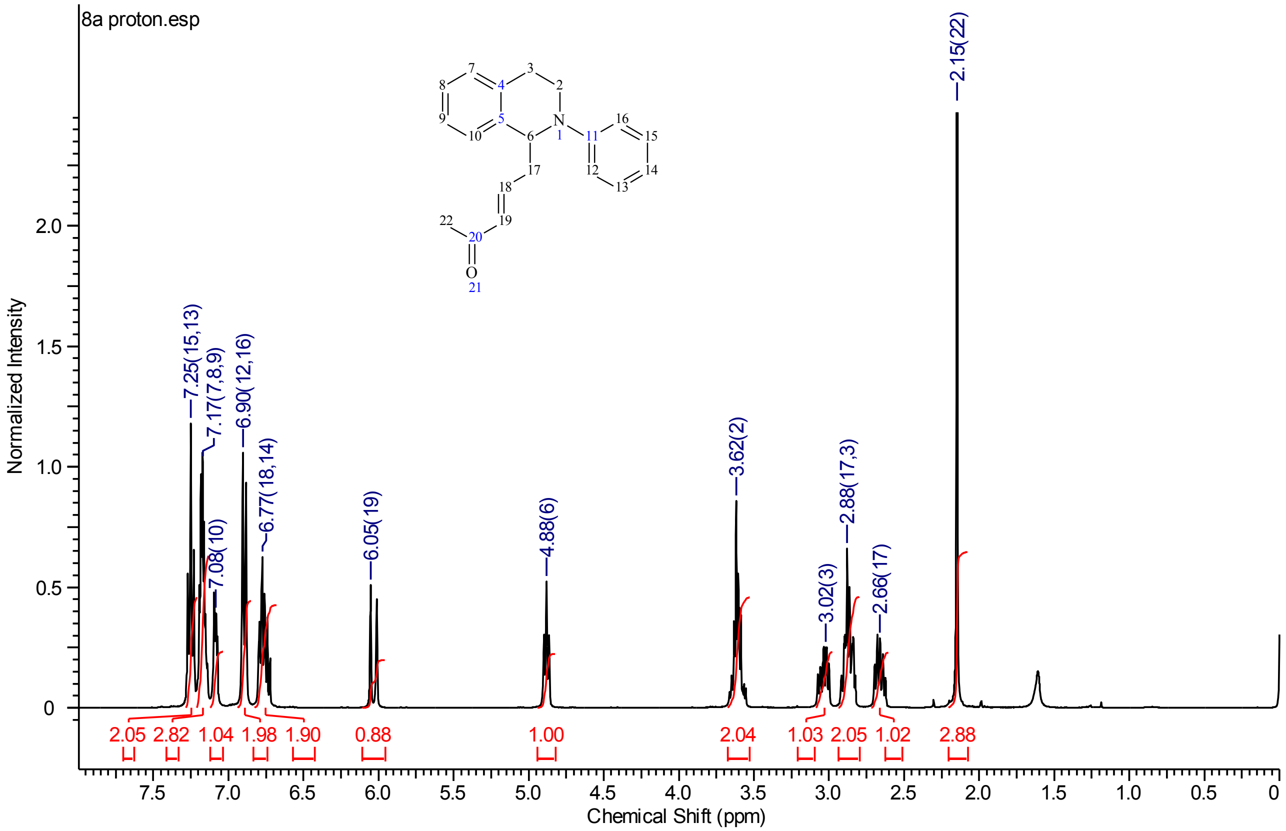

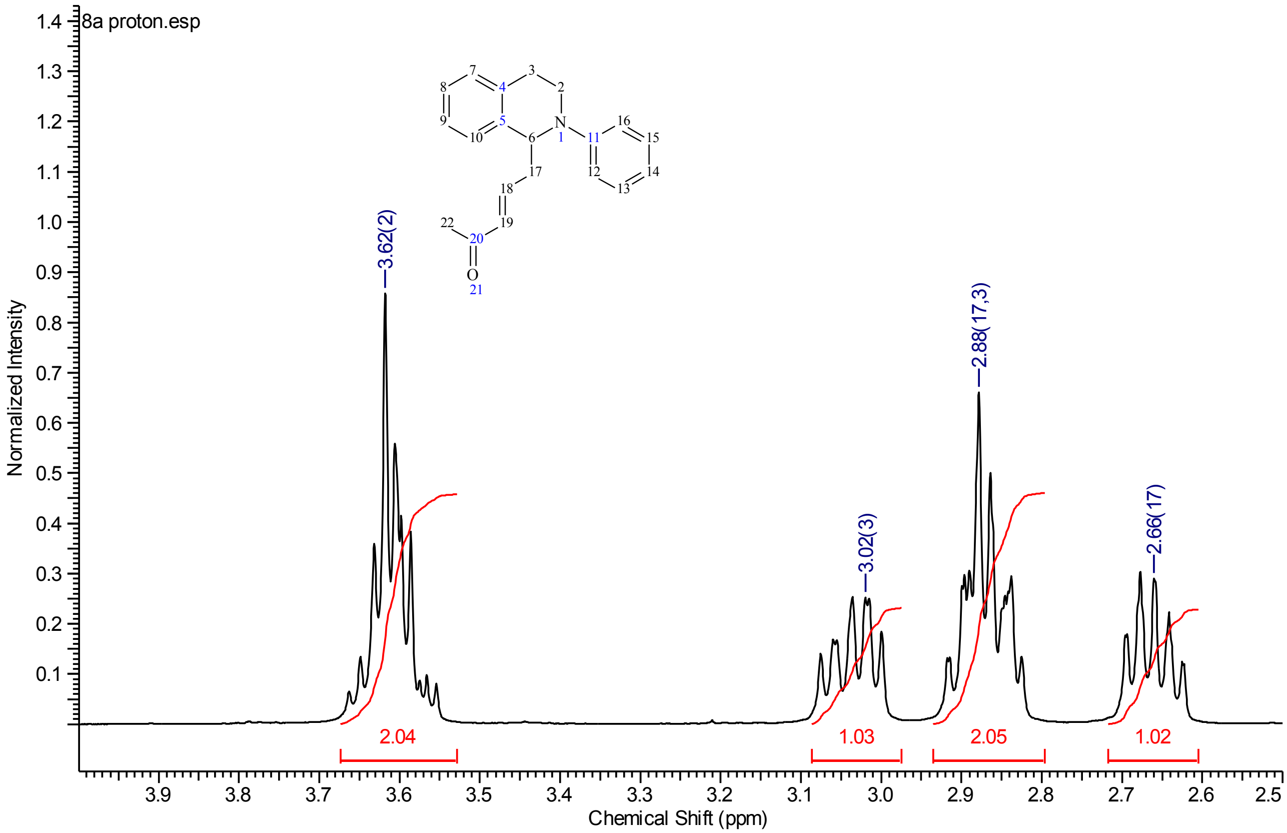
8a proton.esp
3.62(2)
3.02(3)
2.88(17,3)
2.66(17)
2.04
1.03
2.05
1.02
Normalized Intensity
Chemical Shift (ppm)

^{13}C NMR – **8a**

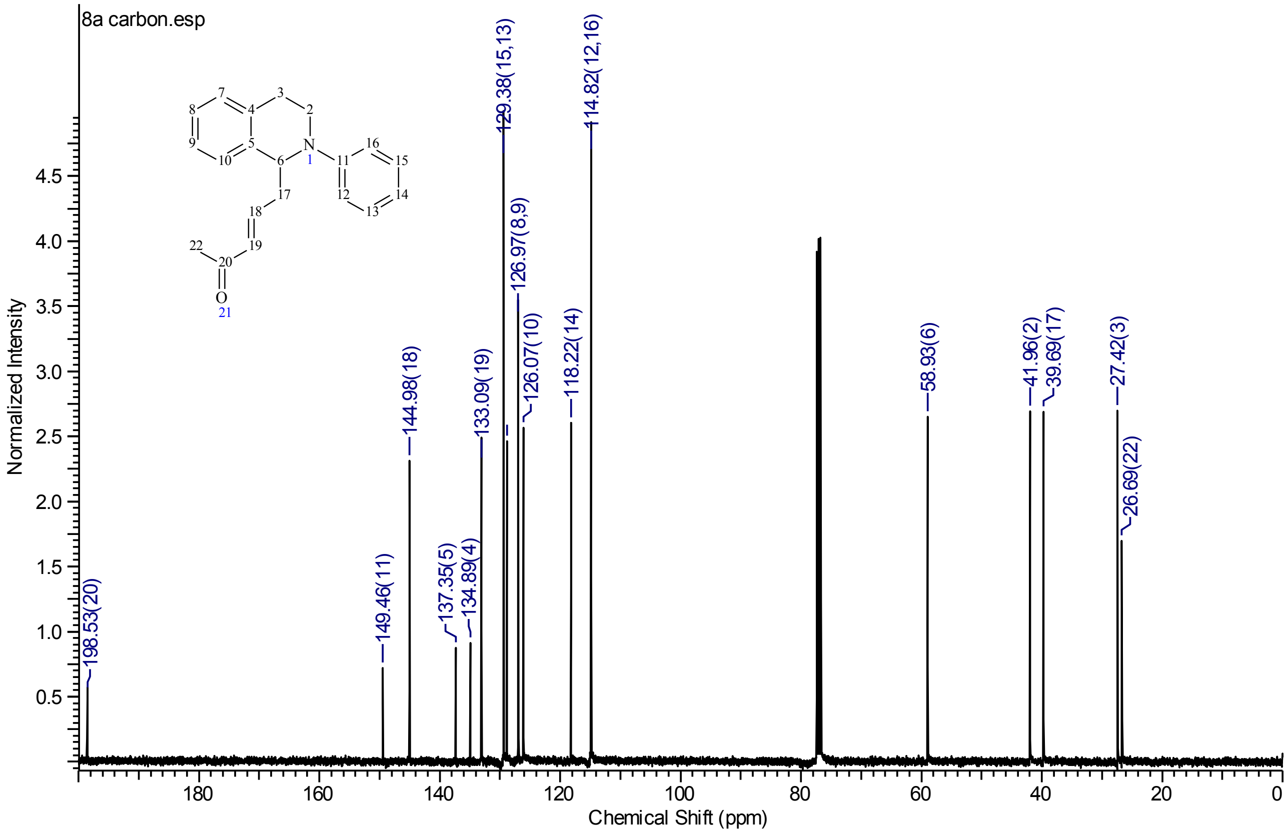
8a carbon.esp
198.53(20)
149.46(11)
144.98(18)
137.35(5)
134.89(4)
133.09(19)
129.38(15,13)
126.97(8,9)
126.07(10)
118.22(14)
114.82(12,16)
58.93(6)
41.96(2)
39.69(17)
27.42(3)
26.69(22)
Normalized Intensity
Chemical Shift (ppm)

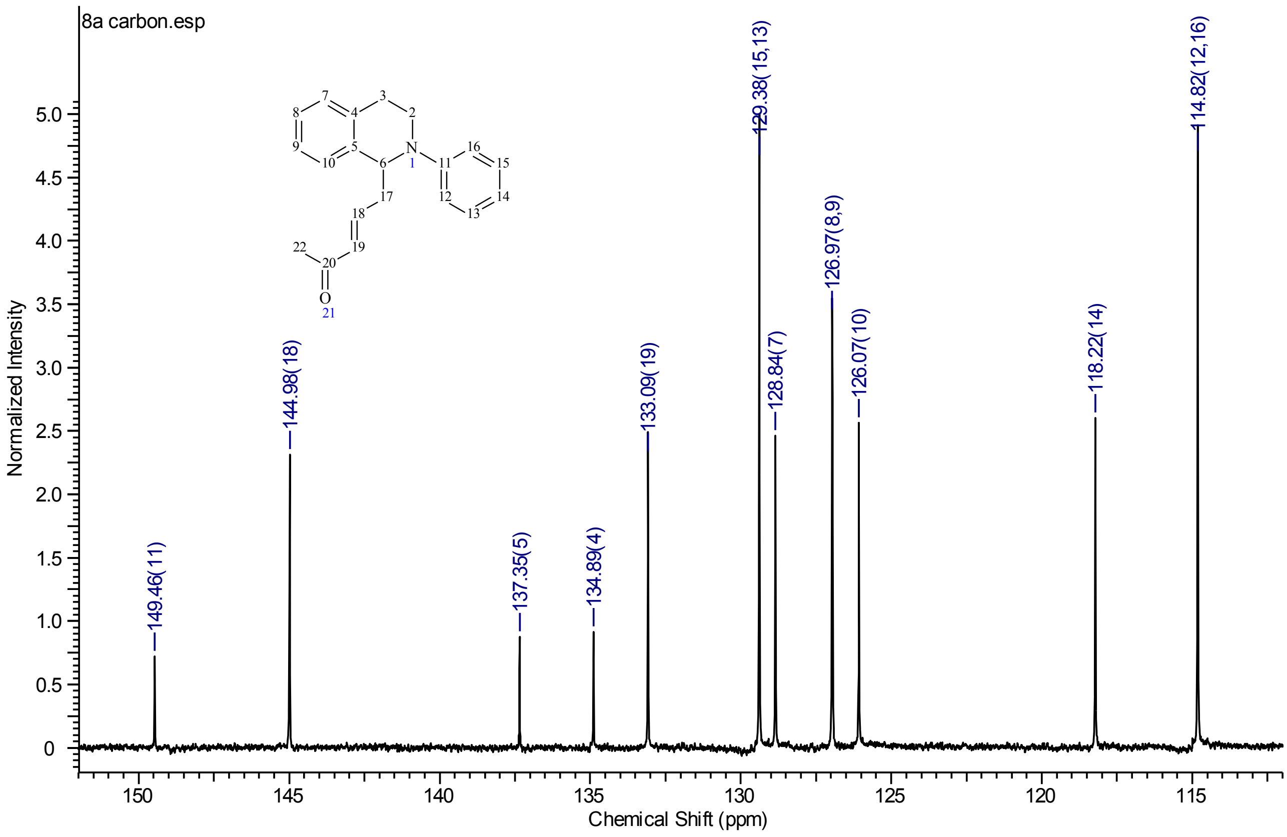
8a carbon.esp
Normalized Intensity
Chemical Shift (ppm)
149.46(11)
144.98(18)
137.35(5)
134.89(4)
133.09(19)
129.38(15,13)
128.84(7)
126.97(8,9)
126.07(10)
118.22(14)
114.82(12,16)

^{1}H NMR – **8b**

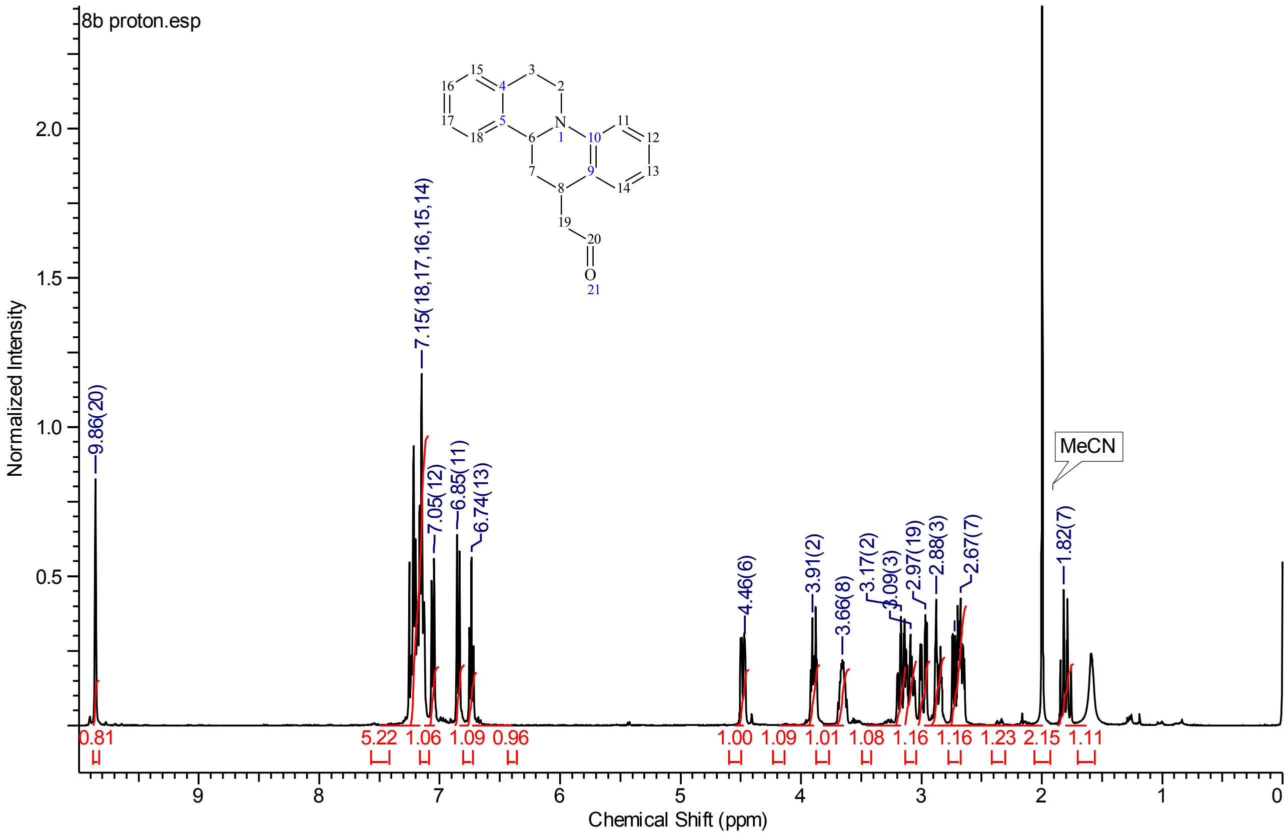
8b proton.esp
Normalized Intensity
Chemical Shift (ppm)
9.86(20)
7.15(18,17,16,15,14)
7.05(12)
6.85(11)
6.74(13)
4.46(6)
3.91(2)
3.66(8)
3.17(2)
3.09(3)
2.97(19)
2.88(3)
2.67(7)
1.82(7)
MeCN
0.81
5.22
1.06
1.09
0.96
1.00
1.09
1.01
1.08
1.16
1.16
1.23
2.15
1.11

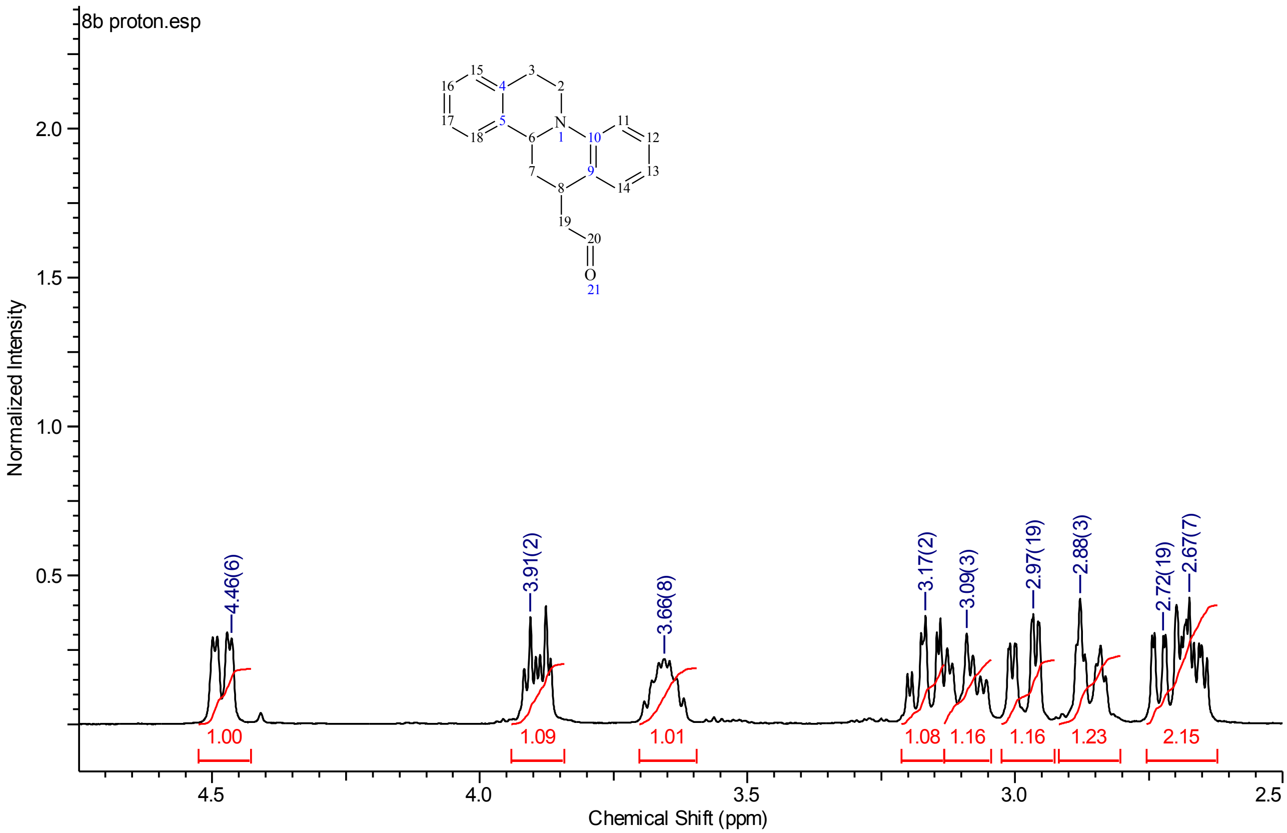
8b proton.esp
Normalized Intensity
Chemical Shift (ppm)
4.46(6)
3.91(2)
3.66(8)
3.17(2)
3.09(3)
2.97(19)
2.88(3)
2.72(19)
2.67(7)
1.00
1.09
1.01
1.08
1.16
1.16
1.23
2.15

^{13}C NMR – **8b**

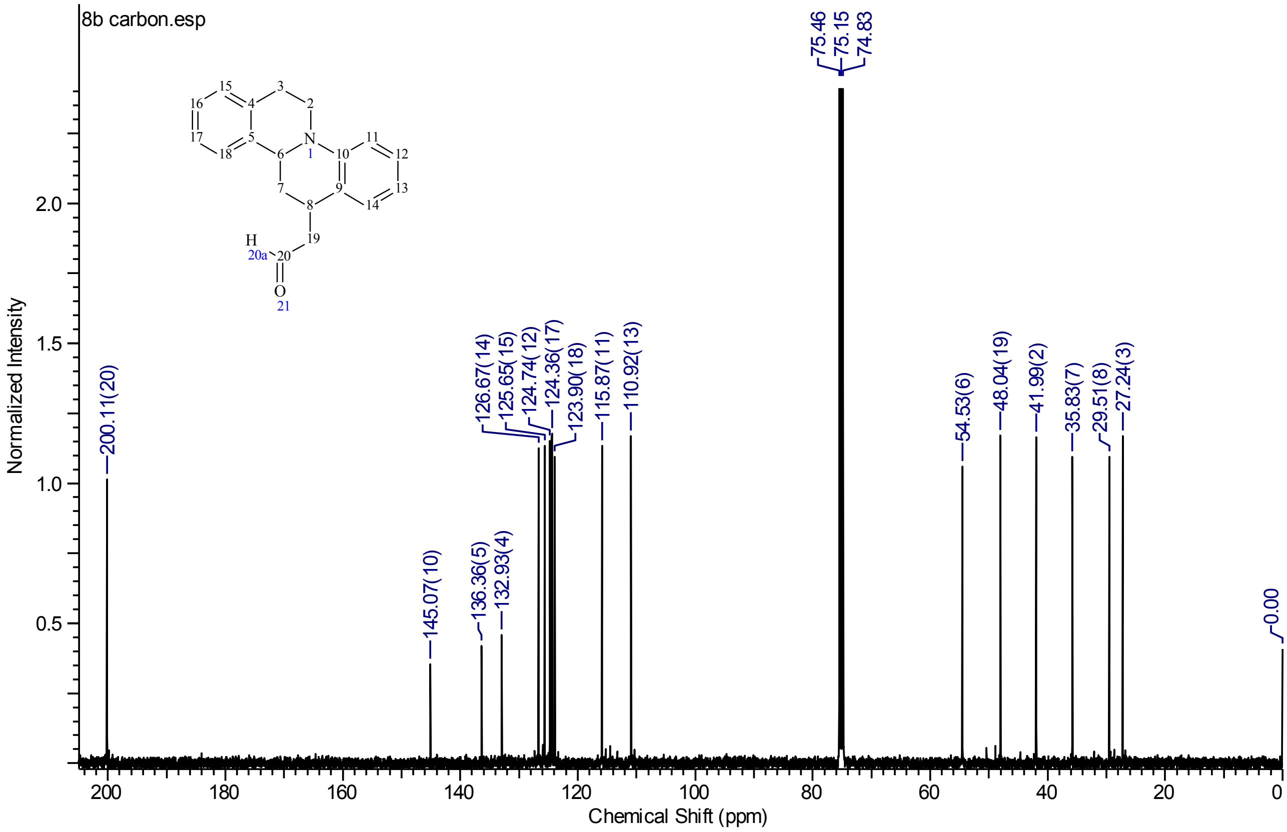
8b carbon.esp
Normalized Intensity
Chemical Shift (ppm)
200.11(20)
145.07(10)
136.36(5)
132.93(4)
126.67(14)
125.65(15)
124.74(12)
124.36(17)
123.90(18)
115.87(11)
110.92(13)
75.46
75.15
74.83
54.53(6)
48.04(19)
41.99(2)
35.83(7)
29.51(8)
27.24(3)
0.00

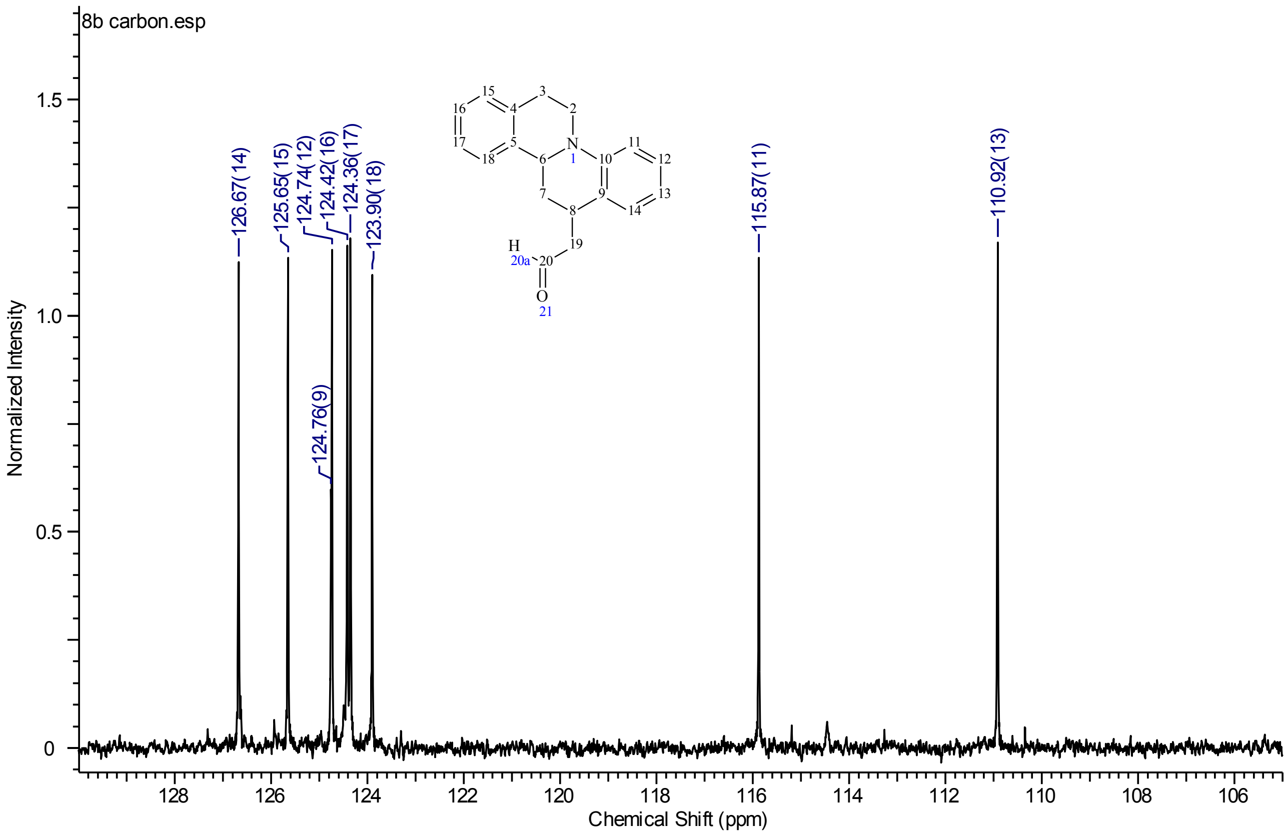
8b carbon.esp
126.67(14)
125.65(15)
124.74(12)
124.42(16)
124.36(17)
123.90(18)
124.76(9)
115.87(11)
110.92(13)
Normalized Intensity
Chemical Shift (ppm)

^{1}H NMR – **6ba**

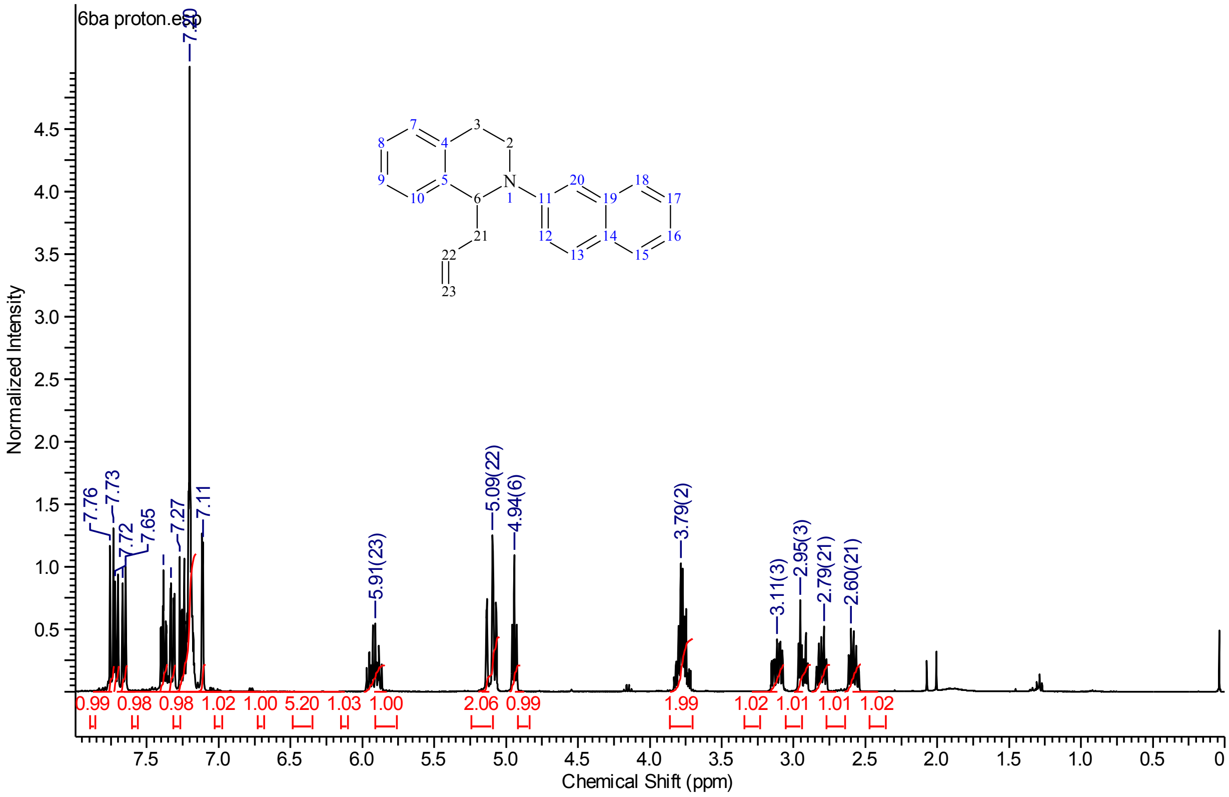
6ba proton.esp
7.20
7.76
7.73
7.72
7.65
7.27
7.11
5.91(23)
5.09(22)
4.94(6)
3.79(2)
3.11(3)
2.95(3)
2.79(21)
2.60(21)
0.99 0.98 0.98 1.02 1.00 5.20 1.03 1.00 2.06 0.99 1.99 1.02 1.01 1.01 1.02
Normalized Intensity
Chemical Shift (ppm)

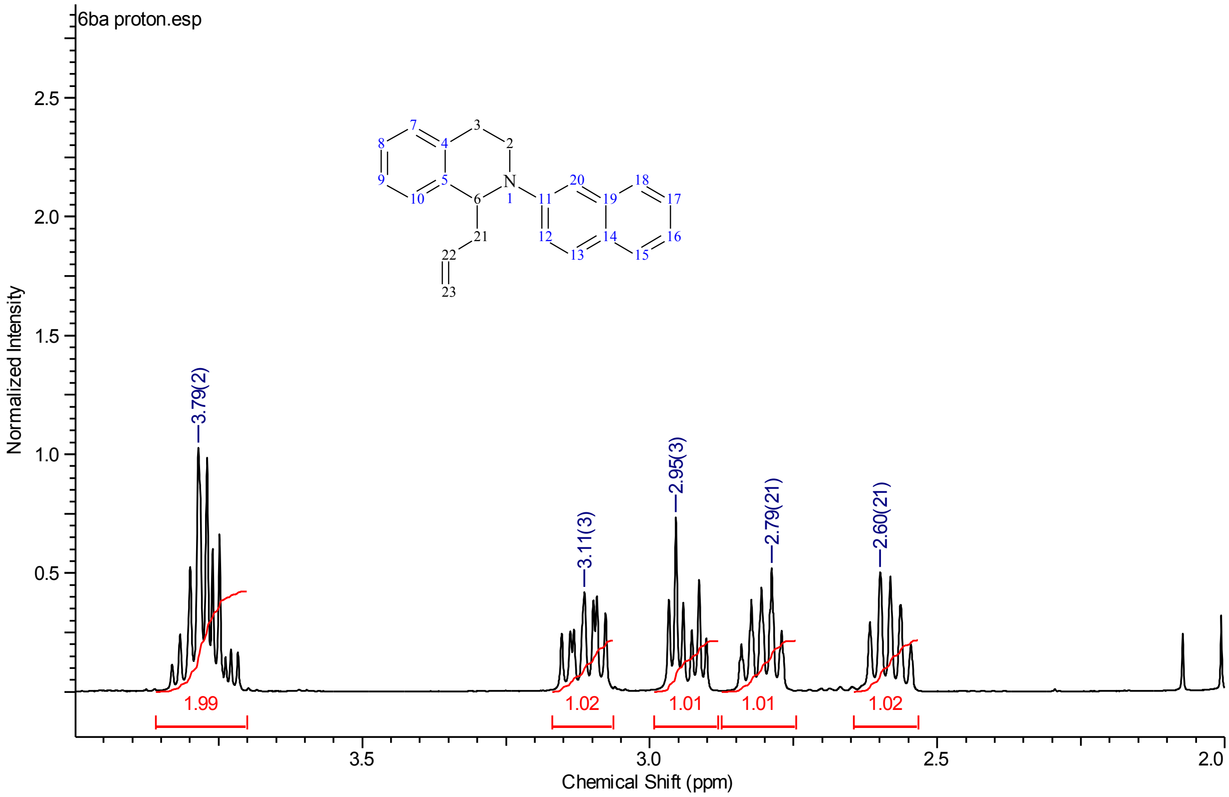
6ba proton.esp
Normalized Intensity
Chemical Shift (ppm)
3.79(2)
3.11(3)
2.95(3)
2.79(21)
2.60(21)
1.99
1.02
1.01
1.01
1.02

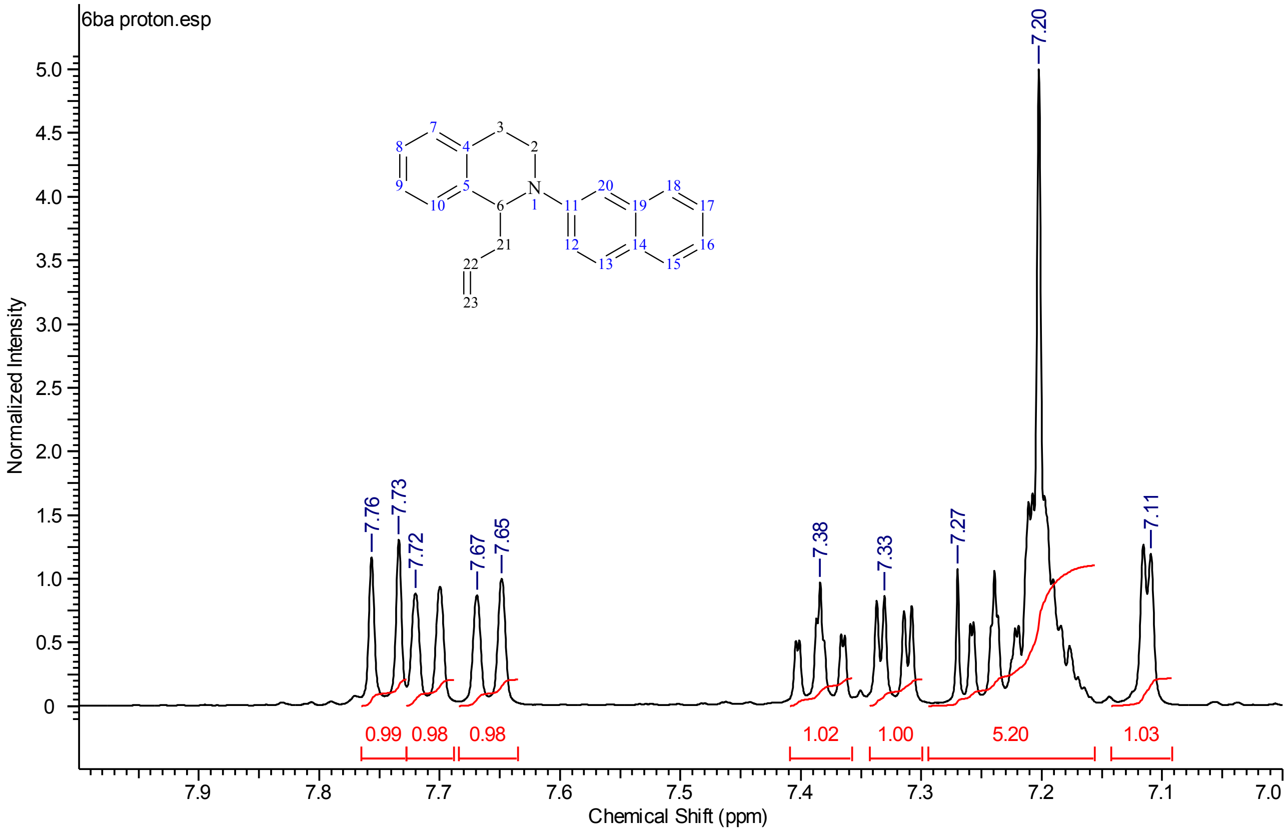
6ba proton.esp
Normalized Intensity
Chemical Shift (ppm)
7.76
7.73
7.72
7.67
7.65
7.38
7.33
7.27
7.20
7.11
0.99
0.98
0.98
1.02
1.00
5.20
1.03

^{13}C NMR – **6ba**

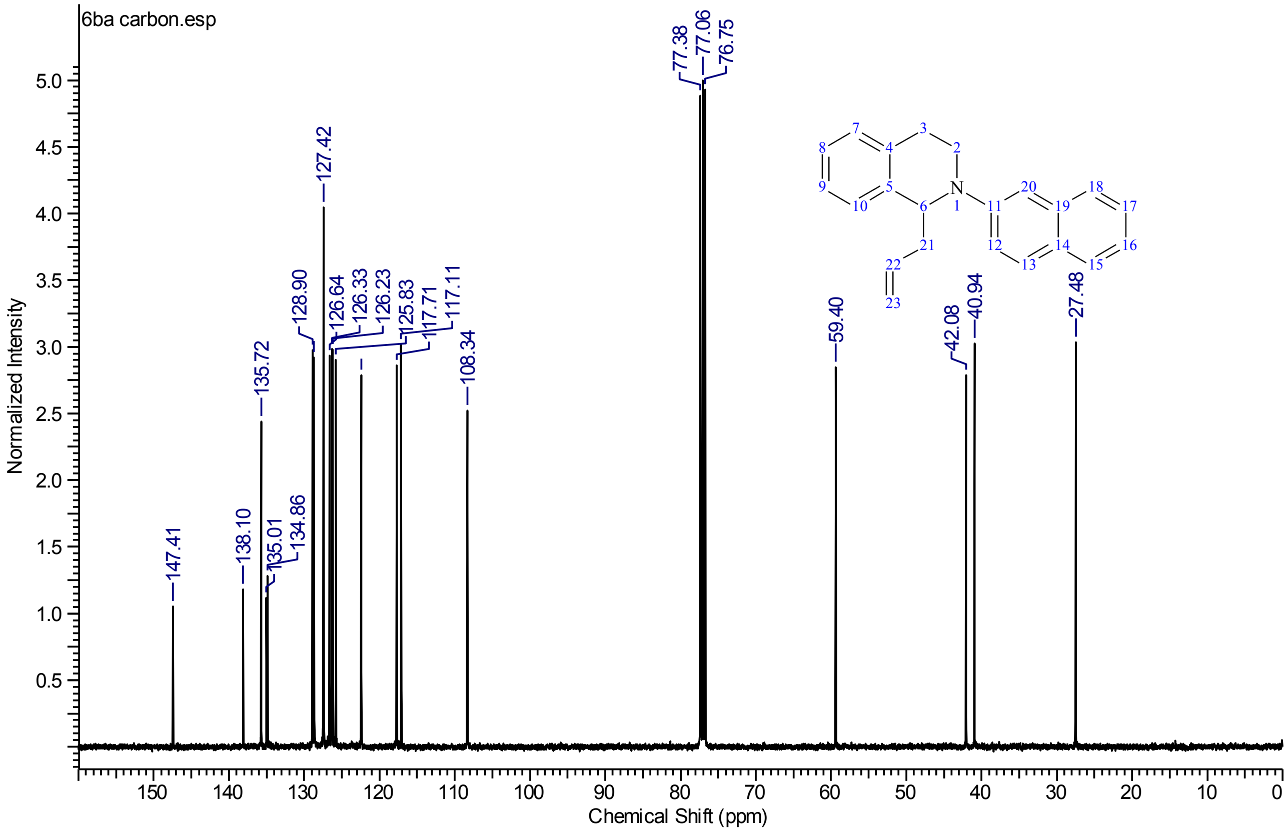

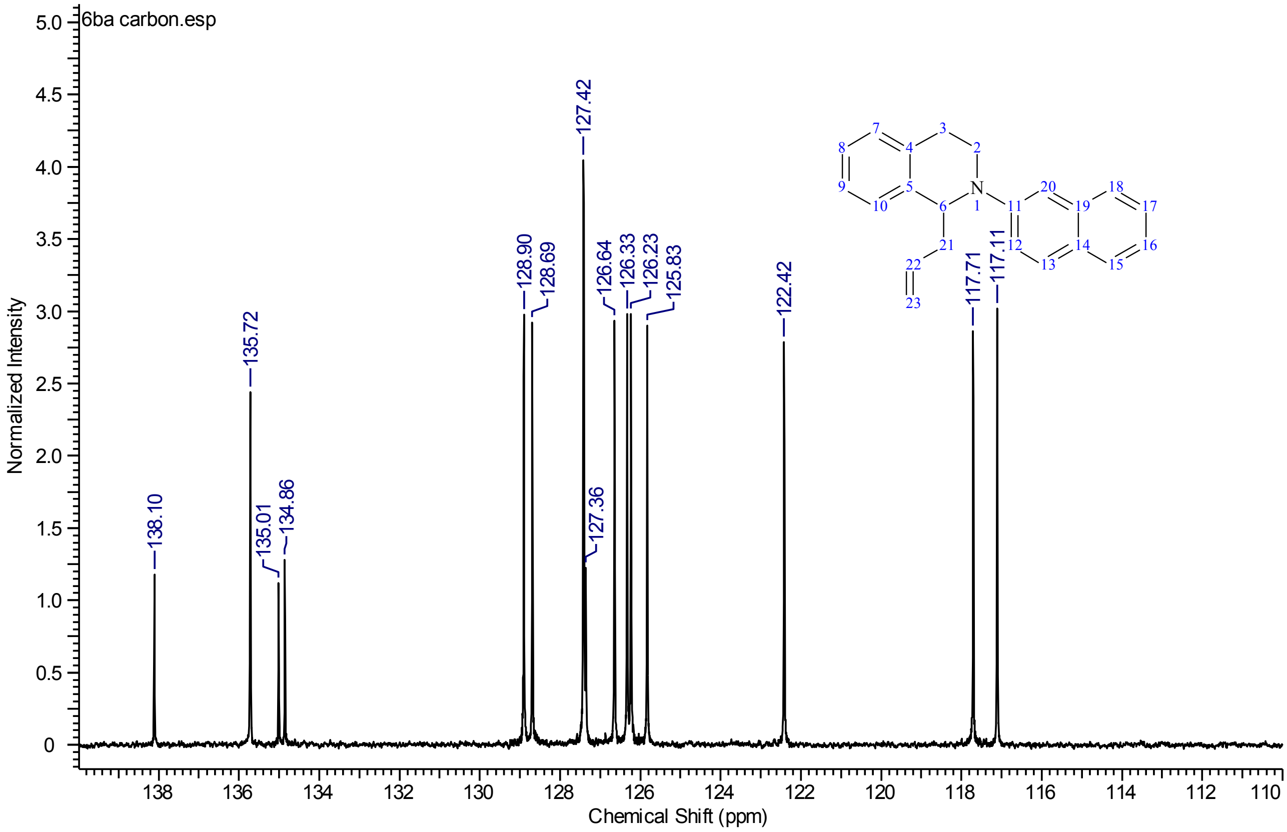

^{1}H NMR – **7b**

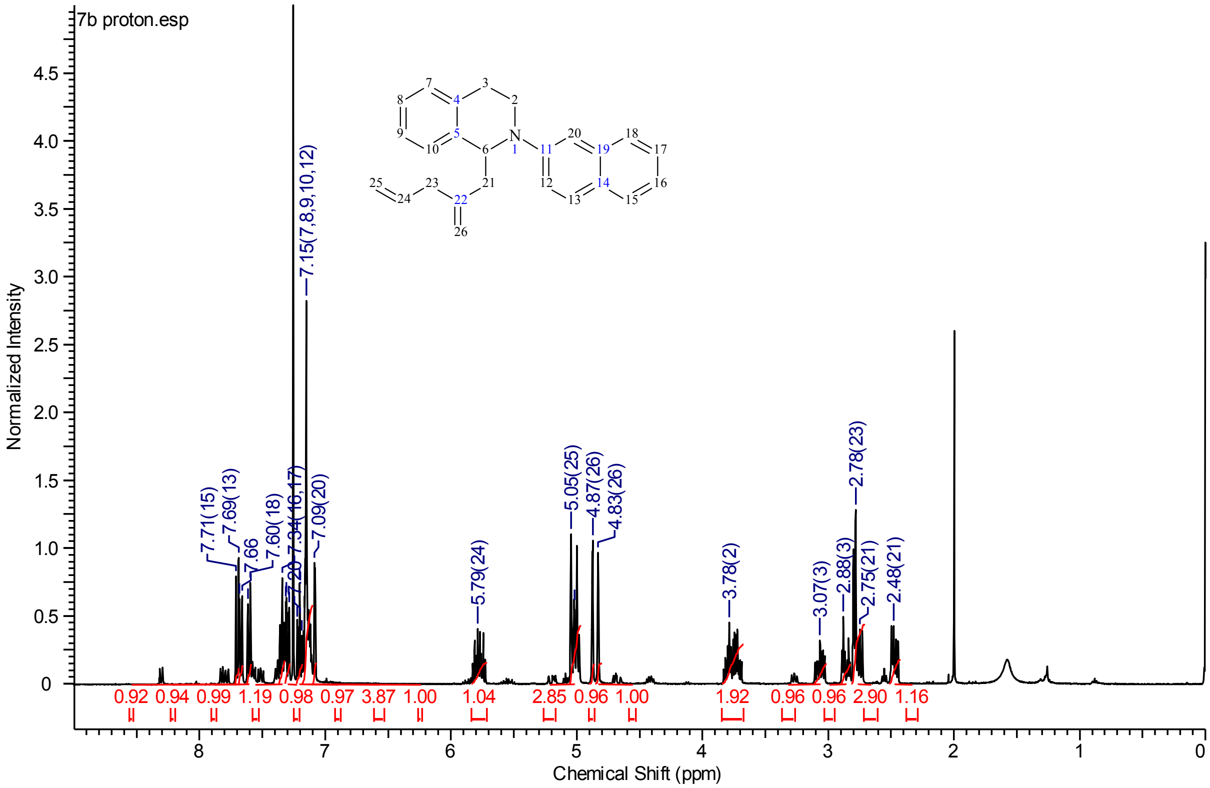

Impurities present. Sufficient purity to identify **7b** as major component by comparison with **7a** ^{1}H NMR

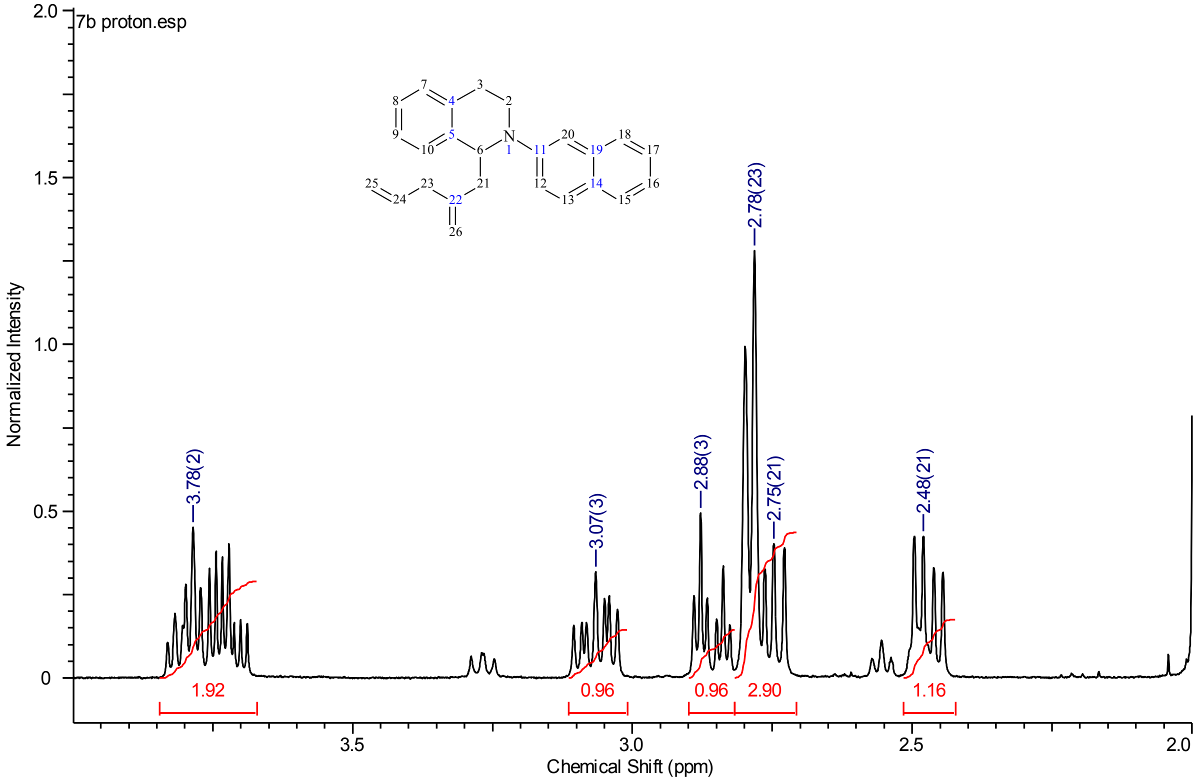

7b signals between 2.0 and 4.0 ppm correspond with **7a** signals between 2.0 and 4.0 ppm.

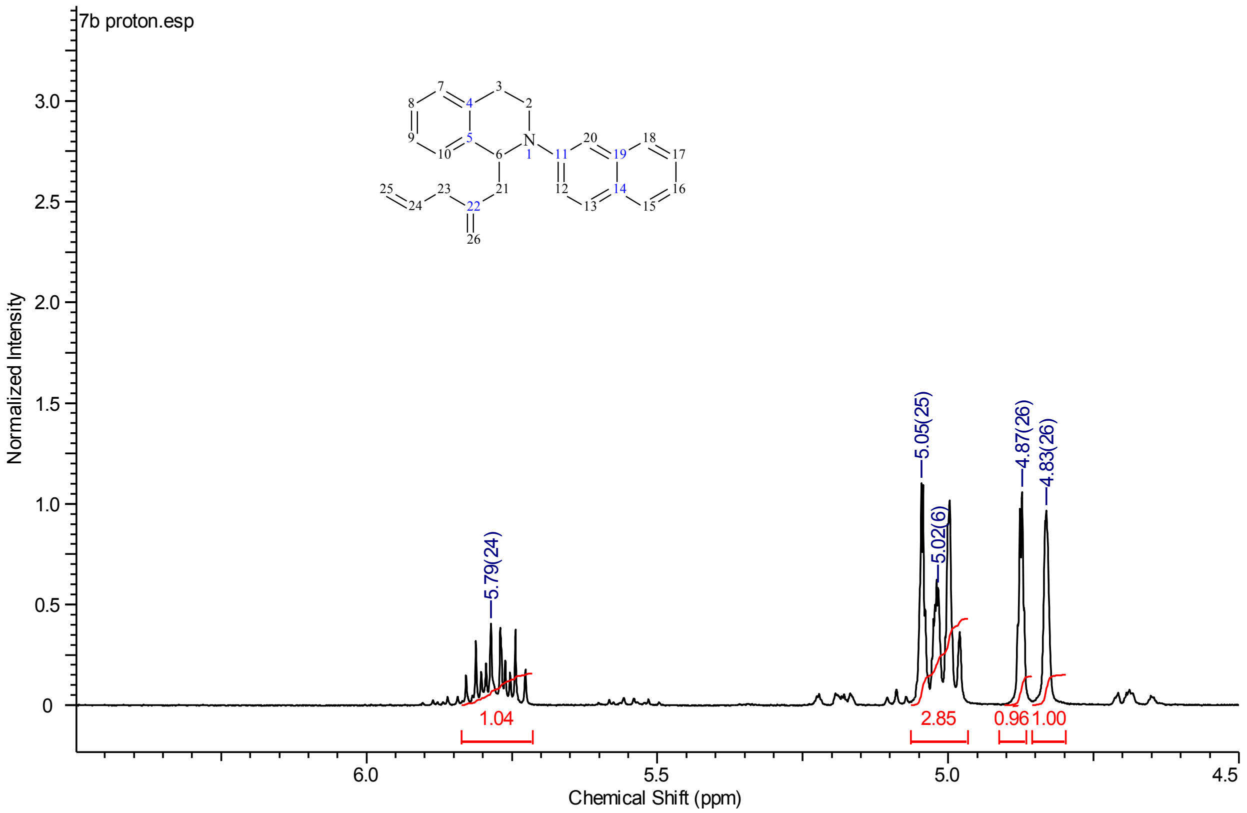

7b signals between 4.5 and 6.0 ppm correspond with **7a** signals between 4.5 and 6.0 ppm.

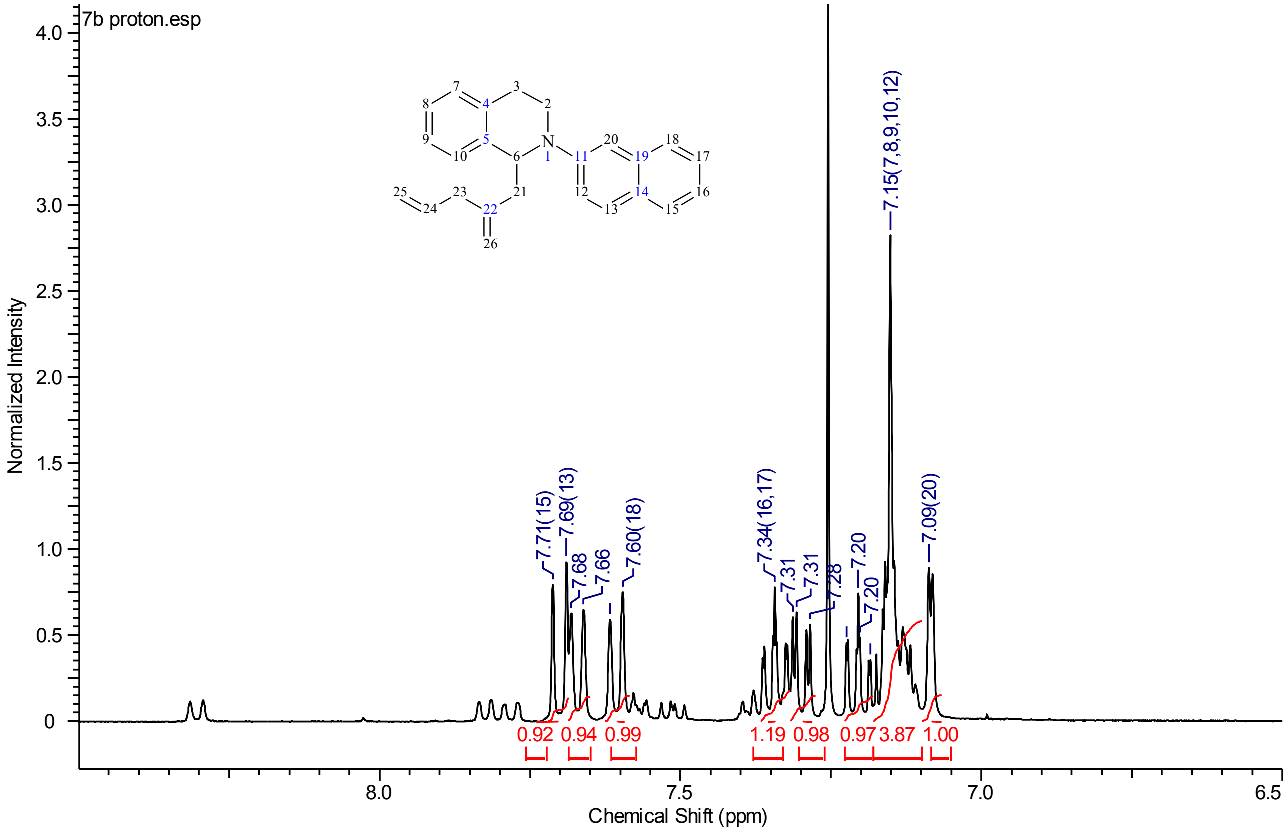

^{1}H NMR – **9**

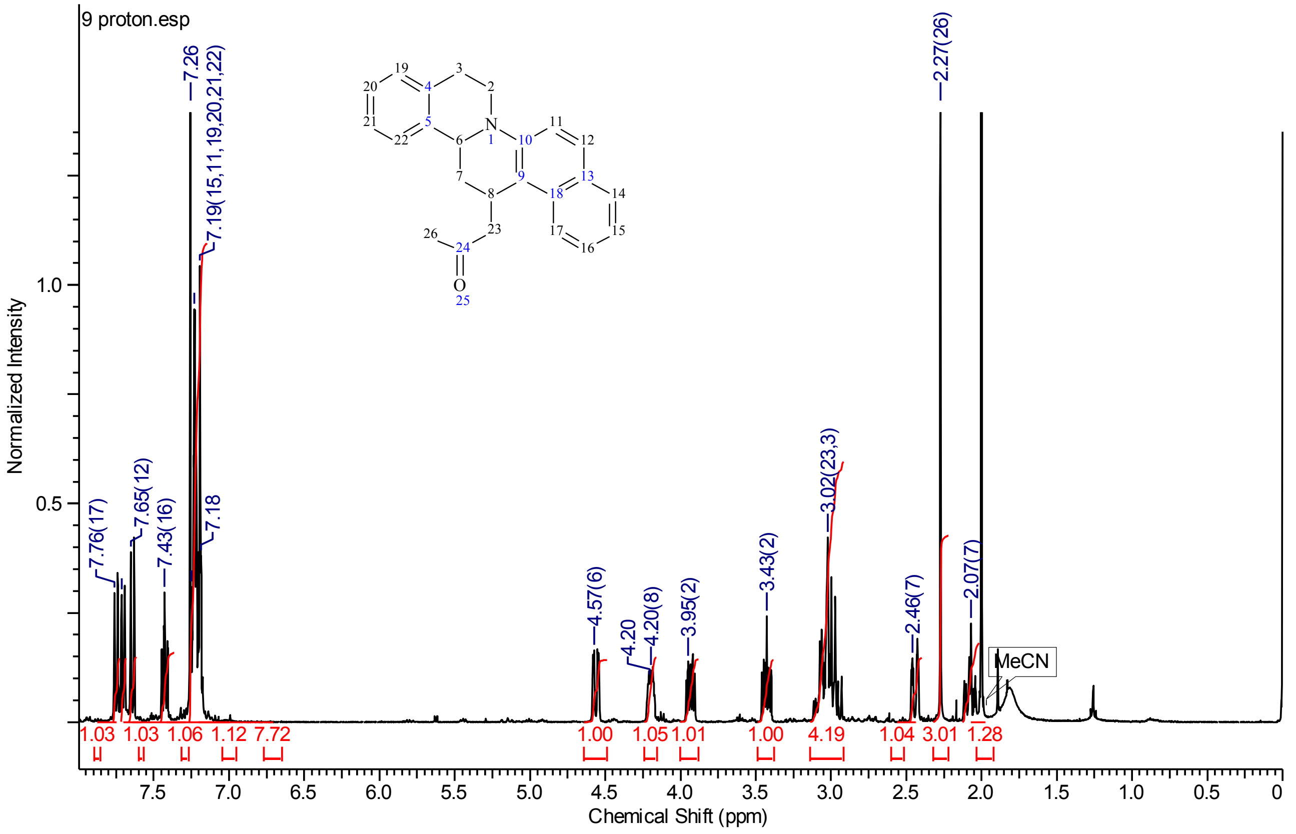
9 proton.esp
7.76(17)
7.65(12)
7.43(16)
7.26
7.19(15,11,19,20,21,22)
7.18
4.57(6)
4.20
4.20(8)
3.95(2)
3.43(2)
3.02(23,3)
2.46(7)
2.27(26)
2.07(7)
MeCN
1.03 1.03 1.06 1.12 7.72 1.00 1.05 1.01 1.00 4.19 1.04 3.01 1.28
Normalized Intensity
Chemical Shift (ppm)

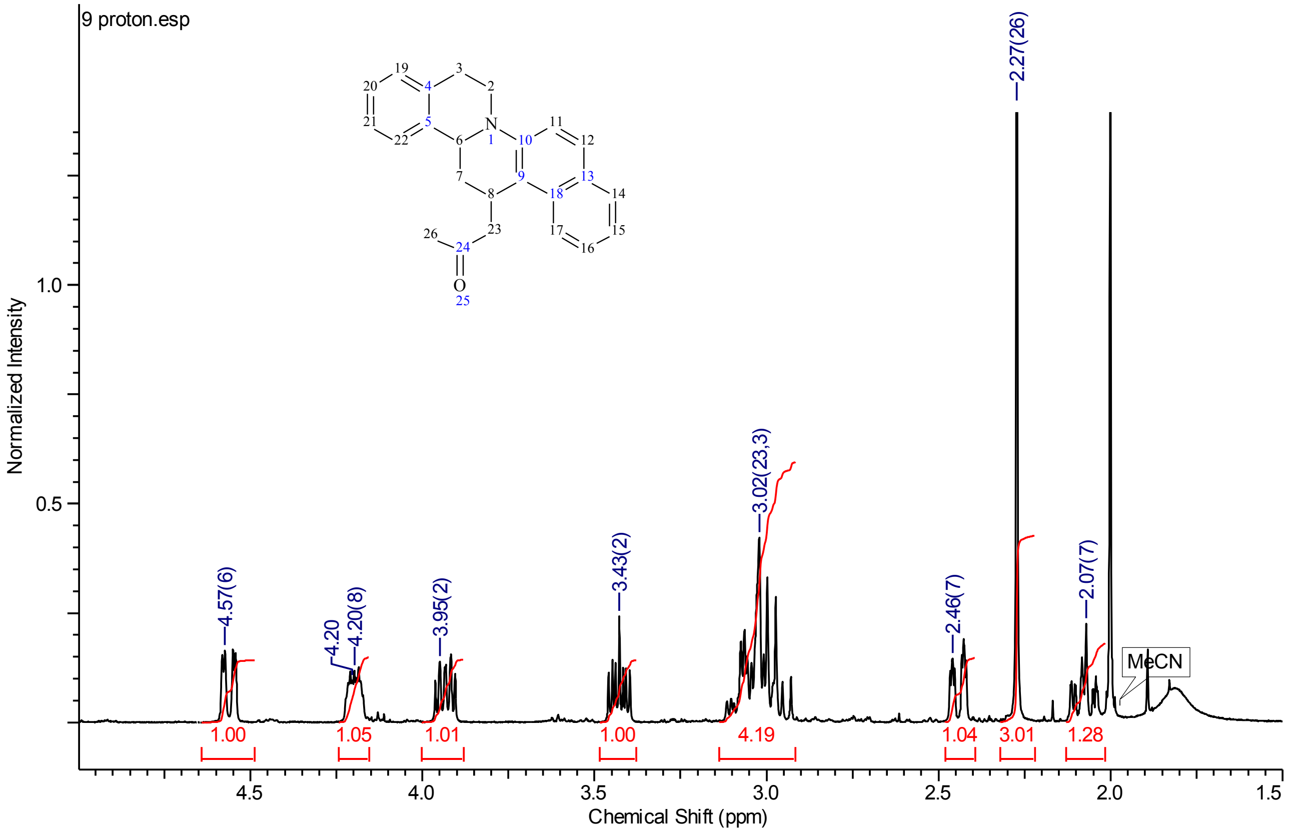
9 proton.esp
4.57(6)
4.20
4.20(8)
3.95(2)
3.43(2)
3.02(23,3)
2.46(7)
2.27(26)
2.07(7)
MeCN
1.00 1.05 1.01 1.00 4.19 1.04 3.01 1.28
Normalized Intensity
Chemical Shift (ppm)

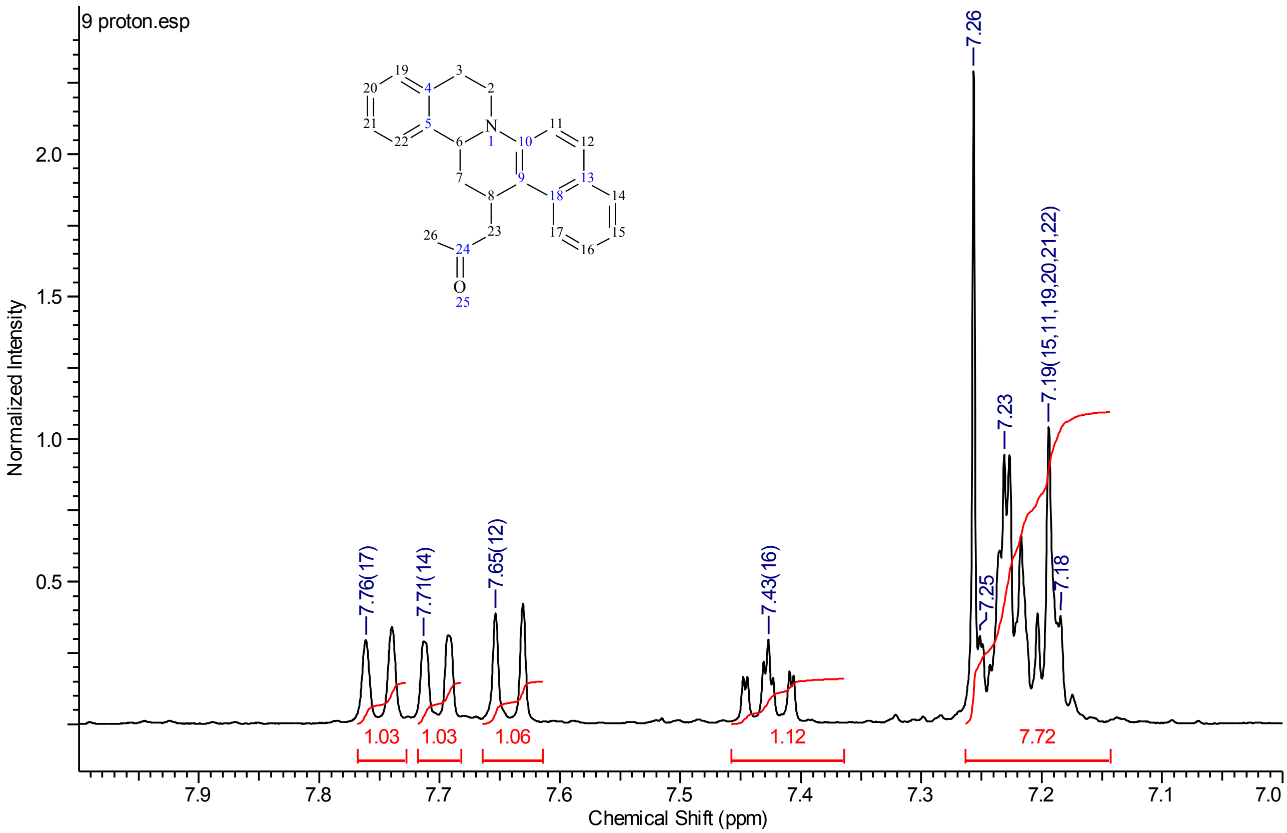
9 proton.esp
Normalized Intensity
Chemical Shift (ppm)
7.26
7.23
7.19(15,11,19,20,21,22)
7.25
7.18
7.76(17)
7.71(14)
7.65(12)
7.43(16)
1.03
1.03
1.06
1.12
7.72

^{13}C NMR – **9**

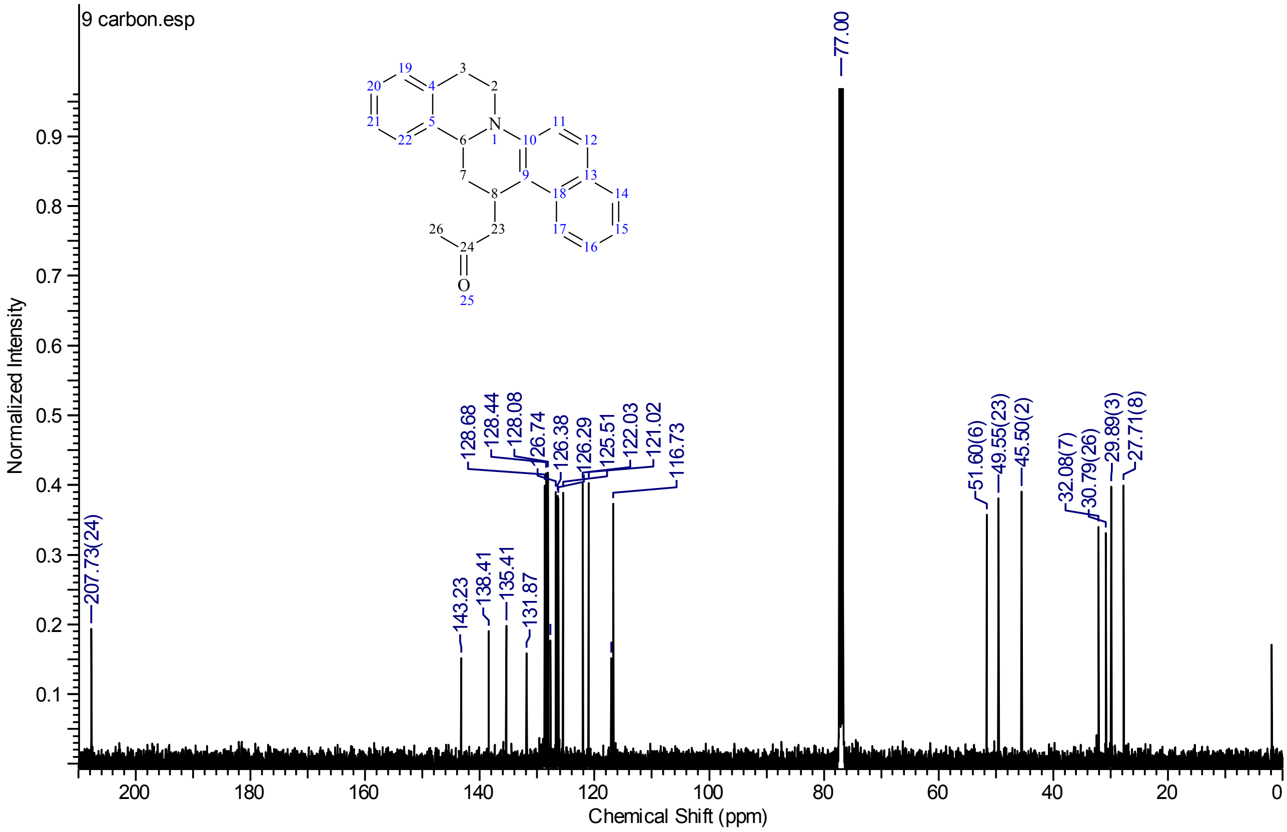
9 carbon.esp
Normalized Intensity
Chemical Shift (ppm)
77.00
207.73(24)
143.23
138.41
135.41
131.87
128.68
128.44
128.08
126.74
126.38
126.29
125.51
122.03
121.02
116.73
51.60(6)
49.55(23)
45.50(2)
32.08(7)
30.79(26)
29.89(3)
27.71(8)

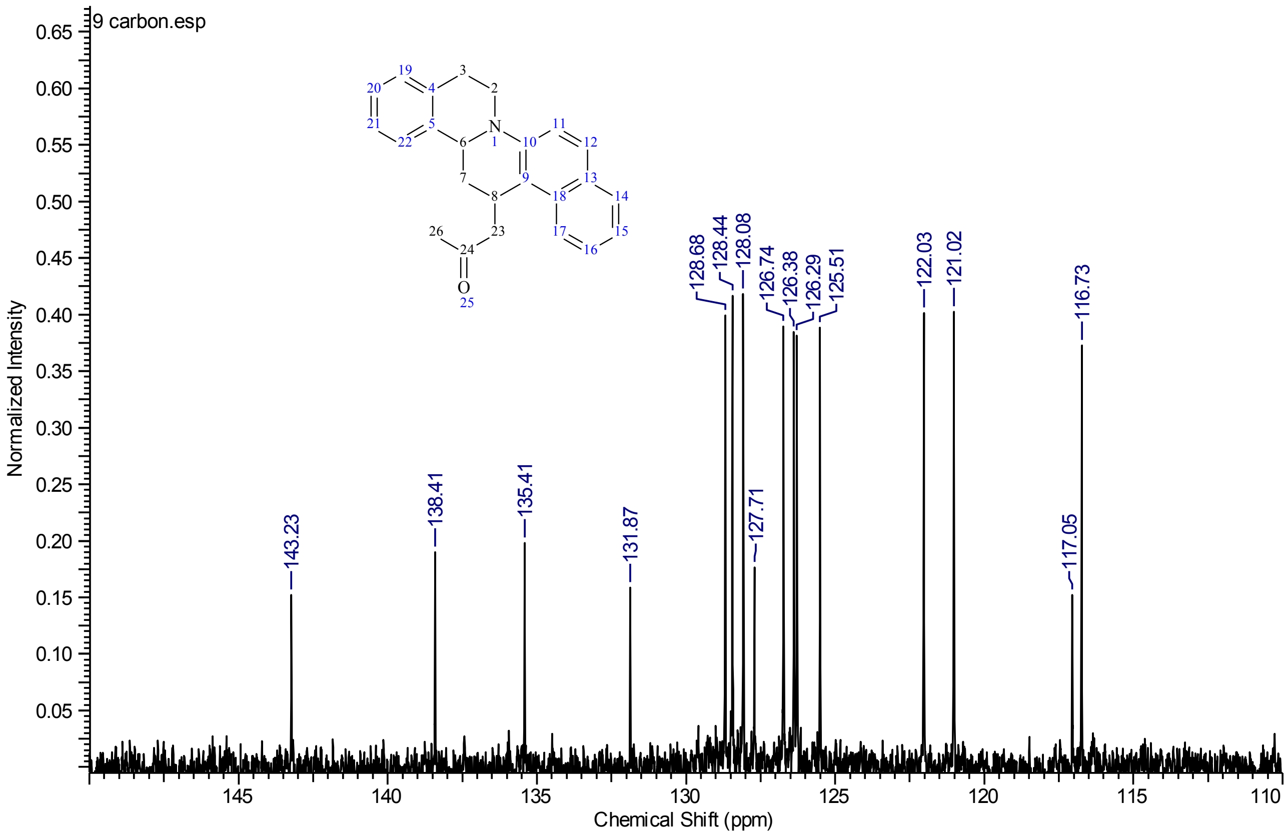
9 carbon.esp
143.23
138.41
135.41
131.87
128.68
128.44
128.08
127.71
126.74
126.38
126.29
125.51
122.03
121.02
117.05
116.73
Normalized Intensity
Chemical Shift (ppm)

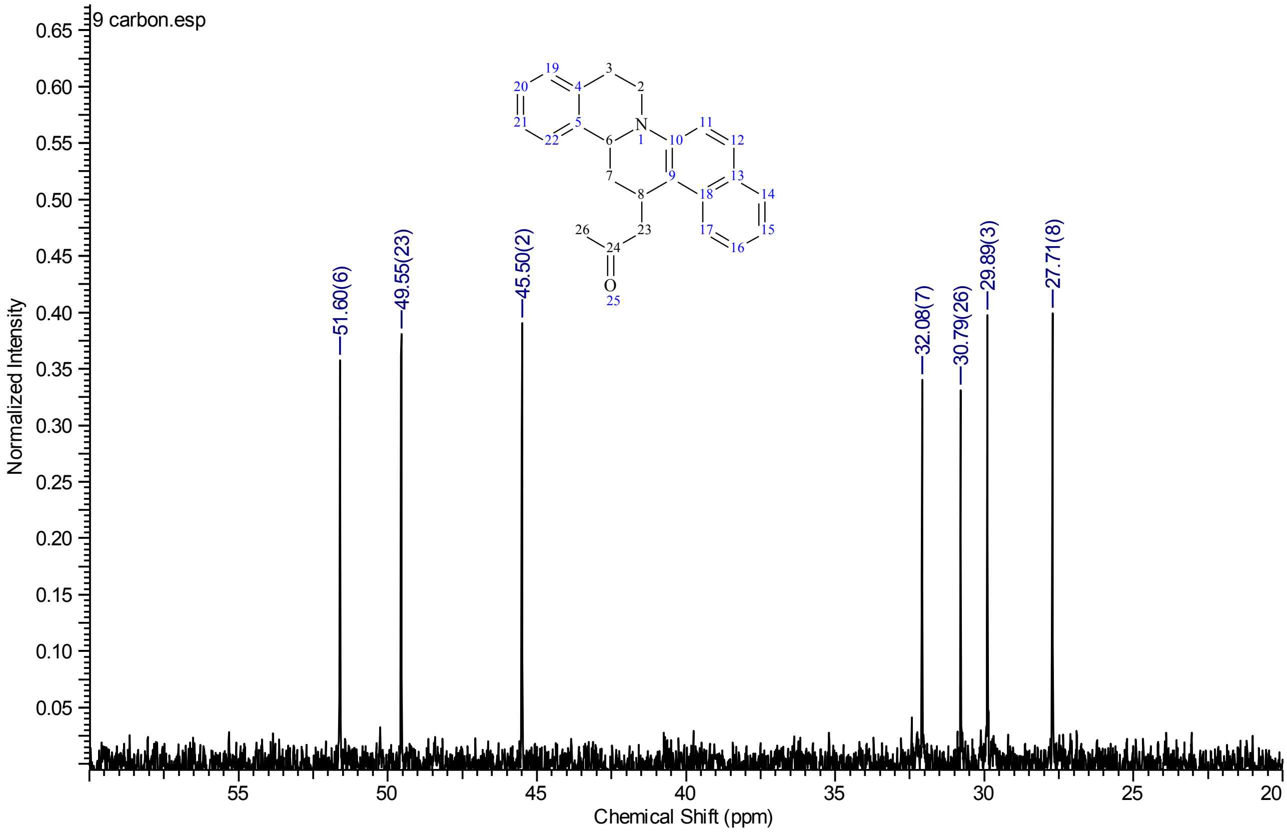
9 carbon.esp
51.60(6)
49.55(23)
45.50(2)
32.08(7)
30.79(26)
29.89(3)
27.71(8)
Normalized Intensity
Chemical Shift (ppm)

^{13}DEPT NMR – **9**

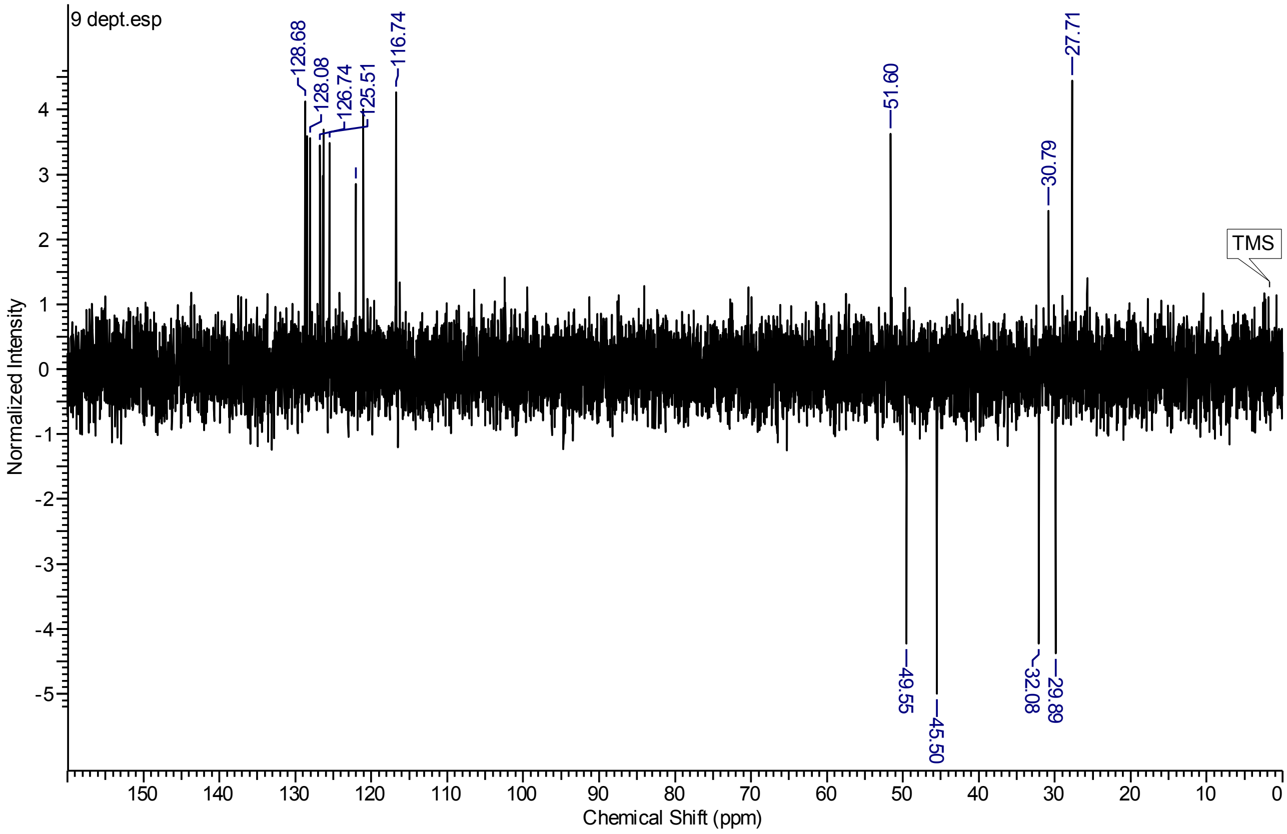

COSY NMR – **9**

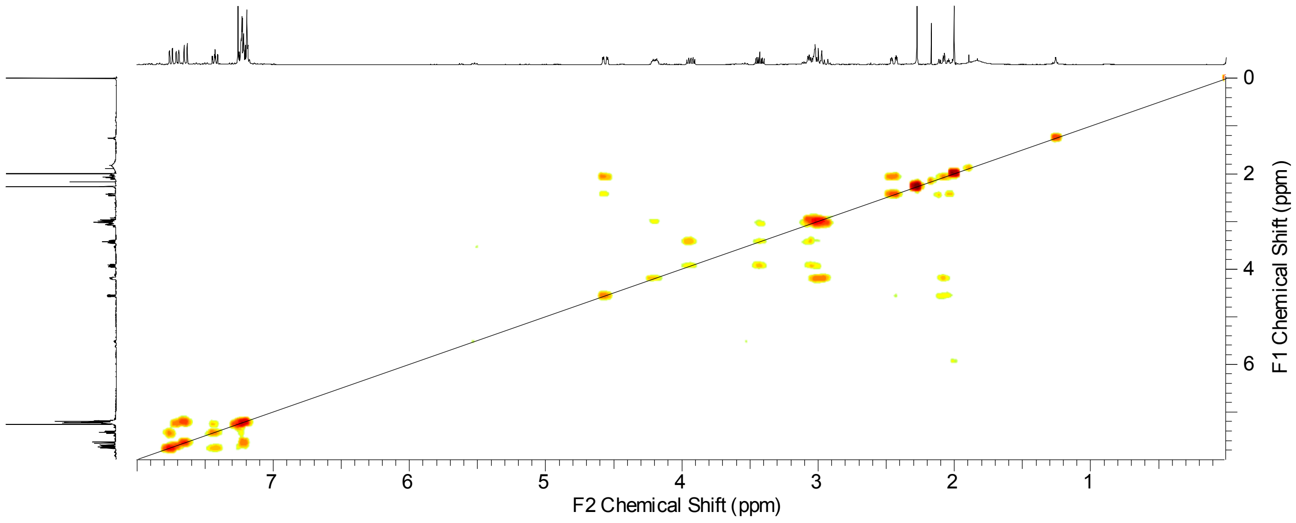

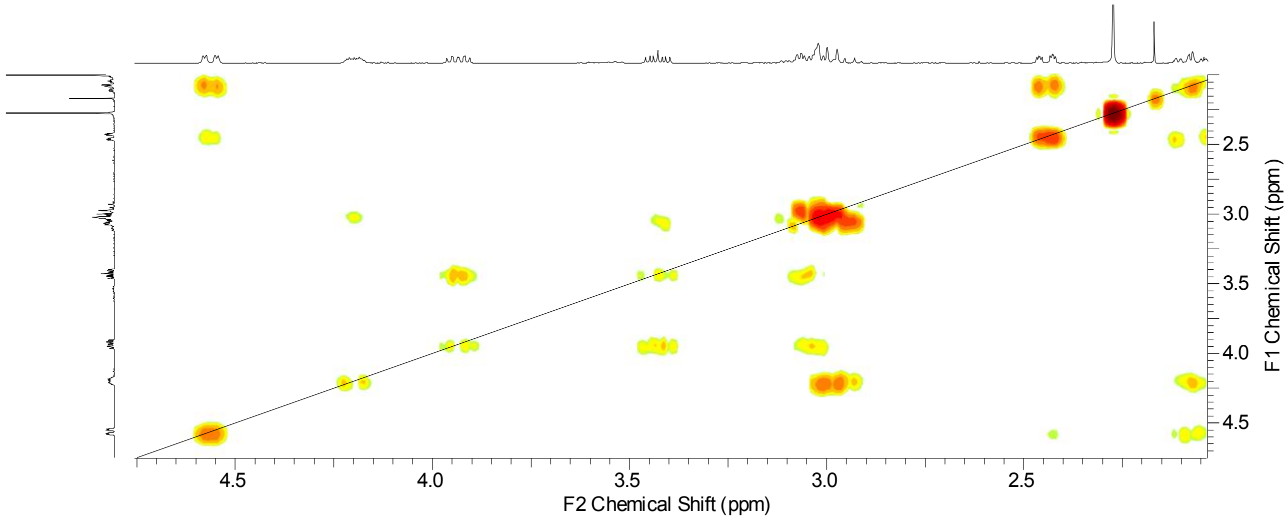
F1 Chemical Shift (ppm)
F2 Chemical Shift (ppm)

HQSC NMR – **9**

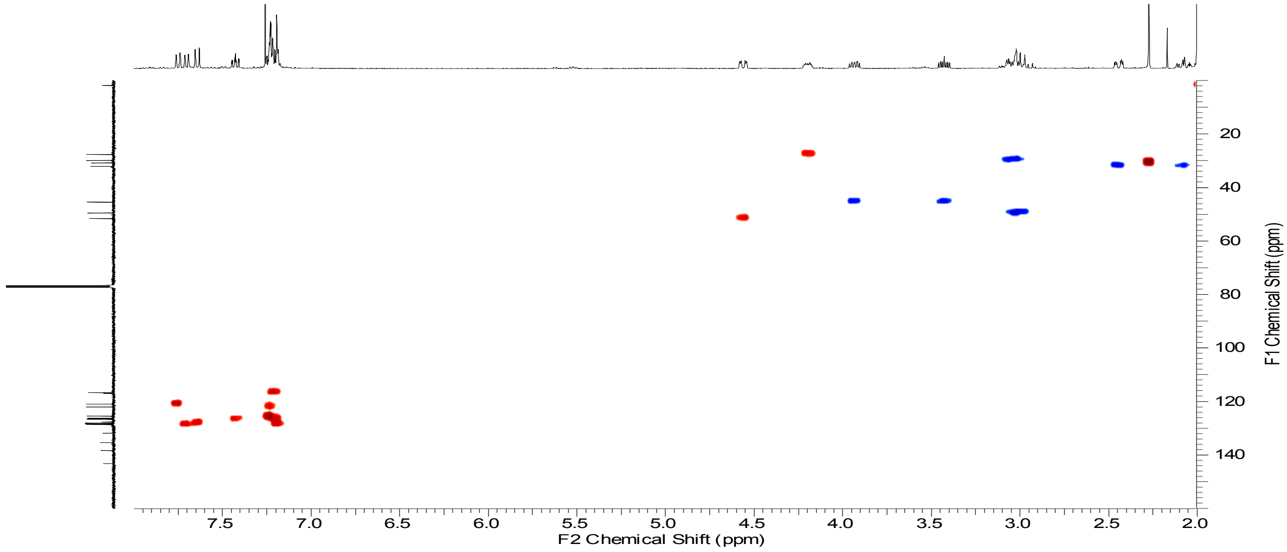
F1 Chemical Shift (ppm)
F2 Chemical Shift (ppm)

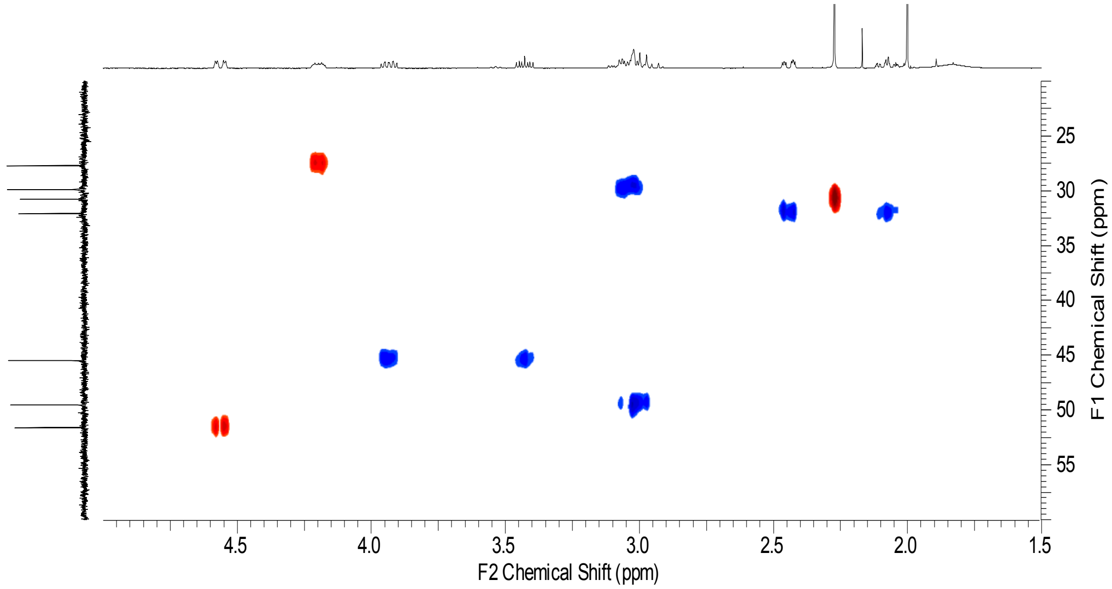
F1 Chemical Shift (ppm)
F2 Chemical Shift (ppm)

HMBC NMR – **9**

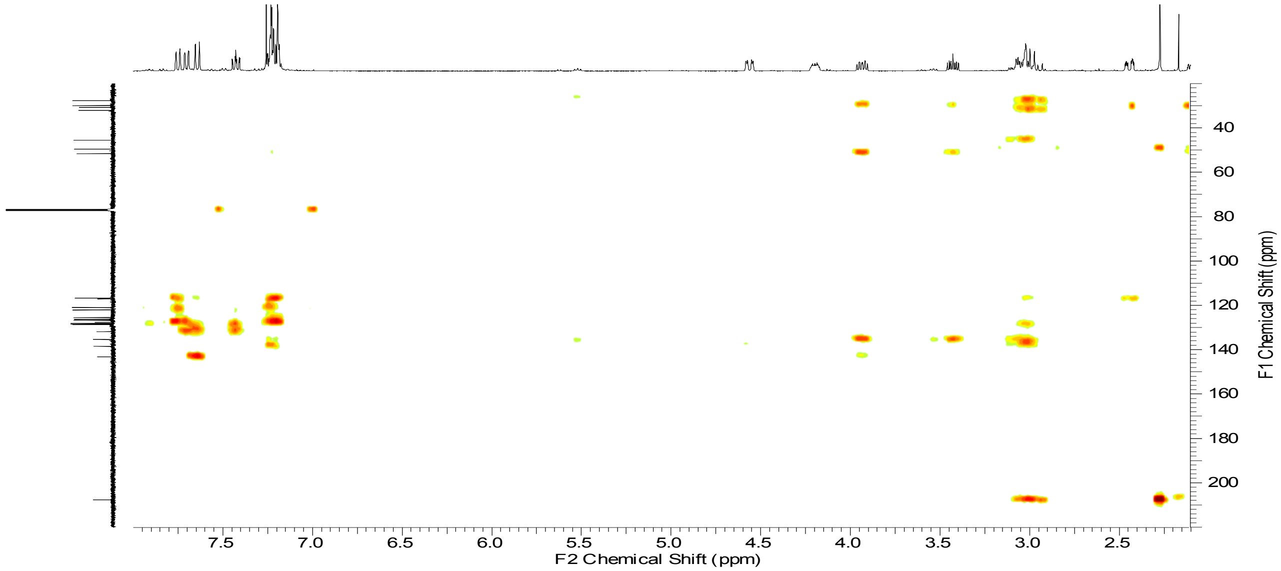

^{1}H NMR - **6al**

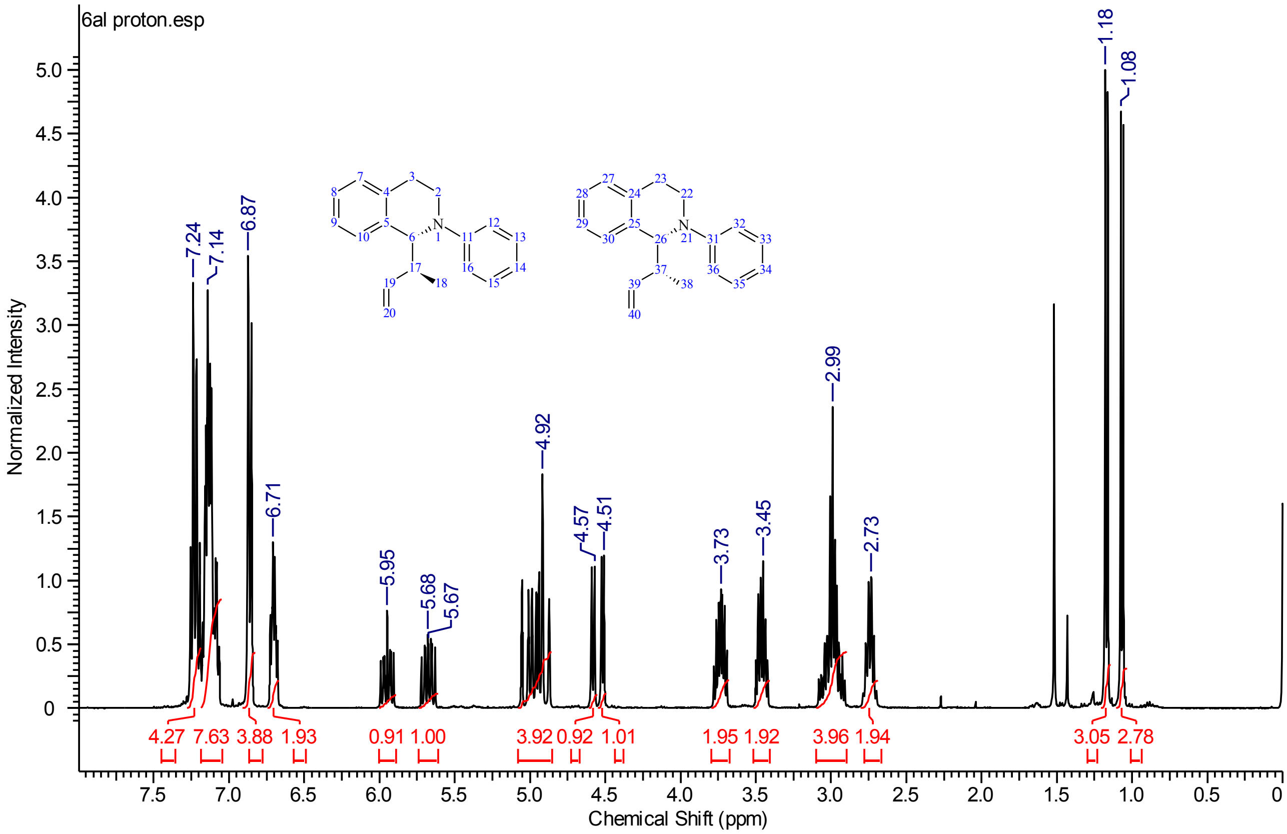

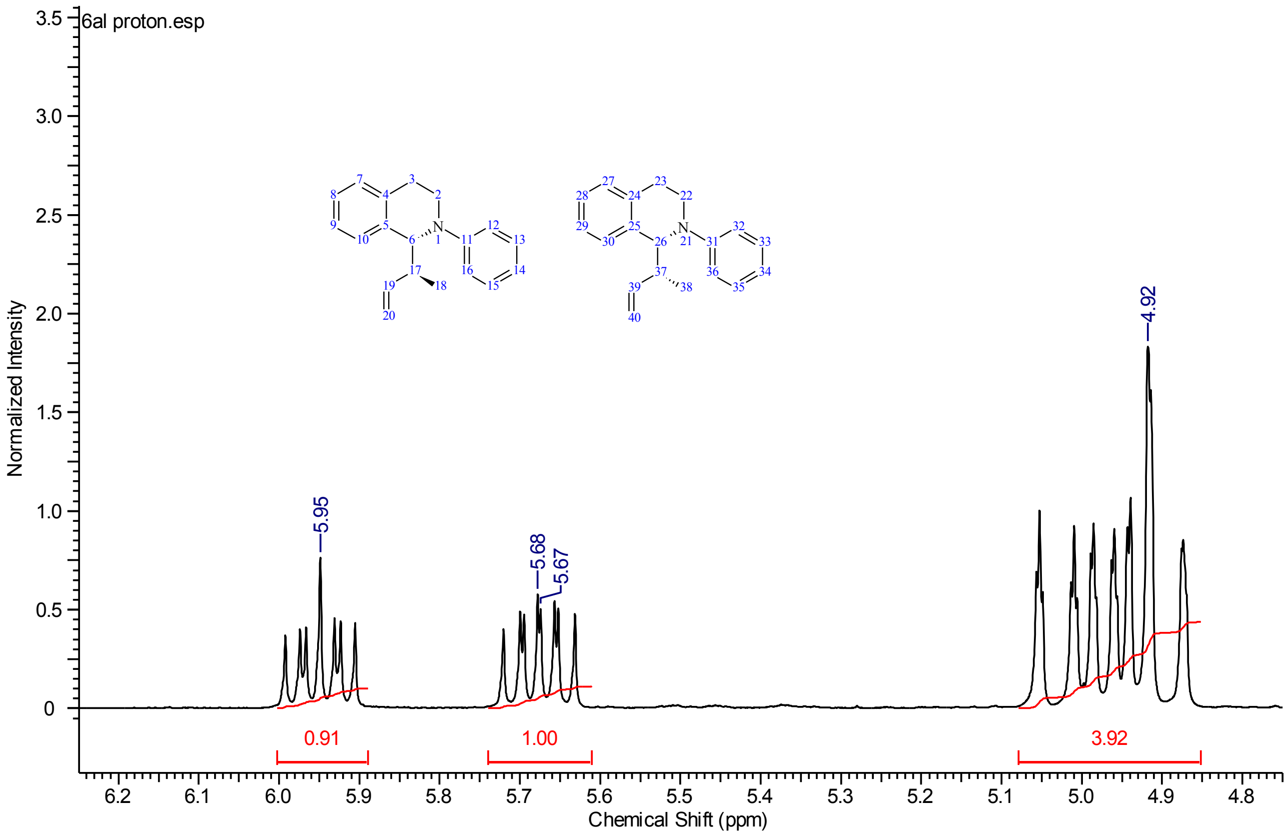
6al proton.esp
Normalized Intensity
Chemical Shift (ppm)
5.95
5.68
5.67
4.92
0.91
1.00
3.92

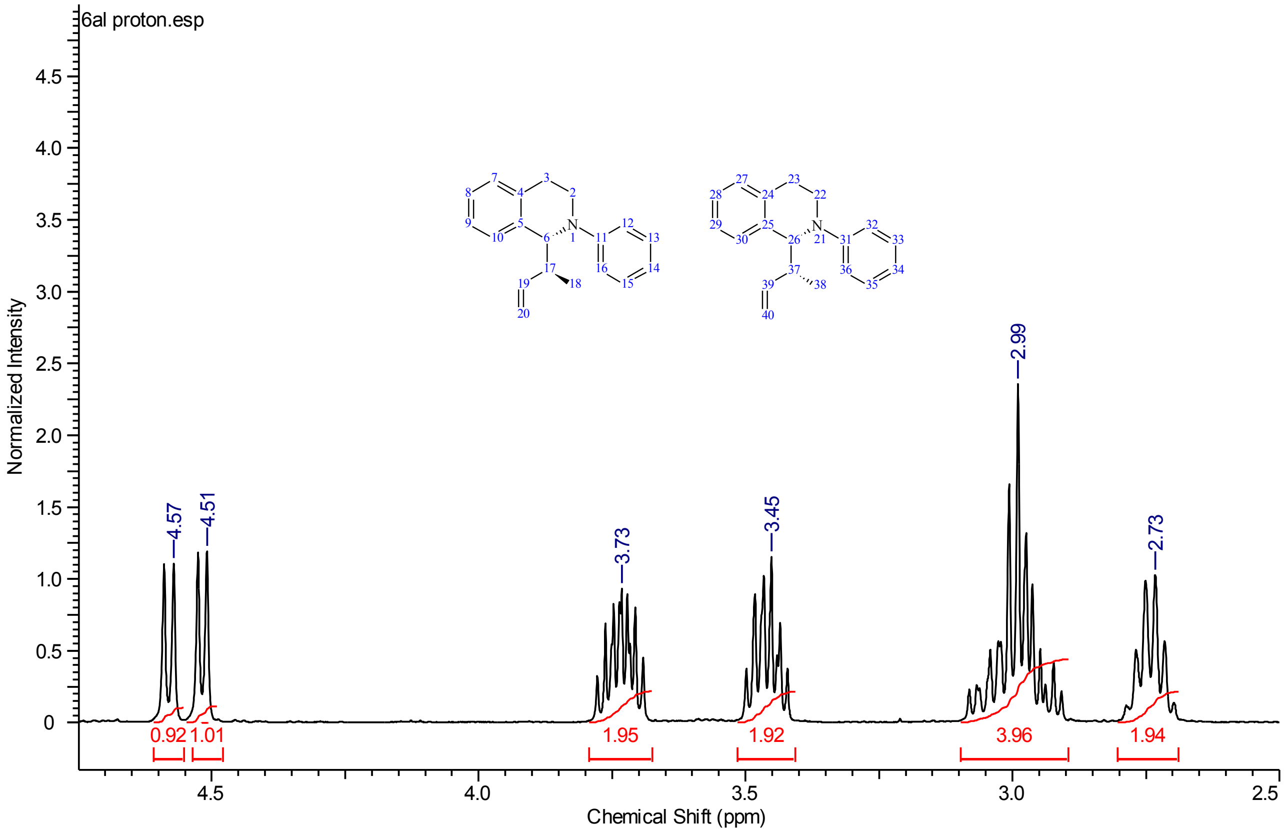
6al proton.esp
Normalized Intensity
Chemical Shift (ppm)
4.57
4.51
3.73
3.45
2.99
2.73
0.92
1.01
1.95
1.92
3.96
1.94

^{13}C NMR – **6al**

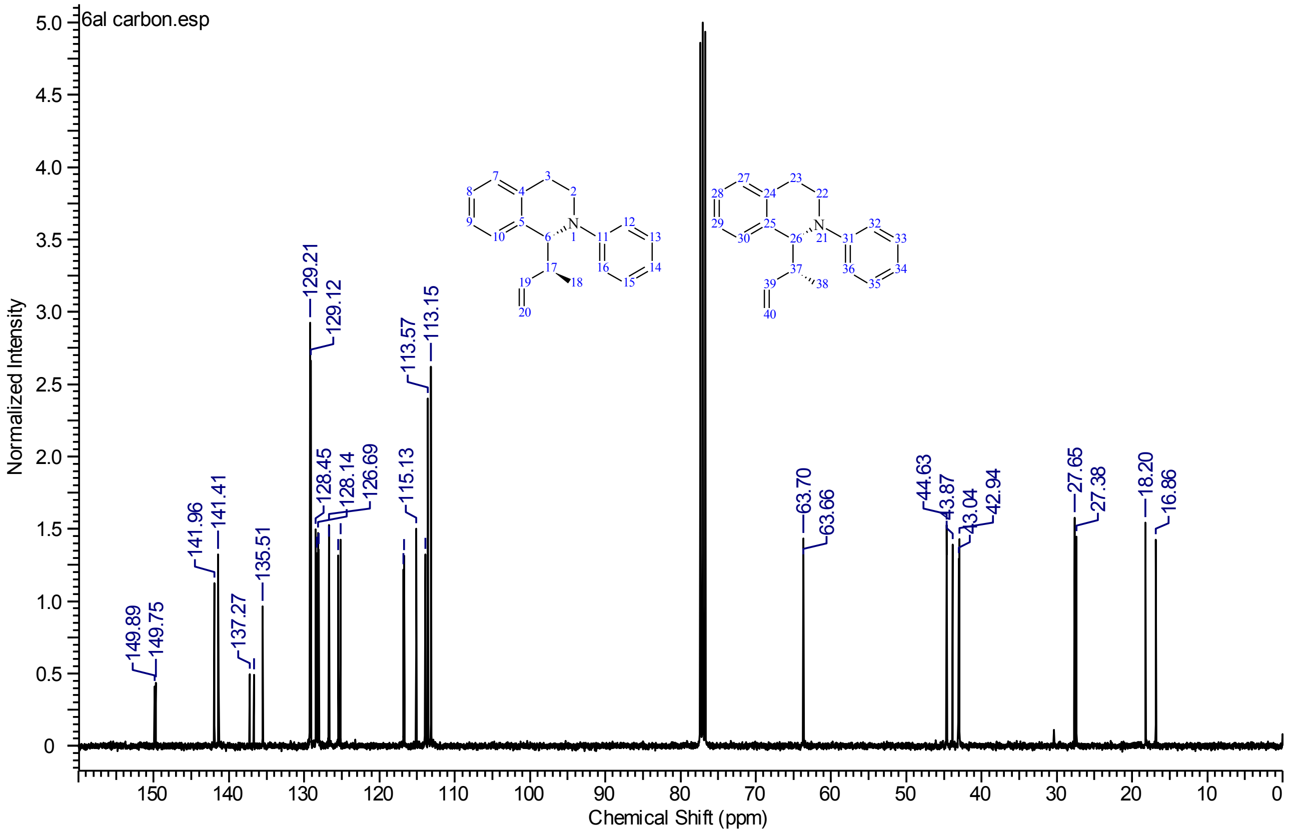
6al carbon.esp
Normalized Intensity
Chemical Shift (ppm)
149.89
149.75
141.96
141.41
137.27
135.51
129.21
129.12
128.45
128.14
126.69
115.13
113.57
113.15
63.70
63.66
44.63
43.87
43.04
42.94
27.65
27.38
18.20
16.86

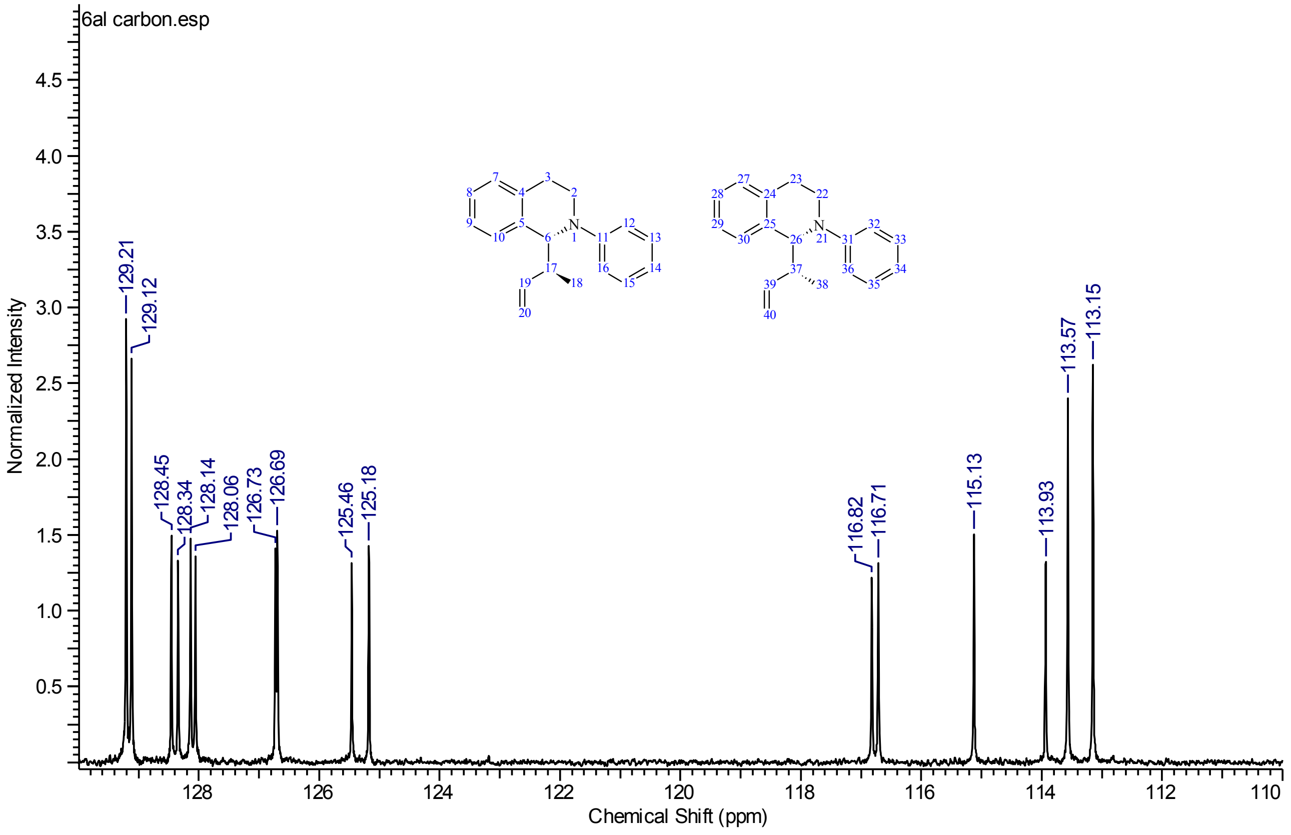
6al carbon.esp
Normalized Intensity
Chemical Shift (ppm)
129.21
129.12
128.45
128.34
128.14
128.06
126.73
126.69
125.46
125.18
116.82
116.71
115.13
113.93
113.57
113.15

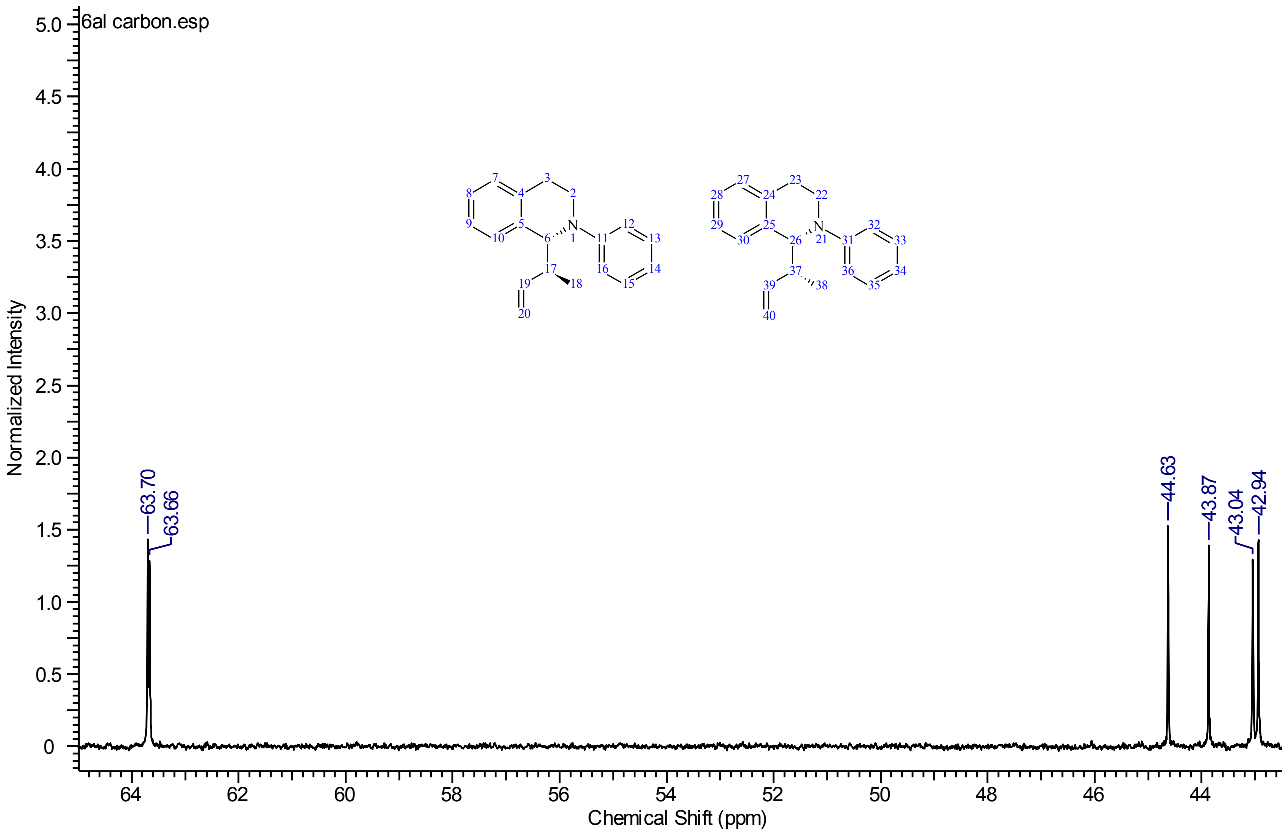
6al carbon.esp
Normalized Intensity
Chemical Shift (ppm)
63.70
63.66
44.63
43.87
43.04
42.94

^{1}H NMR – **6bb**

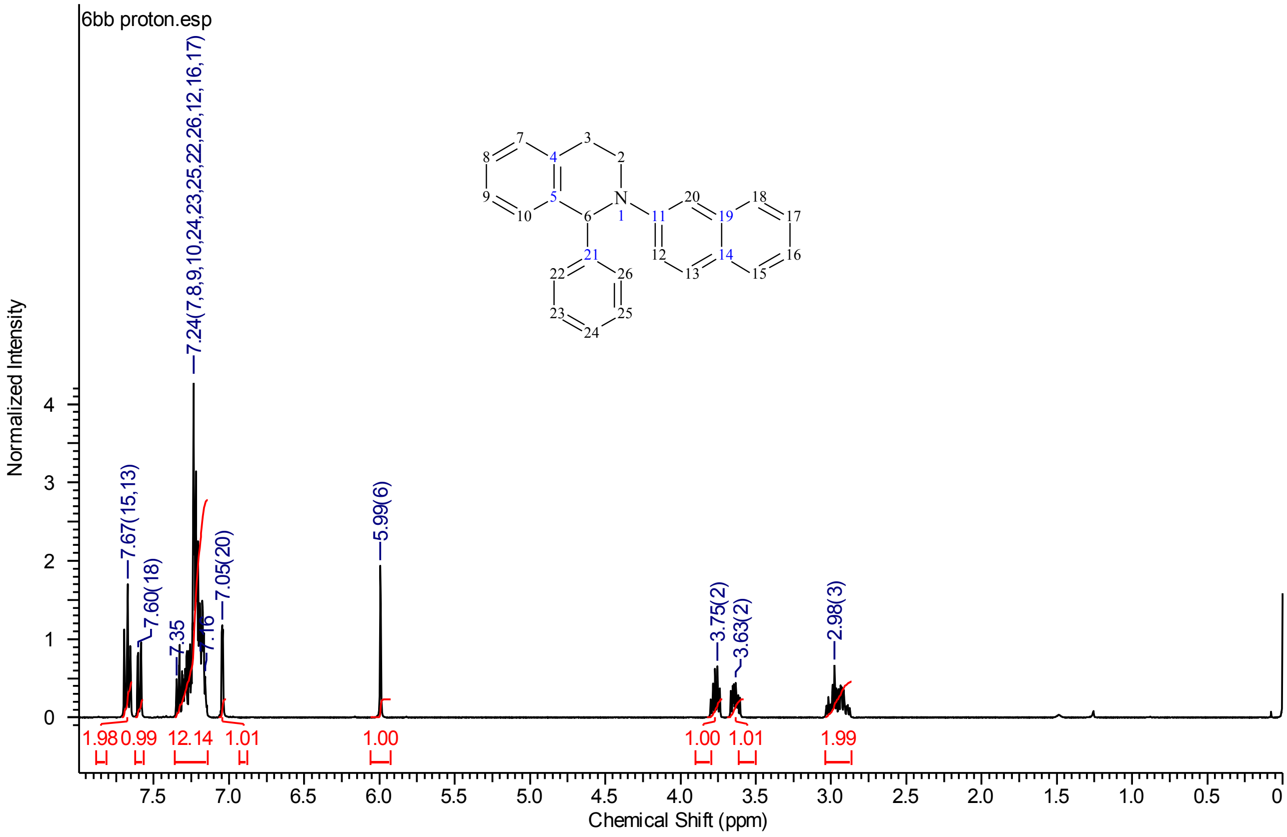
6bb proton.esp
Normalized Intensity
Chemical Shift (ppm)
7.67(15,13)
7.60(18)
7.35
7.24(7,8,9,10,24,23,25,22,26,12,16,17)
7.16
7.05(20)
5.99(6)
3.75(2)
3.63(2)
2.98(3)
1.98
0.99
12.14
1.01
1.00
1.00
1.01
1.99

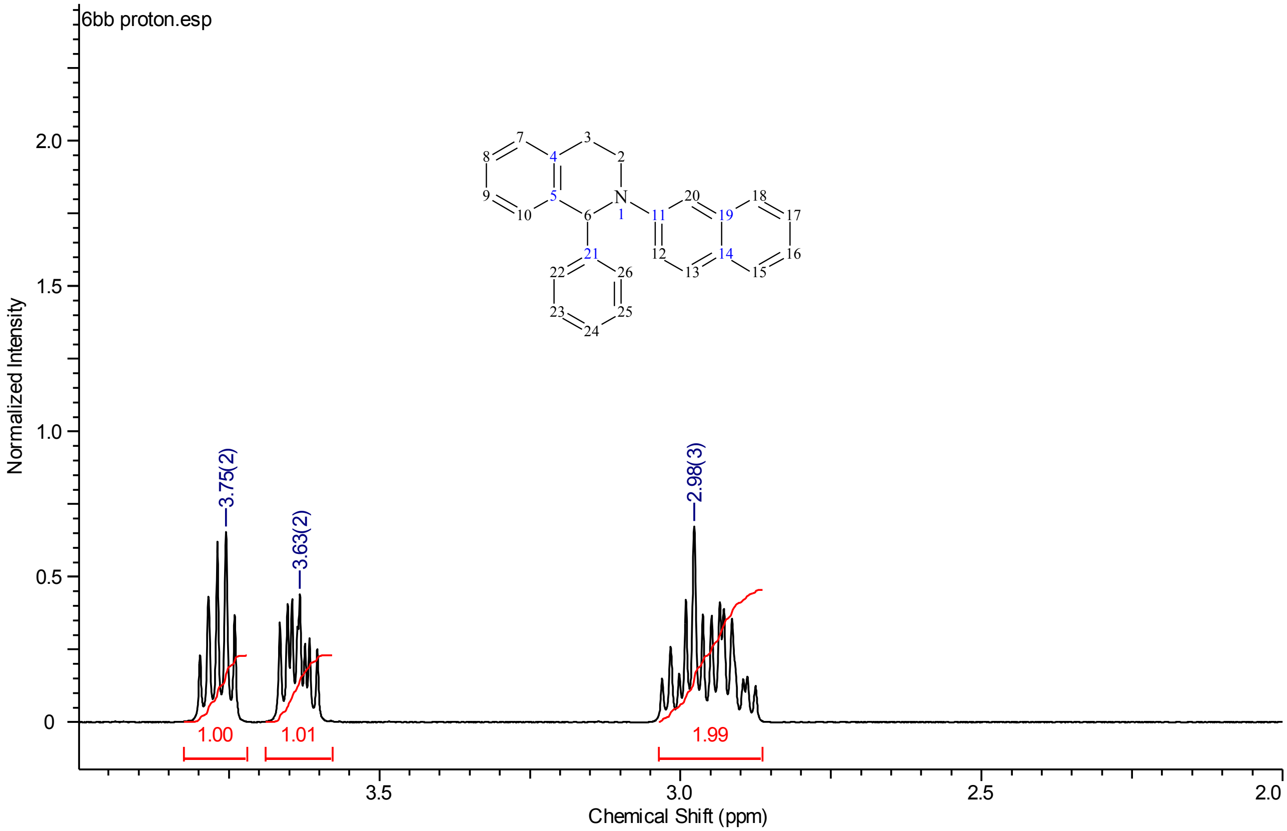
6bb proton.esp
Normalized Intensity
Chemical Shift (ppm)
3.75(2)
3.63(2)
2.98(3)
1.00
1.01
1.99

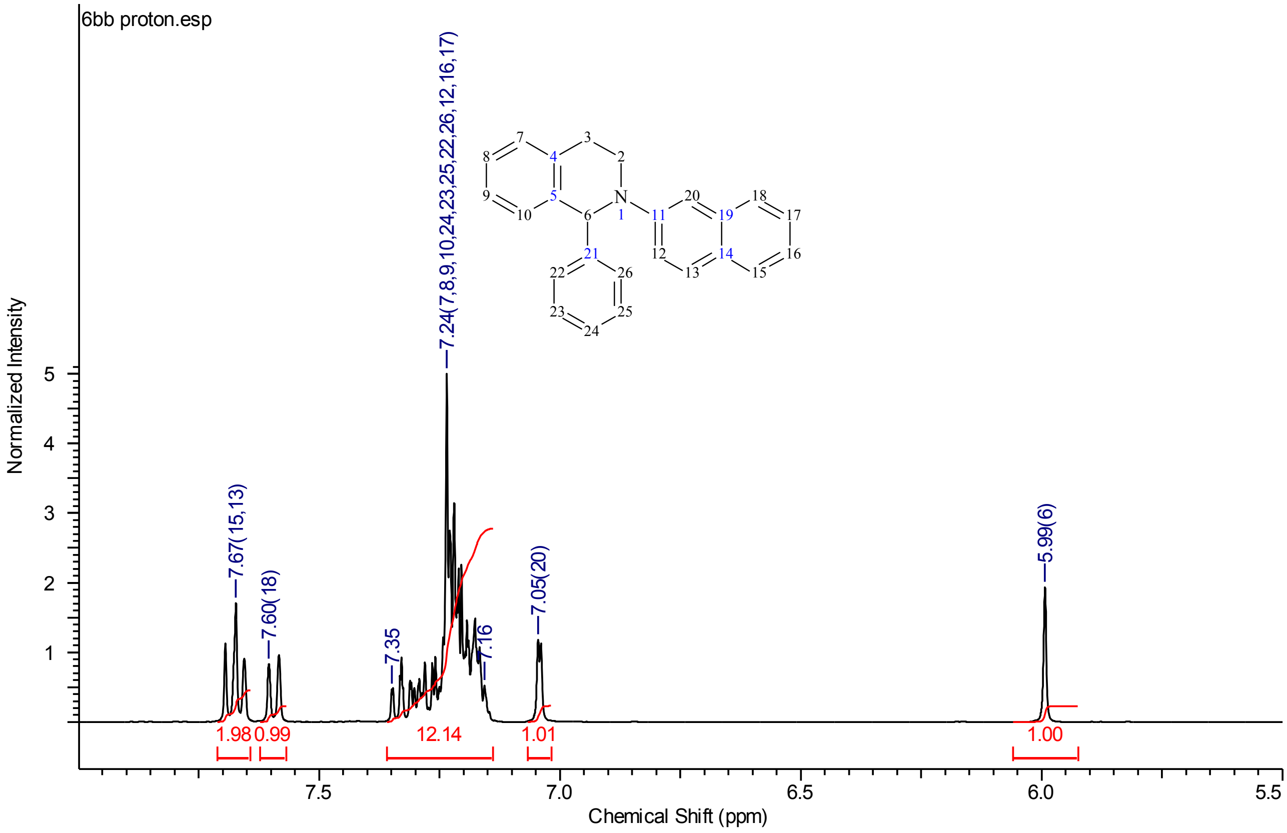
6bb proton.esp
Normalized Intensity
Chemical Shift (ppm)
7.67(15,13)
7.60(18)
7.35
7.24(7,8,9,10,24,23,25,22,26,12,16,17)
7.16
7.05(20)
5.99(6)
1.98
0.99
12.14
1.01
1.00

^{13}C NMR – **6bb**

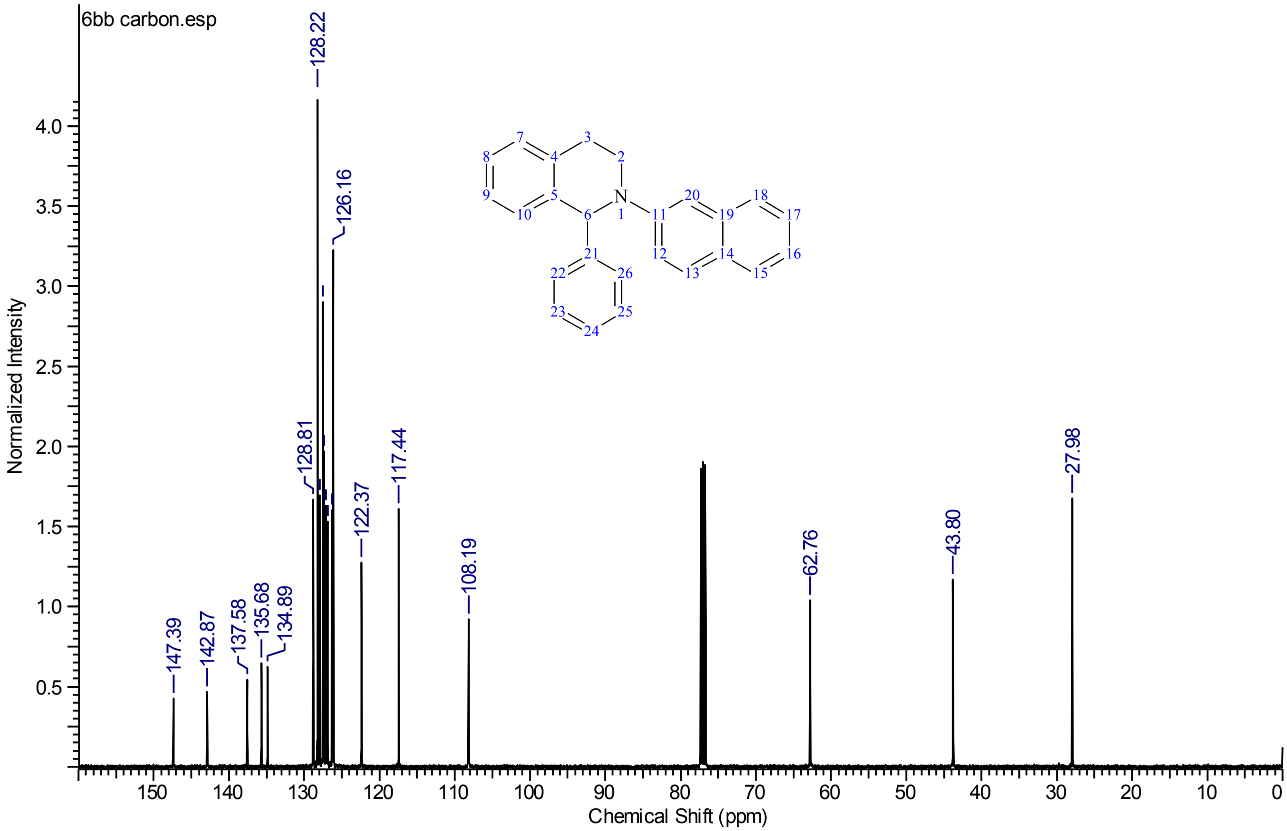

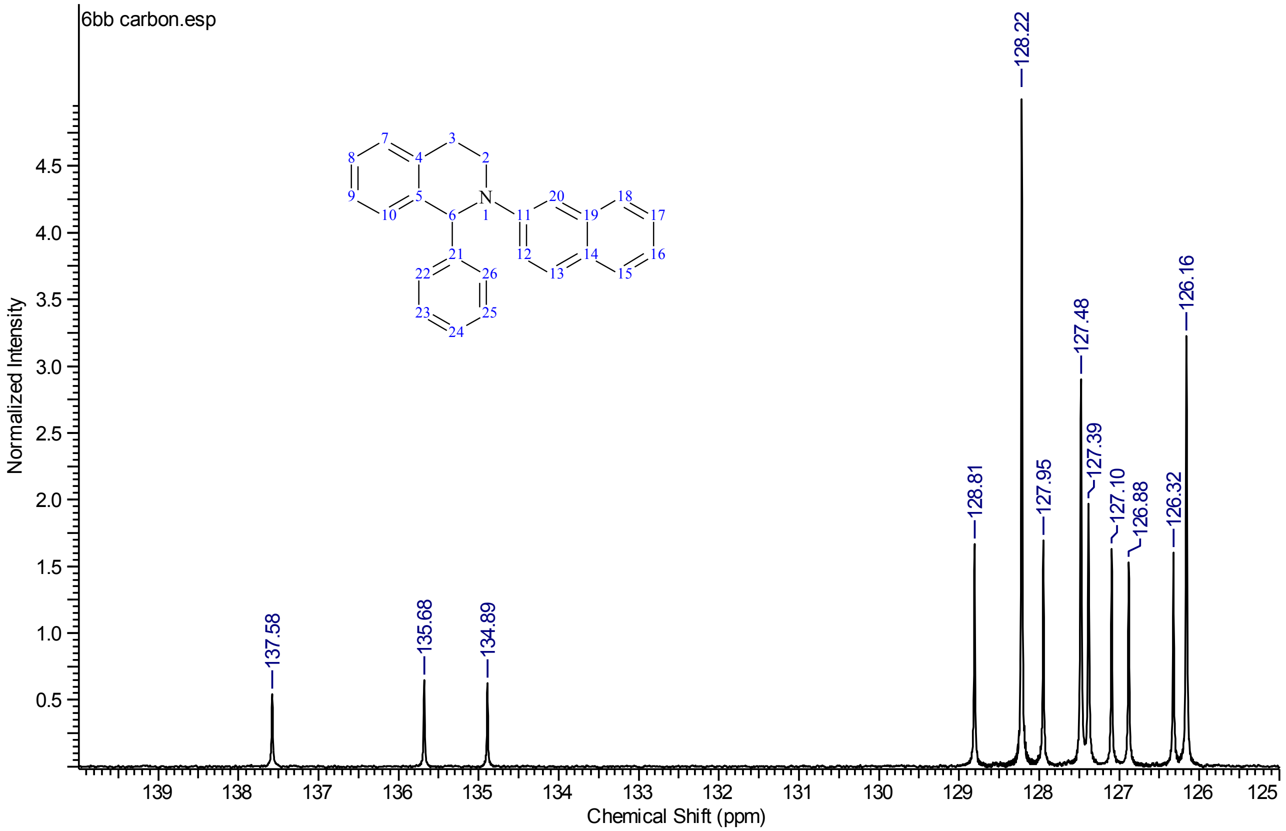

^{1}H NMR – **11b**

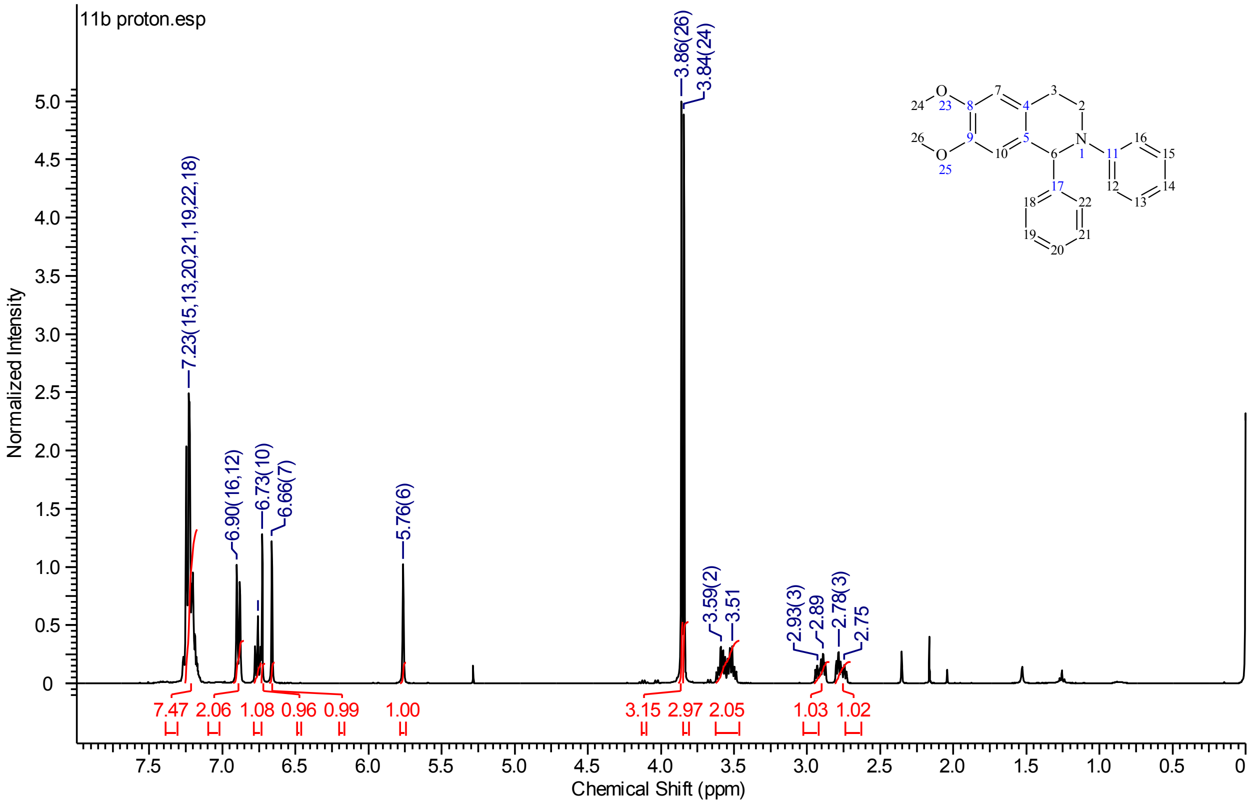
11b proton.esp
7.23(15,13,20,21,19,22,18)
6.90(16,12)
6.73(10)
6.66(7)
5.76(6)
3.86(26)
3.84(24)
3.59(2)
3.51
2.93(3)
2.89
2.78(3)
2.75
7.47 2.06 1.08 0.96 0.99 1.00 3.15 2.97 2.05 1.03 1.02
Normalized Intensity
Chemical Shift (ppm)

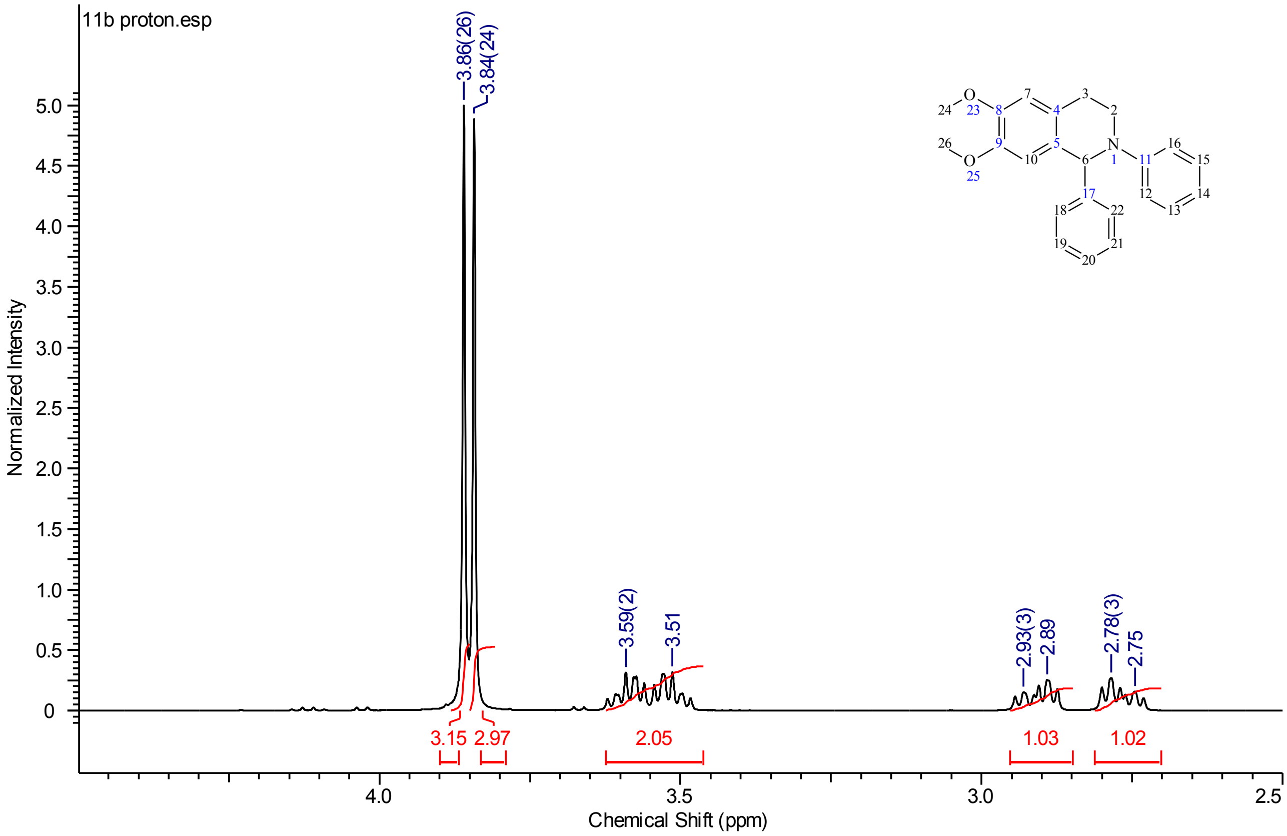
11b proton.esp
3.86(26)
3.84(24)
3.59(2)
3.51
2.93(3)
2.89
2.78(3)
2.75
3.15 2.97 2.05 1.03 1.02
Normalized Intensity
Chemical Shift (ppm)

^{13}C NMR – **11b**

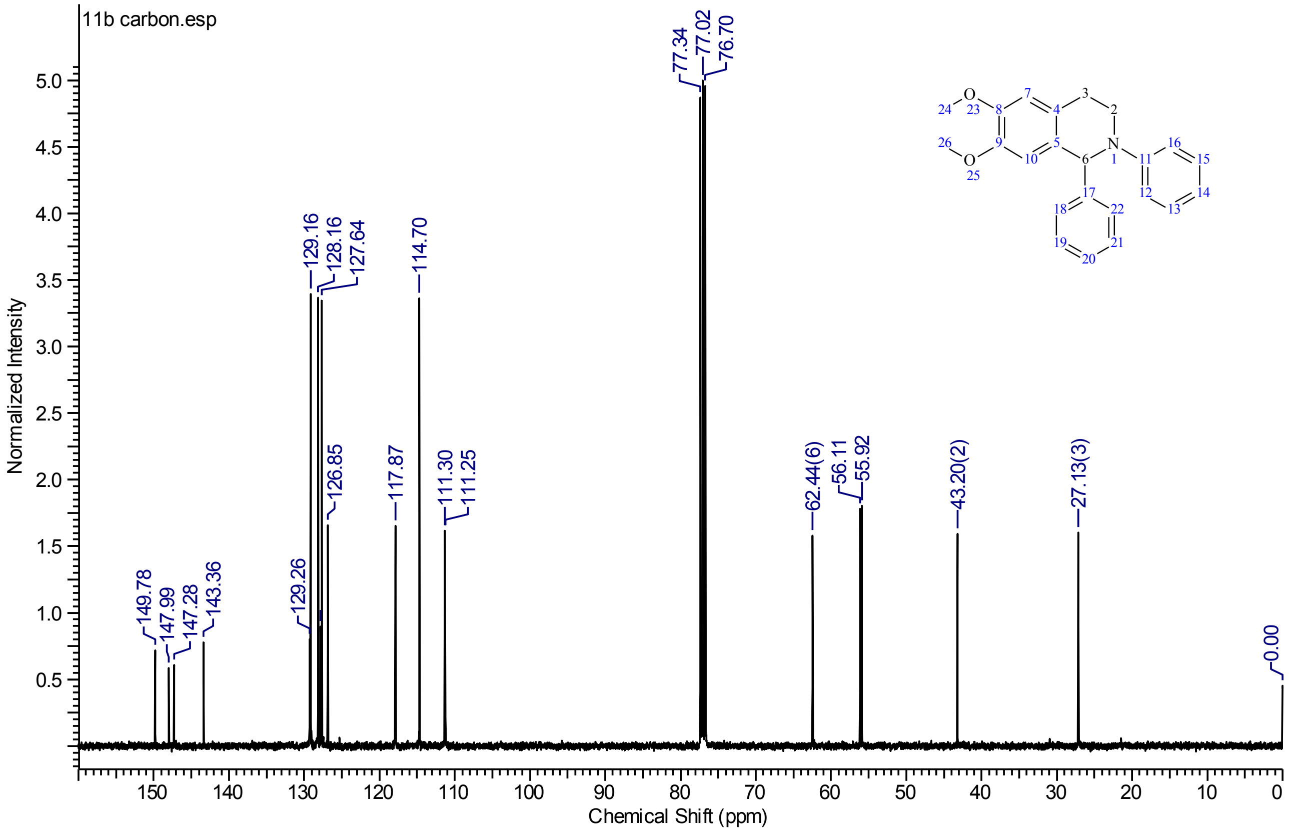

11b carbon.esp
Normalized Intensity
Chemical Shift (ppm)
149.78
147.99
147.28
143.36
129.26
129.16
128.16
127.64
126.85
117.87
114.70
111.30
111.25
77.34
77.02
76.70
62.44(6)
56.11
55.92
43.20(2)
27.13(3)
0.00

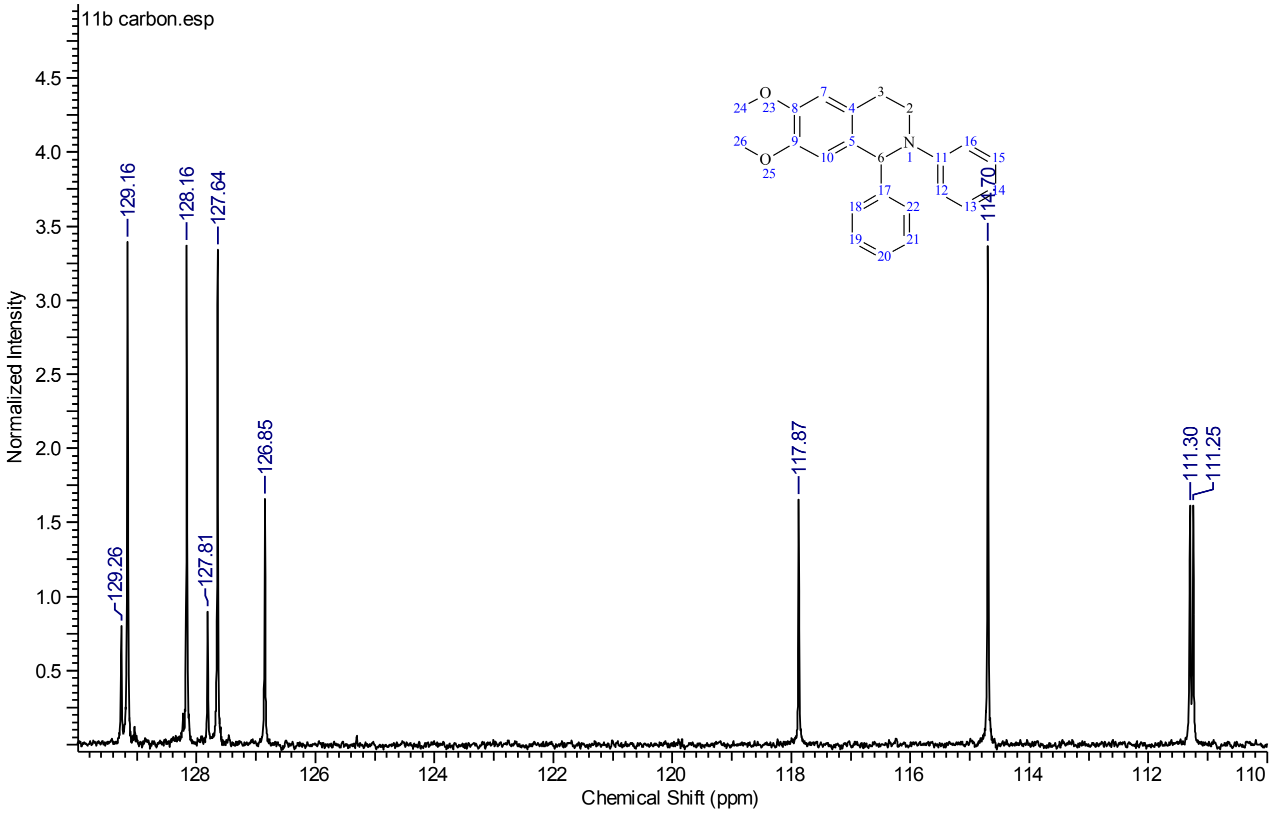

11b carbon.esp
Normalized Intensity
Chemical Shift (ppm)
129.26
129.16
128.16
127.81
127.64
126.85
117.87
114.70
111.30
111.25

^{1}H NMR – **13b**

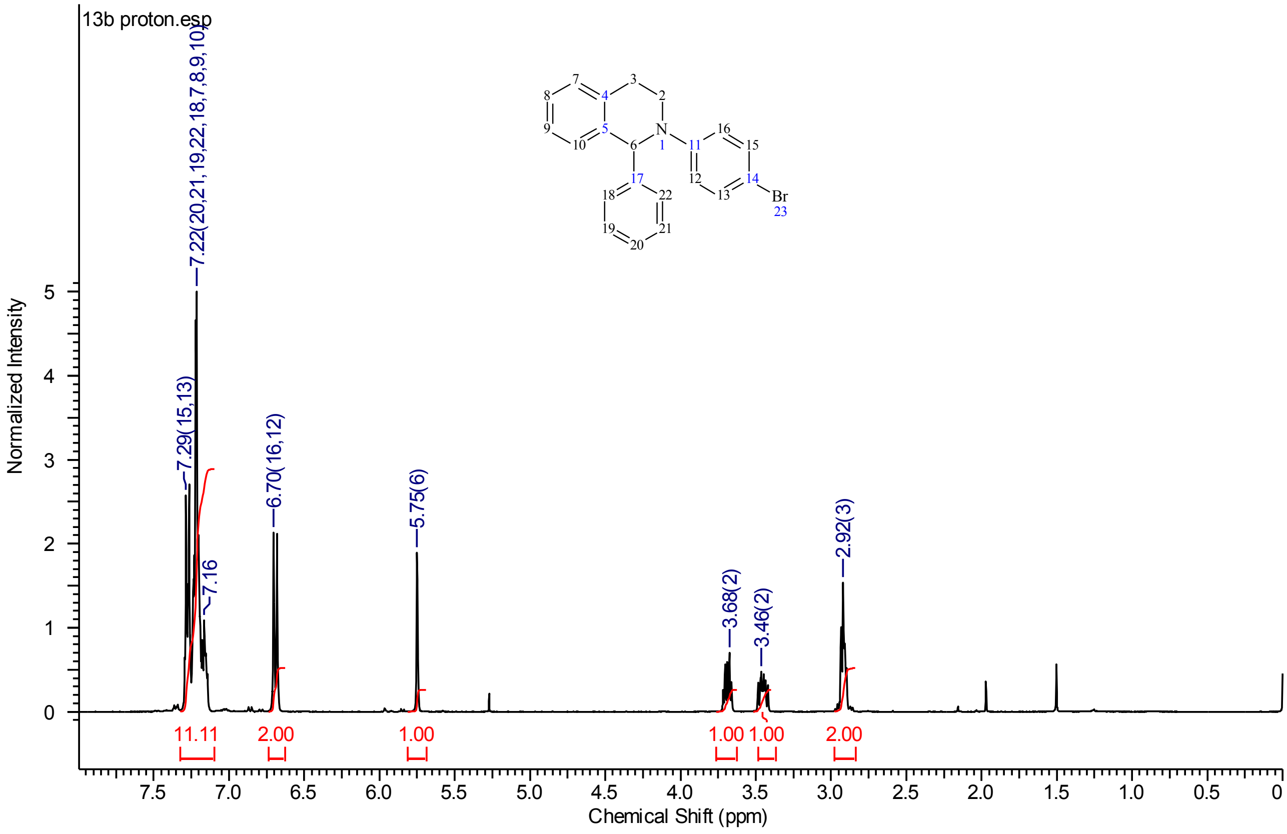

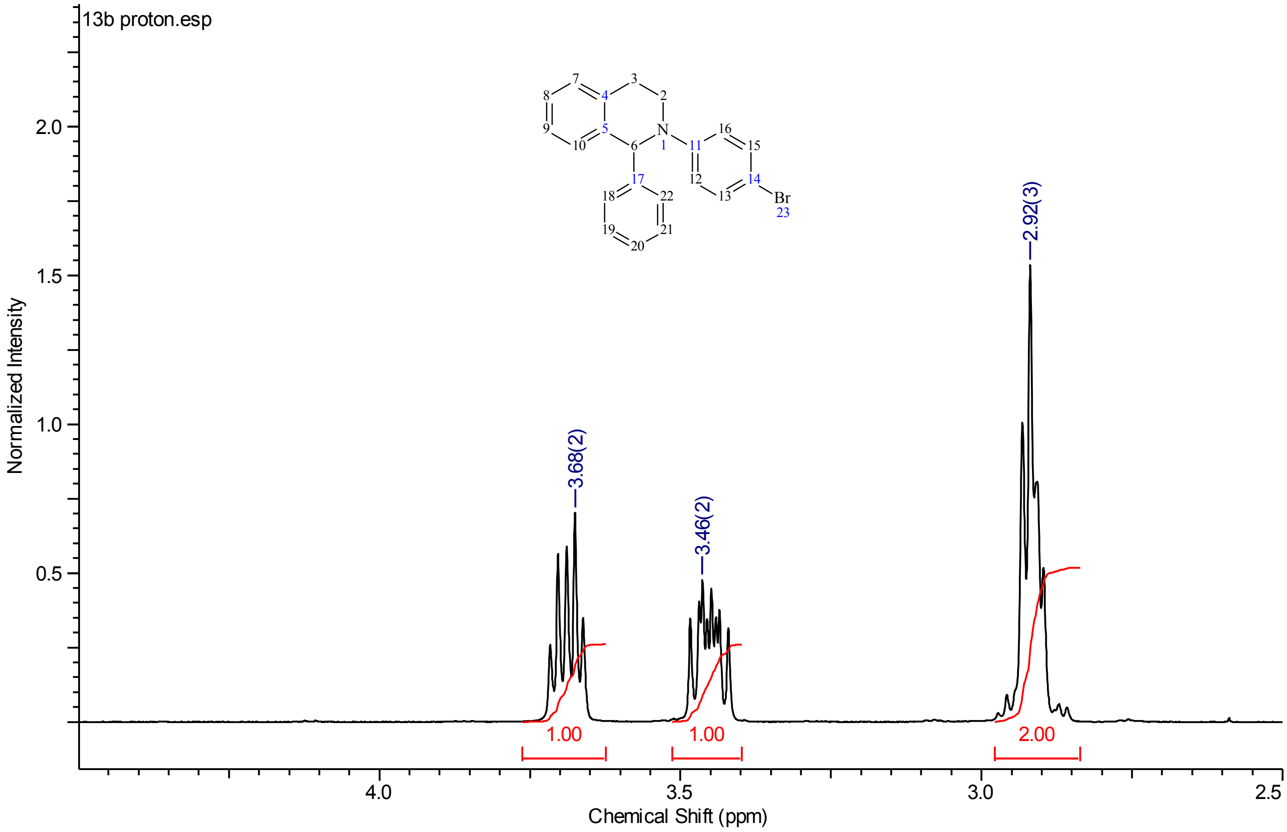

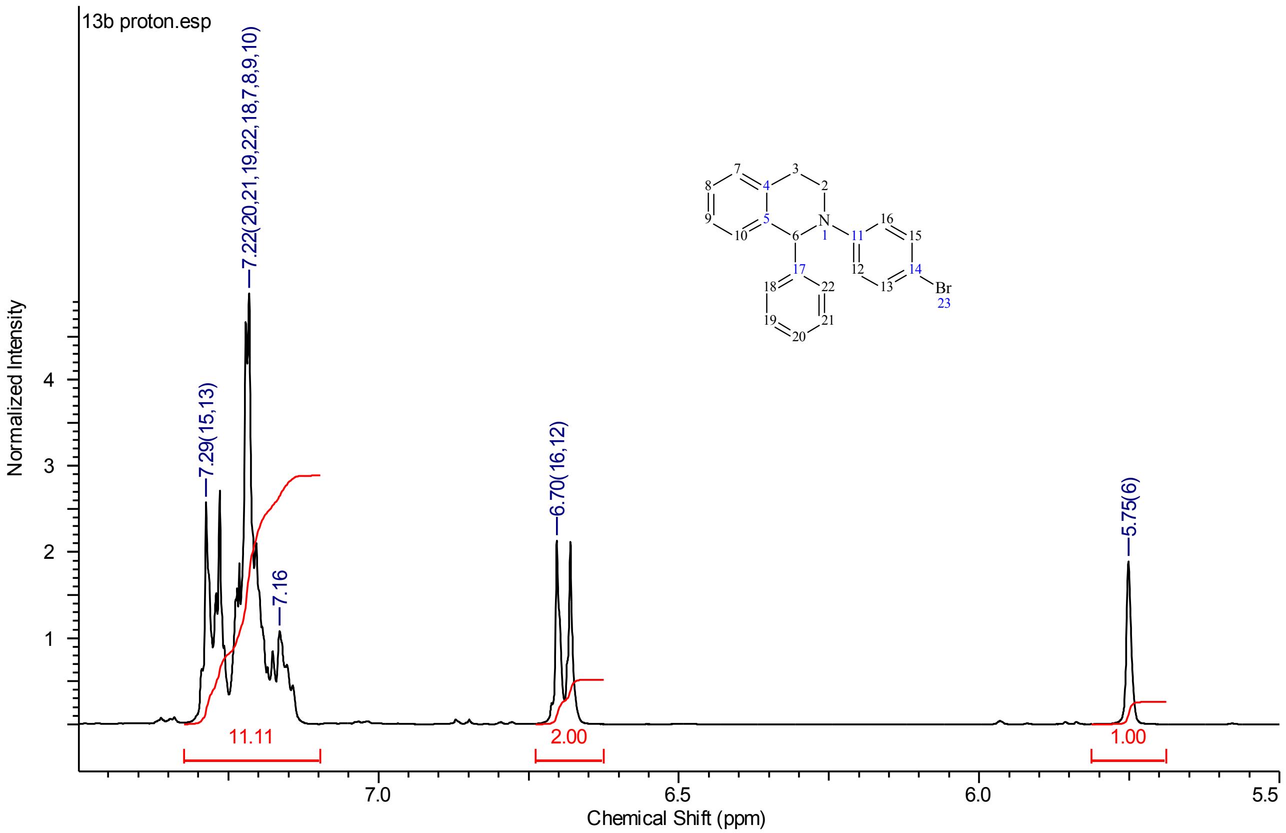
13b proton.esp
7.22(20,21,19,22,18,7,8,9,10)
7.29(15,13)
7.16
6.70(16,12)
5.75(6)
11.11
2.00
1.00
Normalized Intensity
Chemical Shift (ppm)

^{13}C NMR – **13b**

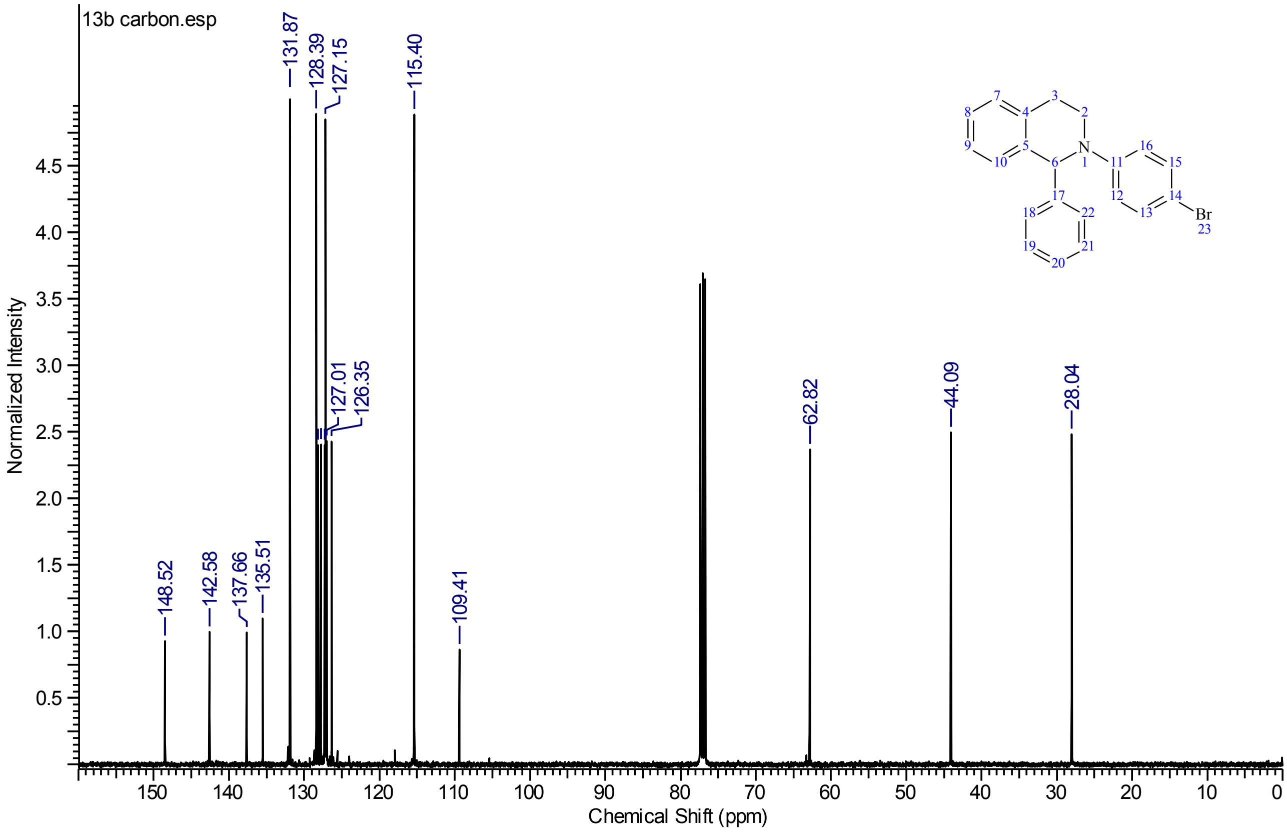
13b carbon.esp
131.87
128.39
127.15
115.40
127.01
126.35
148.52
142.58
137.66
135.51
109.41
62.82
44.09
28.04
Normalized Intensity
Chemical Shift (ppm)

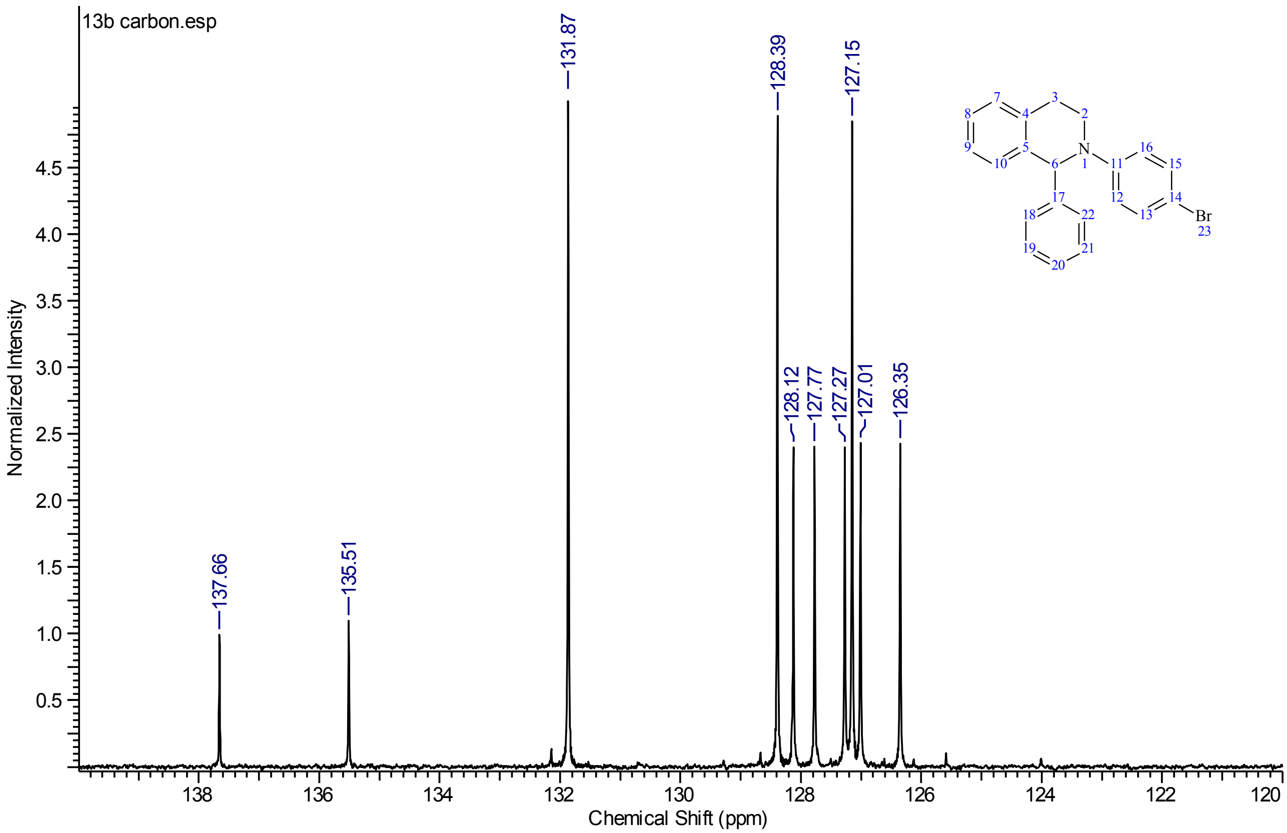

^{1}H NMR – **3**, methopholine

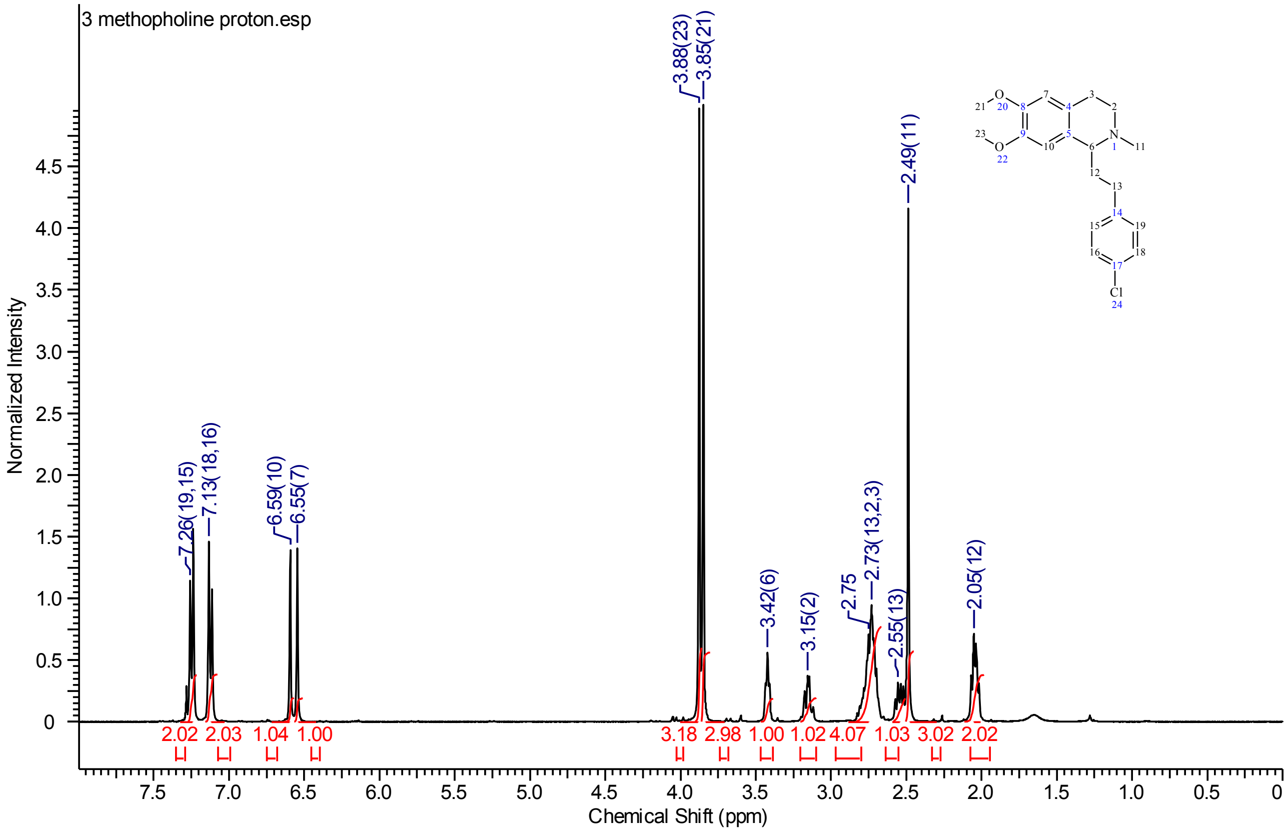

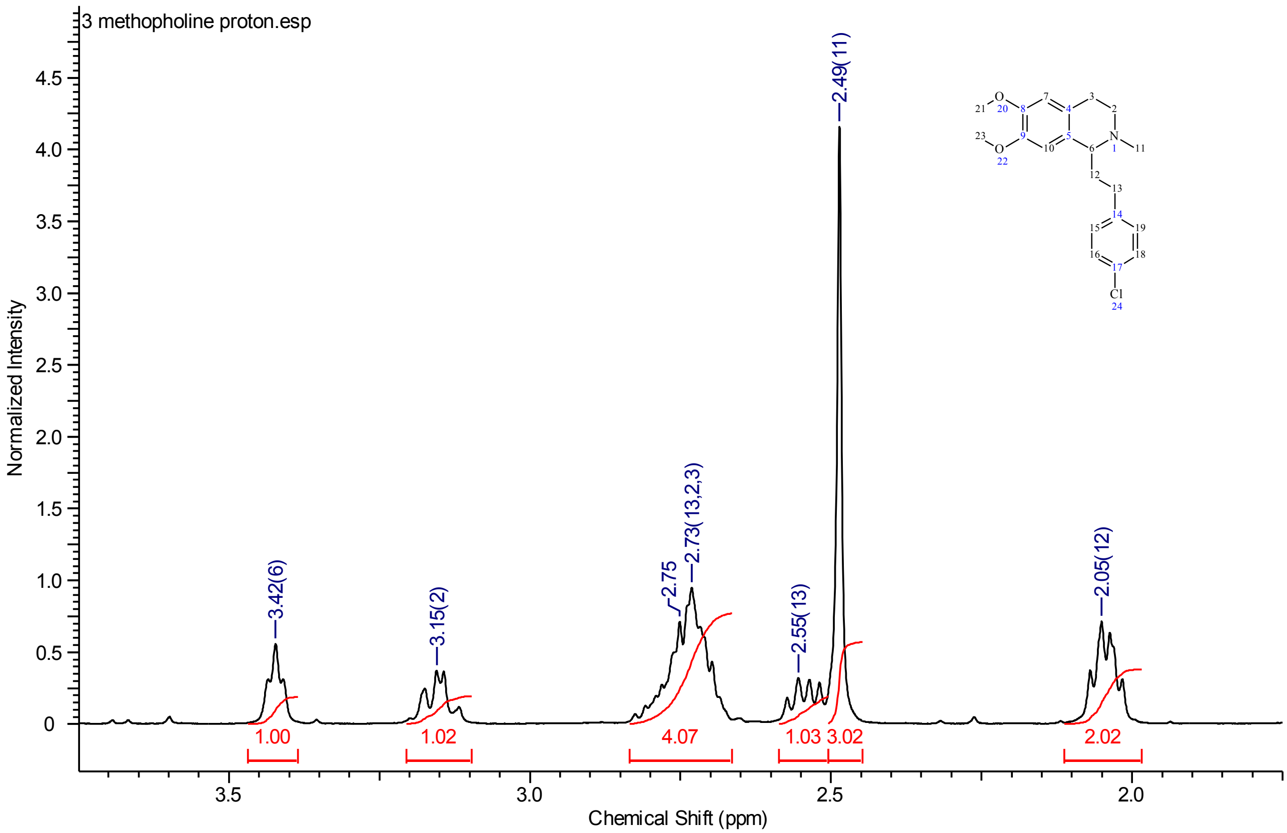
3 methopholine proton.esp
Normalized Intensity
Chemical Shift (ppm)
3.42(6)
3.15(2)
2.75
2.73(13,2,3)
2.55(13)
2.49(11)
2.05(12)
1.00
1.02
4.07
1.03
3.02
2.02

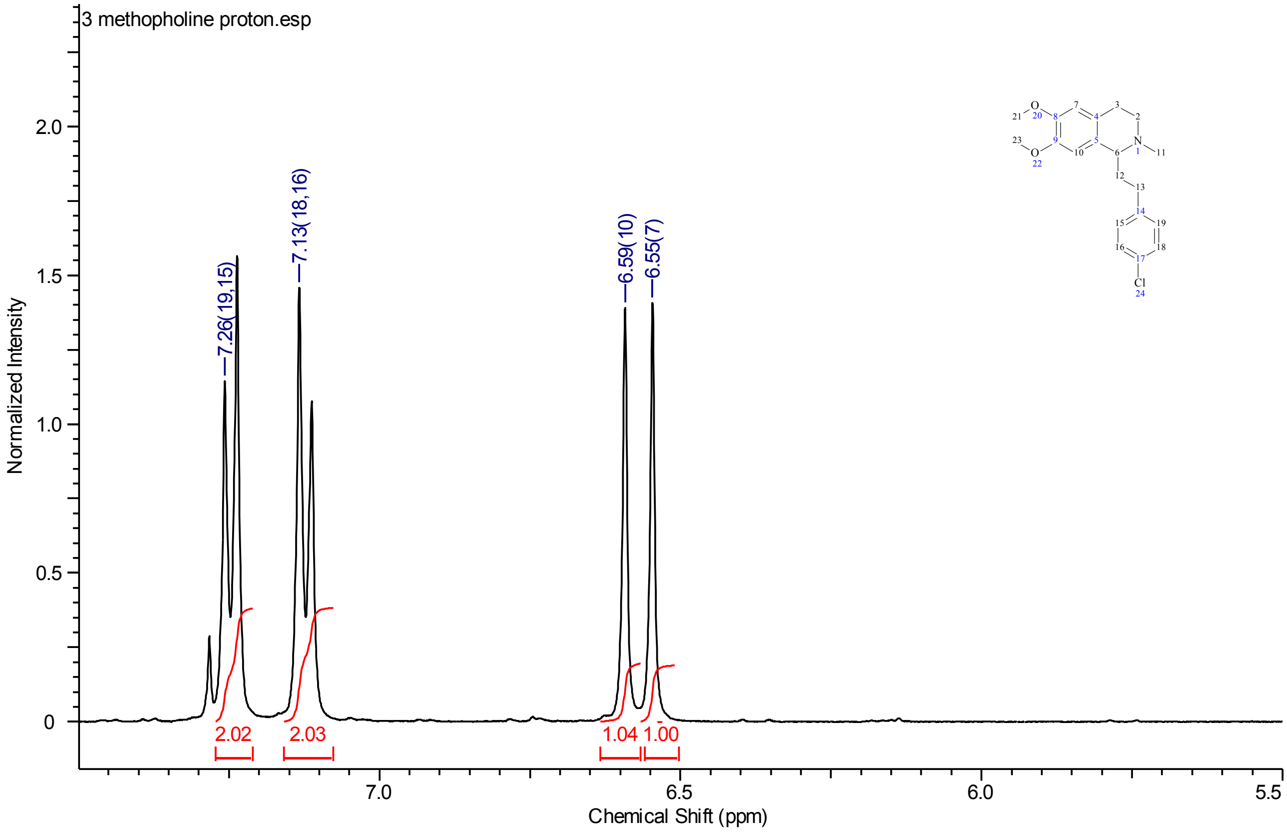
3 methopholine proton.esp
Normalized Intensity
Chemical Shift (ppm)
7.26(19,15)
7.13(18,16)
6.59(10)
6.55(7)
2.02
2.03
1.04
1.00

^{13}C NMR – **3**, methopholine

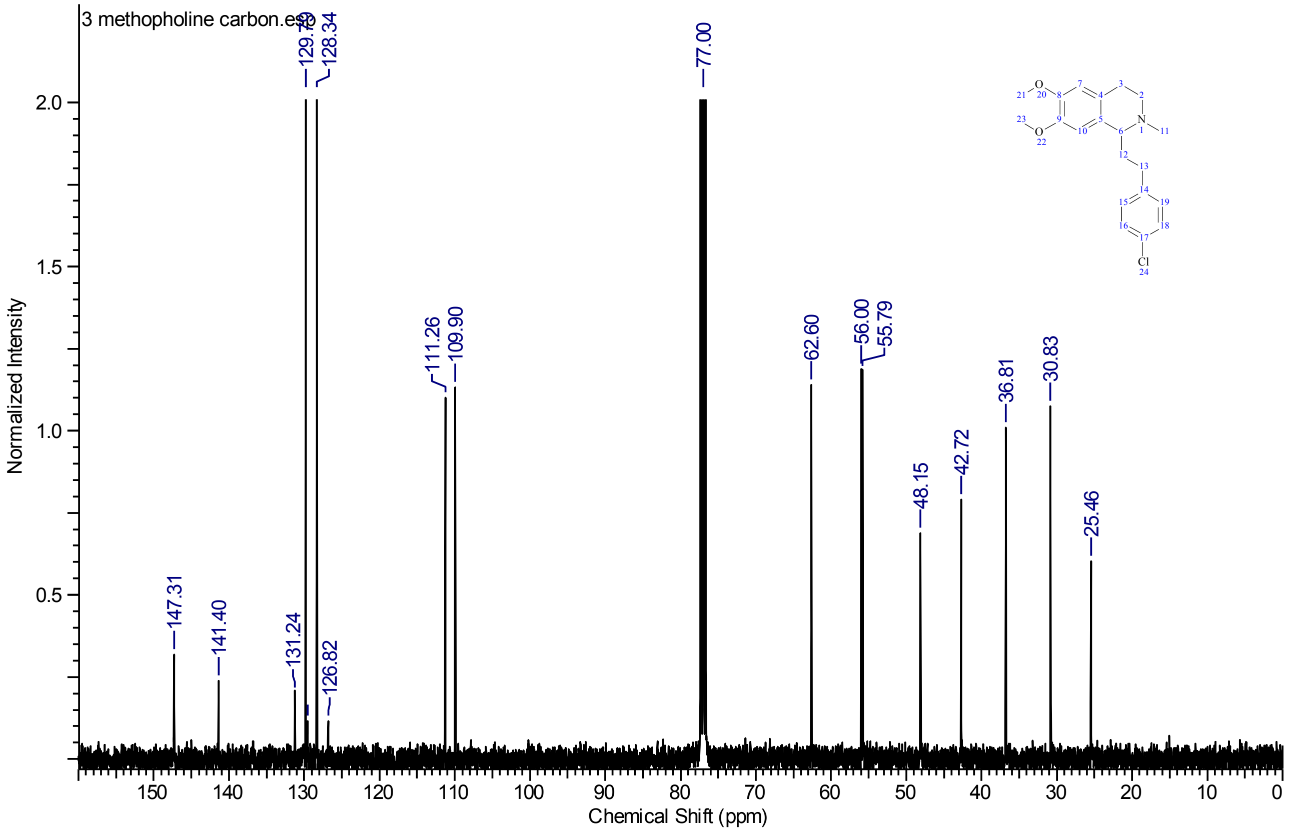

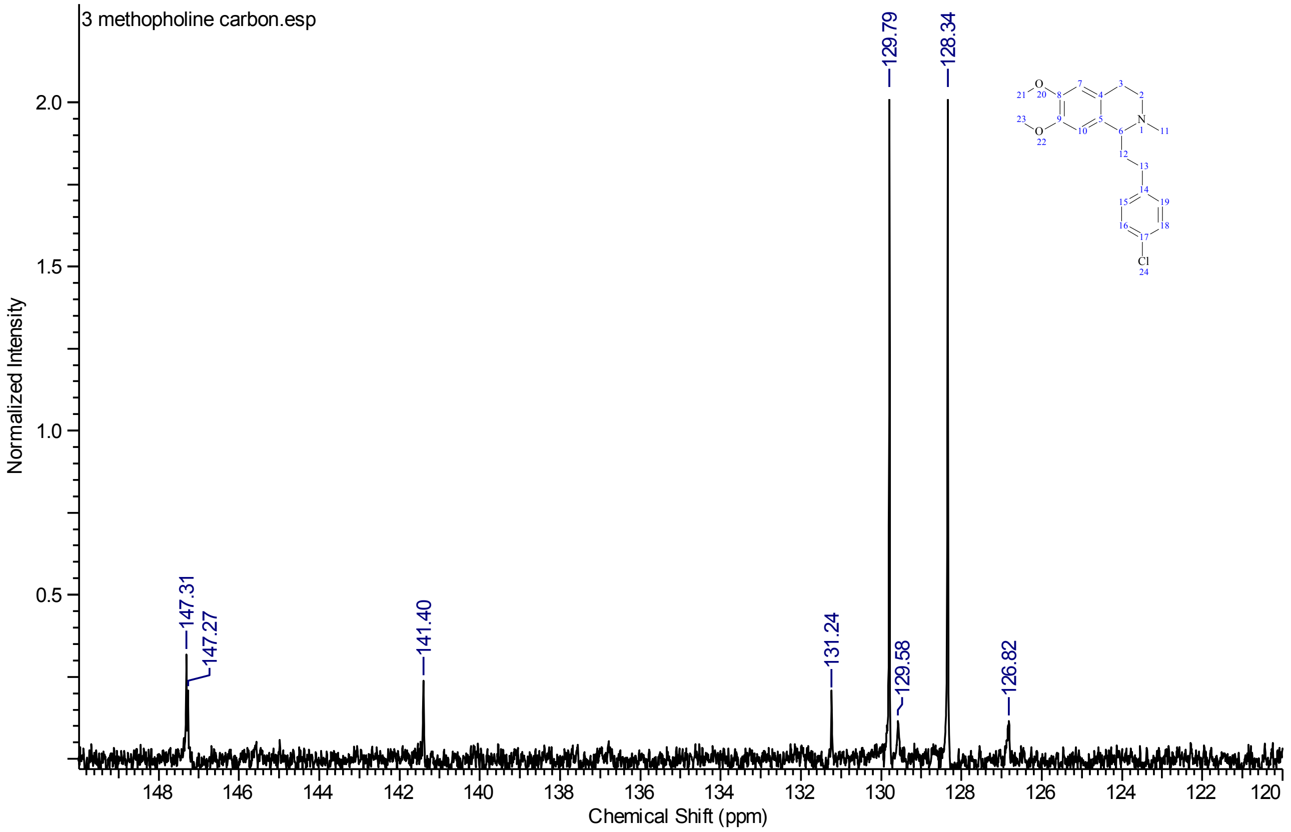

7. Supporting data from which conclusions were drawn

HPLC – Before (left) and after (right) addition of allyl organozinc reagent to **6aj**

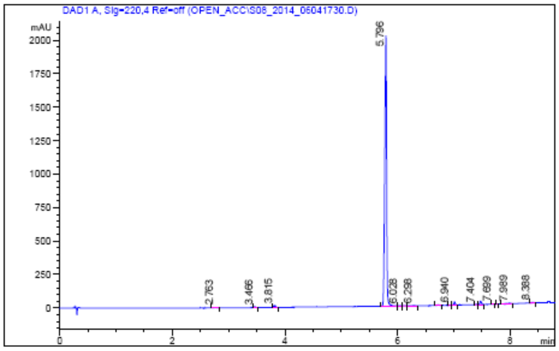

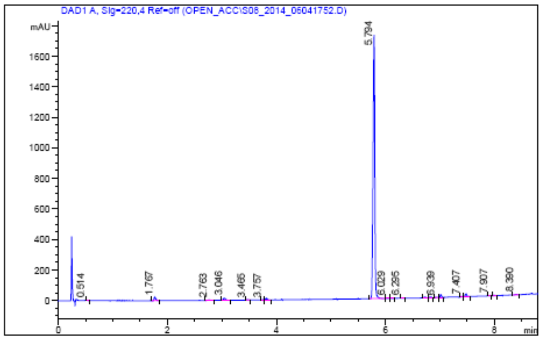

No reaction observed. Therefore it is unlikely that **6aj** and **6ba** are intermediates in formation of **7a**, **7b** and **8**.

^{1}H NMR (crude, in DMSO-d_6) – iminium salt generated from **16a**

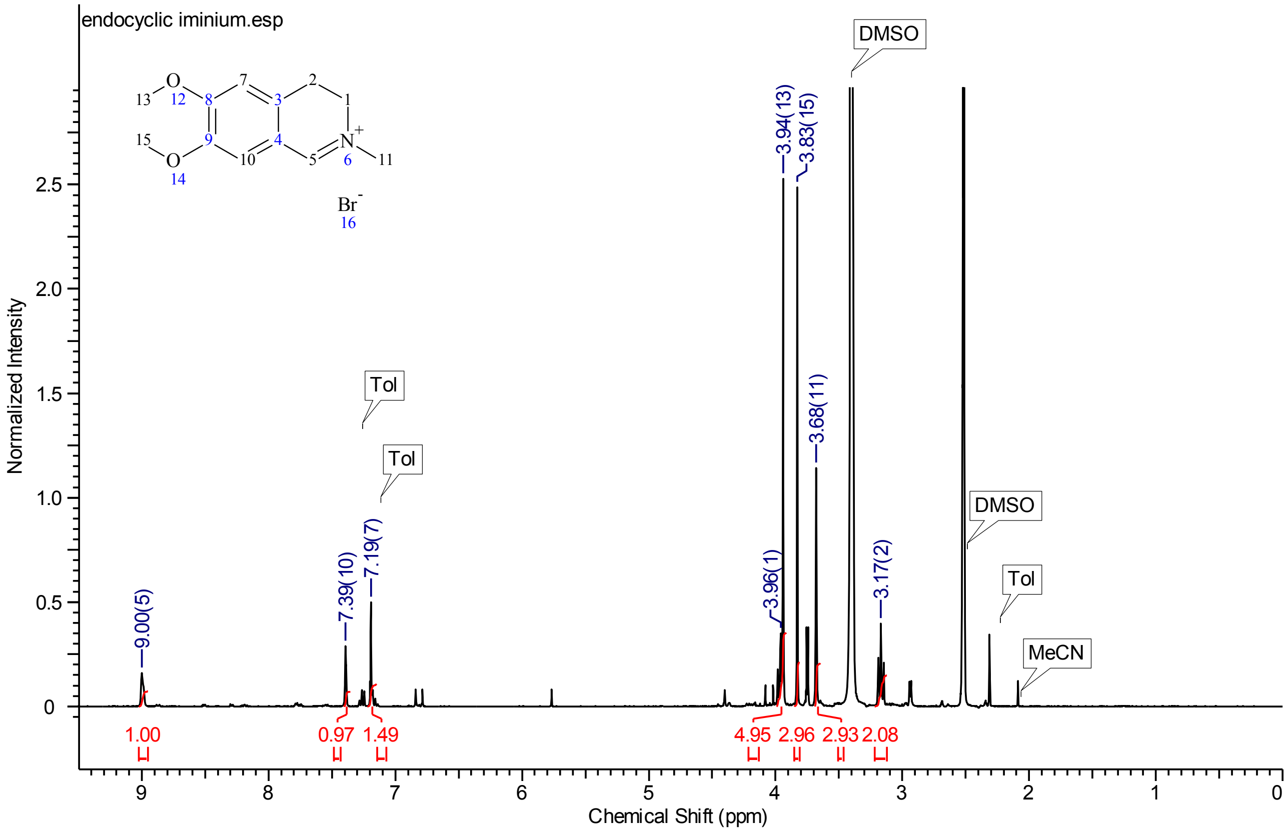

In DMSO-d_6. Singlet at 3.9 ppm (*N*-methyl). Two singlets at 3.9 and 3.8 ppm (2 x OCH_3). Triplets at 3.2 and 4.0 ppm (hidden under singlet at 3.9 ppm) observed (CH_2CH_2N). Toluene and MeCN solvent impurities deemed acceptable for purposes of the following (Grignard addition) step.

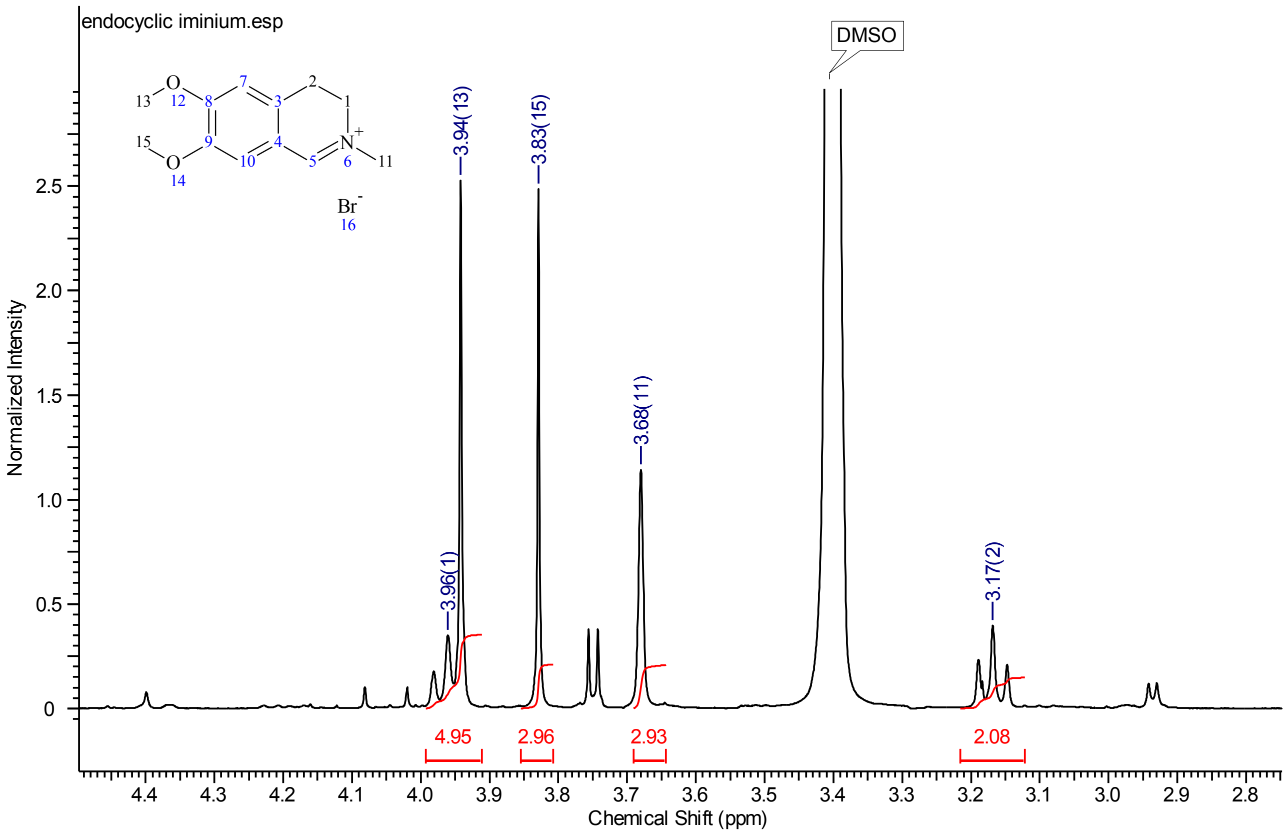

HPLC – Addition of crotonaldehyde to **5a** *via* an enamine intermediate. At 0 h (left) and after stirring for 12 h (right) – ratio of **8b** : **5a** = 2 : 1.

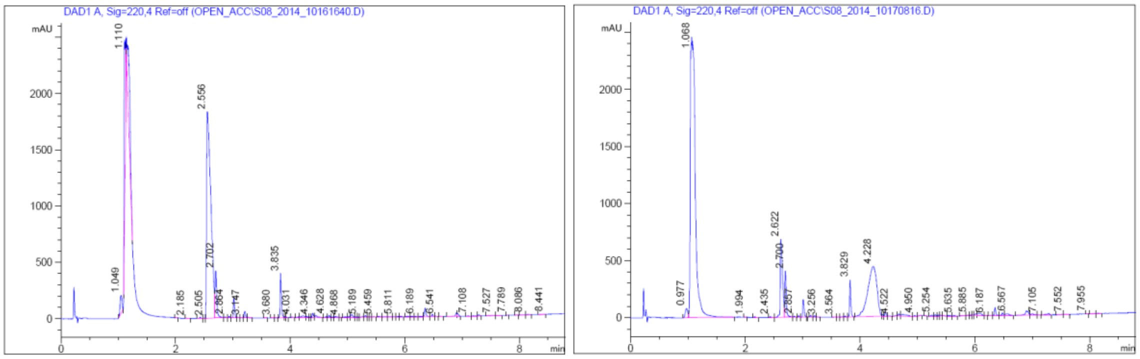

Retention times: crotonaldehyde = 1.1 min, **5a** = 2.6 min, imidazolidinone catalyst = 3.8 min, **8b** = 4.2 min.

8. References

(1) Buckley, B.; Christie, S.; Elsegood, M.; Gillings, C.; Page, P.; Pardoe, W. *Synlett* **2010**, 939.

(2) Stanforth, S. P.; Shawcross, A. P. *J. Heterocycl. Chem.* **1990**, *27*, 367.

(3) Das, B.; Venkateswarlu, K.; Majhi, A.; Siddaiah, V.; Reddy, K. R. *J. Mol. Catal. A Chem.* **2007**, *267*, 30.

(4) Jones, K. M.; Karier, P.; Klussmann, M. *ChemCatChem* **2012**, *4*, 51.

(5) Schrittwieser, J. H.; Resch, V.; Wallner, S.; Lienhart, W.-D.; Sattler, J. H.; Resch, J.; Macheroux, P.; Kroutil, W. *J. Org. Chem.* **2011**, *76*, 6703.

(6) Araki, S.; Shimizu, T.; Johar, P. S.; Jin, S.-J.; Butsugan, Y. *J. Org. Chem.* **1991**, *56*, 2538.

(7) Baslé, O.; Li, C.-J. *Org. Lett.* **2008**, *10*, 3661.

(8) Zhang, Y.; Kindelin, P.; DeSchepper, D.; Zheng, C.; Klumpp, D. *Synthesis* **2006**, *11*, 1775.

(9) Freeman, D. B.; Furst, L.; Condie, A. G.; Stephenson, C. R. J. *Org. Lett.* **2012**, *14*, 94.

(10) Kumaraswamy, G.; Murthy, A. N.; Pitchaiah, A. *J. Org. Chem.* **2010**, *75*, 3916.

(11) Hedley, K. A.; Stanforth, S. P. *Tetrahedron* **1992**, *48*, 743.

(12) Gray, N. M.; Cheng, B. K.; Mick, S. J.; Lair, C. M.; Contreras, P. C. *J. Med. Chem.* **1989**, *32*, 1242.

(13) Valpuesta, M.; Ariza, M.; Díaz, A.; Suau, R. *European J. Org. Chem.* **2010**, 4393.

(14) Richter, H.; Fröhlich, R.; Daniliuc, C.-G.; García Mancheño, O. *Angew. Chem. Int. Ed.* **2012**, *51*, 8656.
